# Supplementary figures and images for: NBR1-mediated selective autophagy of ARF7 modulates root branching (part 1 of 2)
Source: EMBO Rep. 2024 Apr 29;25(6):8. doi: 10.1038/s44319-024-00142-5 (PMC11169494; doi:10.1038/s44319-024-00142-5)

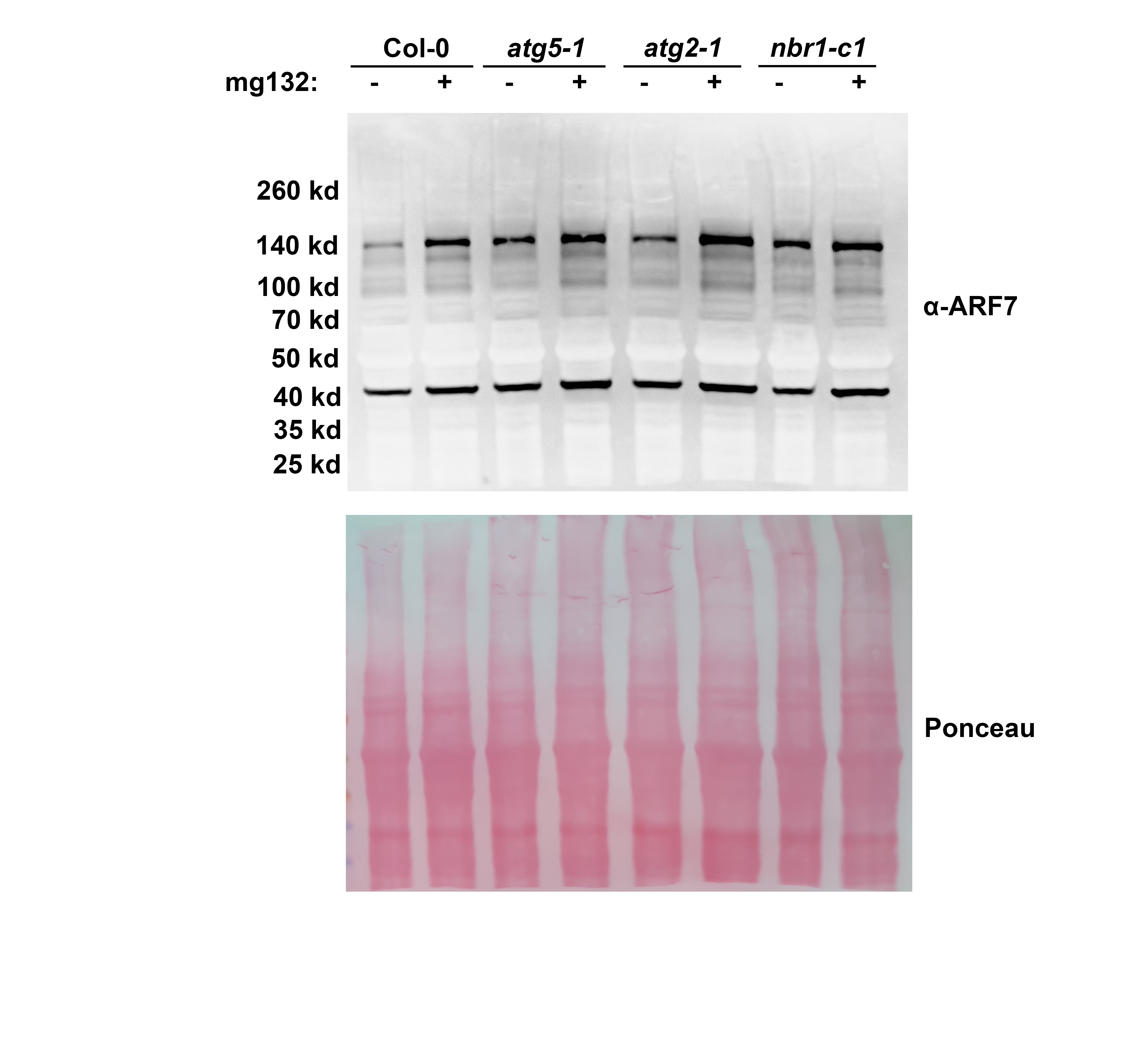

Supplement: Supplementary file 1 — Source data Fig. 1 [file 44319_2024_142_MOESM1_ESM.zip › Figure 1/1A/Western blot mg132 treatment replicate/Western blot mg132 treatment repliate 2.tif]

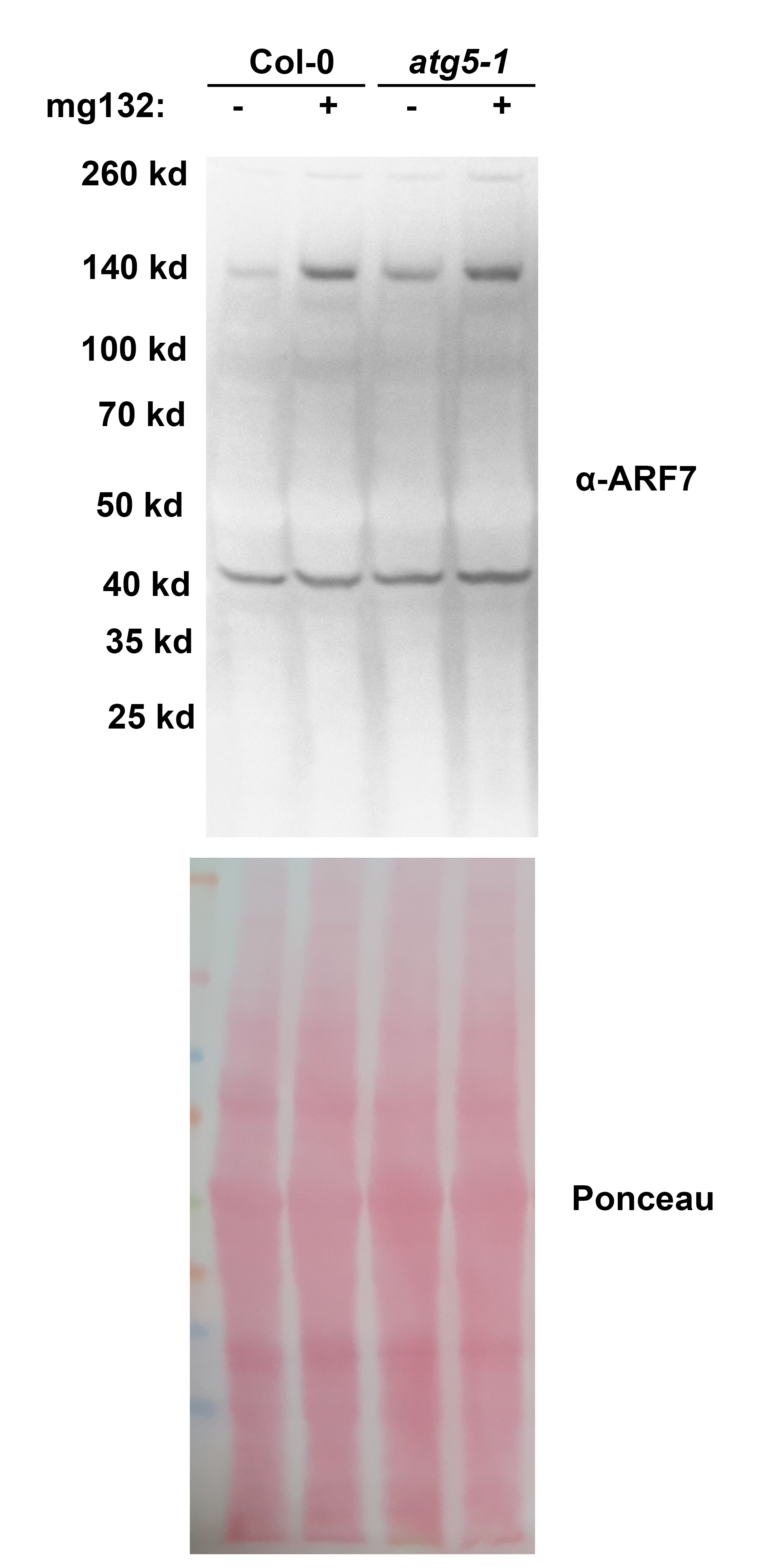

Supplement: Supplementary file 1 — Source data Fig. 1 [file 44319_2024_142_MOESM1_ESM.zip › Figure 1/1A/Western blot mg132 treatment replicate/Western blot mg132 treatment repliate 3.tif]

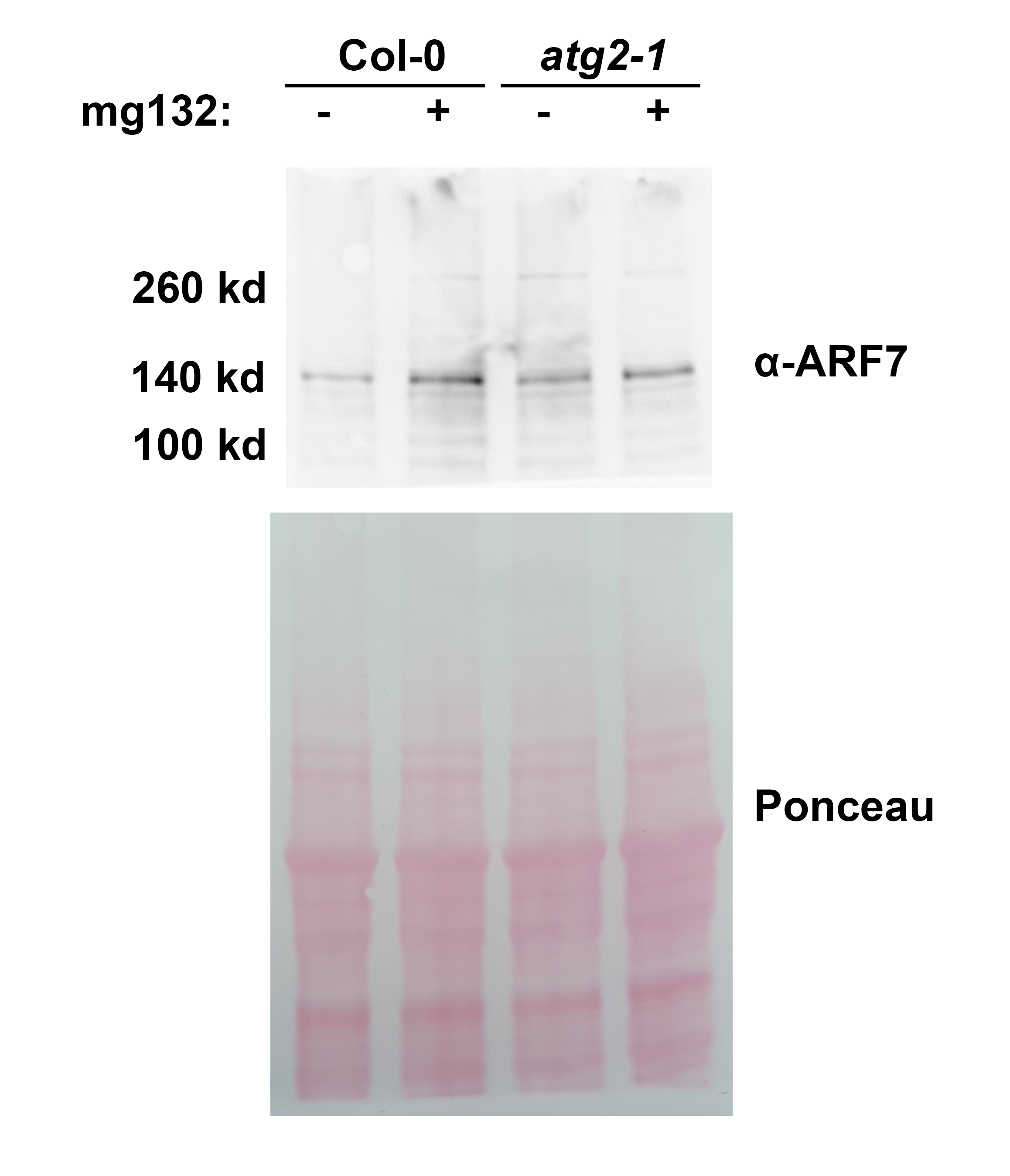

Supplement: Supplementary file 1 — Source data Fig. 1 [file 44319_2024_142_MOESM1_ESM.zip › Figure 1/1A/Western blot mg132 treatment replicate/Western blot mg132 treatment repliate 4.tif]

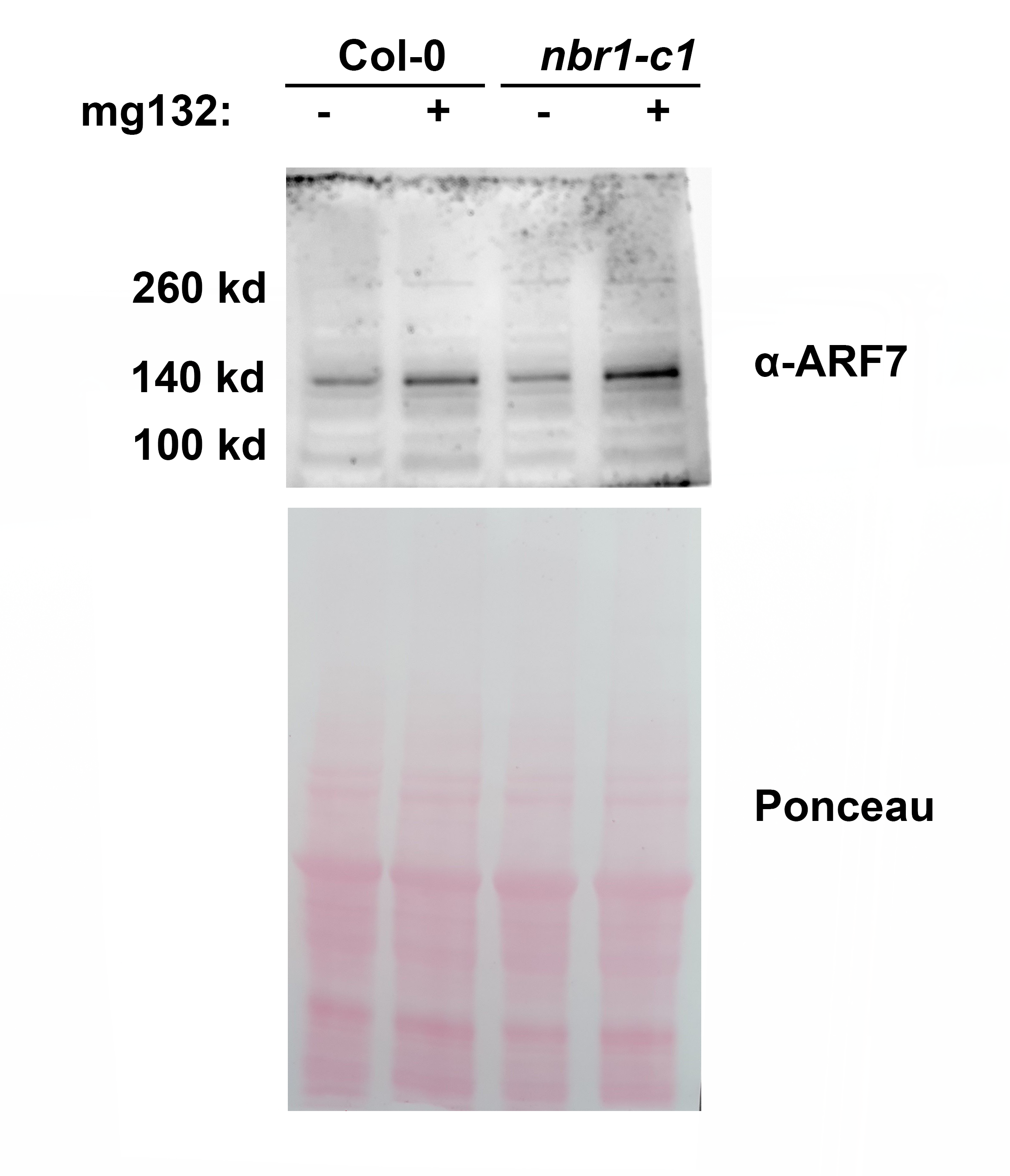

Supplement: Supplementary file 1 — Source data Fig. 1 [file 44319_2024_142_MOESM1_ESM.zip › Figure 1/1A/Western blot mg132 treatment replicate/Western blot mg132 treatment repliate 5.tif]

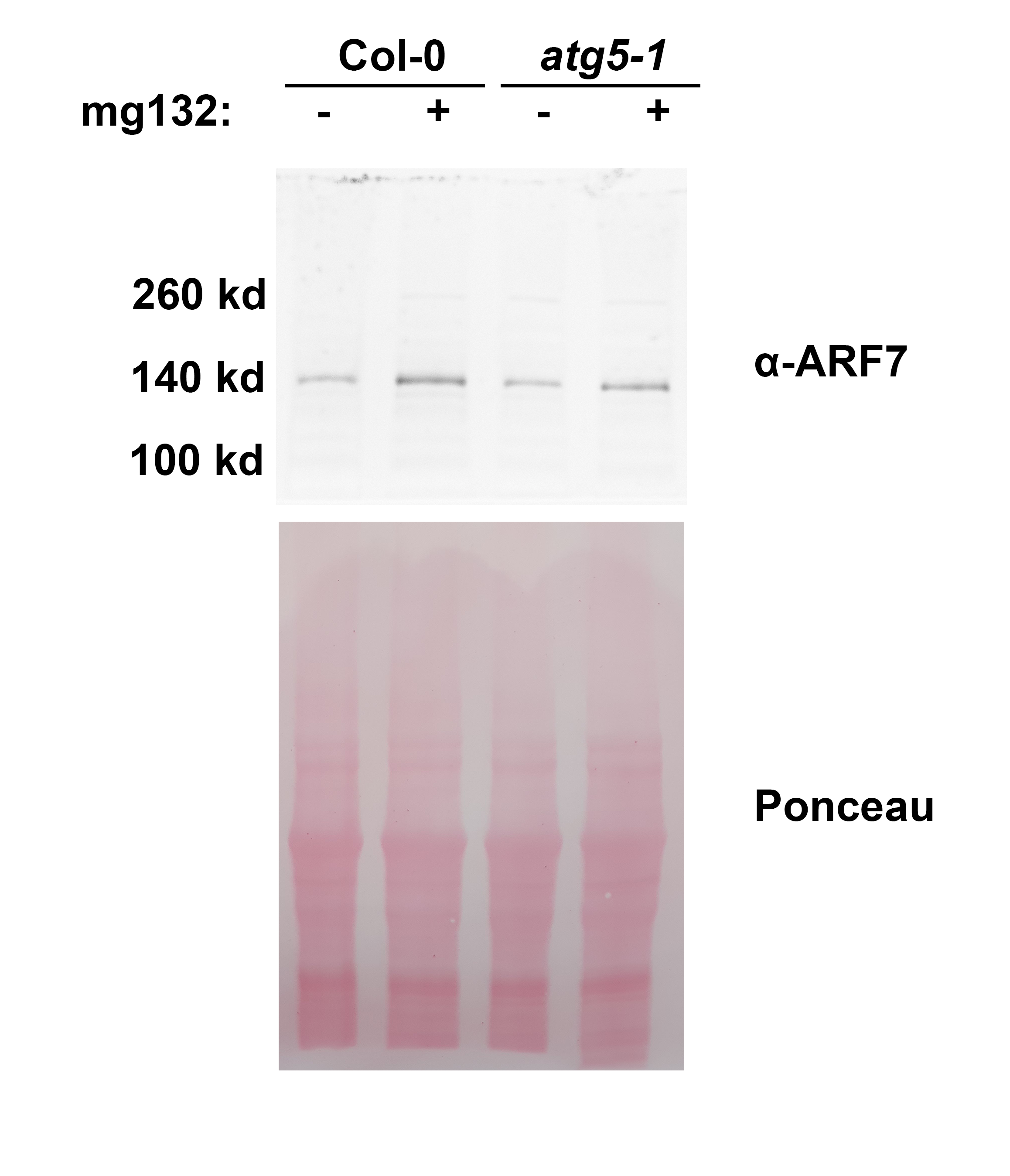

Supplement: Supplementary file 1 — Source data Fig. 1 [file 44319_2024_142_MOESM1_ESM.zip › Figure 1/1A/Western blot mg132 treatment replicate/Western blot mg132 treatment repliate 6.tif]

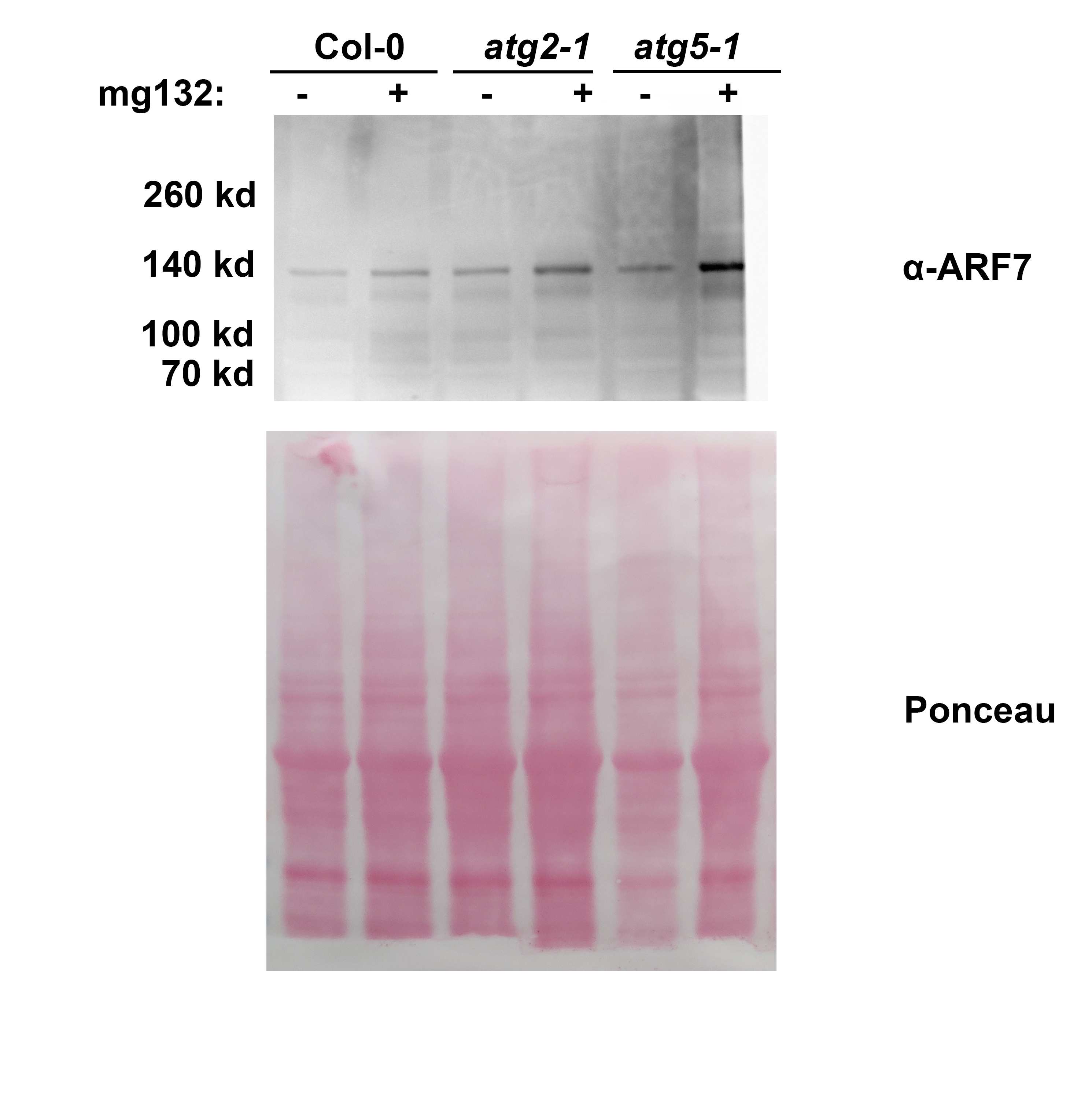

Supplement: Supplementary file 1 — Source data Fig. 1 [file 44319_2024_142_MOESM1_ESM.zip › Figure 1/1A/Western blot mg132 treatment replicate/Western blot mg132 treatment repliate 7.tif]

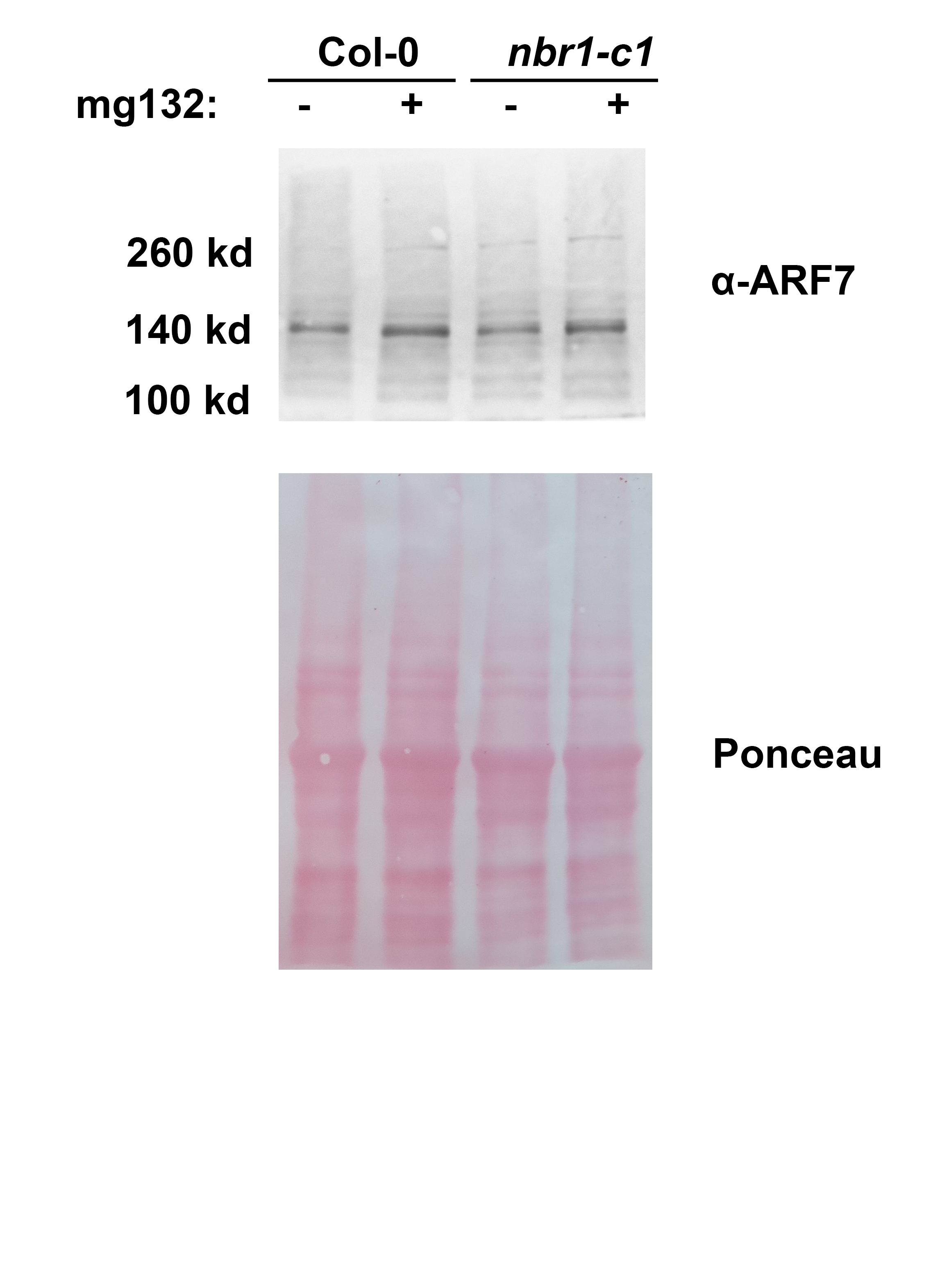

Supplement: Supplementary file 1 — Source data Fig. 1 [file 44319_2024_142_MOESM1_ESM.zip › Figure 1/1A/Western blot mg132 treatment replicate/Western blot mg132 treatment repliate 8.tif]

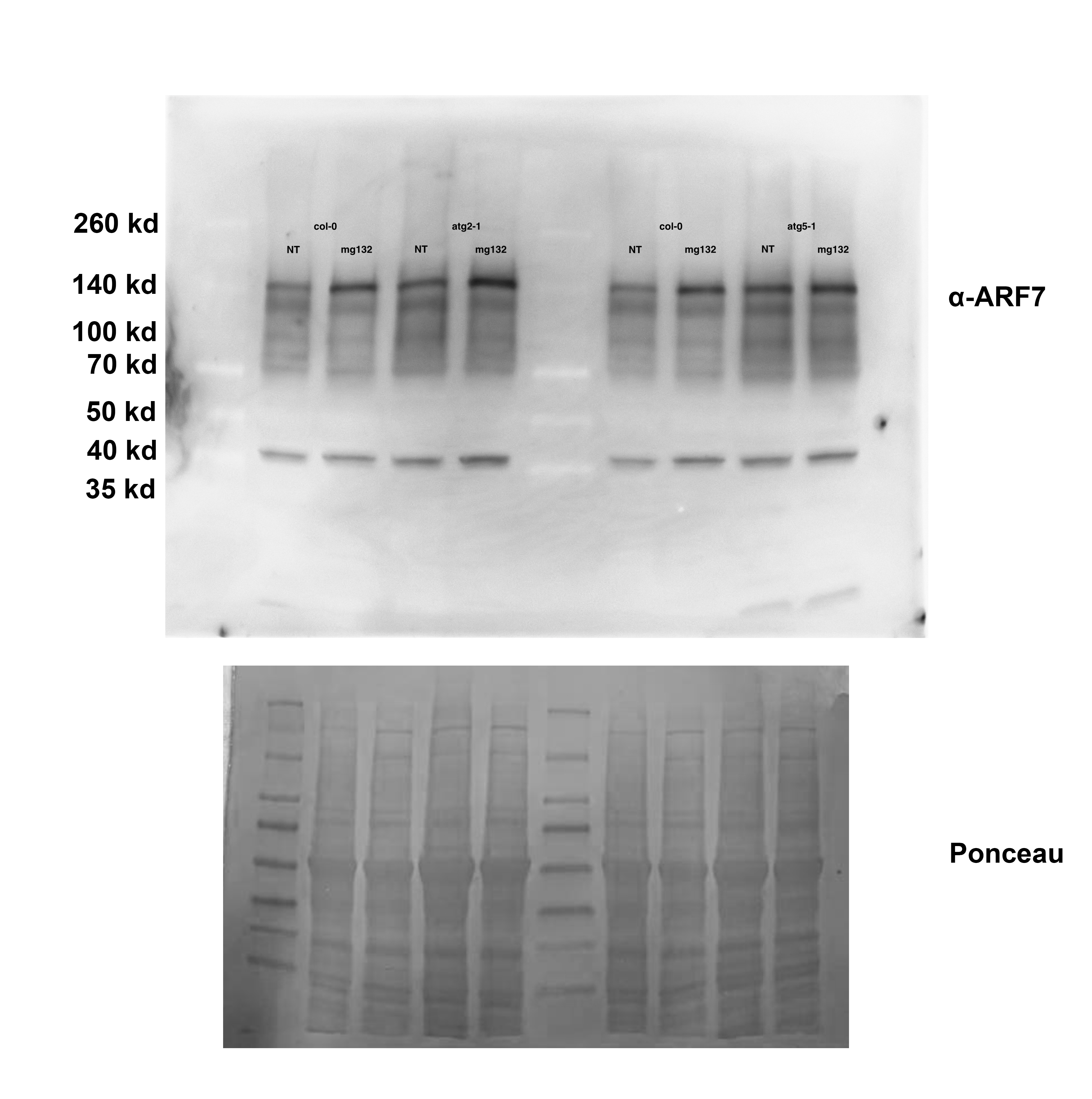

Supplement: Supplementary file 1 — Source data Fig. 1 [file 44319_2024_142_MOESM1_ESM.zip › Figure 1/1A/Western blot mg132 treatment replicate/Western blot mg132 treatment repliate 9.tif]

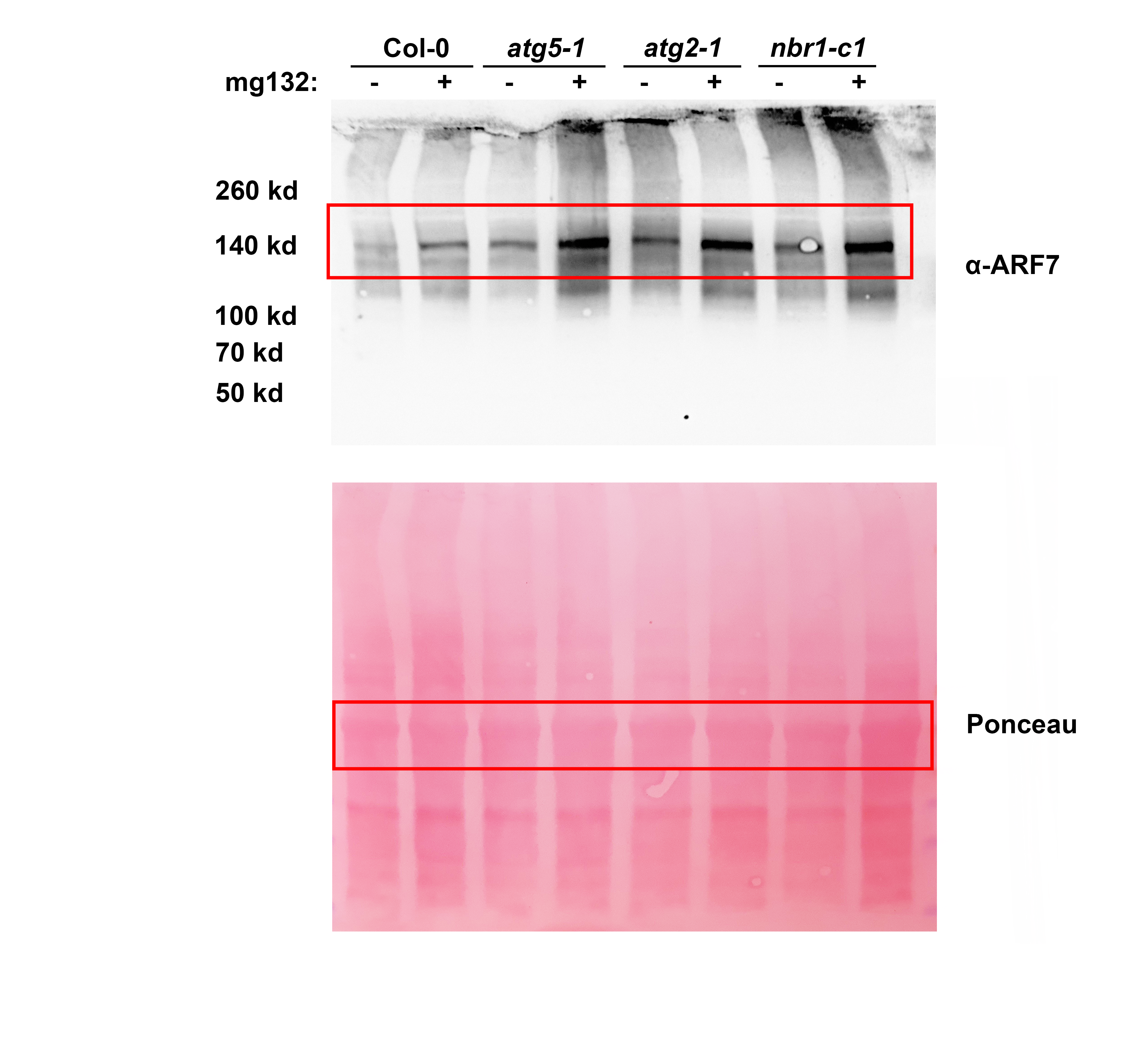

Supplement: Supplementary file 1 — Source data Fig. 1 [file 44319_2024_142_MOESM1_ESM.zip › Figure 1/1A/Western blot mg132 treatment.tif]

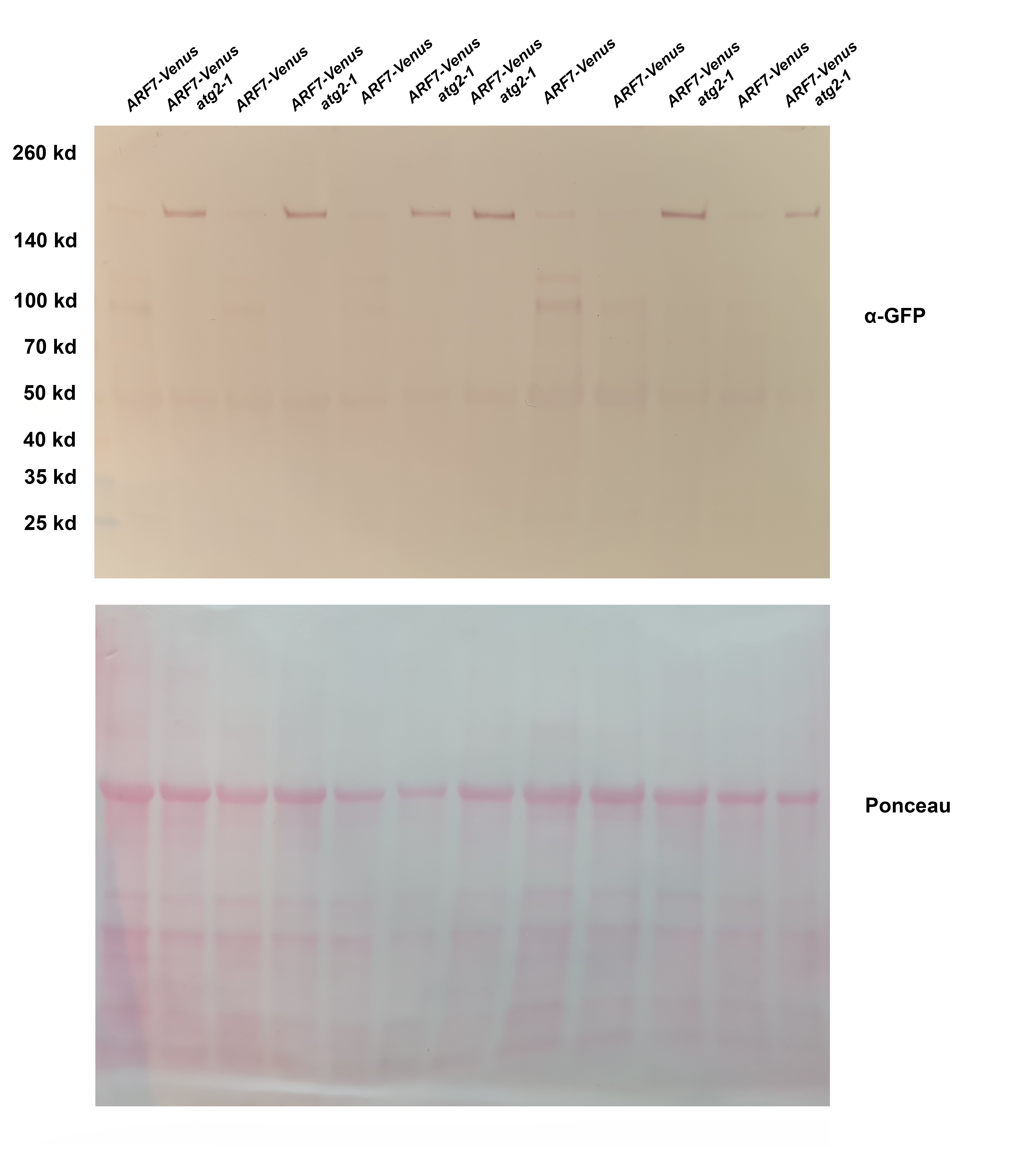

Supplement: Supplementary file 1 — Source data Fig. 1 [file 44319_2024_142_MOESM1_ESM.zip › Figure 1/1C/Western blot ARF7-Venus replicate/Western blot ARF7-Venus replicate.tif]

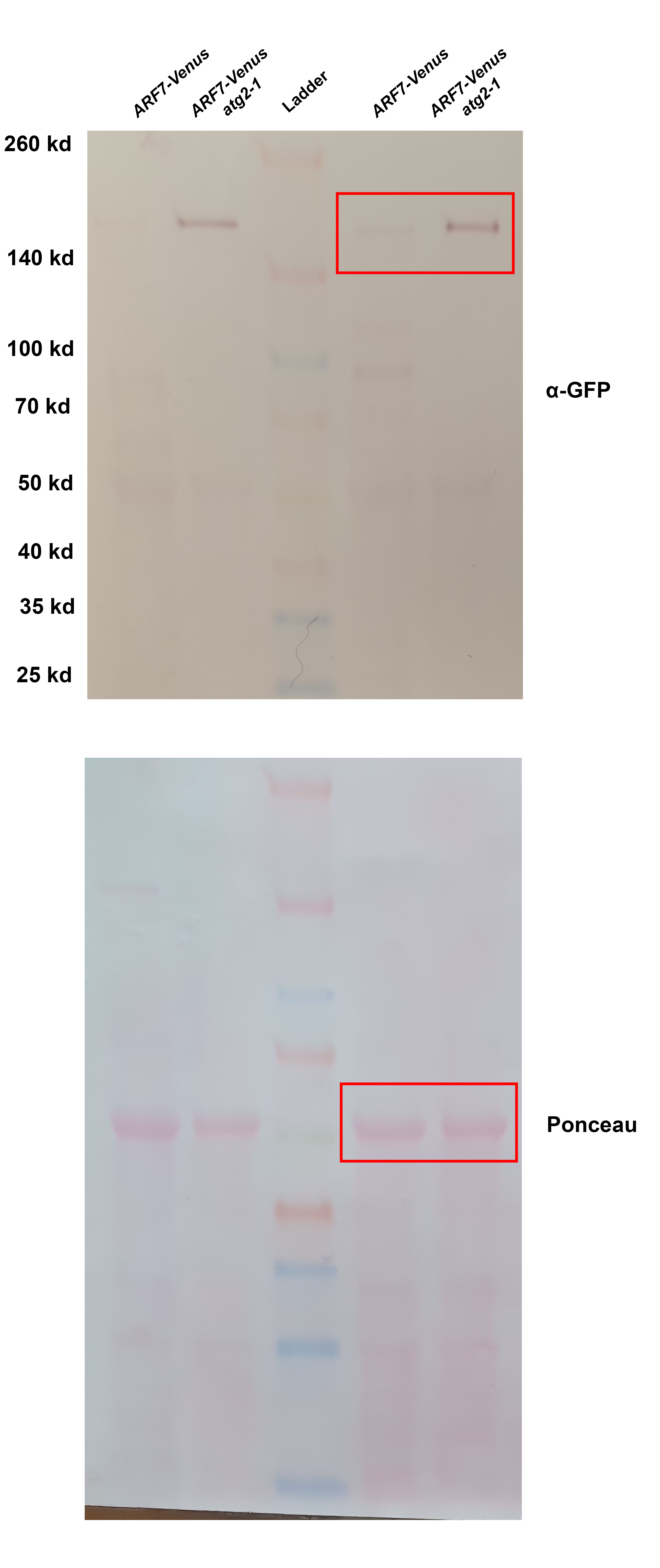

Supplement: Supplementary file 1 — Source data Fig. 1 [file 44319_2024_142_MOESM1_ESM.zip › Figure 1/1C/Western blot ARF7-Venus.tif]

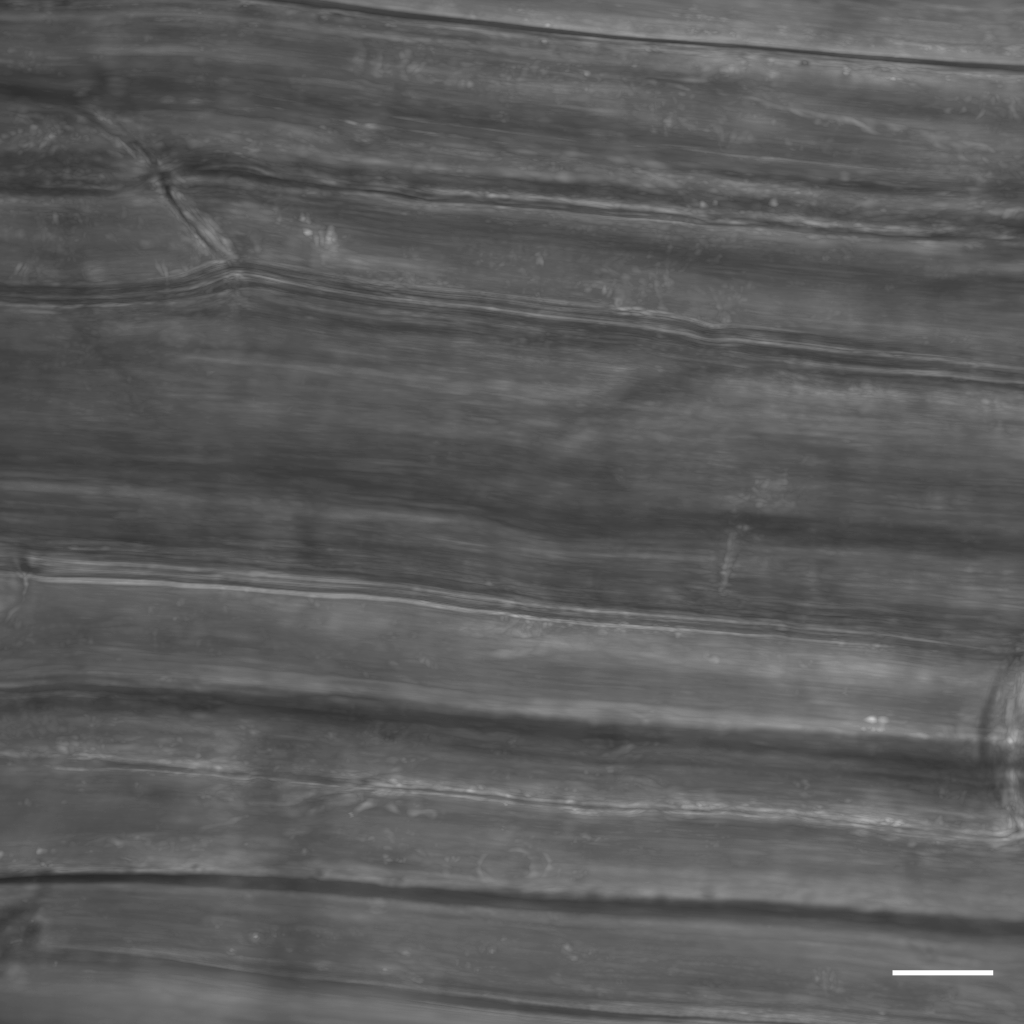

Supplement: Supplementary file 1 — Source data Fig. 1 [file 44319_2024_142_MOESM1_ESM.zip › Figure 1/1D/1D Mature Bright-field.tif]

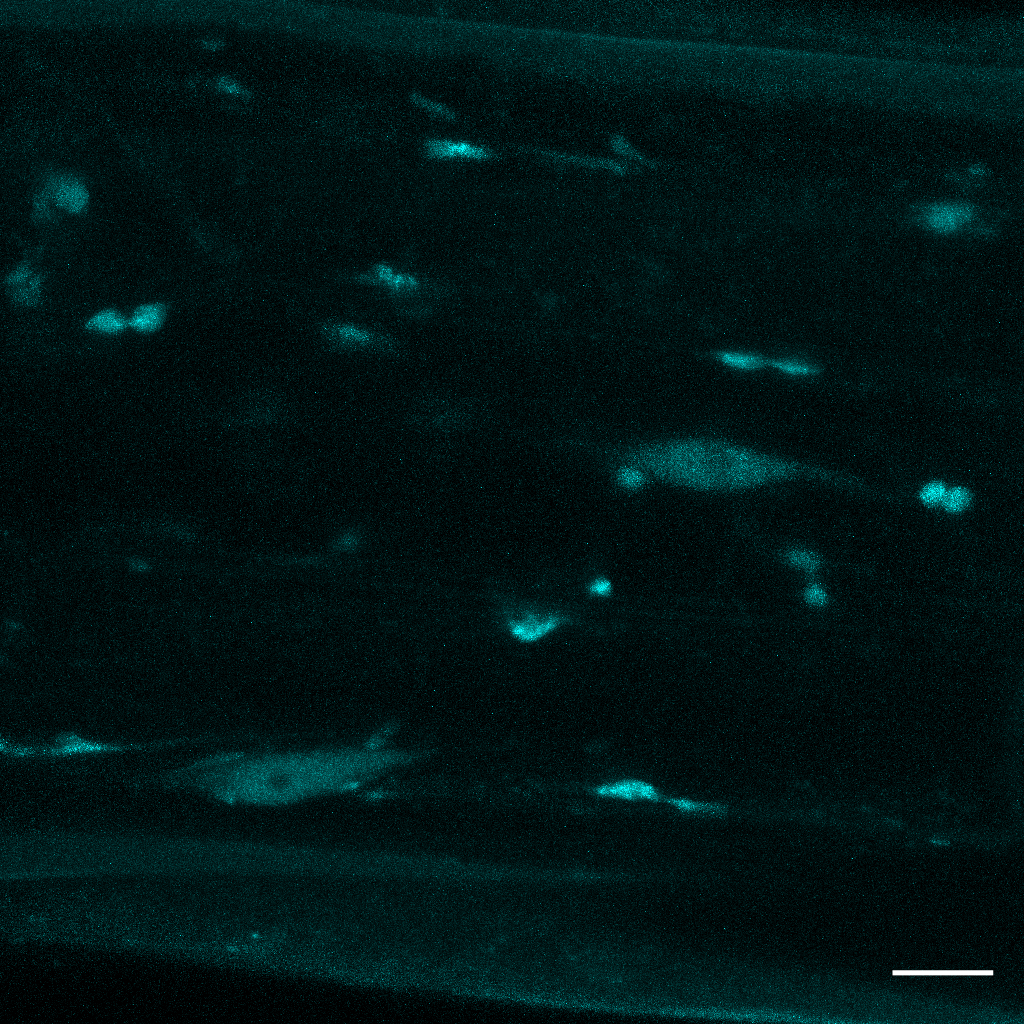

Supplement: Supplementary file 1 — Source data Fig. 1 [file 44319_2024_142_MOESM1_ESM.zip › Figure 1/1D/1D Mature Venus.tif]

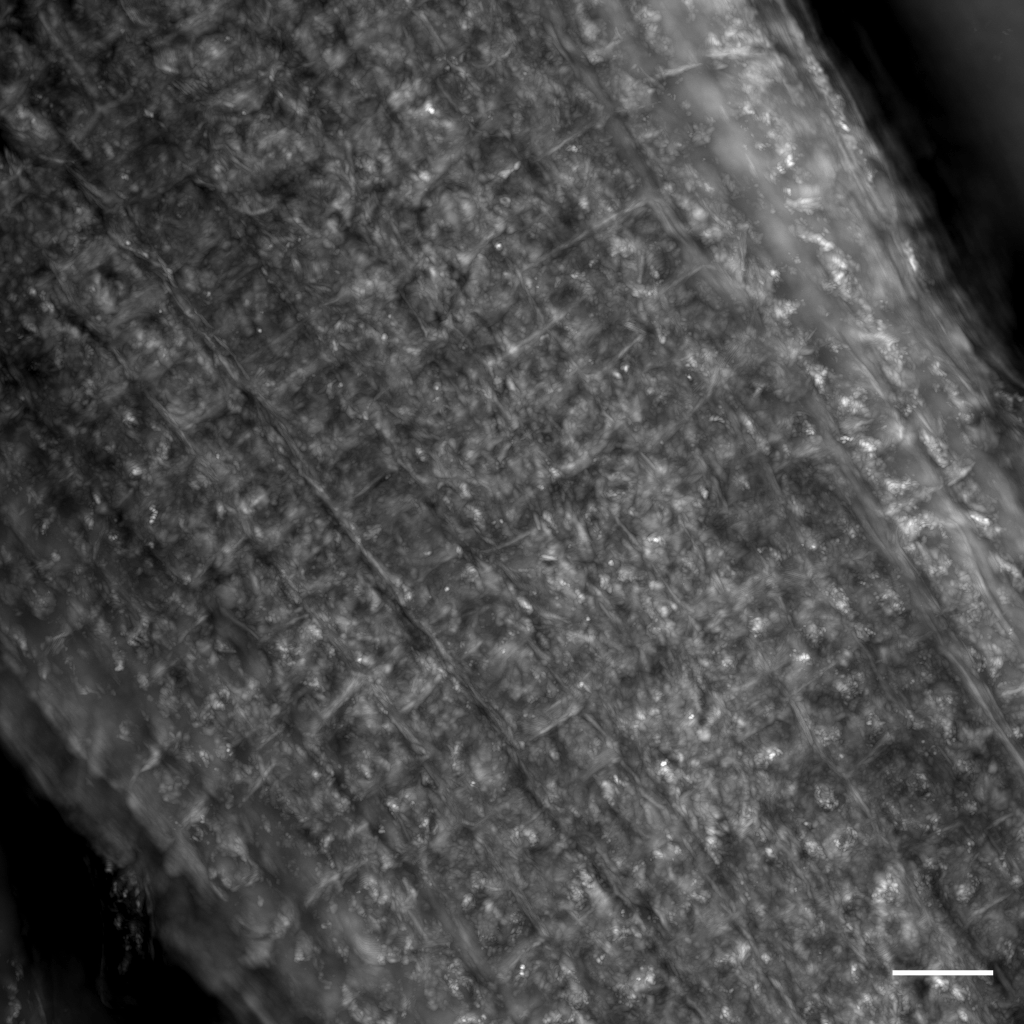

Supplement: Supplementary file 1 — Source data Fig. 1 [file 44319_2024_142_MOESM1_ESM.zip › Figure 1/1D/1D Meristem Bright-field.tif]

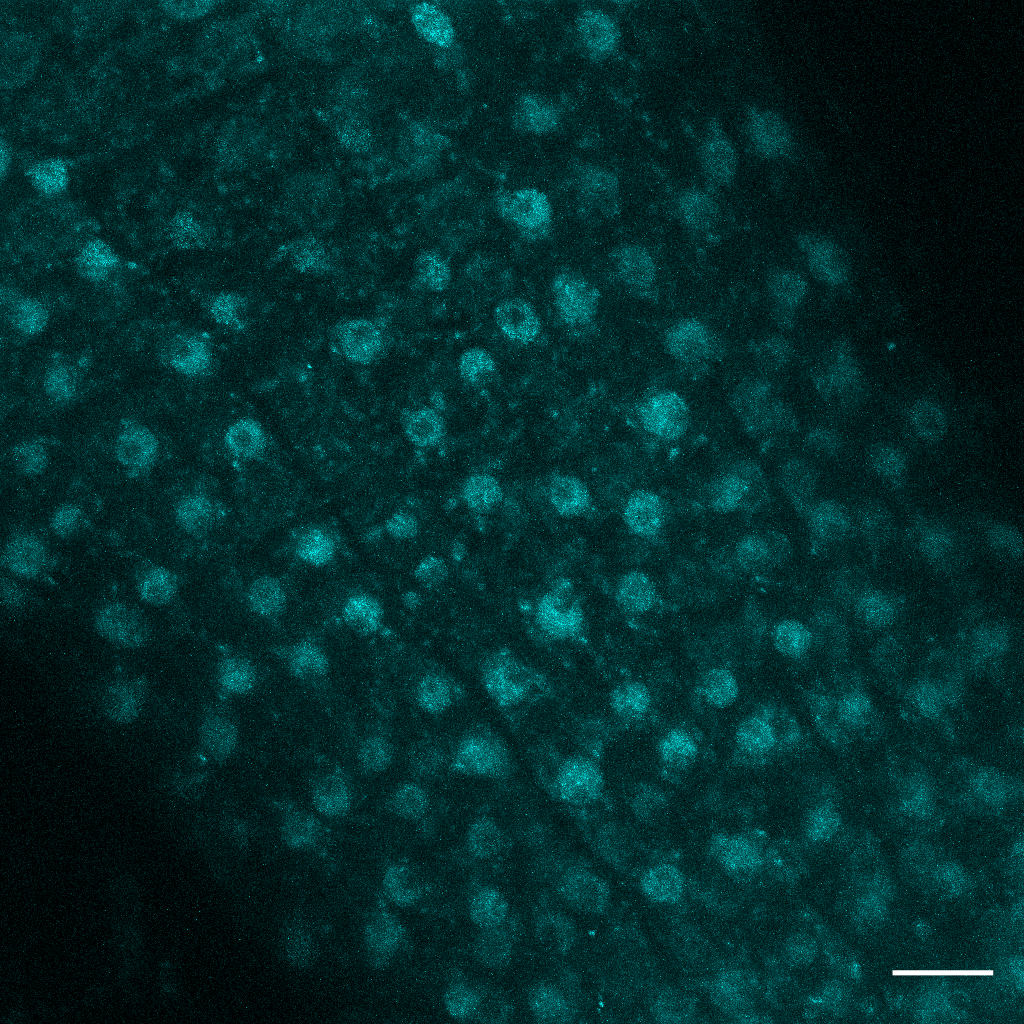

Supplement: Supplementary file 1 — Source data Fig. 1 [file 44319_2024_142_MOESM1_ESM.zip › Figure 1/1D/1D Meristem Venus.tif]

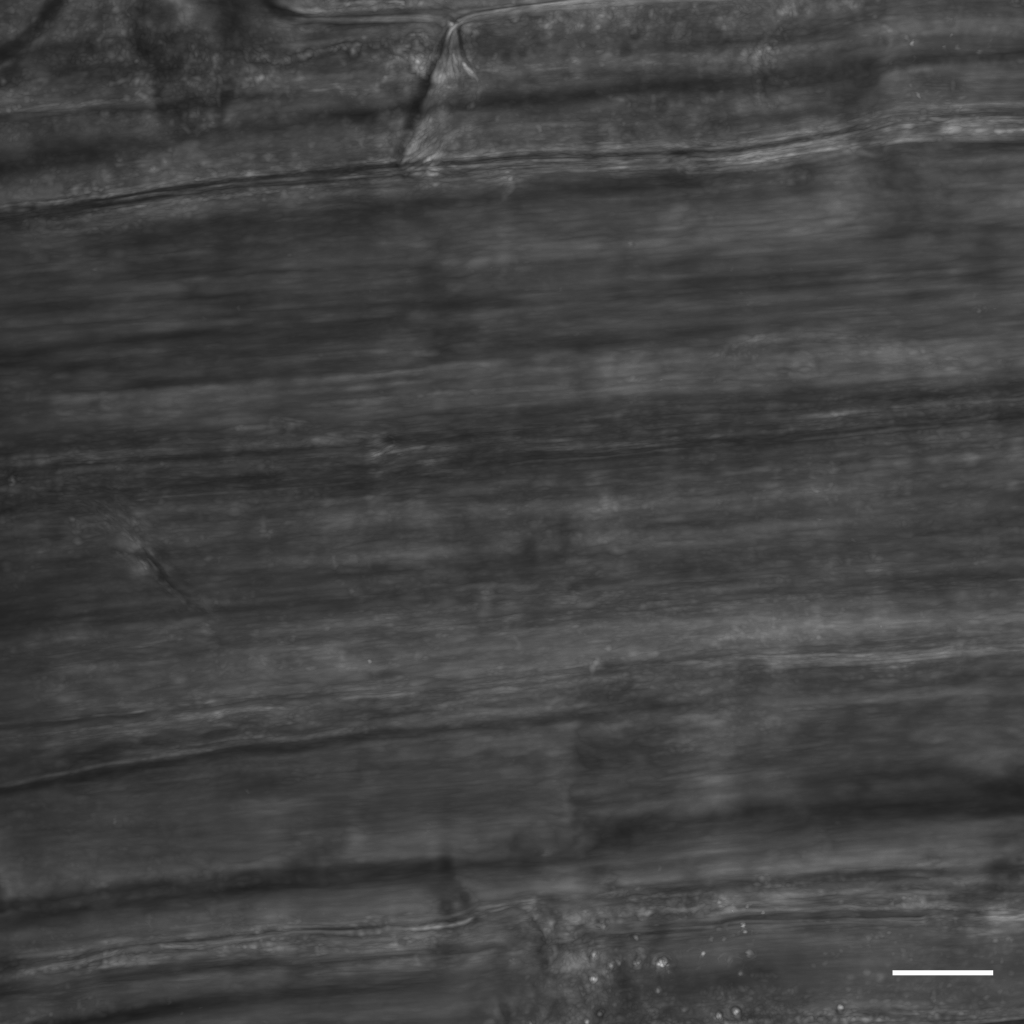

Supplement: Supplementary file 1 — Source data Fig. 1 [file 44319_2024_142_MOESM1_ESM.zip › Figure 1/1E/1E Mature Bright-field.tif]

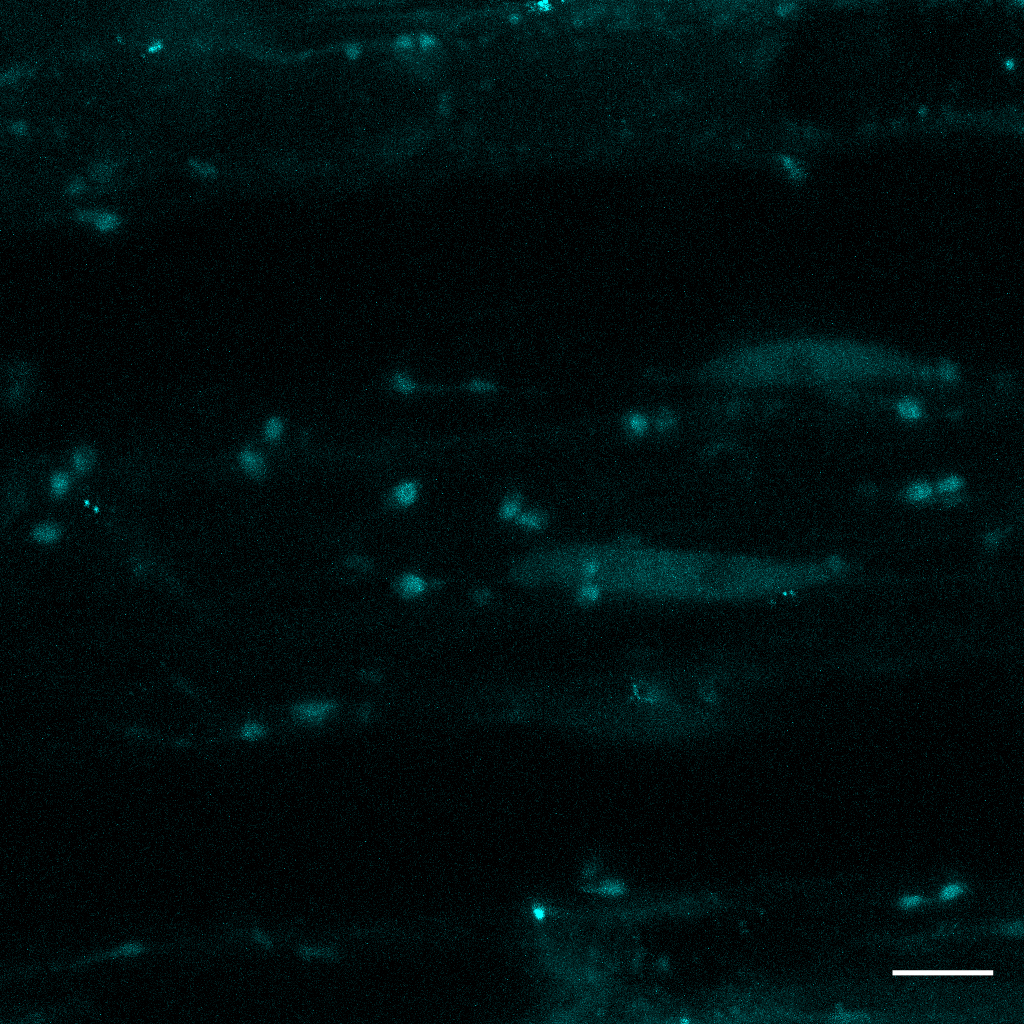

Supplement: Supplementary file 1 — Source data Fig. 1 [file 44319_2024_142_MOESM1_ESM.zip › Figure 1/1E/1E Mature Venus.tif]

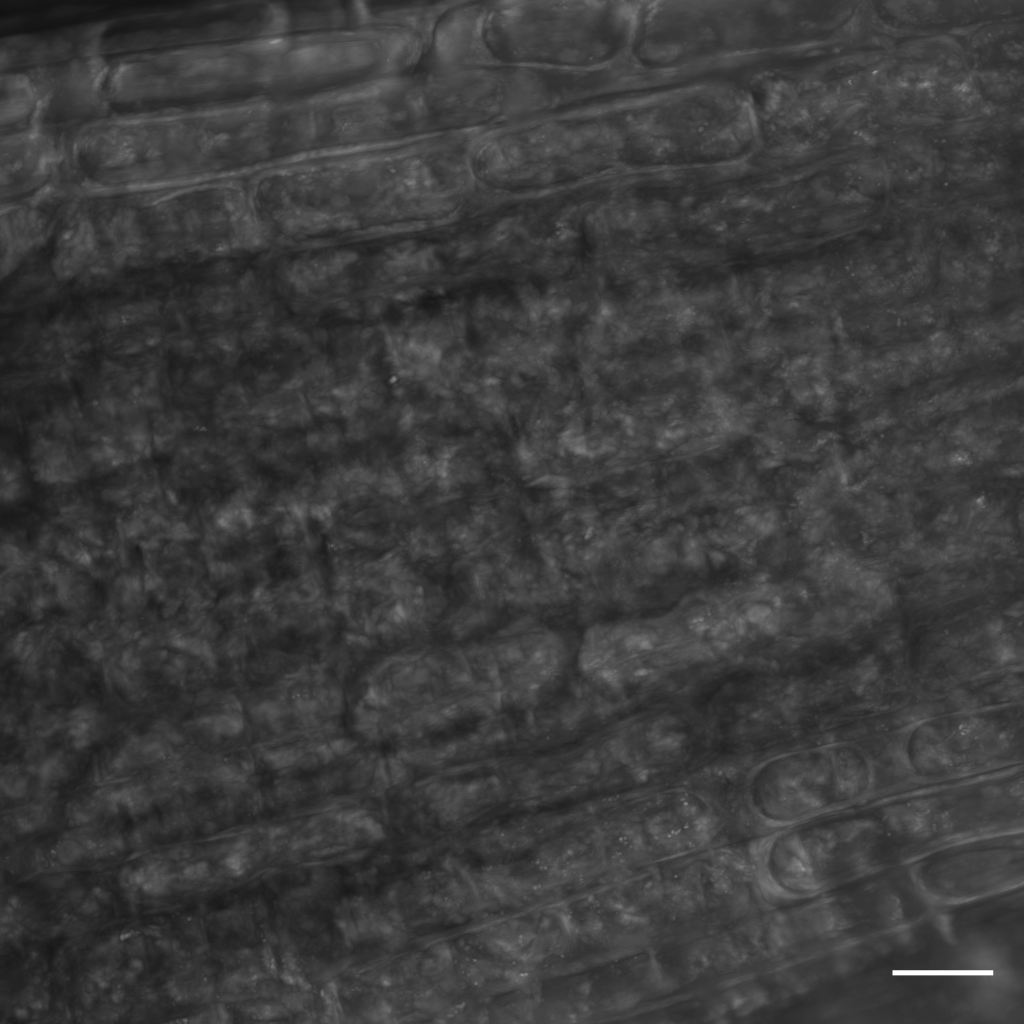

Supplement: Supplementary file 1 — Source data Fig. 1 [file 44319_2024_142_MOESM1_ESM.zip › Figure 1/1E/1E Meristem Bright-field.tif]

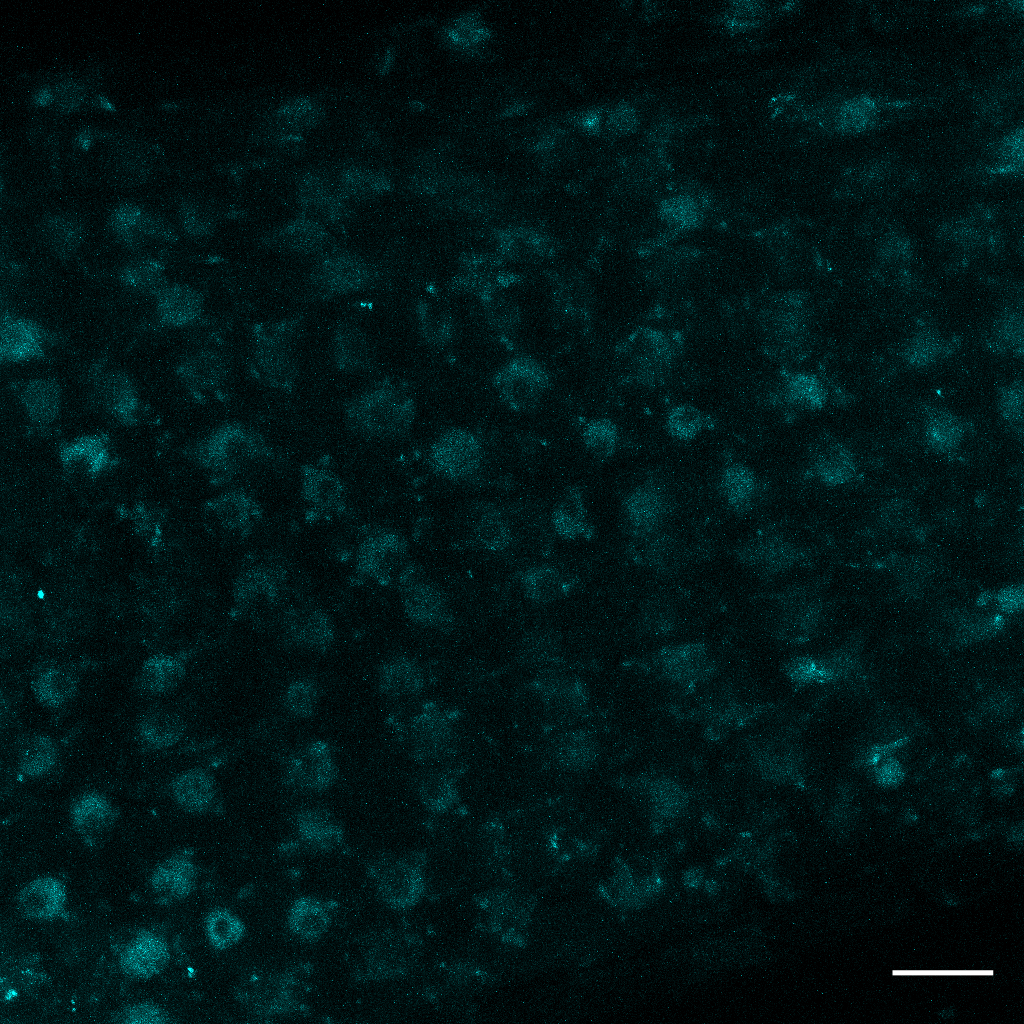

Supplement: Supplementary file 1 — Source data Fig. 1 [file 44319_2024_142_MOESM1_ESM.zip › Figure 1/1E/1E Meristem Venus.tif]

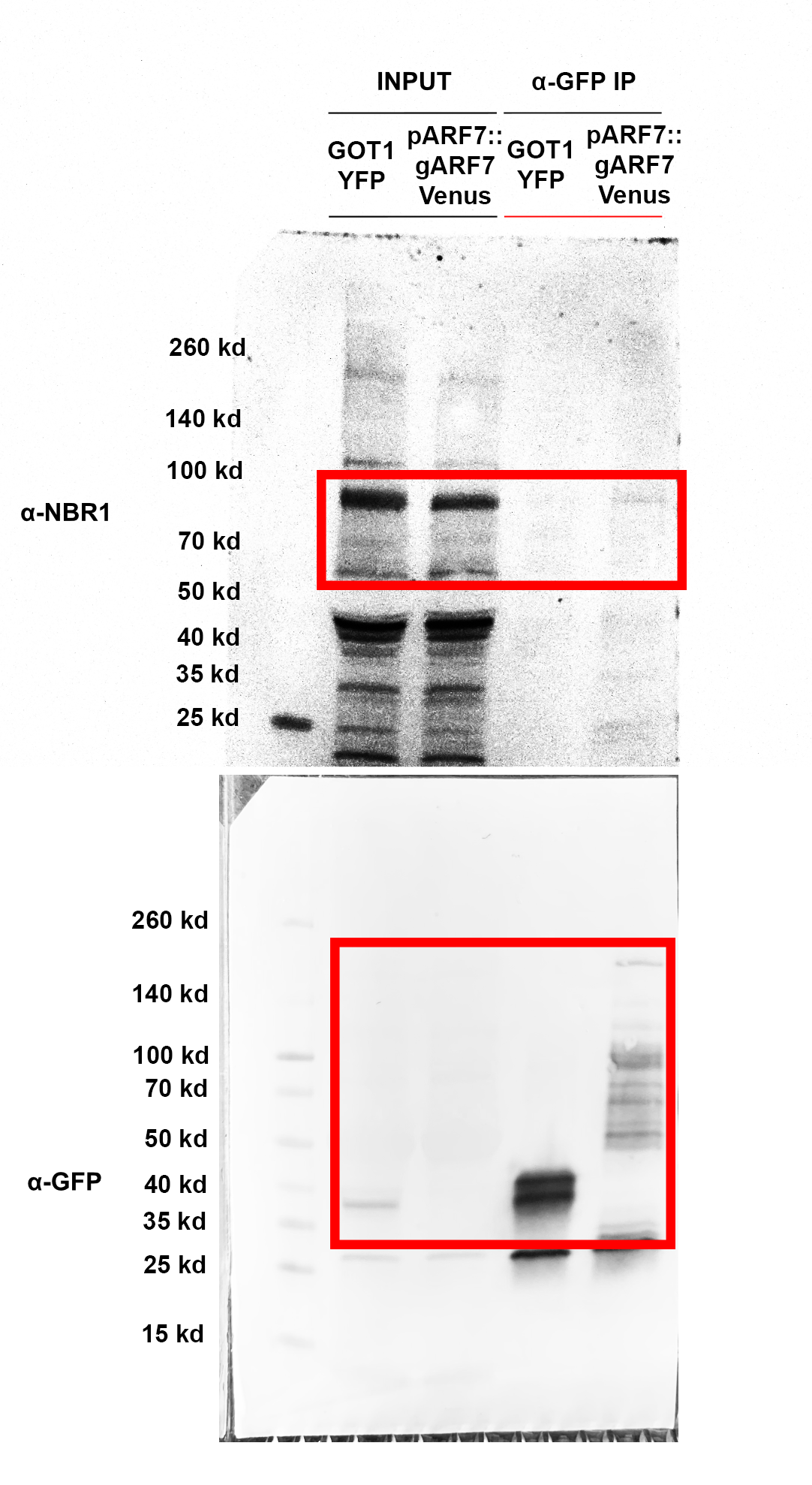

Supplement: Supplementary file 2 — Source data Fig. 2 [file 44319_2024_142_MOESM2_ESM.zip › Figure 2/2A/IP NBR1.tif]

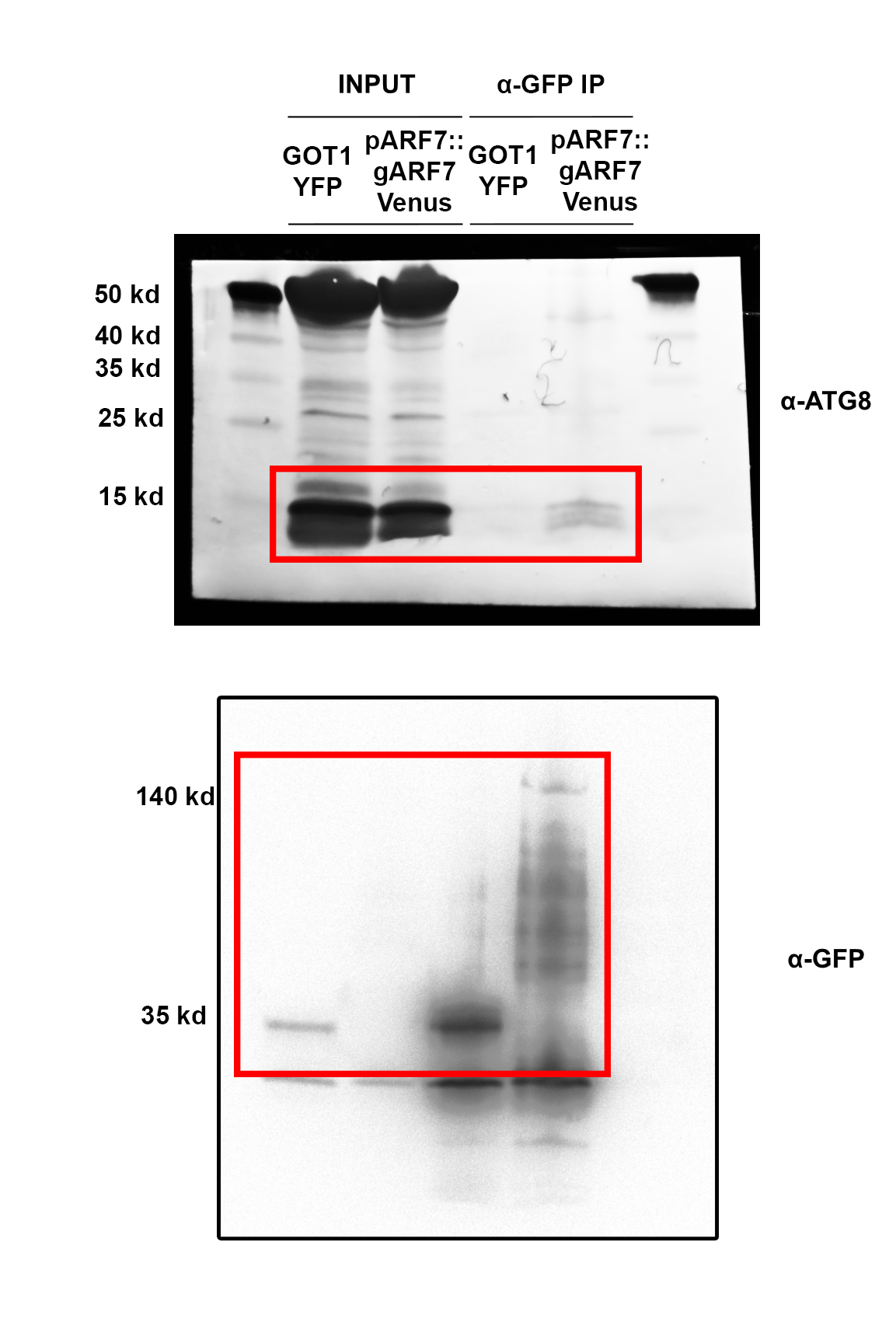

Supplement: Supplementary file 2 — Source data Fig. 2 [file 44319_2024_142_MOESM2_ESM.zip › Figure 2/2B/IP ATG8.tif]

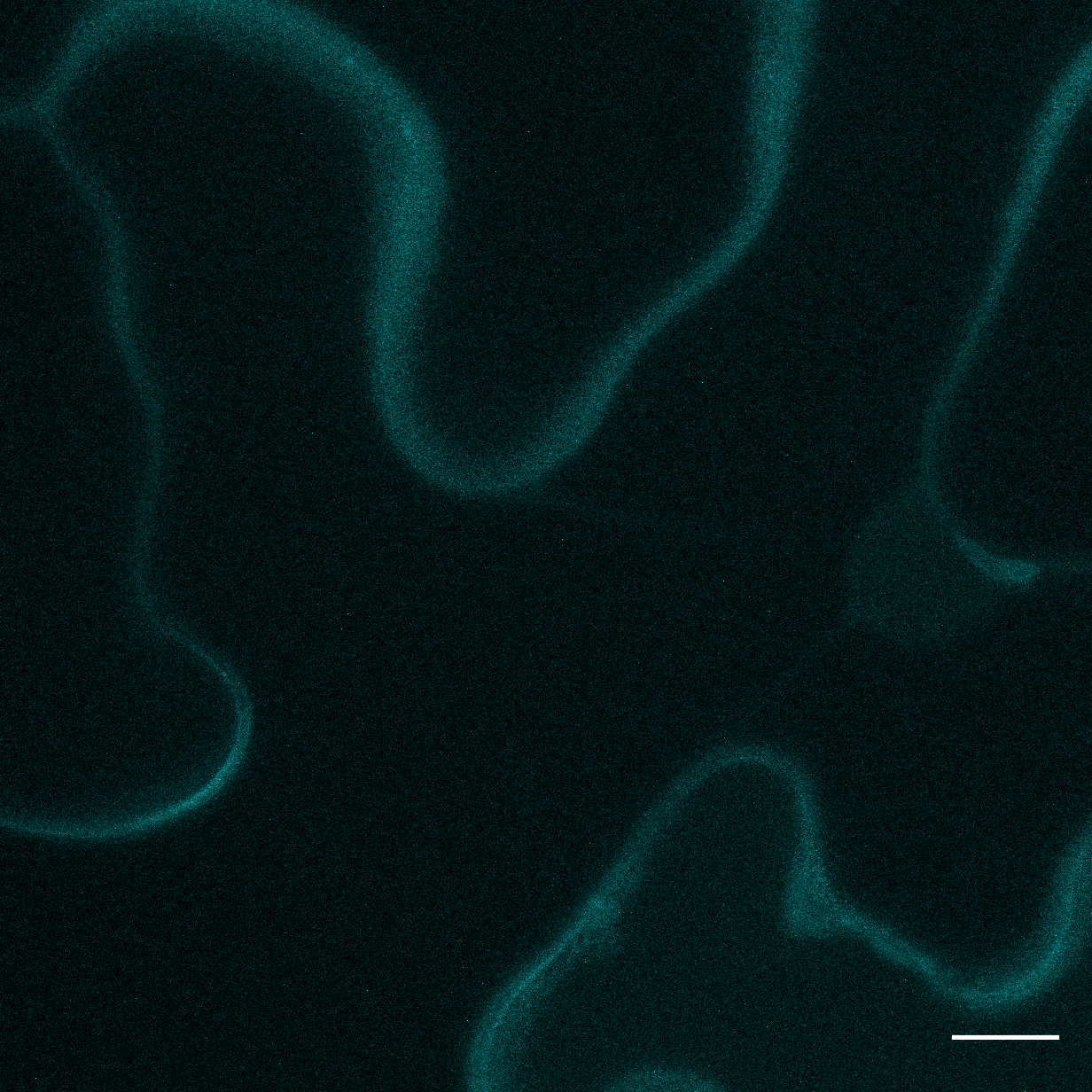

Supplement: Supplementary file 2 — Source data Fig. 2 [file 44319_2024_142_MOESM2_ESM.zip › Figure 2/2C/Confocal CFP cCFP-TSPO nVenus-ARF7.tif]

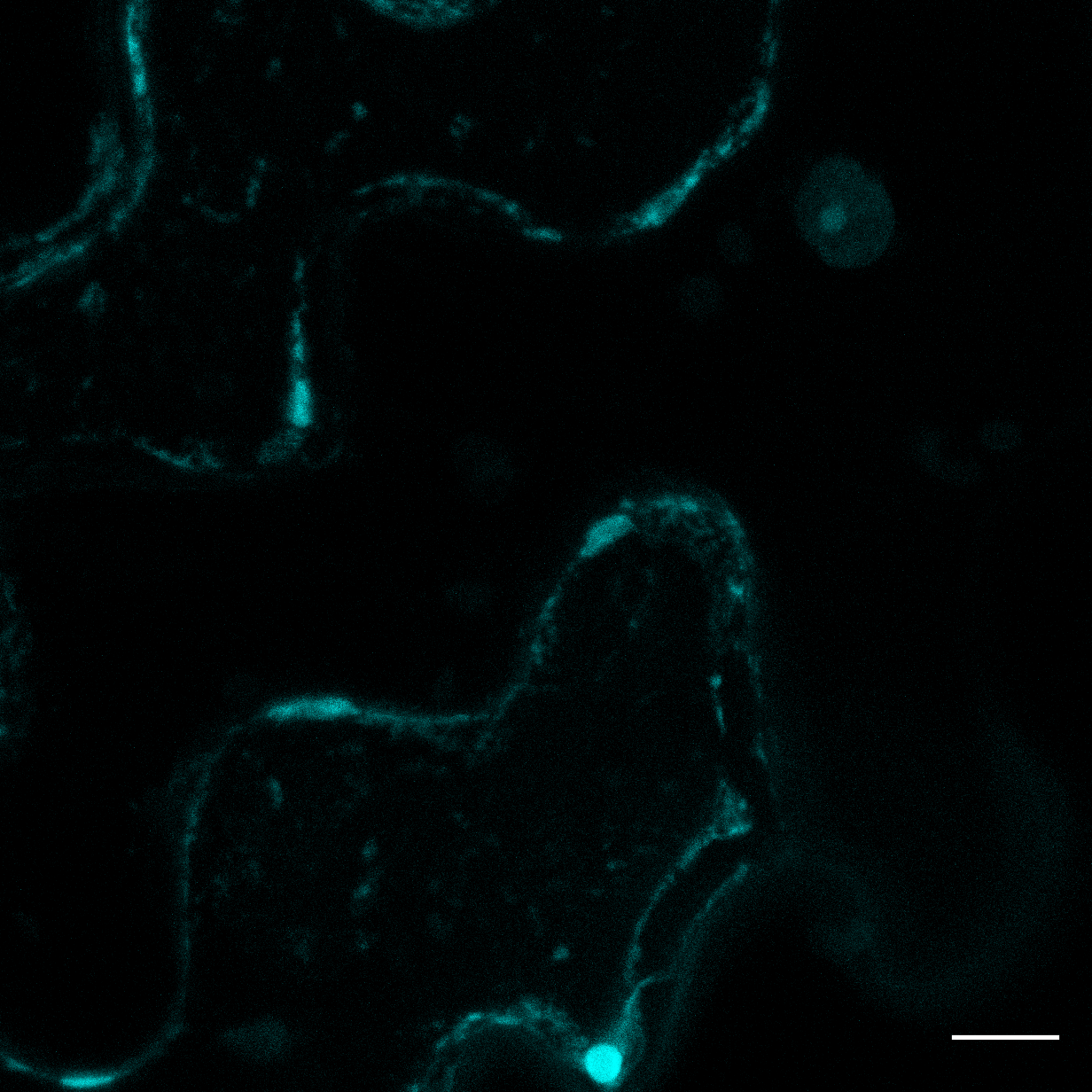

Supplement: Supplementary file 2 — Source data Fig. 2 [file 44319_2024_142_MOESM2_ESM.zip › Figure 2/2C/Confocal CFP nCFP-ATG8 cCFP-NBR1 nVenus-ARF7.tif]

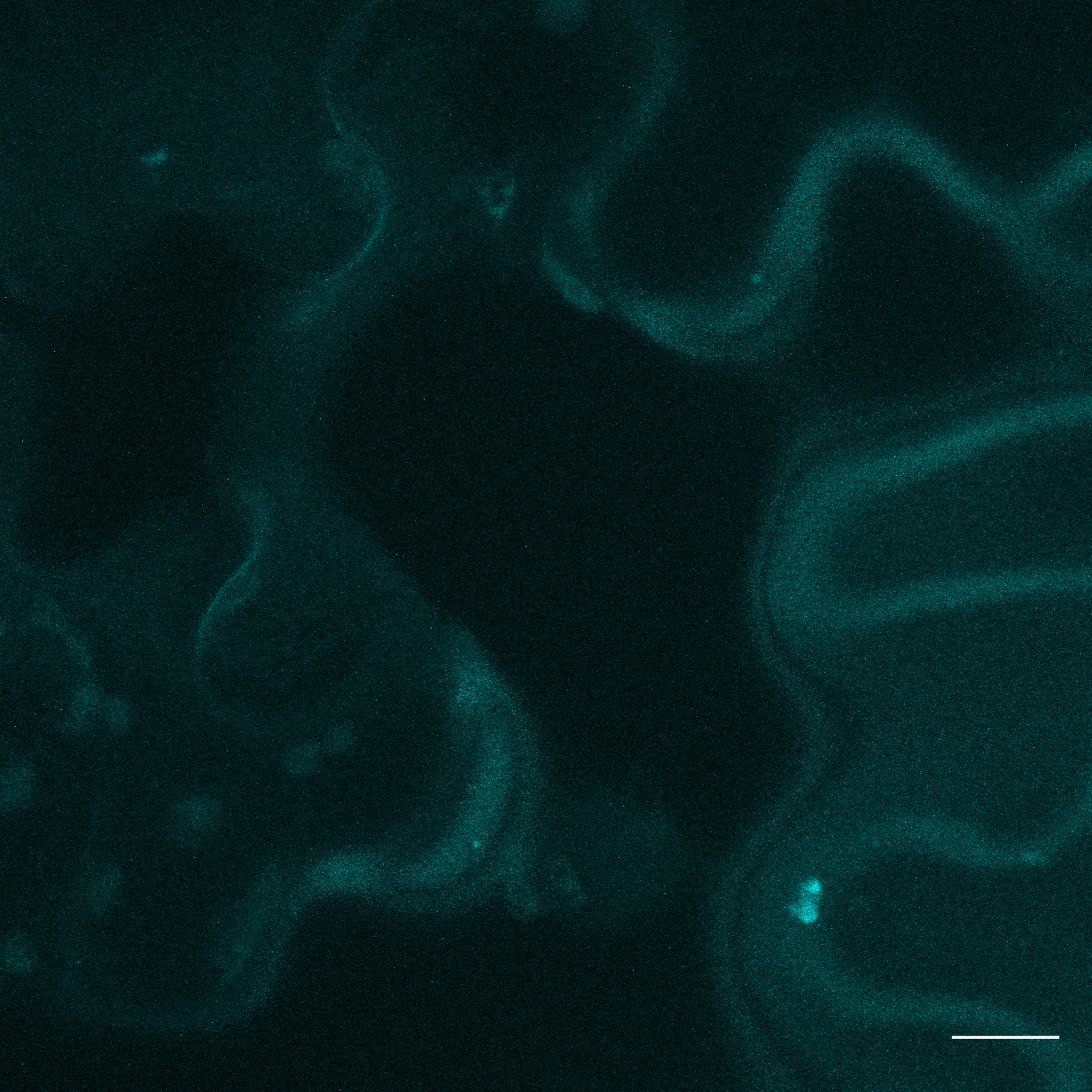

Supplement: Supplementary file 2 — Source data Fig. 2 [file 44319_2024_142_MOESM2_ESM.zip › Figure 2/2C/Confocal CFP nVenus-ARF7.tif]

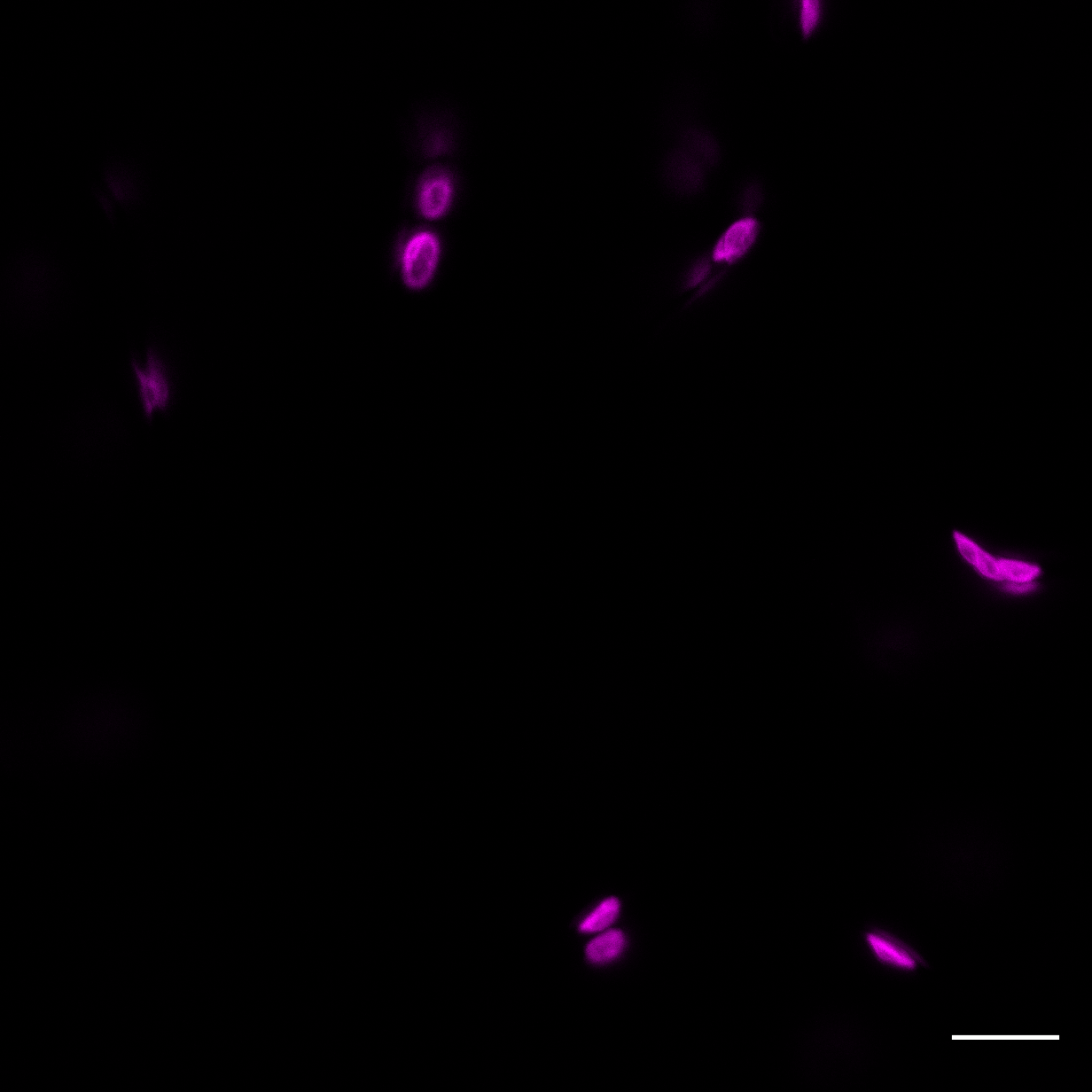

Supplement: Supplementary file 2 — Source data Fig. 2 [file 44319_2024_142_MOESM2_ESM.zip › Figure 2/2C/Confocal Chlorophyll cCFP-TSPO nVenus-ARF7.tif]

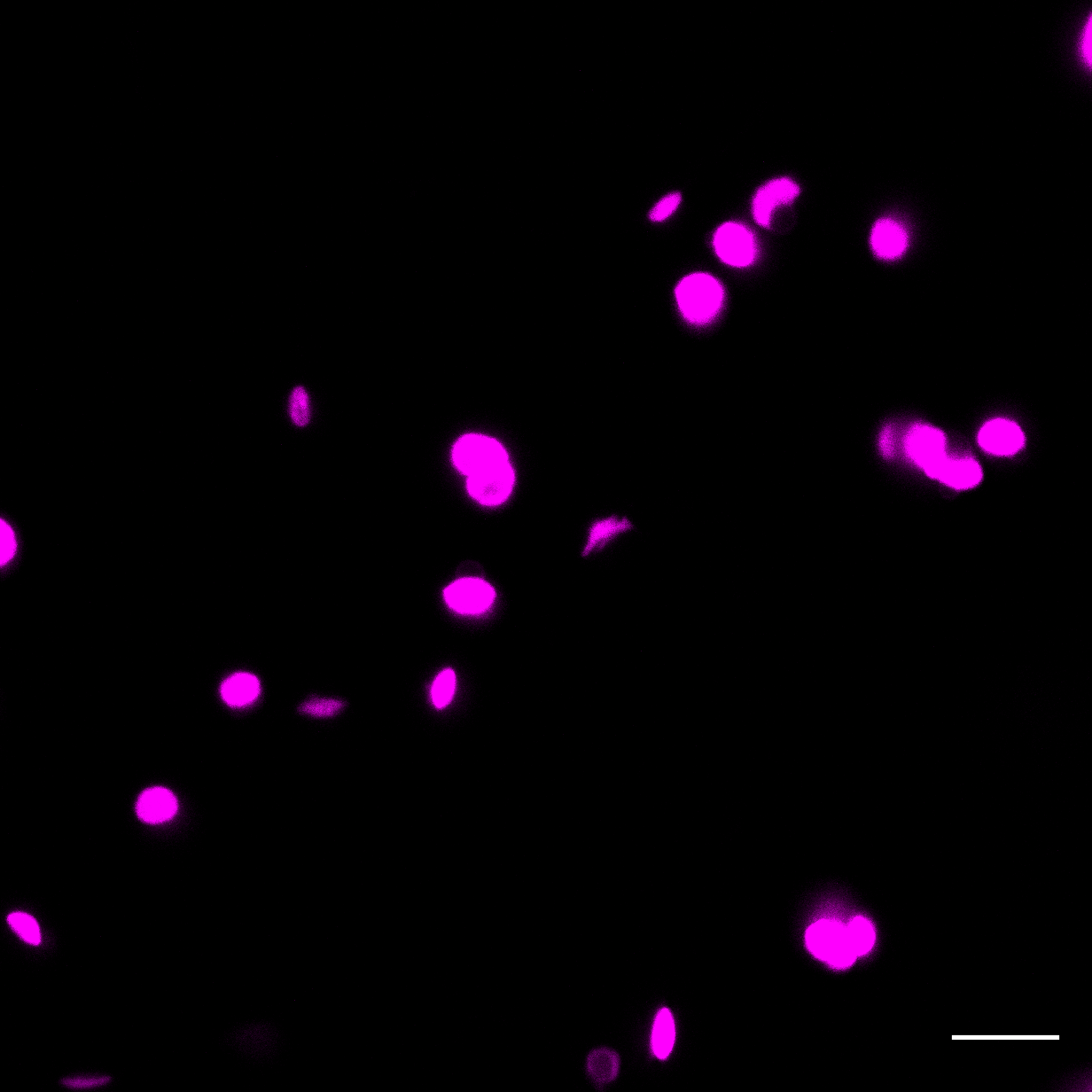

Supplement: Supplementary file 2 — Source data Fig. 2 [file 44319_2024_142_MOESM2_ESM.zip › Figure 2/2C/Confocal Chlorophyll nCFP-ATG8 cCFP-NBR1 nVenus-ARF7.tif]

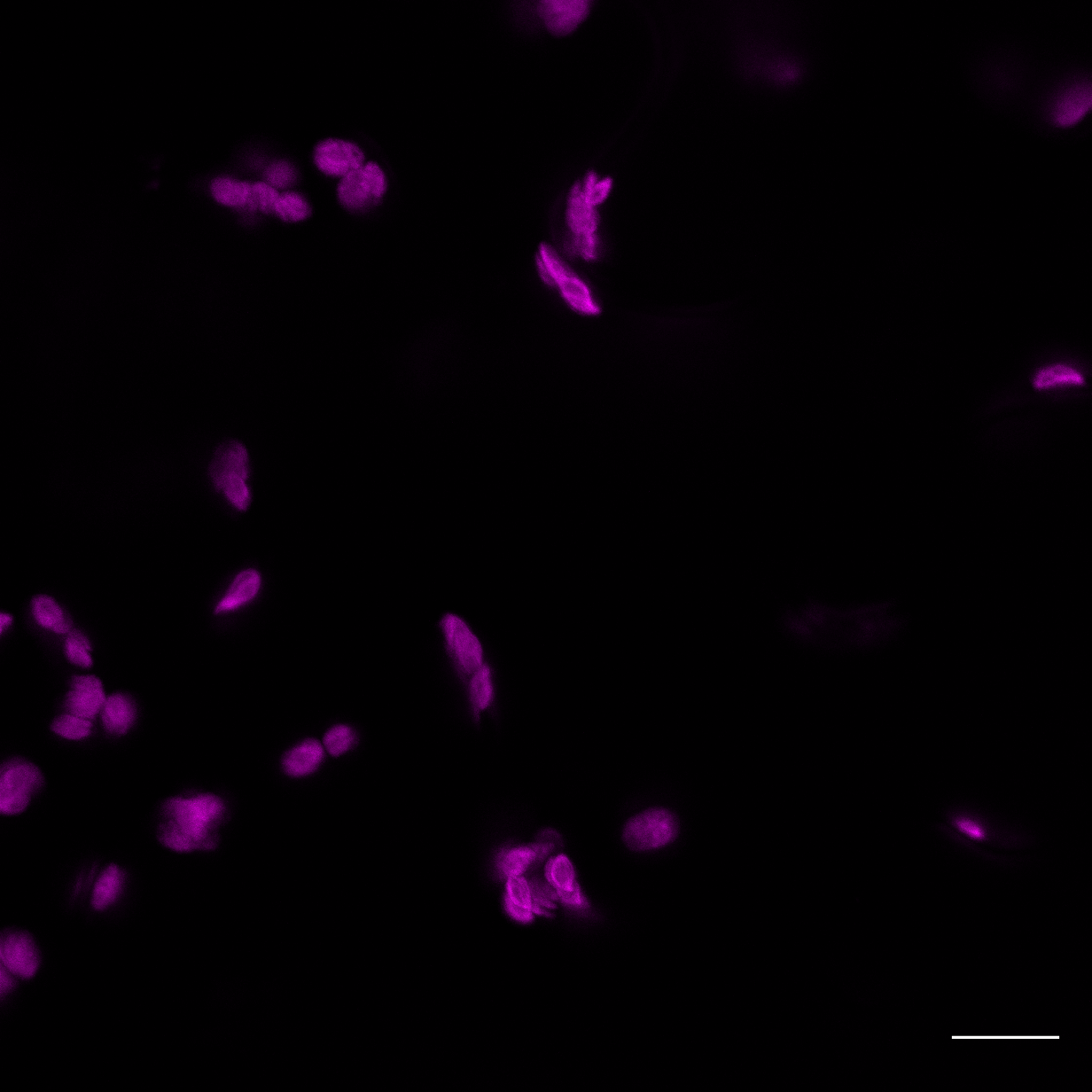

Supplement: Supplementary file 2 — Source data Fig. 2 [file 44319_2024_142_MOESM2_ESM.zip › Figure 2/2C/Confocal Chlorophyll nVenus-ARF7.tif]

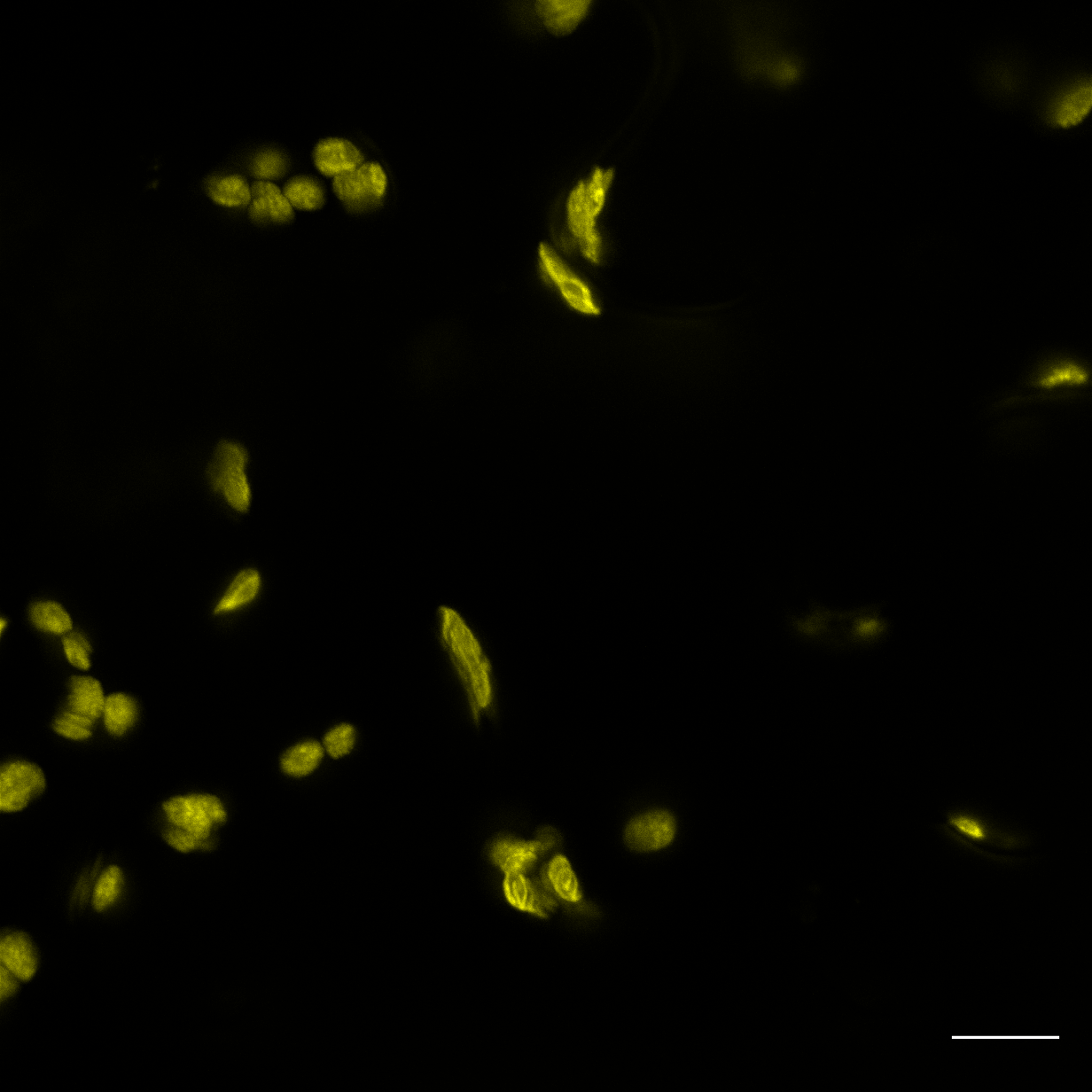

Supplement: Supplementary file 2 — Source data Fig. 2 [file 44319_2024_142_MOESM2_ESM.zip › Figure 2/2C/Confocal GFP nVenus-ARF7.tif]

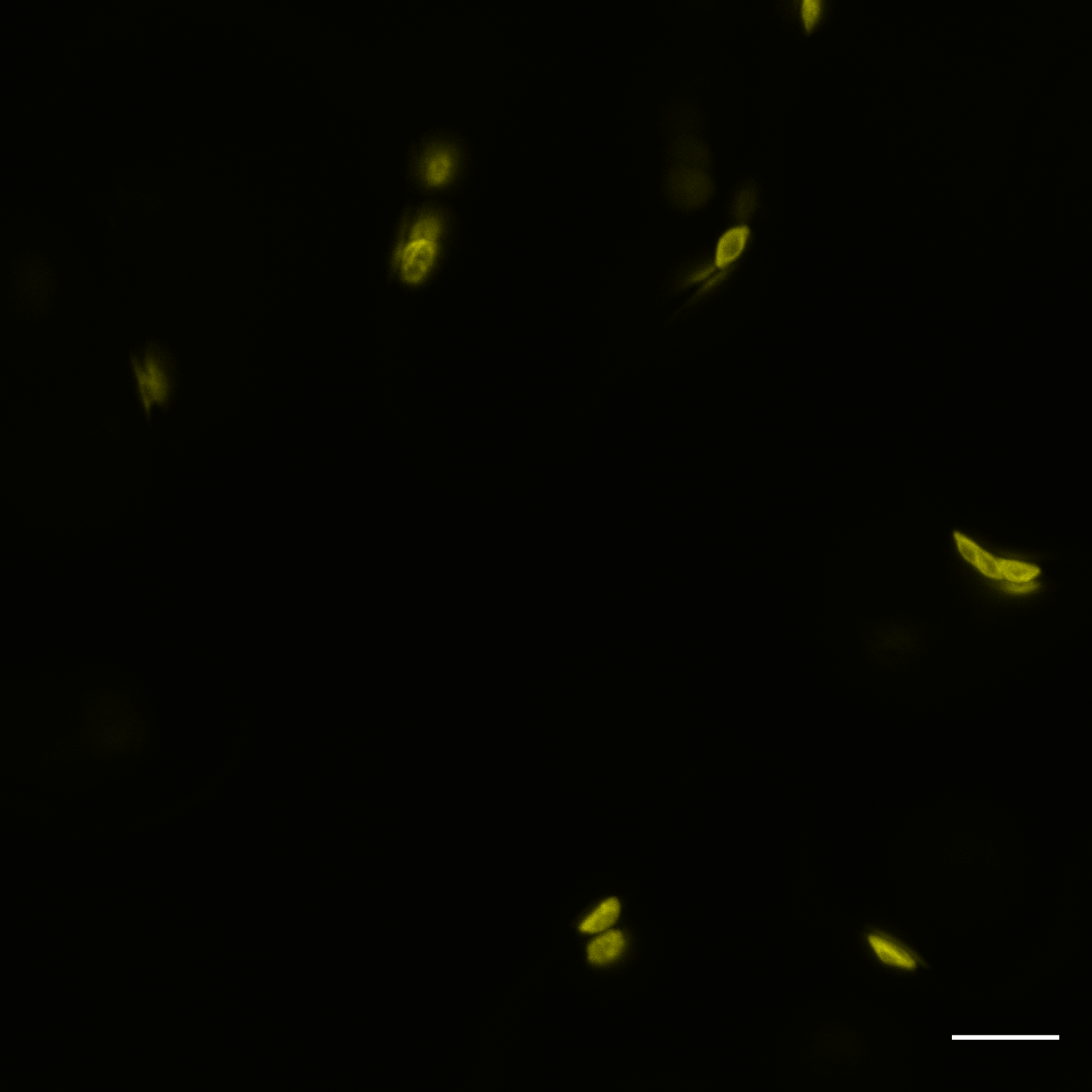

Supplement: Supplementary file 2 — Source data Fig. 2 [file 44319_2024_142_MOESM2_ESM.zip › Figure 2/2C/Confocal GFP cCFP-TSPO nVenus-ARF7.tif]

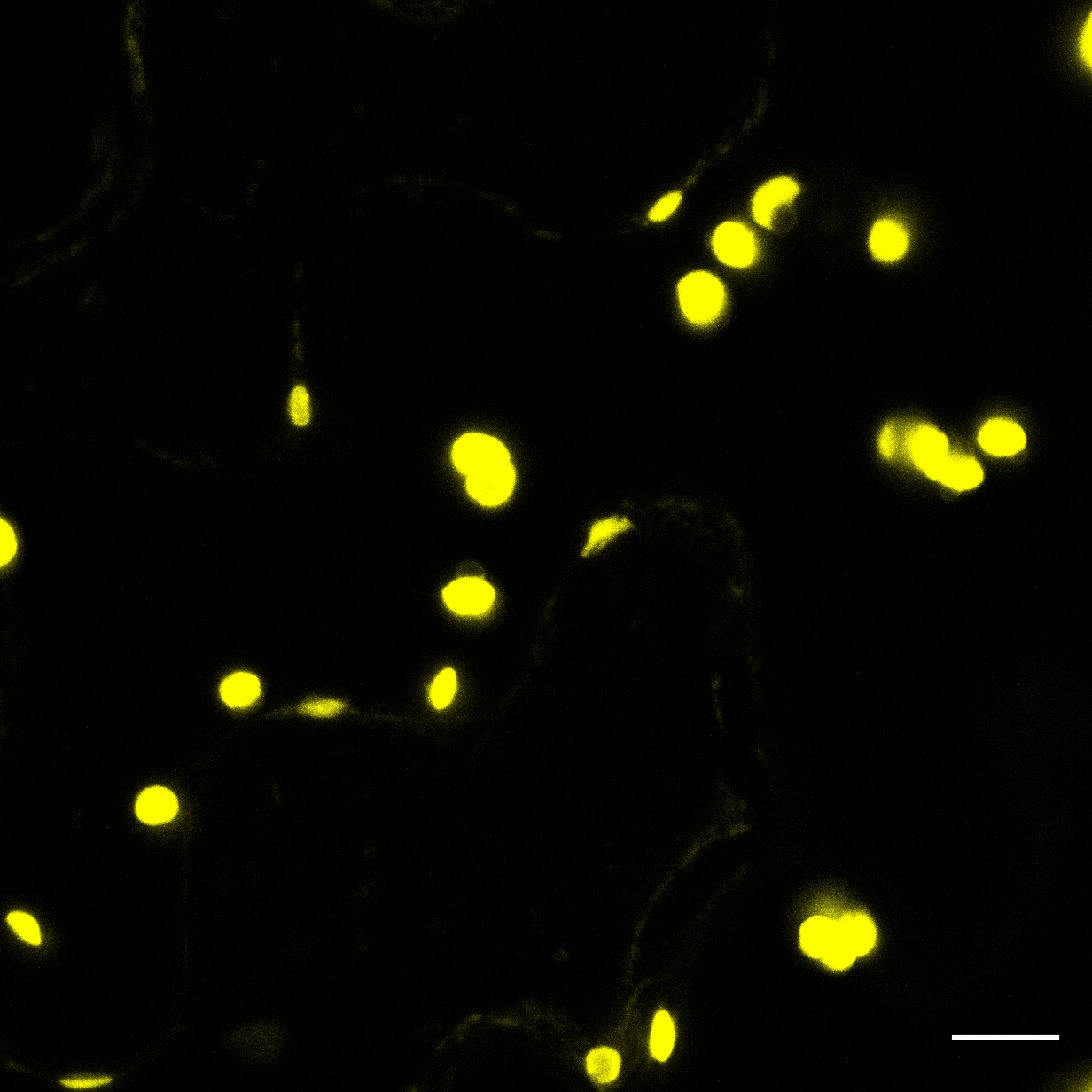

Supplement: Supplementary file 2 — Source data Fig. 2 [file 44319_2024_142_MOESM2_ESM.zip › Figure 2/2C/Confocal GFP nCFP-ATG8 cCFP-NBR1 nVenus-ARF7.tif]

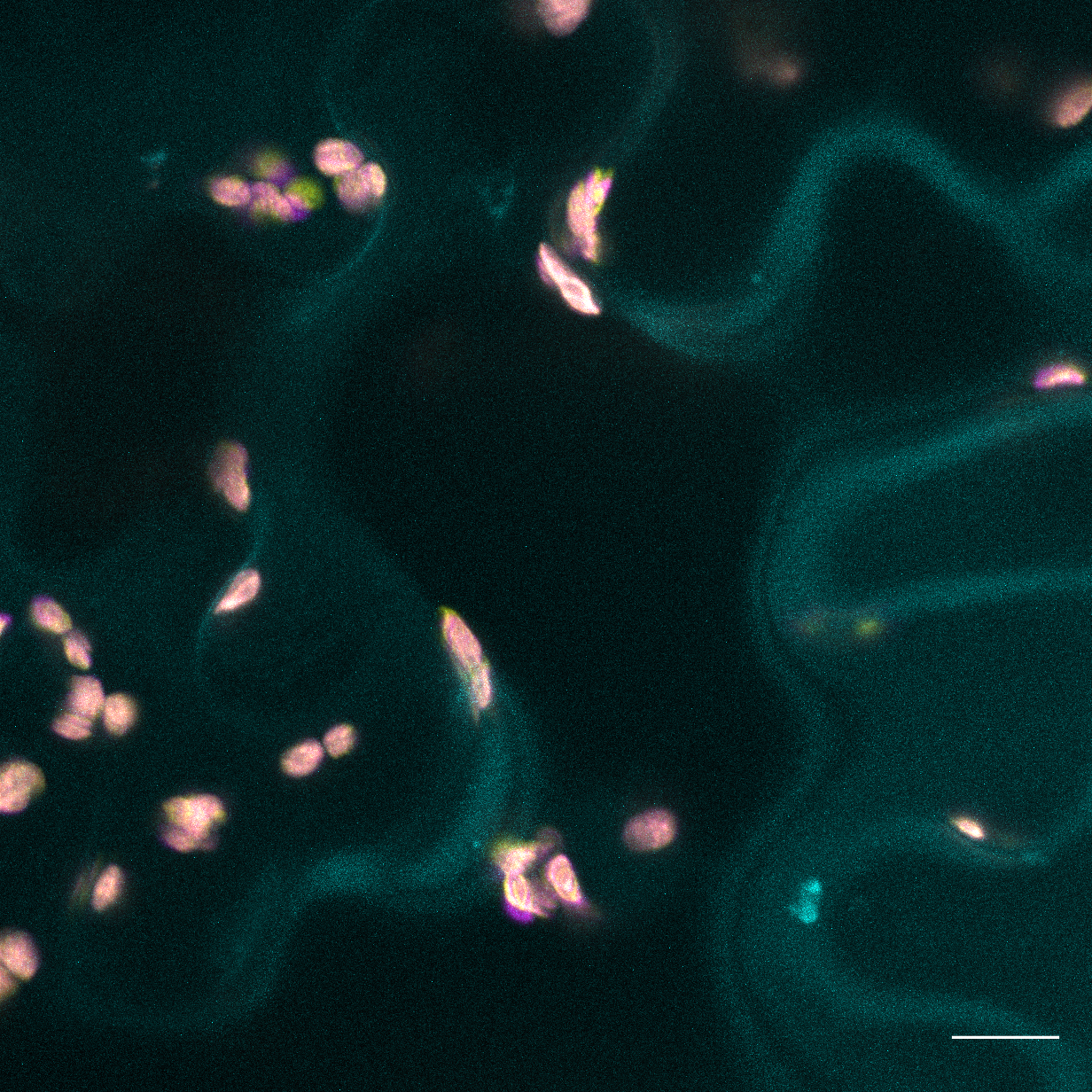

Supplement: Supplementary file 2 — Source data Fig. 2 [file 44319_2024_142_MOESM2_ESM.zip › Figure 2/2C/Confocal Merged nVenus-ARF7.tif]

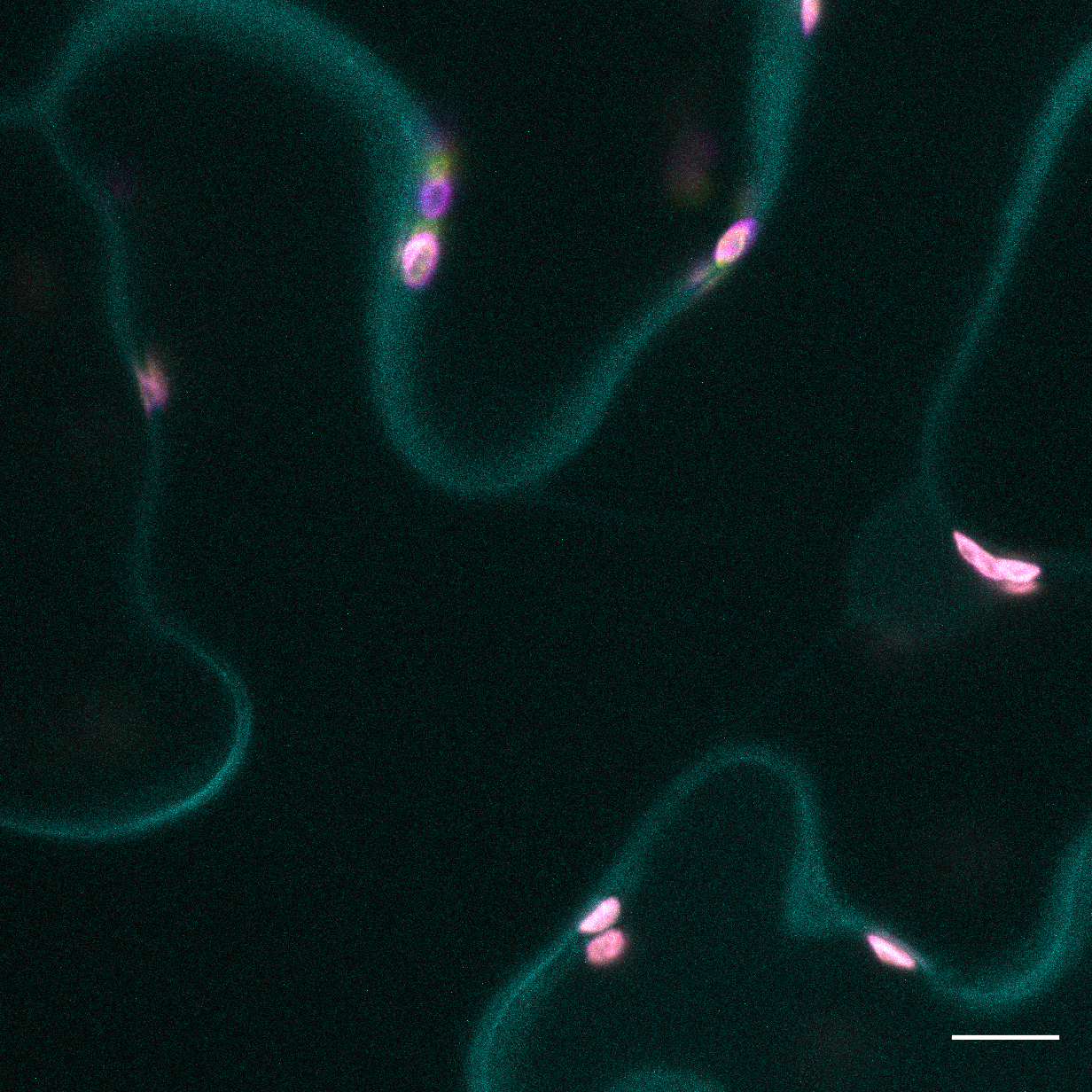

Supplement: Supplementary file 2 — Source data Fig. 2 [file 44319_2024_142_MOESM2_ESM.zip › Figure 2/2C/Confocal Merged cCFP-TSPO nVenus-ARF7.tif]

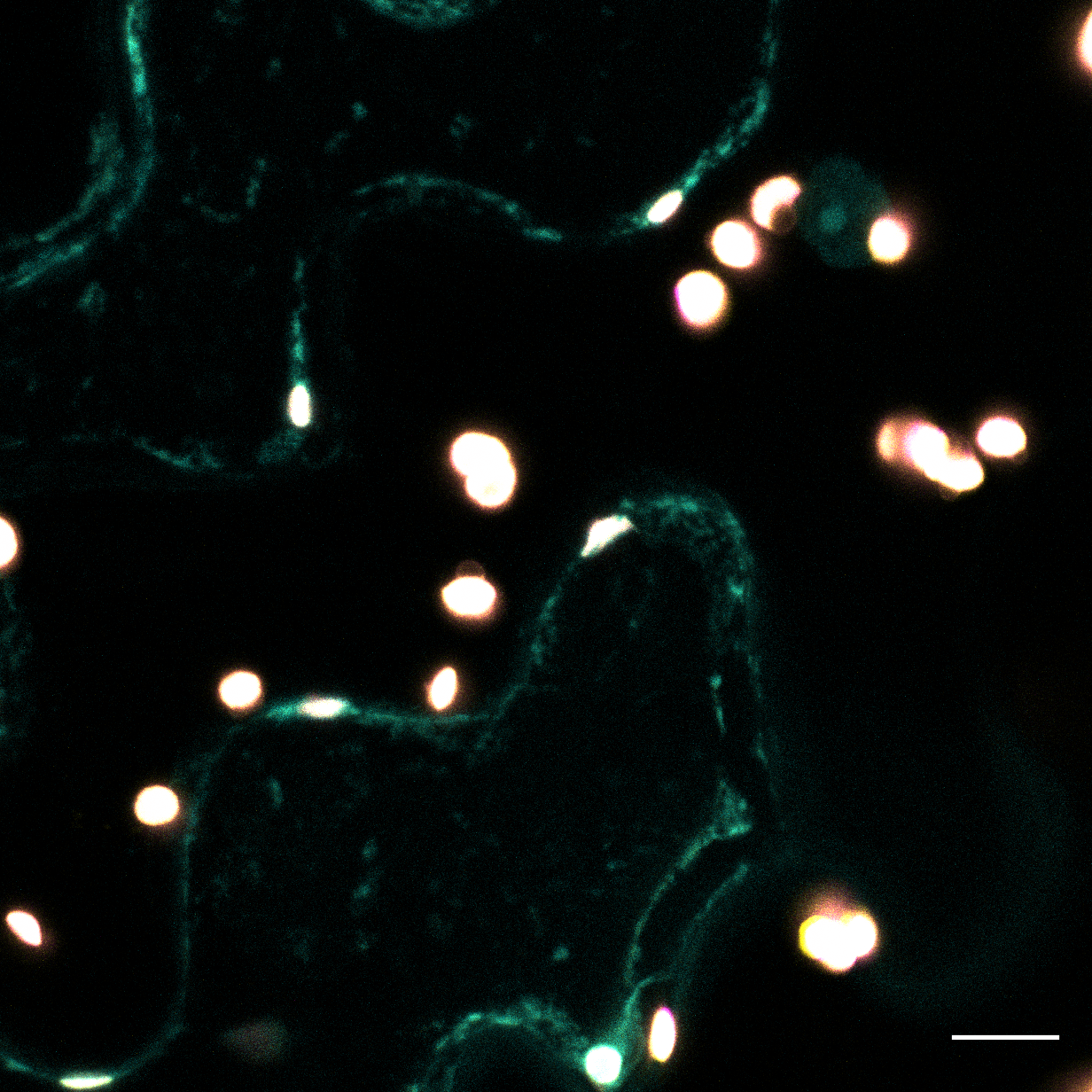

Supplement: Supplementary file 2 — Source data Fig. 2 [file 44319_2024_142_MOESM2_ESM.zip › Figure 2/2C/Confocal Merged nCFP-ATG8 cCFP-NBR1 nVenus-ARF7.tif]

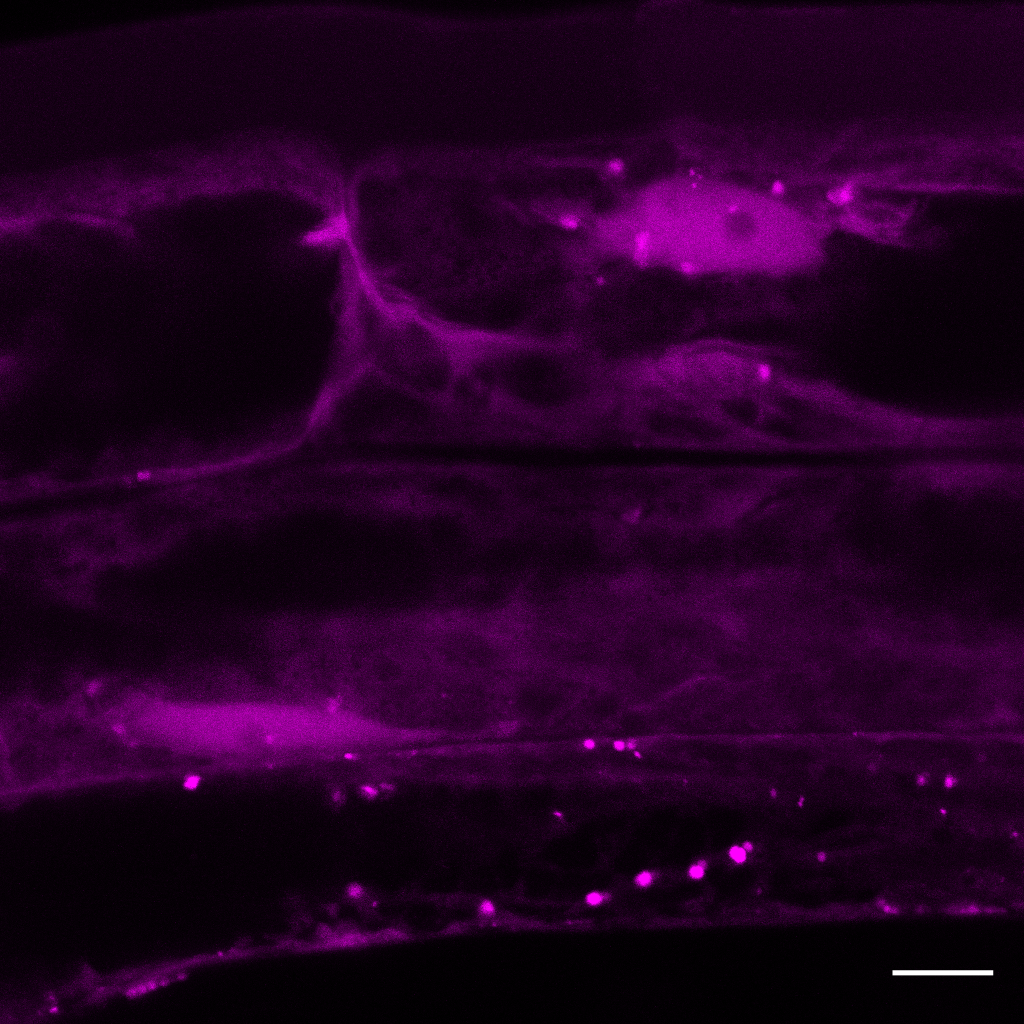

Supplement: Supplementary file 3 — Source data Fig. 3 [file 44319_2024_142_MOESM3_ESM.zip › Figure 3/3A/mCherry-ATG8 ARF7-Venus Mature Zone mCherry.tif]

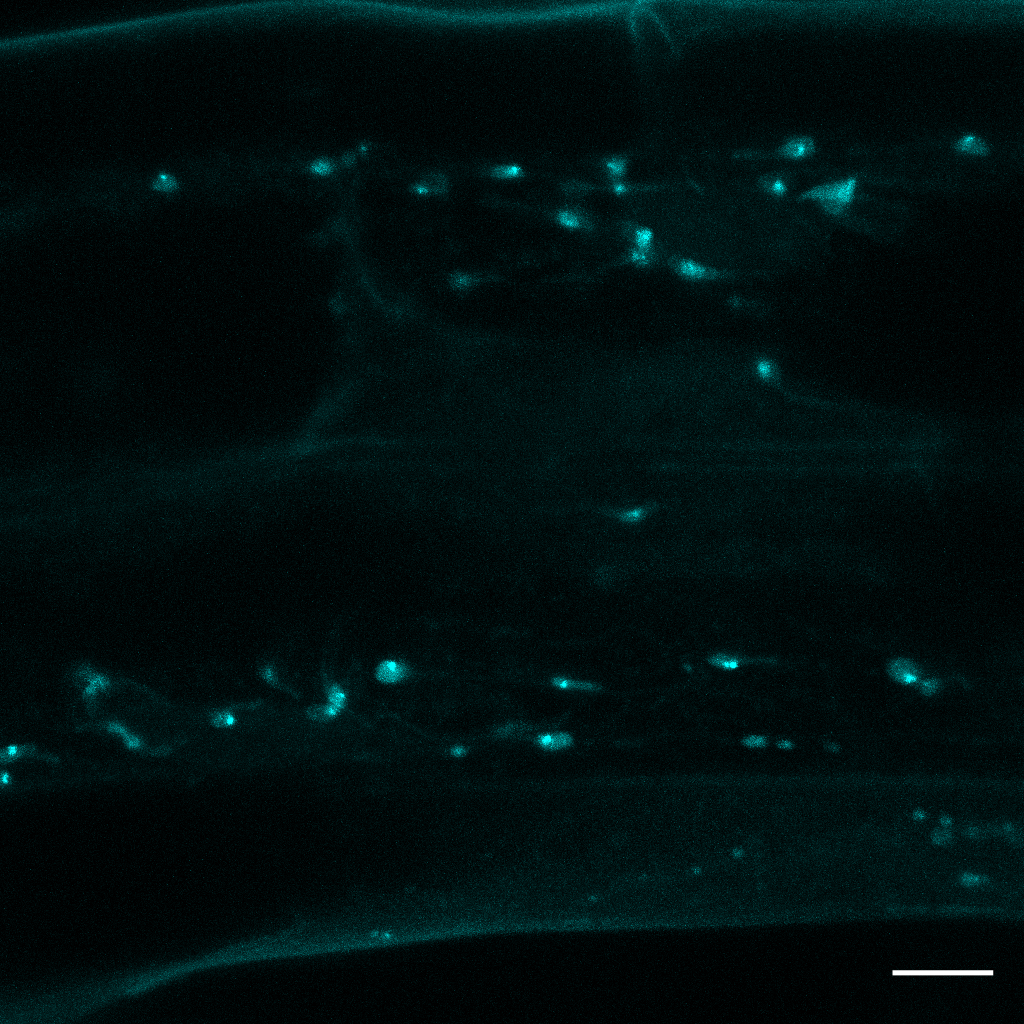

Supplement: Supplementary file 3 — Source data Fig. 3 [file 44319_2024_142_MOESM3_ESM.zip › Figure 3/3A/mCherry-ATG8 ARF7-Venus Mature Zone Venus.tif]

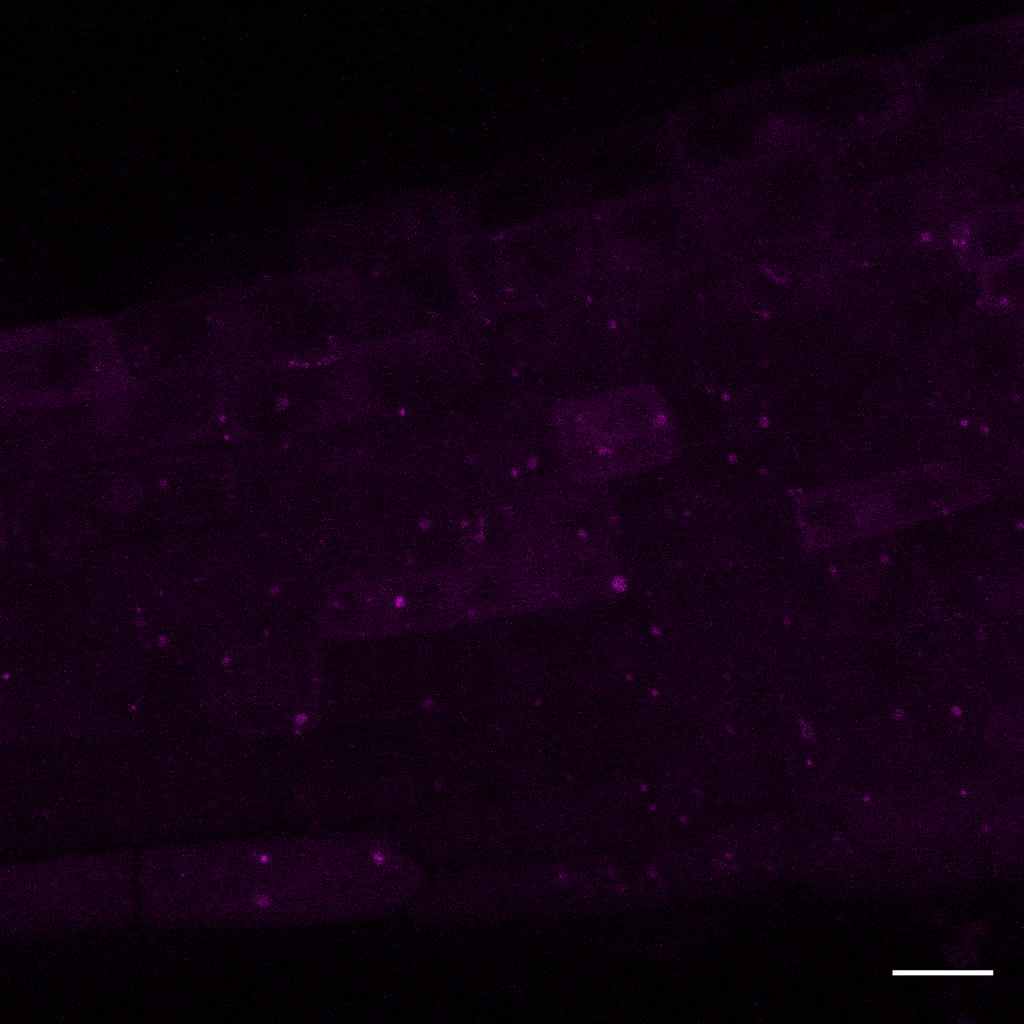

Supplement: Supplementary file 3 — Source data Fig. 3 [file 44319_2024_142_MOESM3_ESM.zip › Figure 3/3A/mCherry-ATG8 ARF7-Venus Meristem mCherry.tif]

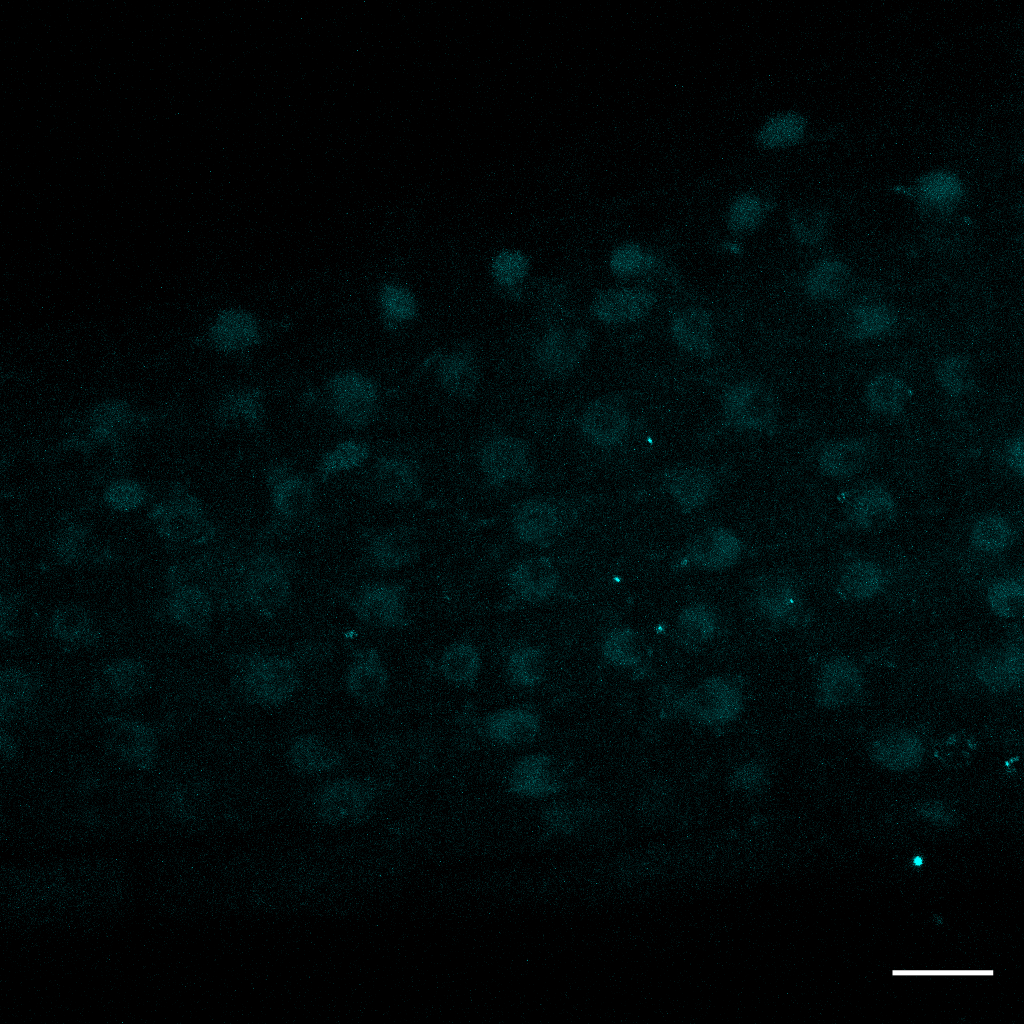

Supplement: Supplementary file 3 — Source data Fig. 3 [file 44319_2024_142_MOESM3_ESM.zip › Figure 3/3A/mCherry-ATG8 ARF7-Venus Meristem Venus.tif]

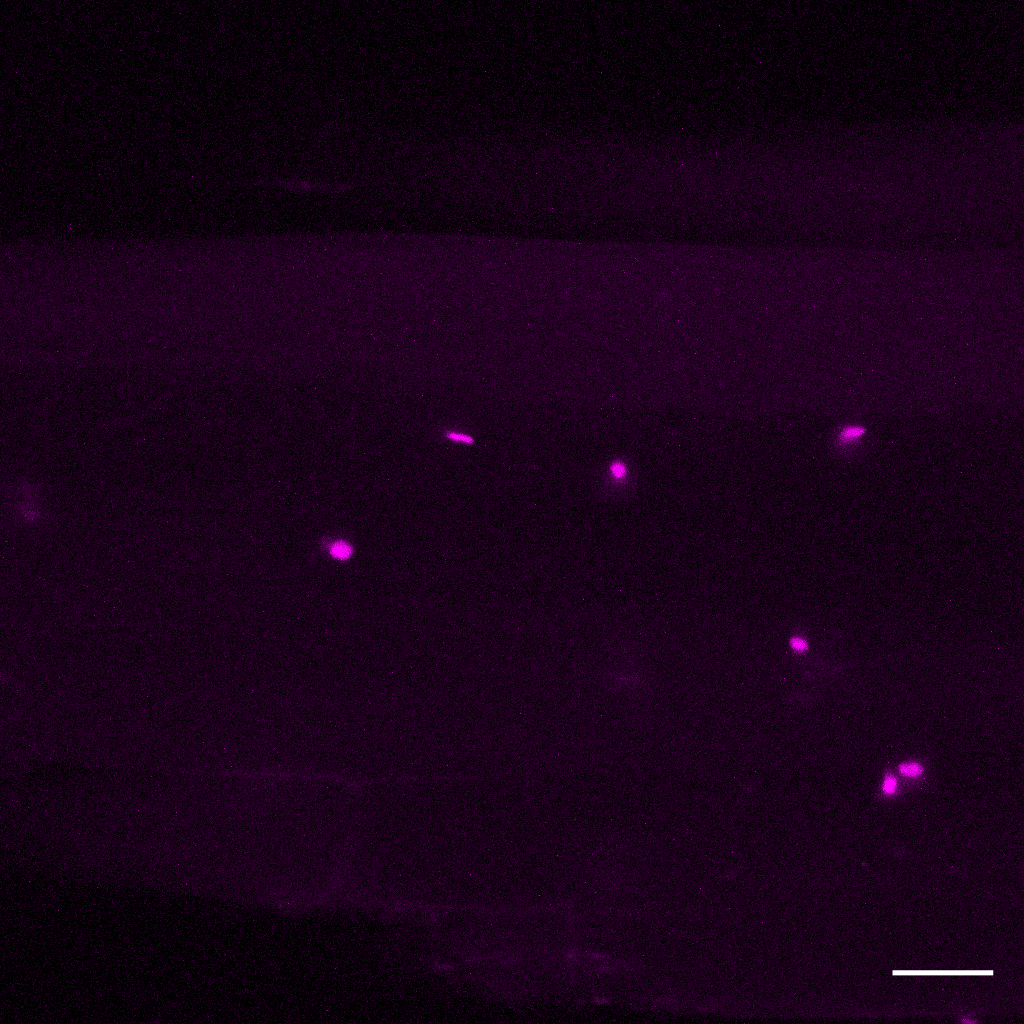

Supplement: Supplementary file 3 — Source data Fig. 3 [file 44319_2024_142_MOESM3_ESM.zip › Figure 3/3A/mCherry-NBR1 ARF7-Venus Mature Zone mCherry.tif]

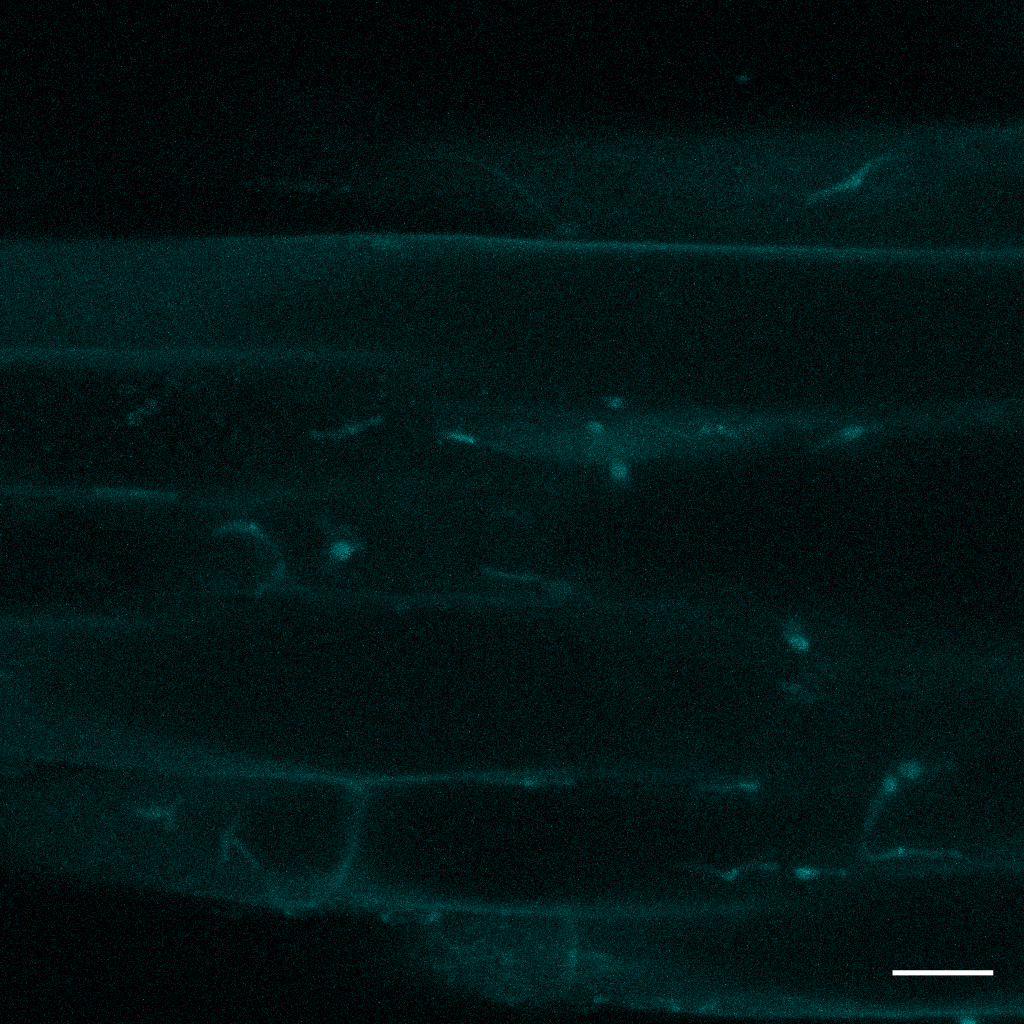

Supplement: Supplementary file 3 — Source data Fig. 3 [file 44319_2024_142_MOESM3_ESM.zip › Figure 3/3A/mCherry-NBR1 ARF7-Venus Mature Zone Venus.tif]

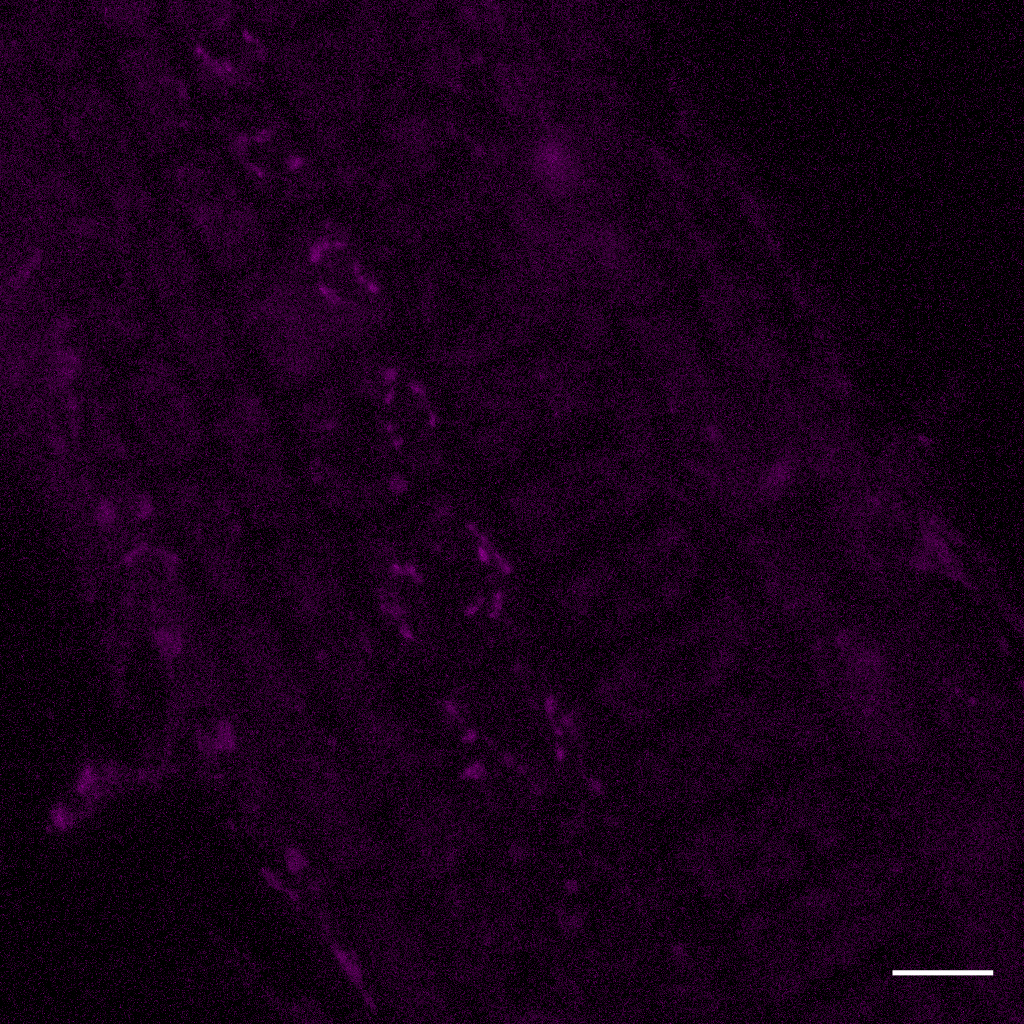

Supplement: Supplementary file 3 — Source data Fig. 3 [file 44319_2024_142_MOESM3_ESM.zip › Figure 3/3A/mCherry-NBR1 ARF7-Venus Meristem mCherry.tif]

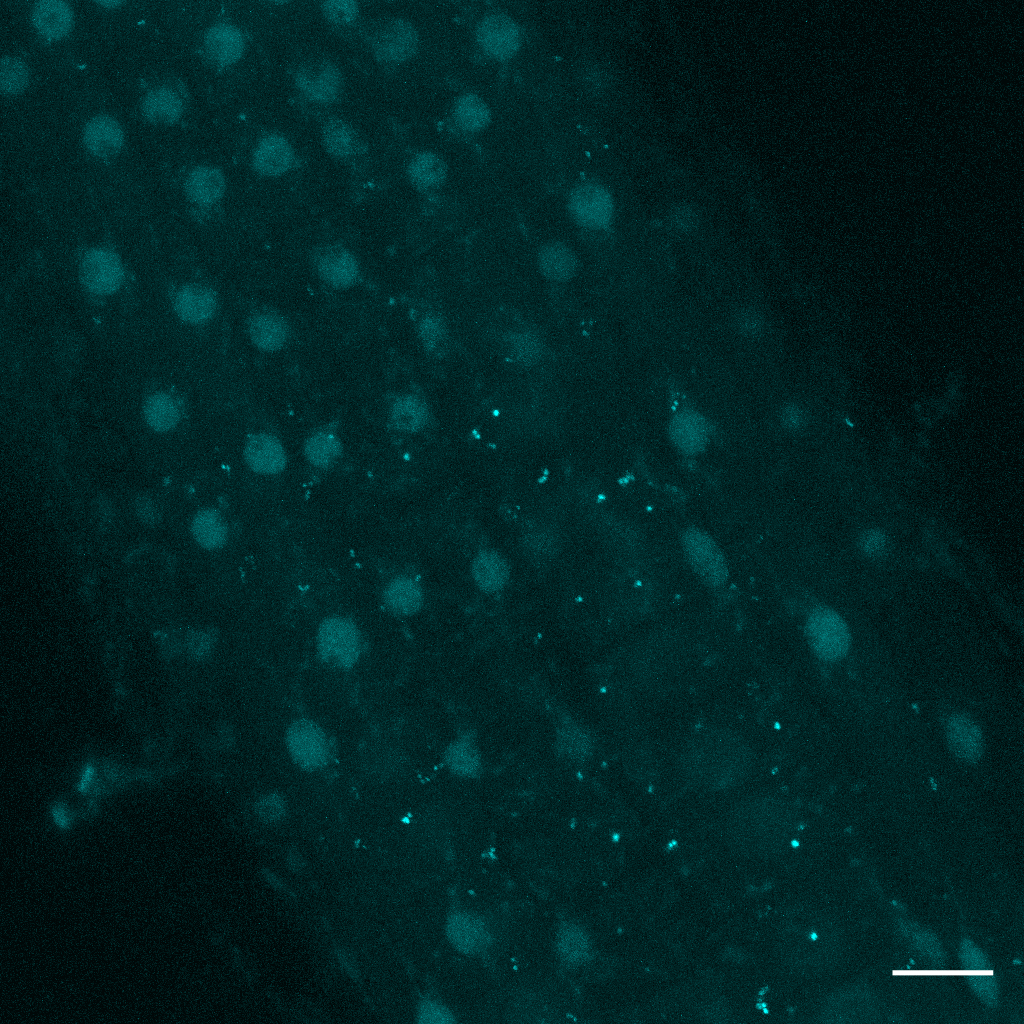

Supplement: Supplementary file 3 — Source data Fig. 3 [file 44319_2024_142_MOESM3_ESM.zip › Figure 3/3A/mCherry-NBR1 ARF7-Venus Meristem Venus.tif]

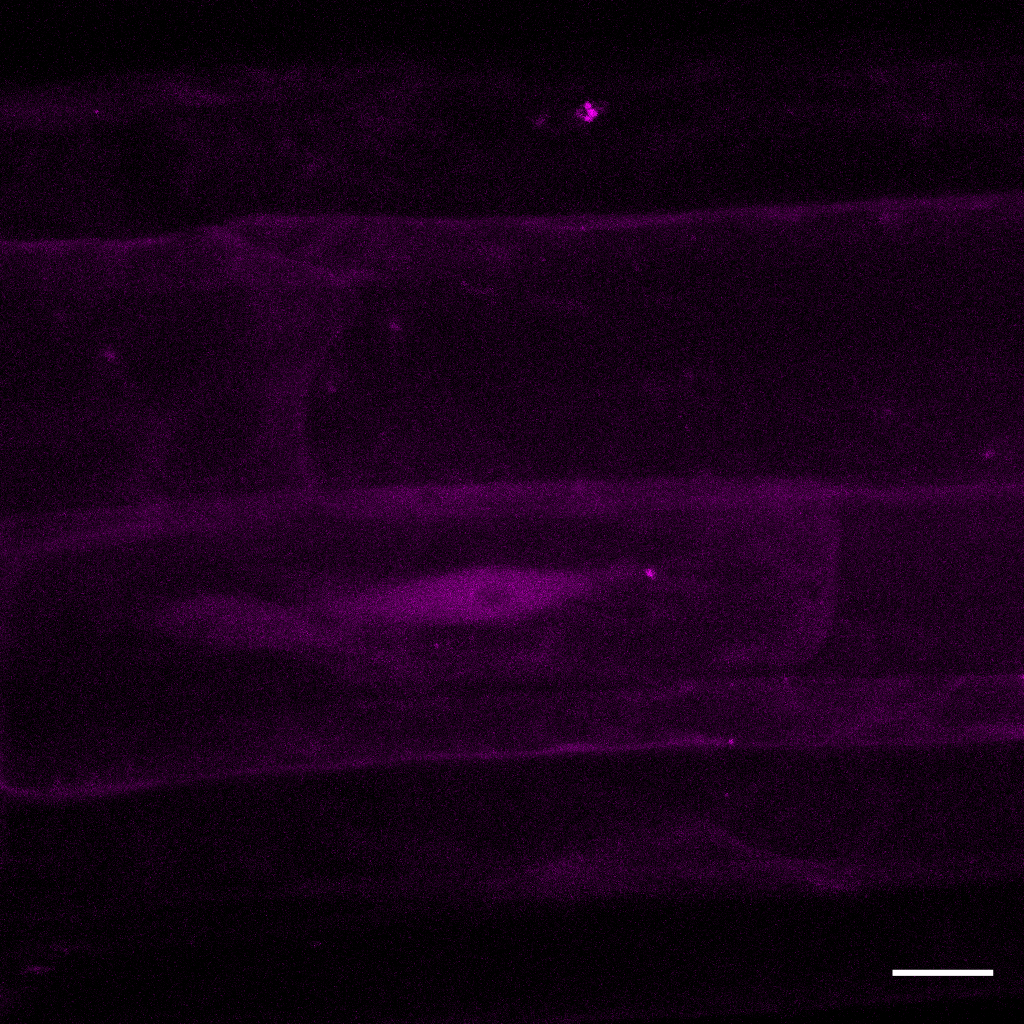

Supplement: Supplementary file 3 — Source data Fig. 3 [file 44319_2024_142_MOESM3_ESM.zip › Figure 3/3C/mCherry-ATG8 ARF7-Venus Mature Zone 0 hours mCherry.tif]

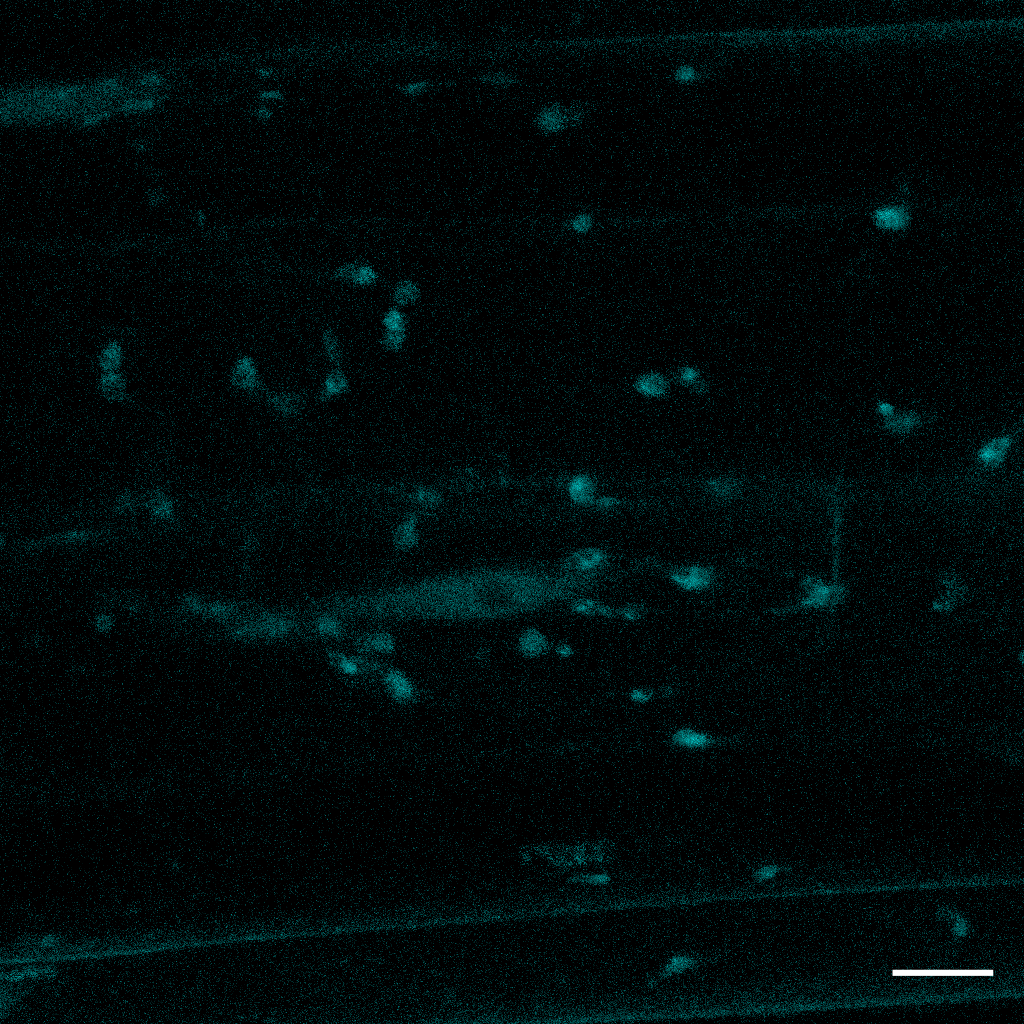

Supplement: Supplementary file 3 — Source data Fig. 3 [file 44319_2024_142_MOESM3_ESM.zip › Figure 3/3C/mCherry-ATG8 ARF7-Venus Mature Zone 0 hours Venus.tif]

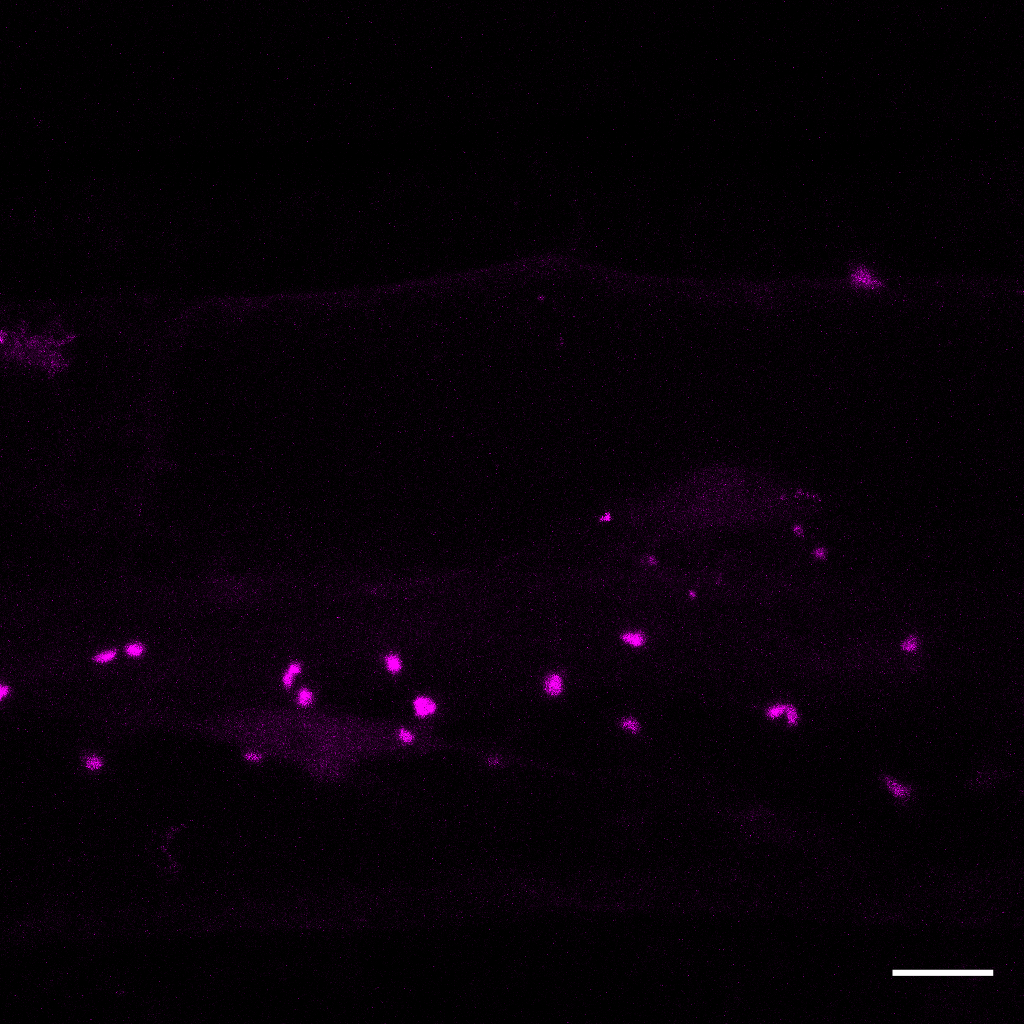

Supplement: Supplementary file 3 — Source data Fig. 3 [file 44319_2024_142_MOESM3_ESM.zip › Figure 3/3C/mCherry-ATG8 ARF7-Venus Mature Zone 6 hours mCherry.tif]

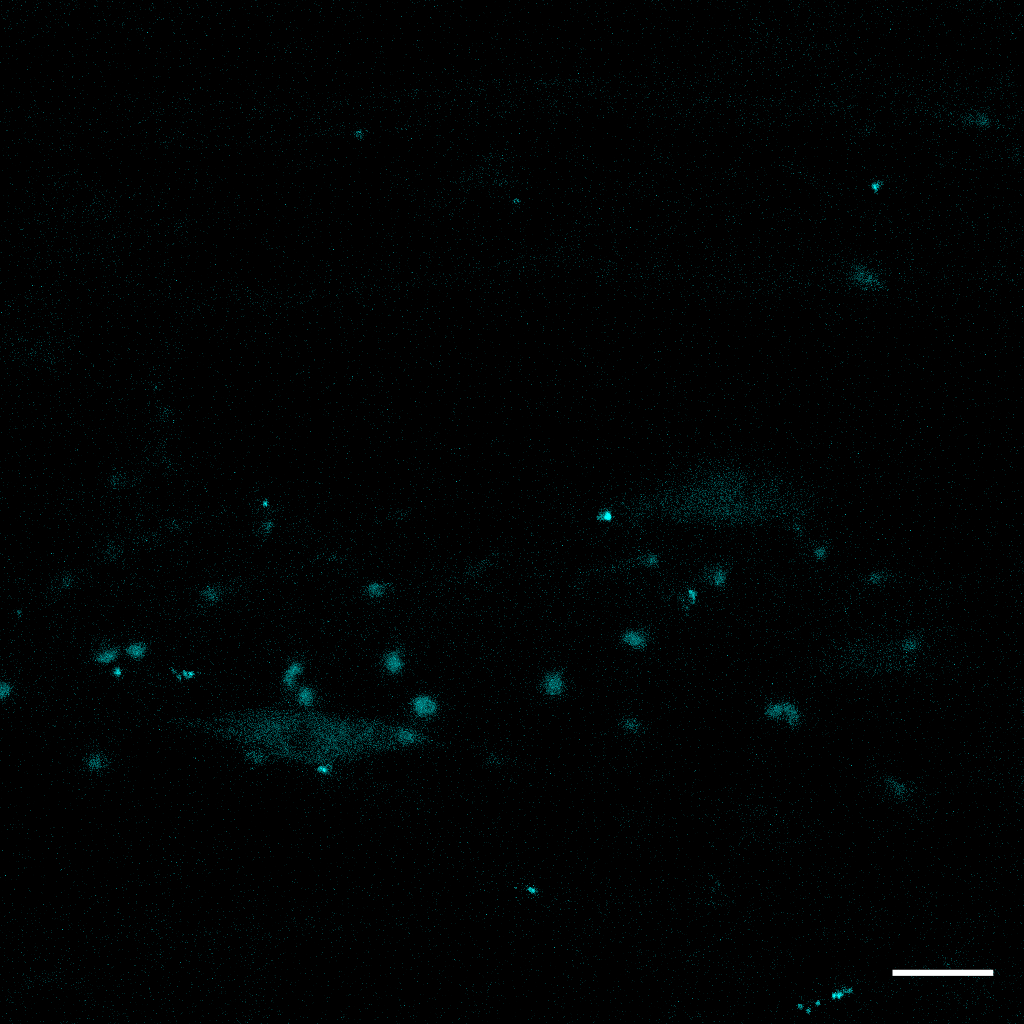

Supplement: Supplementary file 3 — Source data Fig. 3 [file 44319_2024_142_MOESM3_ESM.zip › Figure 3/3C/mCherry-ATG8 ARF7-Venus Mature Zone 6 hours Venus.tif]

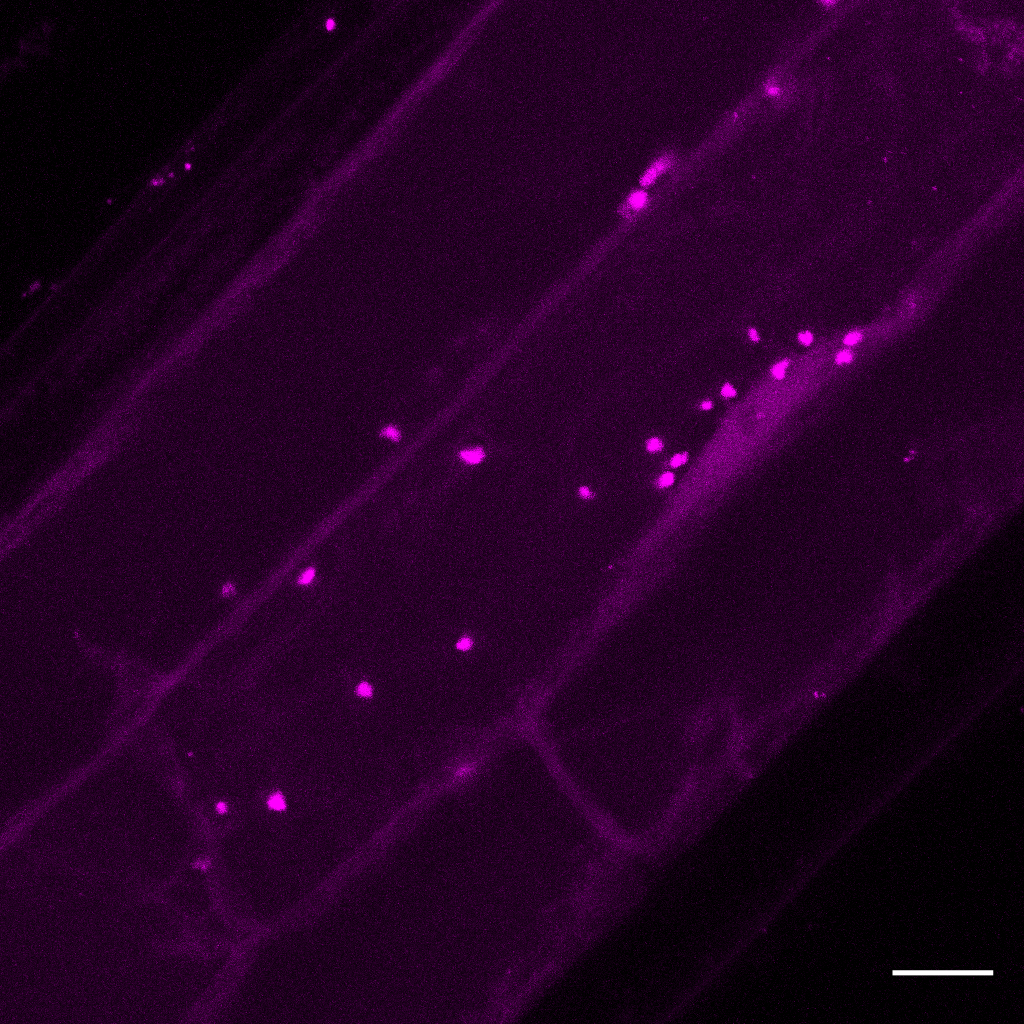

Supplement: Supplementary file 3 — Source data Fig. 3 [file 44319_2024_142_MOESM3_ESM.zip › Figure 3/3C/mCherry-ATG8 ARF7-Venus Mature Zone E64d pepstatin A mCherry.tif]

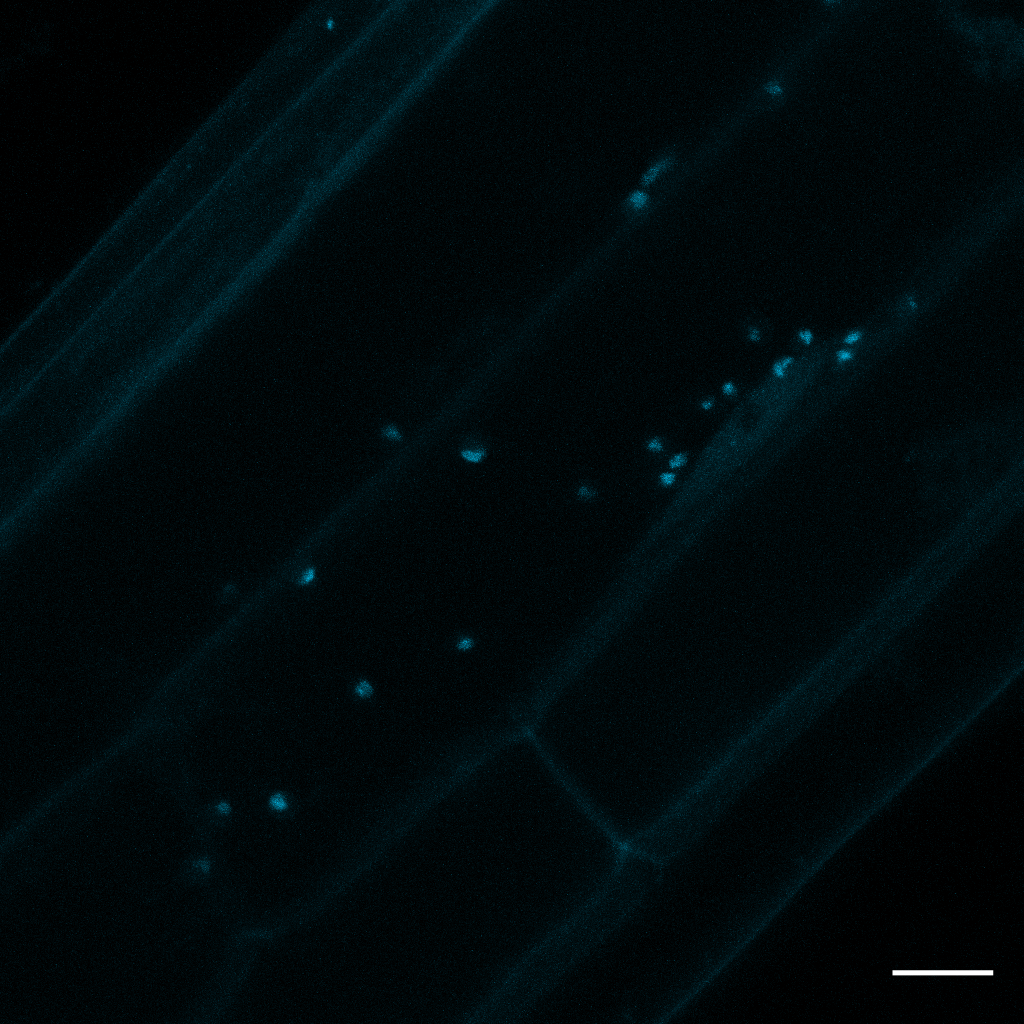

Supplement: Supplementary file 3 — Source data Fig. 3 [file 44319_2024_142_MOESM3_ESM.zip › Figure 3/3C/mCherry-ATG8 ARF7-Venus Mature Zone E64d pepstatin A venus.tif]

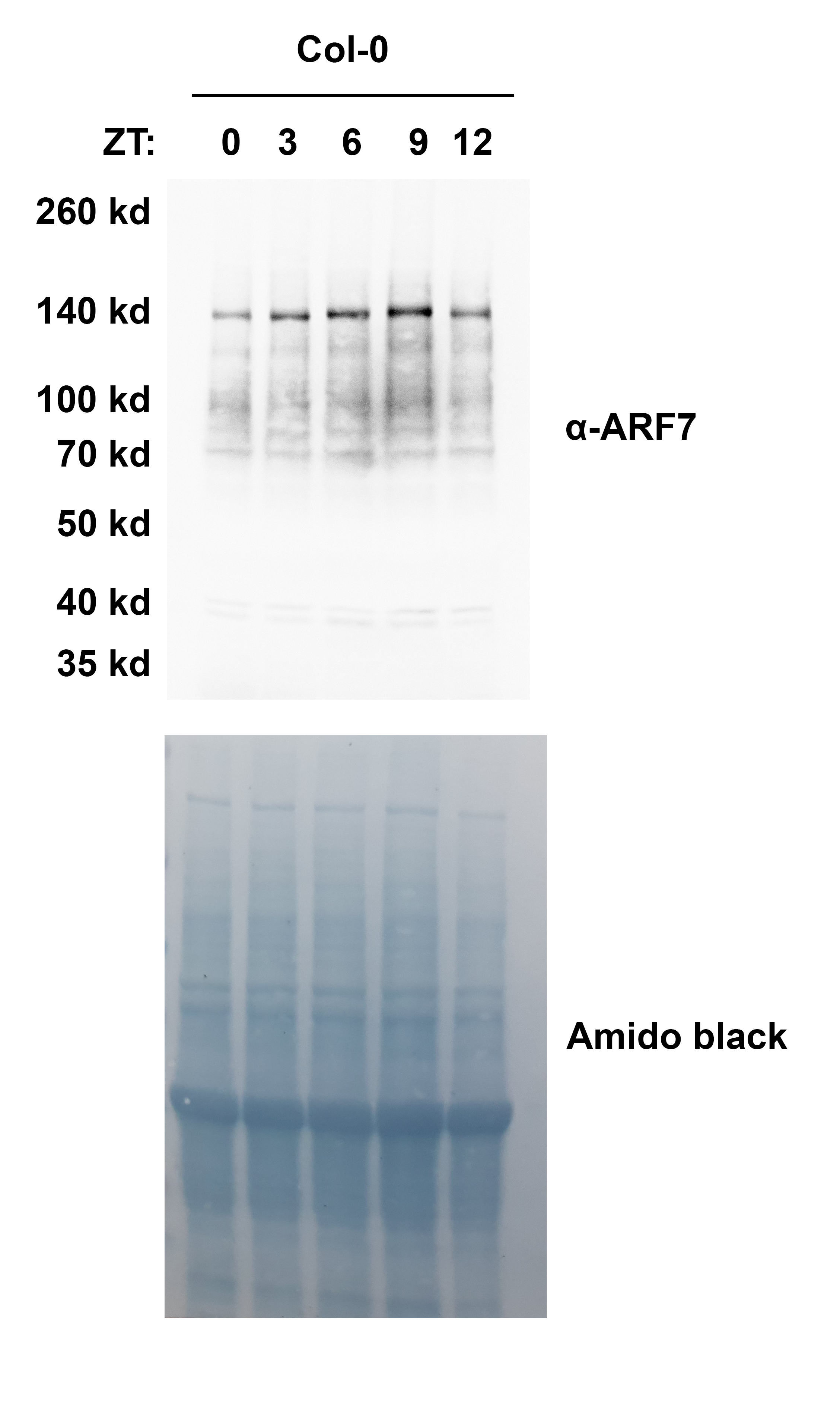

Supplement: Supplementary file 4 — Source data Fig. 4 [file 44319_2024_142_MOESM4_ESM.zip › Figure 4/4A/replicate/Western blot oscillation ARF7 in Col-0 replicate 1.tif]

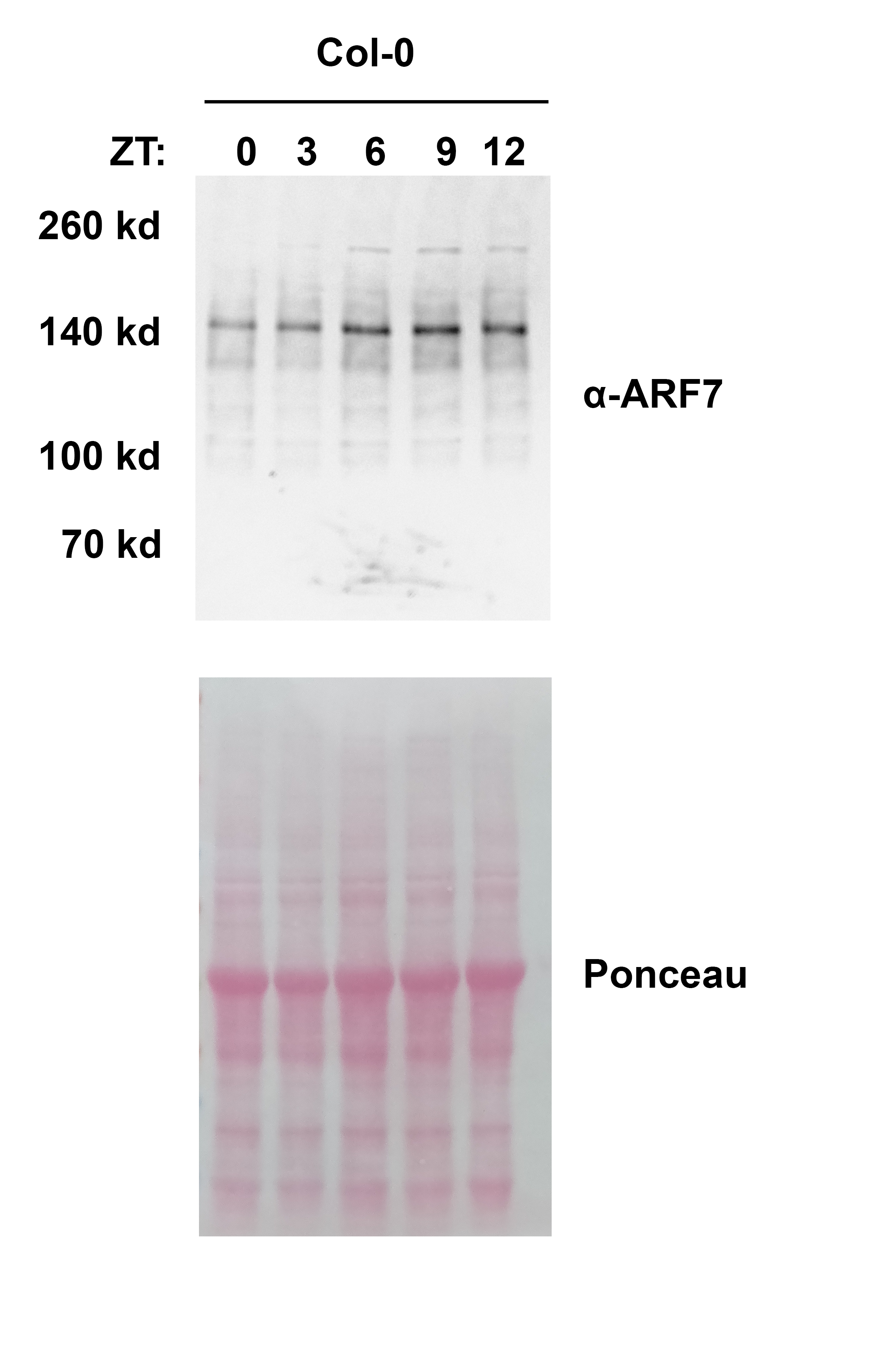

Supplement: Supplementary file 4 — Source data Fig. 4 [file 44319_2024_142_MOESM4_ESM.zip › Figure 4/4A/replicate/Western blot oscillation ARF7 in Col-0 replicate 2.tif]

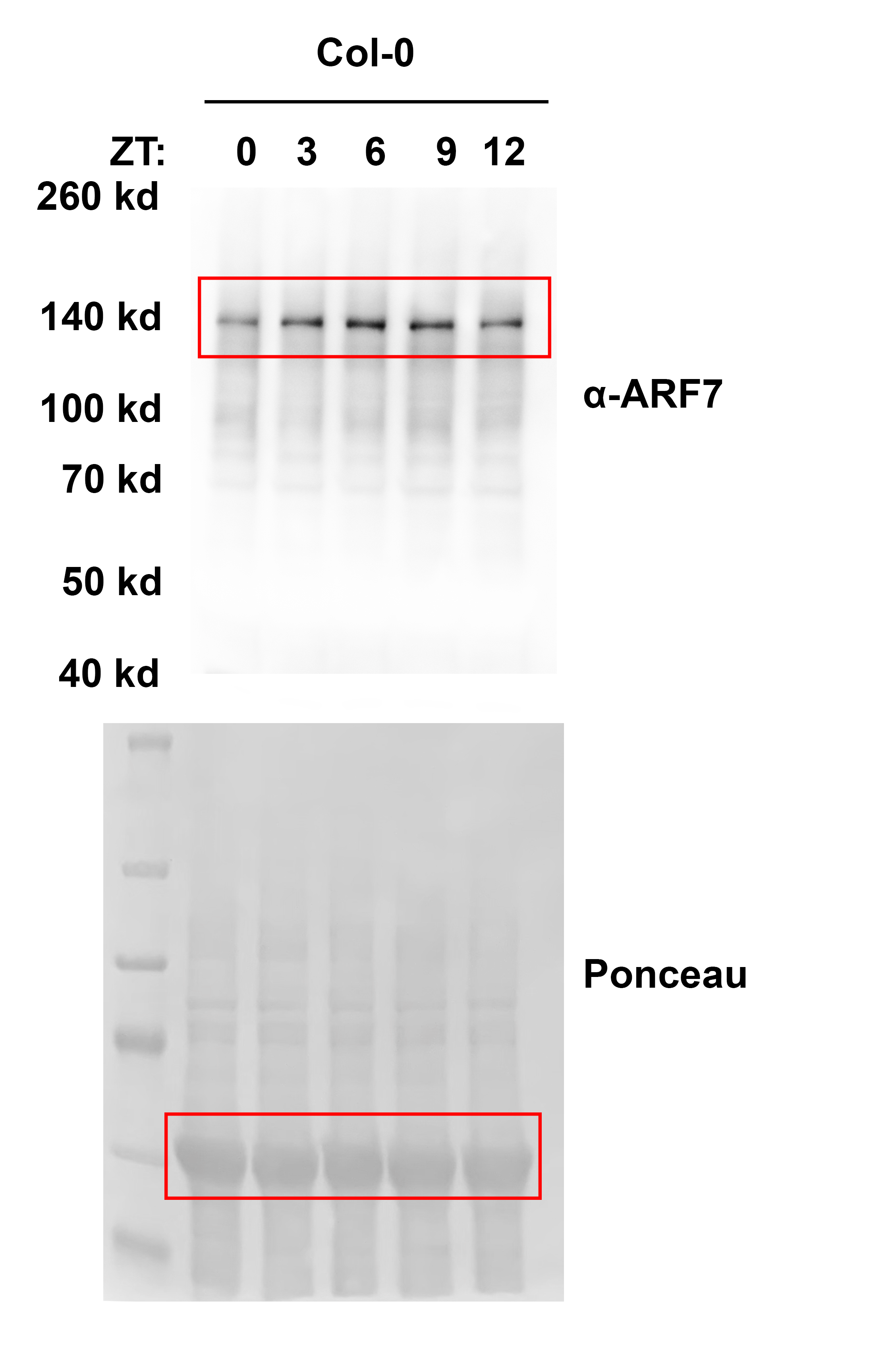

Supplement: Supplementary file 4 — Source data Fig. 4 [file 44319_2024_142_MOESM4_ESM.zip › Figure 4/4A/Western blot oscillation ARF7 in Col-0.tif]

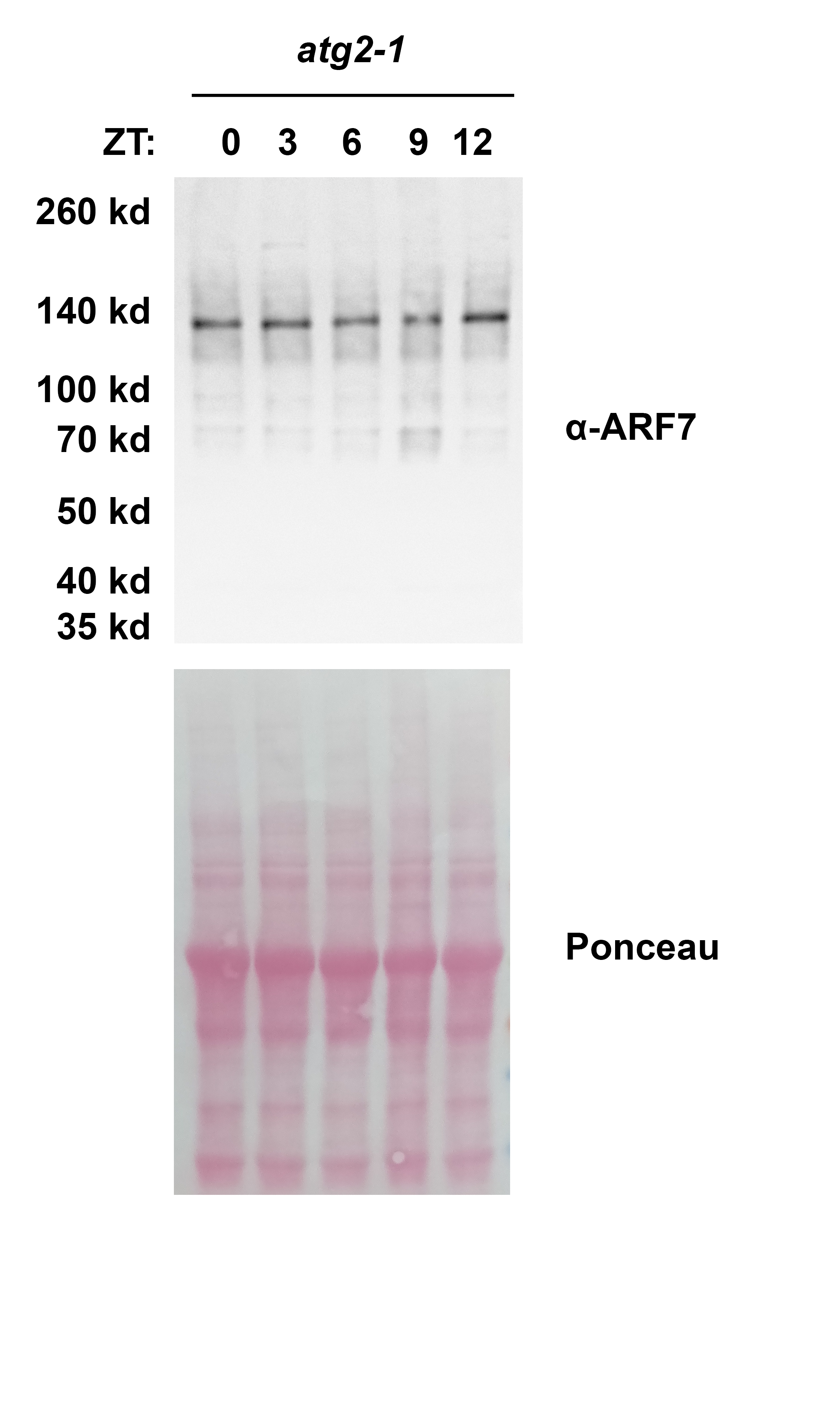

Supplement: Supplementary file 4 — Source data Fig. 4 [file 44319_2024_142_MOESM4_ESM.zip › Figure 4/4B/replicate/Western blot oscillation ARF7 in atg2-1 replicate 1.tif]

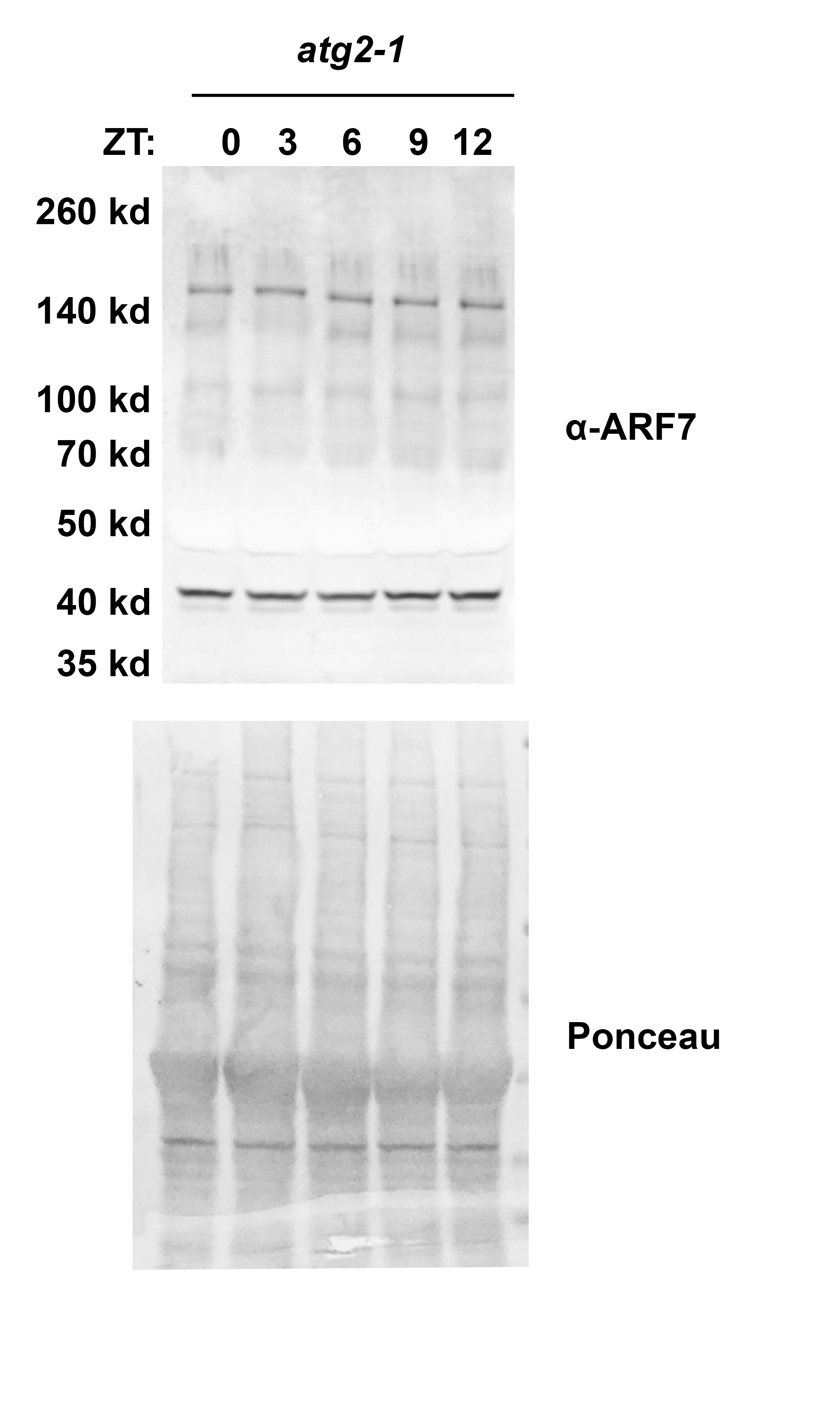

Supplement: Supplementary file 4 — Source data Fig. 4 [file 44319_2024_142_MOESM4_ESM.zip › Figure 4/4B/replicate/Western blot oscillation ARF7 in atg2-1 replicate 2.tif]

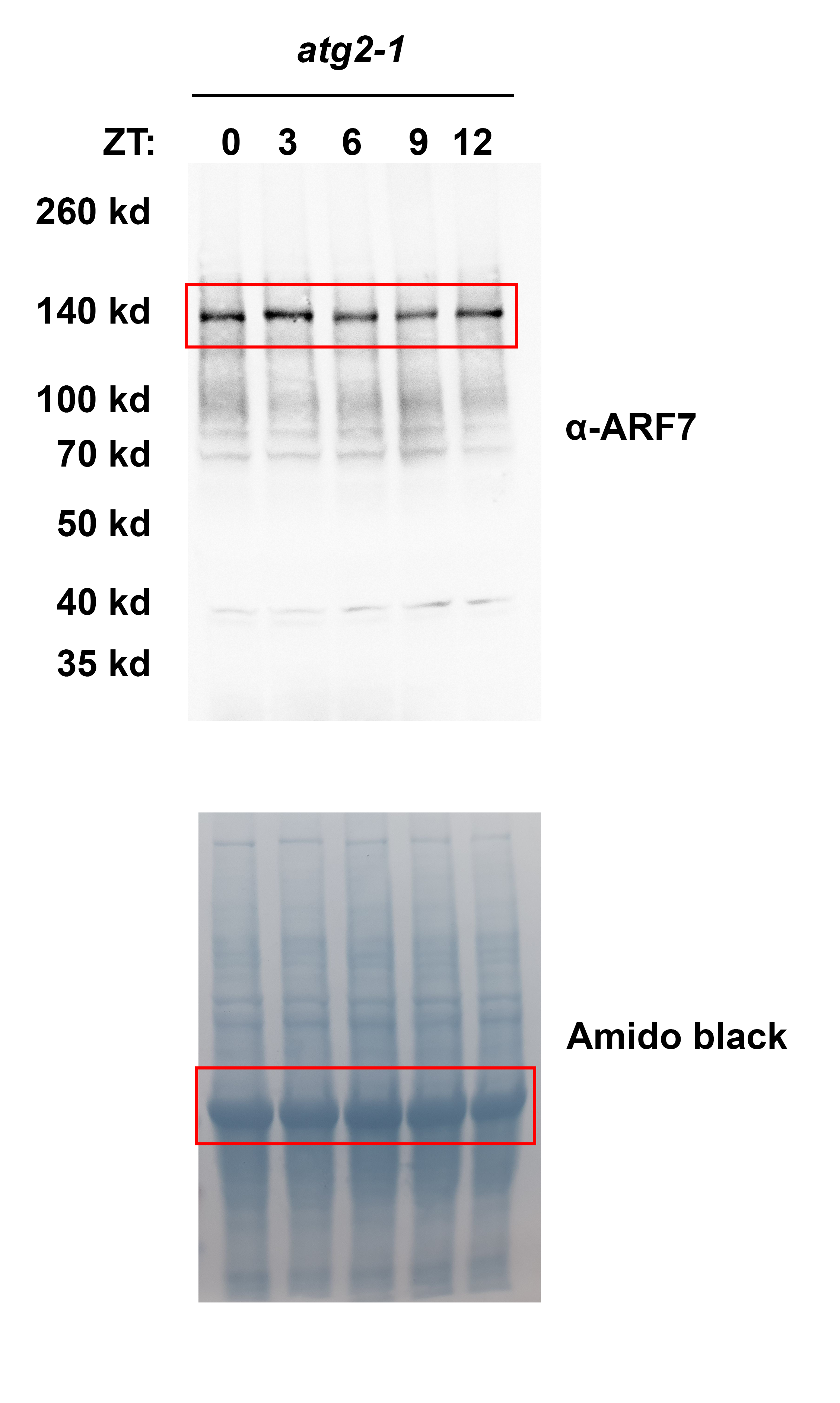

Supplement: Supplementary file 4 — Source data Fig. 4 [file 44319_2024_142_MOESM4_ESM.zip › Figure 4/4B/Western blot oscillation ARF7 in atg2-1.tif]

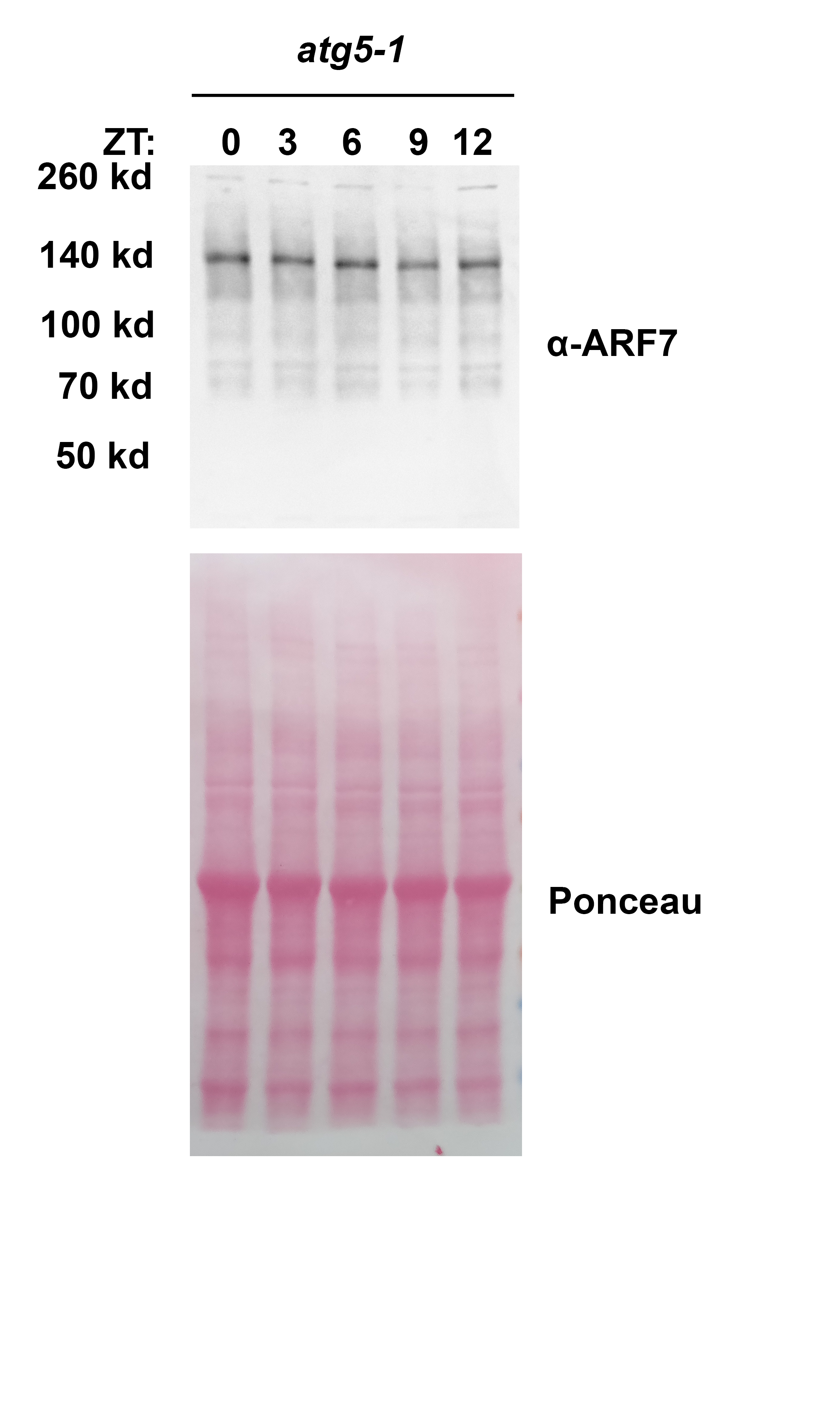

Supplement: Supplementary file 4 — Source data Fig. 4 [file 44319_2024_142_MOESM4_ESM.zip › Figure 4/4C/replicate/Western blot oscillation ARF7 in atg5-1 replicate 1.tif]

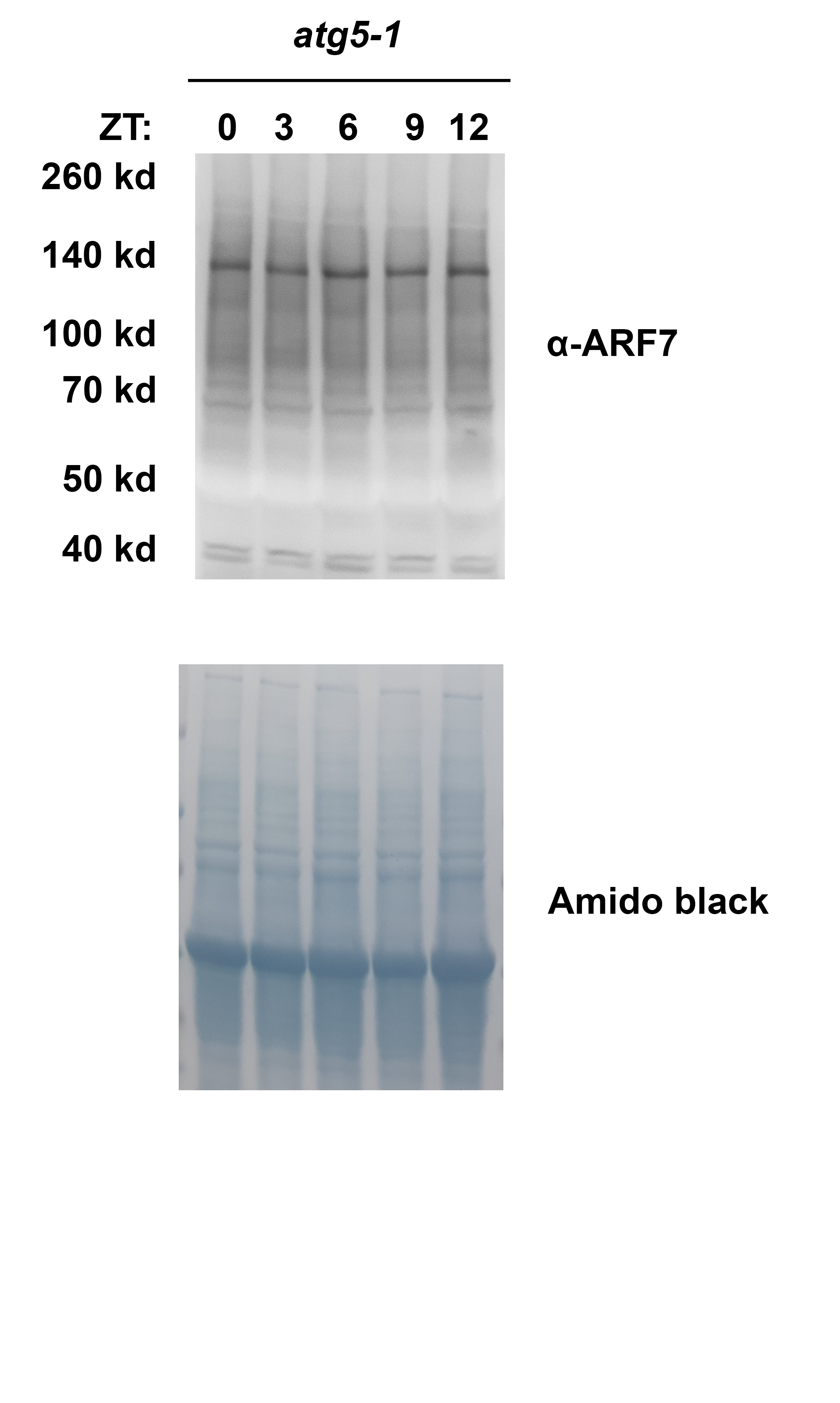

Supplement: Supplementary file 4 — Source data Fig. 4 [file 44319_2024_142_MOESM4_ESM.zip › Figure 4/4C/replicate/Western blot oscillation ARF7 in atg5-1 replicate 2.tif]

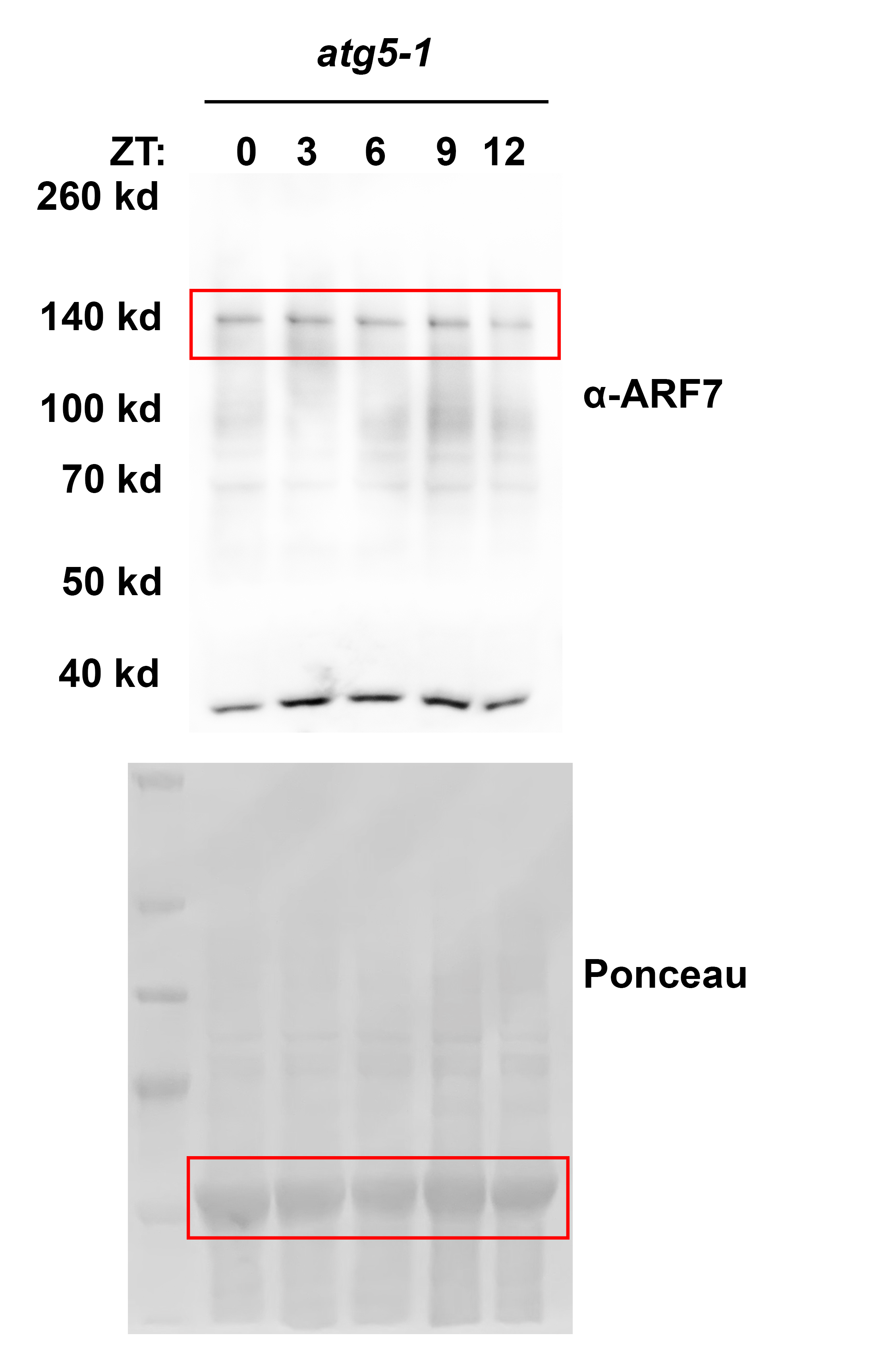

Supplement: Supplementary file 4 — Source data Fig. 4 [file 44319_2024_142_MOESM4_ESM.zip › Figure 4/4C/Western blot oscillation ARF7 in atg5-1.tif]

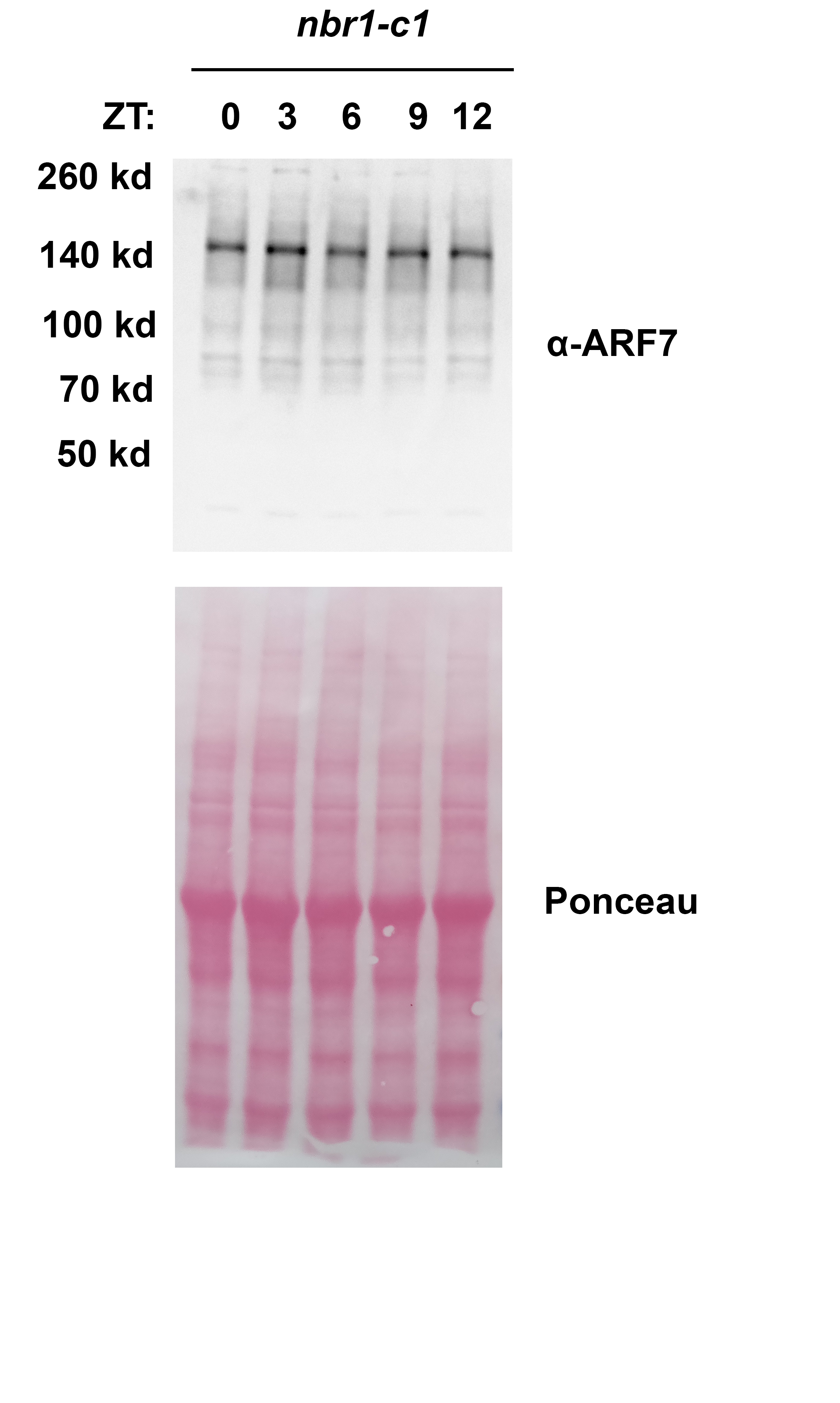

Supplement: Supplementary file 4 — Source data Fig. 4 [file 44319_2024_142_MOESM4_ESM.zip › Figure 4/4D/replicate/Western blot oscillation ARF7 in nbr1 replicate 1.tif]

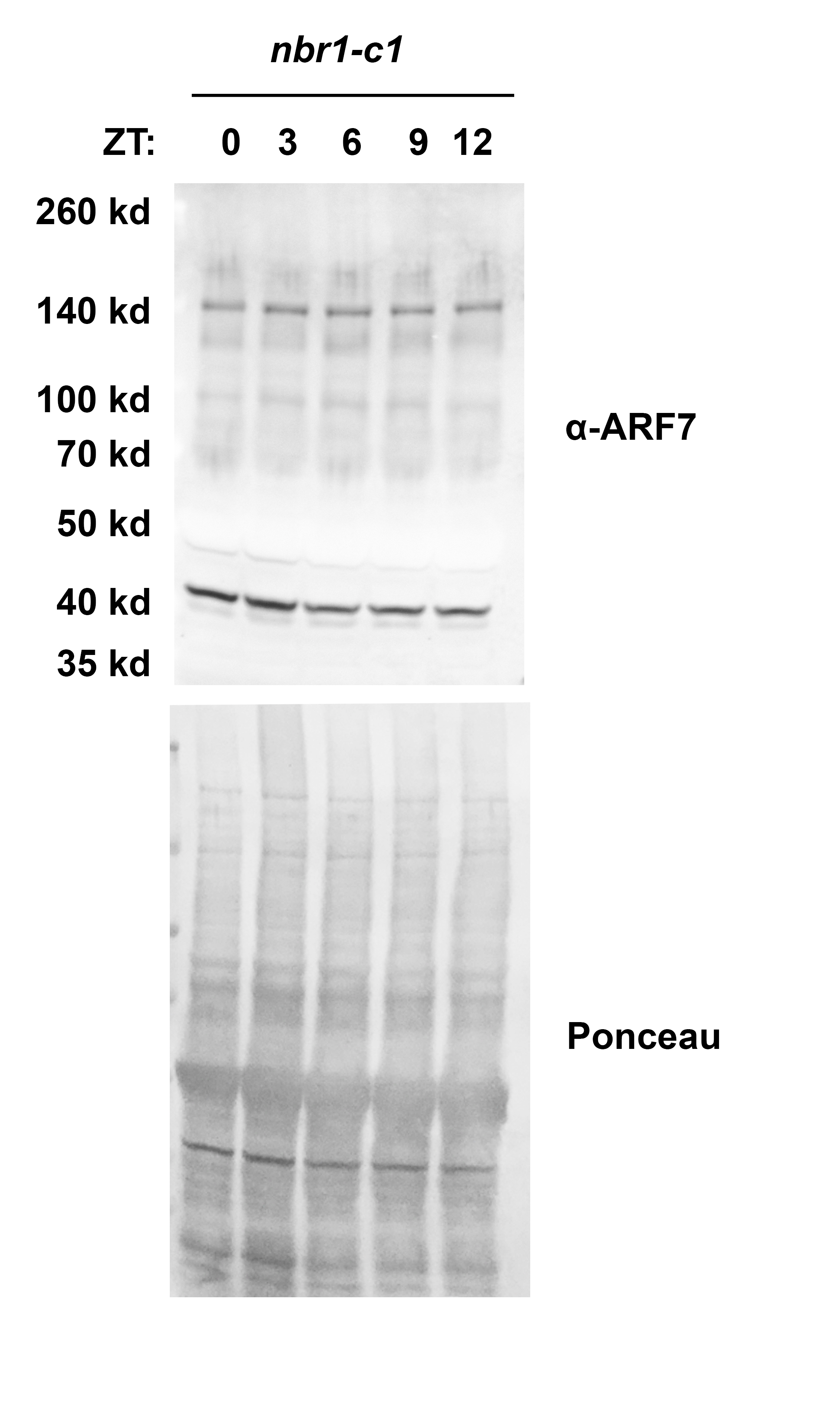

Supplement: Supplementary file 4 — Source data Fig. 4 [file 44319_2024_142_MOESM4_ESM.zip › Figure 4/4D/replicate/Western blot oscillation ARF7 in nbr1 replicate 2.tif]

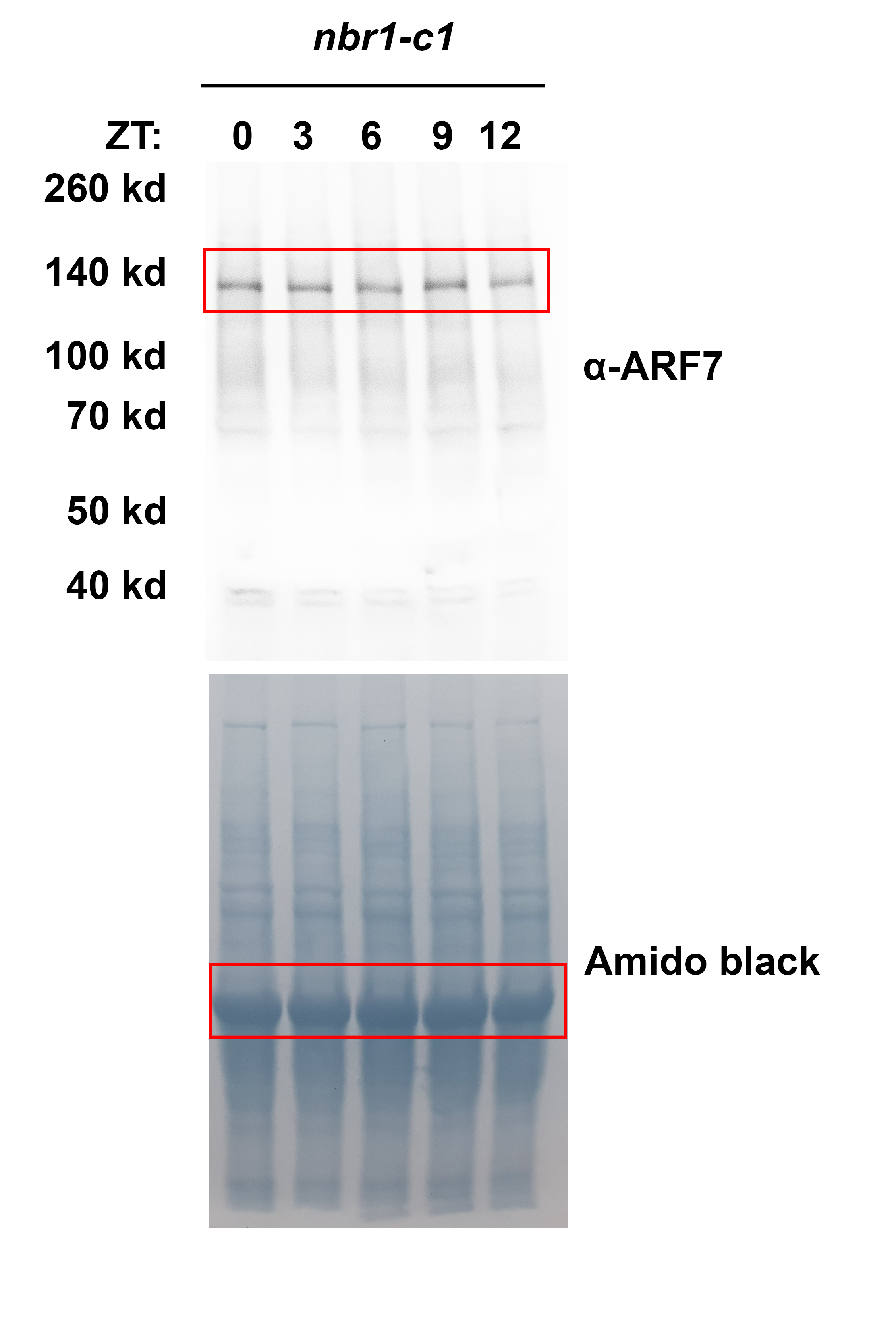

Supplement: Supplementary file 4 — Source data Fig. 4 [file 44319_2024_142_MOESM4_ESM.zip › Figure 4/4D/Western blot oscillation ARF7 in nbr1.tif]

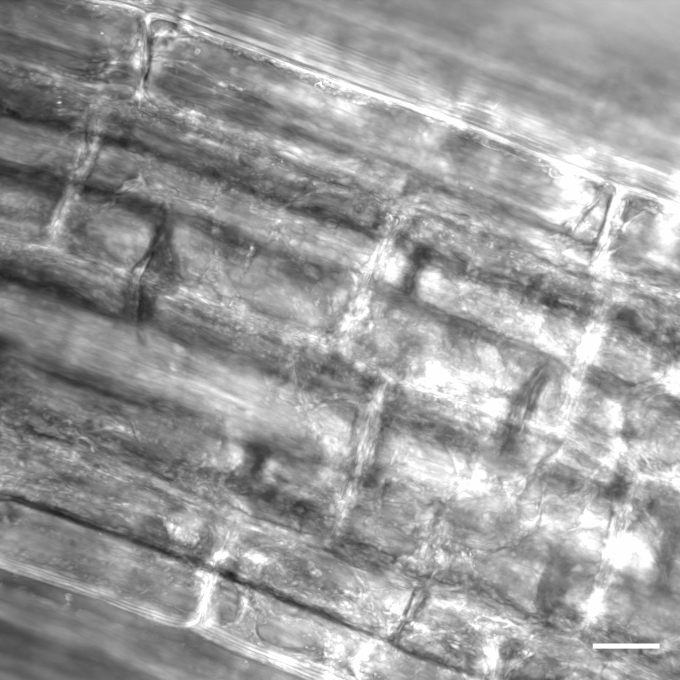

Supplement: Supplementary file 4 — Source data Fig. 4 [file 44319_2024_142_MOESM4_ESM.zip › Figure 4/4J/ARF7-Venus atg2-1 Oscillation Zone Fluorescence Bright-Field.tif]

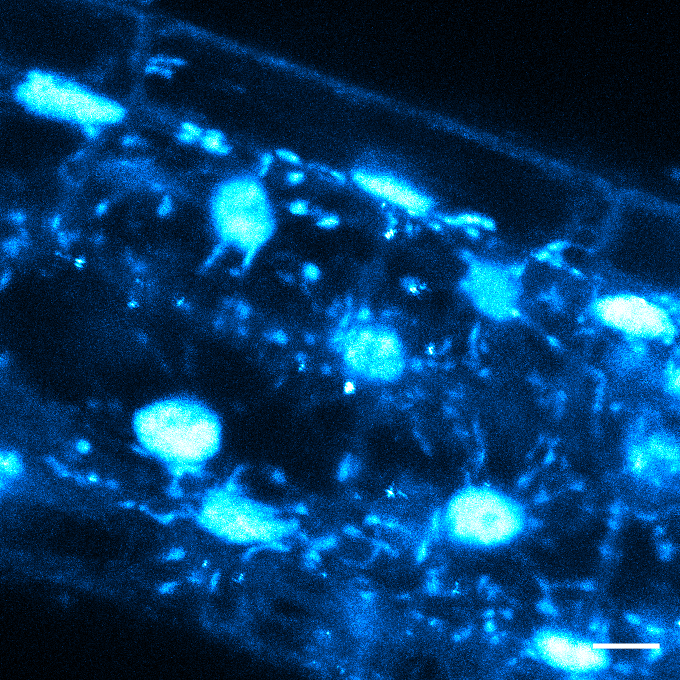

Supplement: Supplementary file 4 — Source data Fig. 4 [file 44319_2024_142_MOESM4_ESM.zip › Figure 4/4J/ARF7-Venus atg2-1 Oscillation Zone Fluorescence Venus.tif]

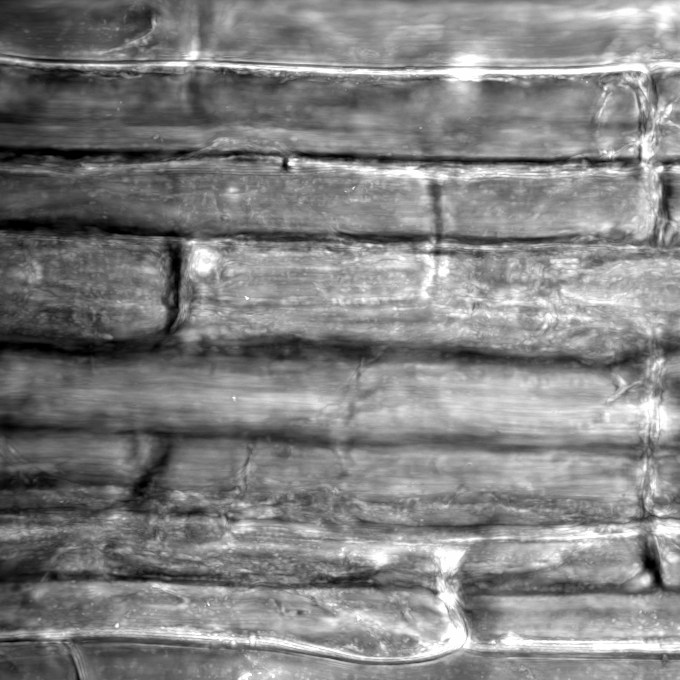

Supplement: Supplementary file 4 — Source data Fig. 4 [file 44319_2024_142_MOESM4_ESM.zip › Figure 4/4J/ARF7-Venus Oscillation Zone Fluorescence Bright-Field.tif]

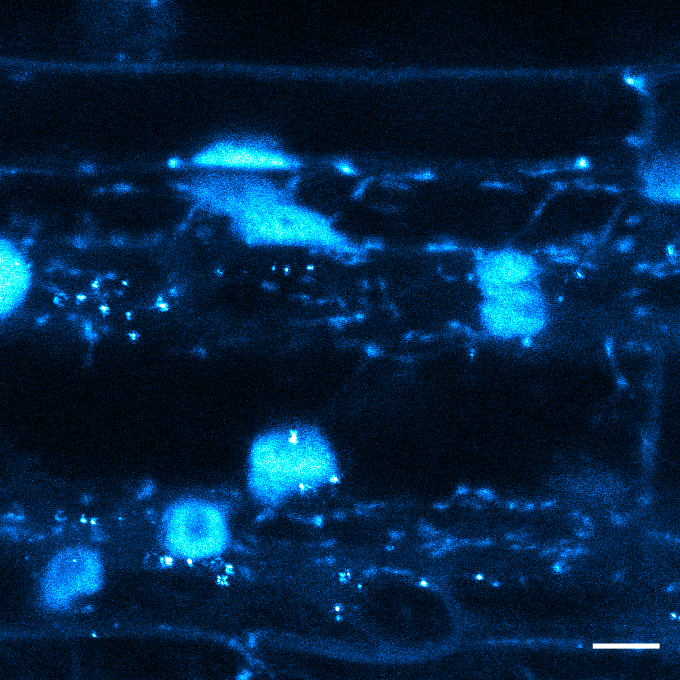

Supplement: Supplementary file 4 — Source data Fig. 4 [file 44319_2024_142_MOESM4_ESM.zip › Figure 4/4J/ARF7-Venus Oscillation Zone Fluorescence Venus.tif]

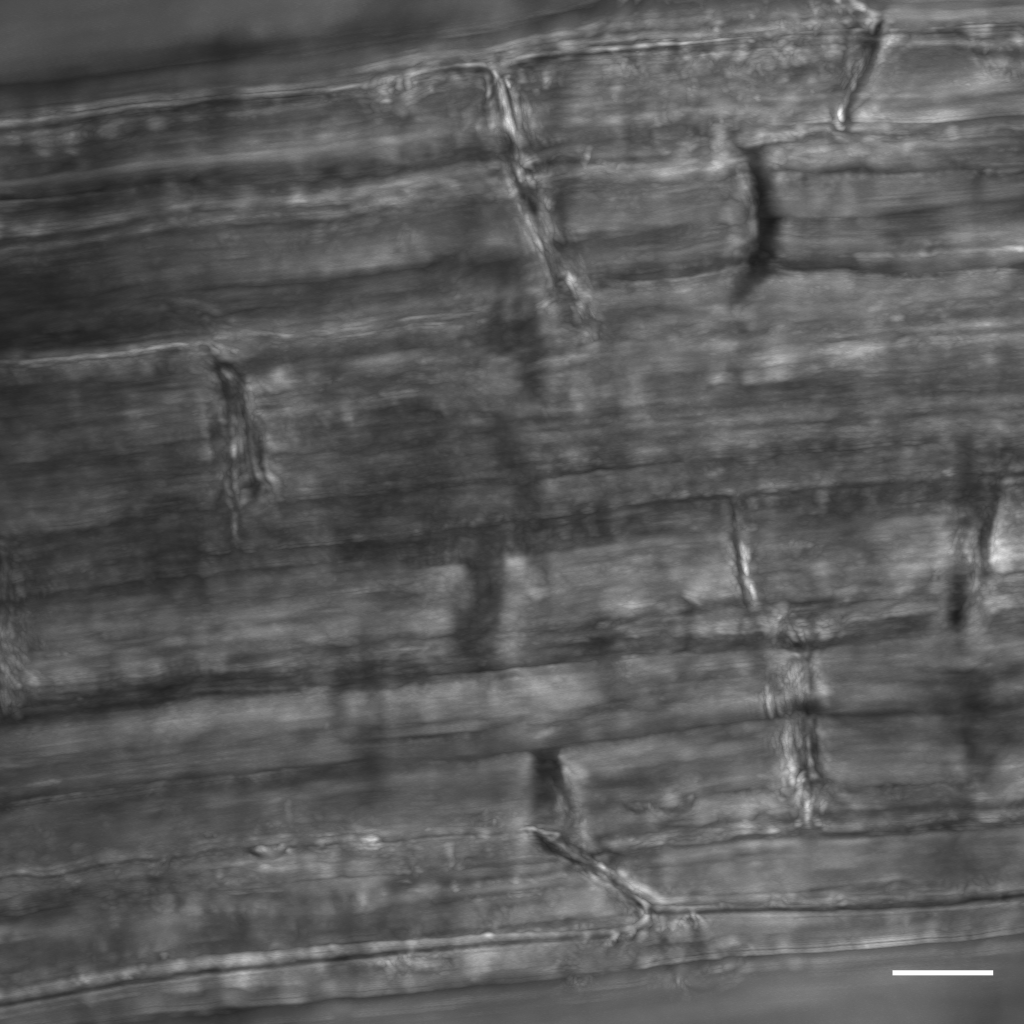

Supplement: Supplementary file 5 — Source data Fig. 5 [file 44319_2024_142_MOESM5_ESM.zip › Figure 5/5A/mCherry-ATG8 ARF7-Venus Mature Zone NAA Bright-Field.tif]

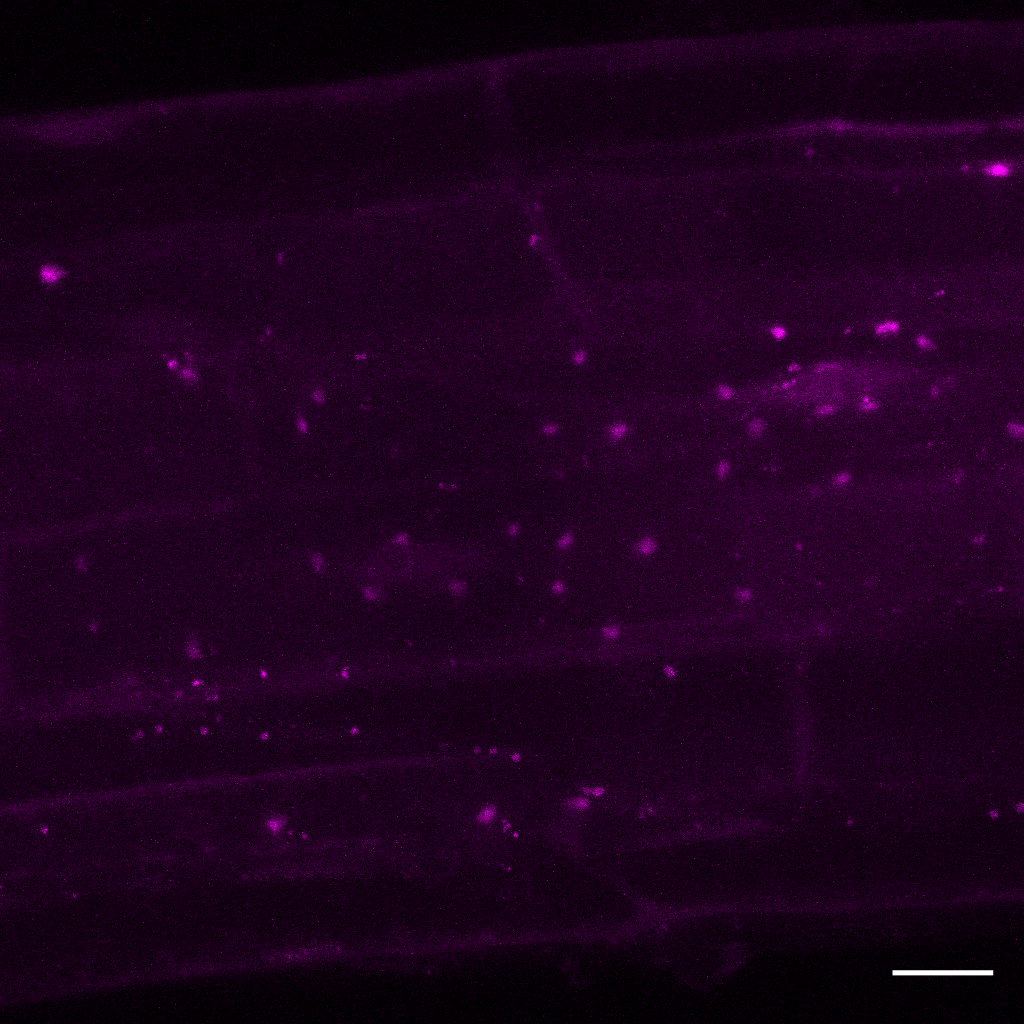

Supplement: Supplementary file 5 — Source data Fig. 5 [file 44319_2024_142_MOESM5_ESM.zip › Figure 5/5A/mCherry-ATG8 ARF7-Venus Mature Zone NAA mCherry.tif]

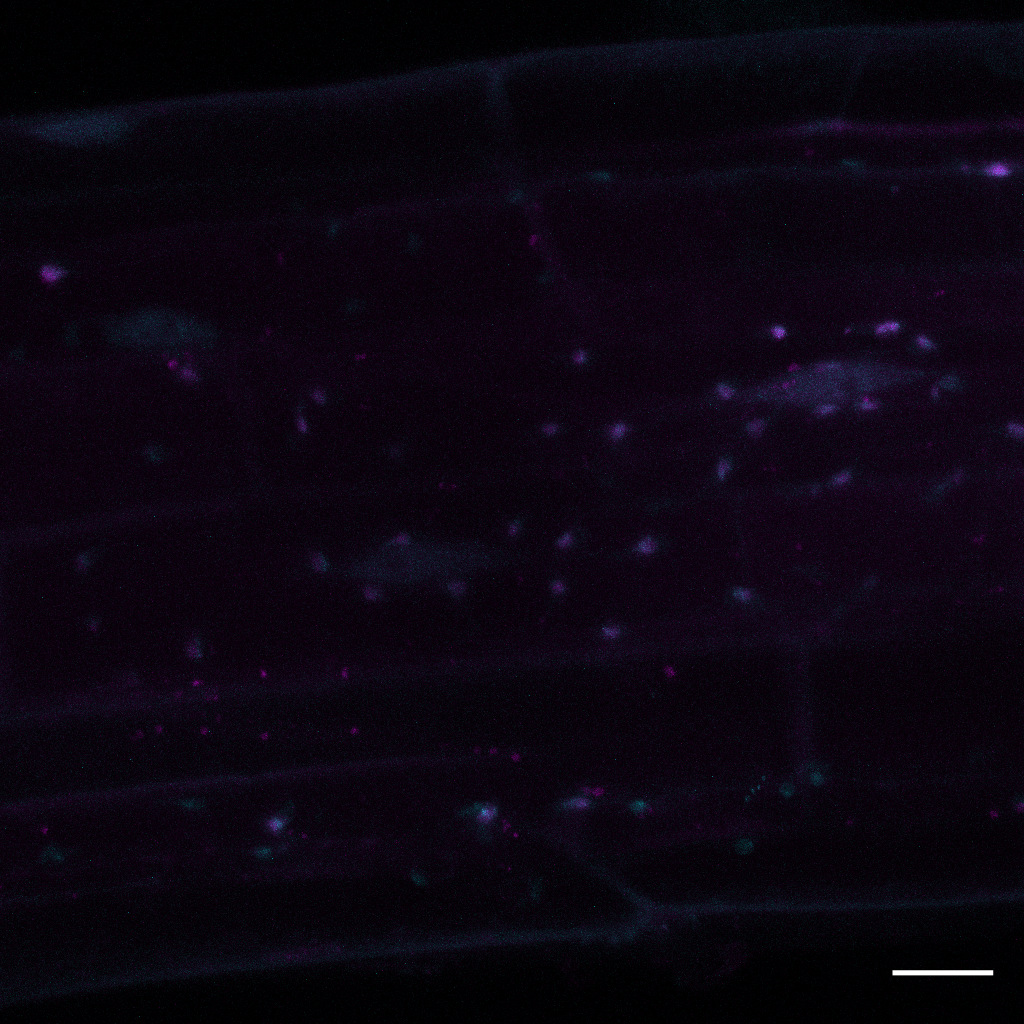

Supplement: Supplementary file 5 — Source data Fig. 5 [file 44319_2024_142_MOESM5_ESM.zip › Figure 5/5A/mCherry-ATG8 ARF7-Venus Mature Zone NAA Merged.tif]

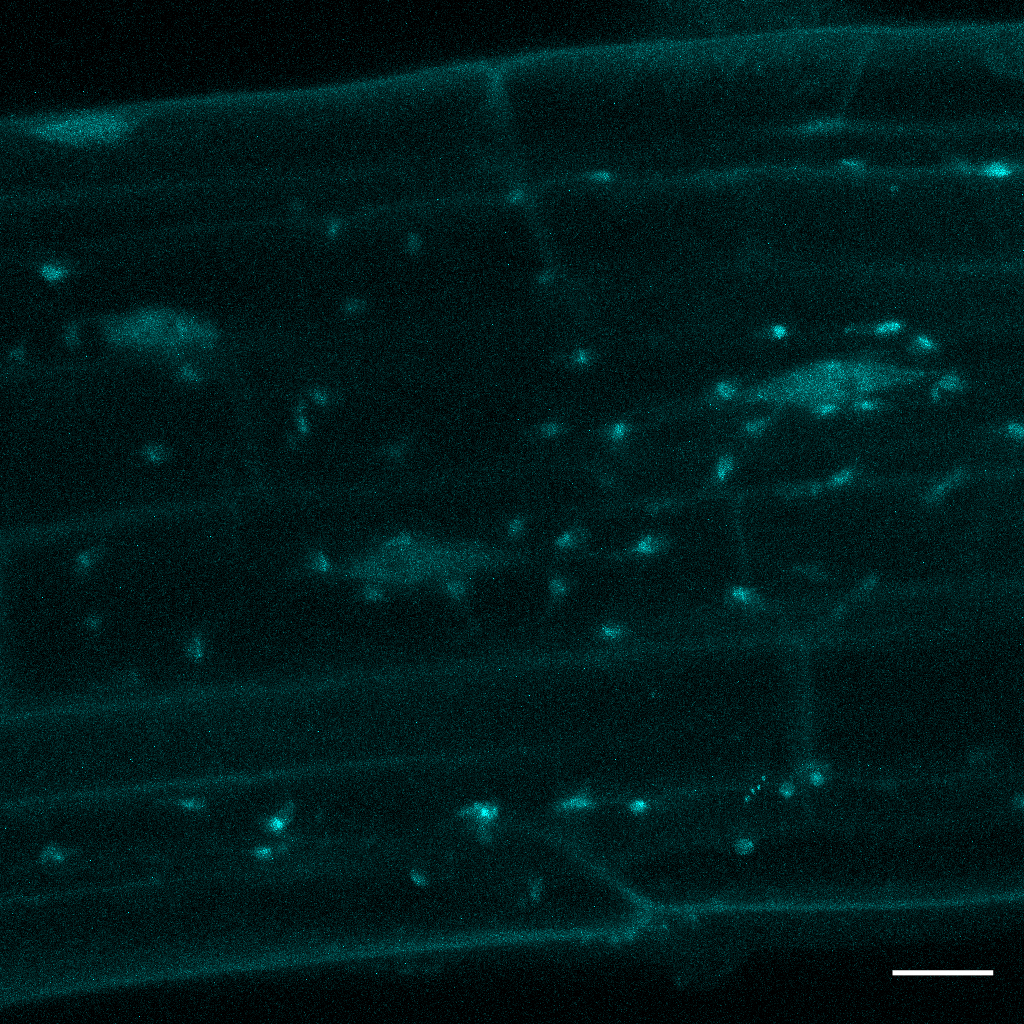

Supplement: Supplementary file 5 — Source data Fig. 5 [file 44319_2024_142_MOESM5_ESM.zip › Figure 5/5A/mCherry-ATG8 ARF7-Venus Mature Zone NAA YFP.tif]

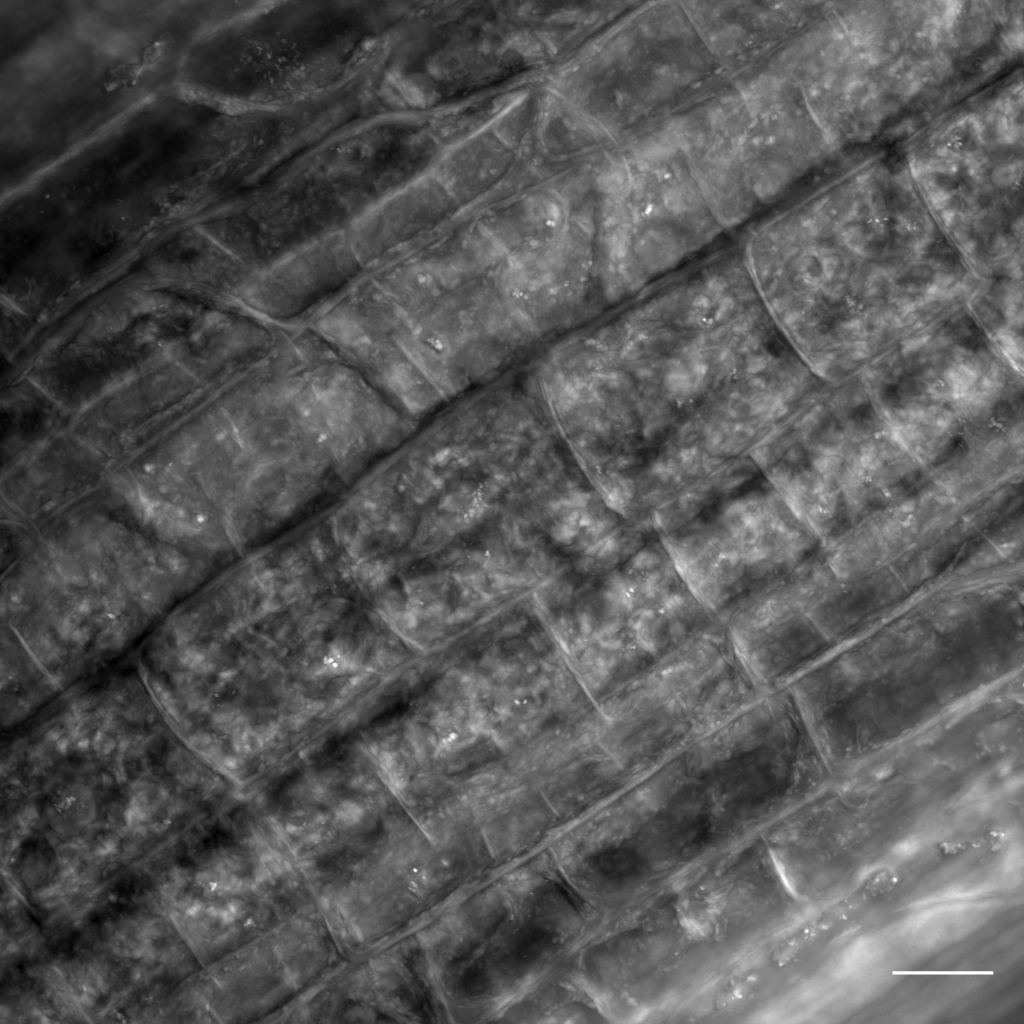

Supplement: Supplementary file 5 — Source data Fig. 5 [file 44319_2024_142_MOESM5_ESM.zip › Figure 5/5A/mCherry-ATG8 ARF7-Venus Meristem NAA Bright-Field.tif]

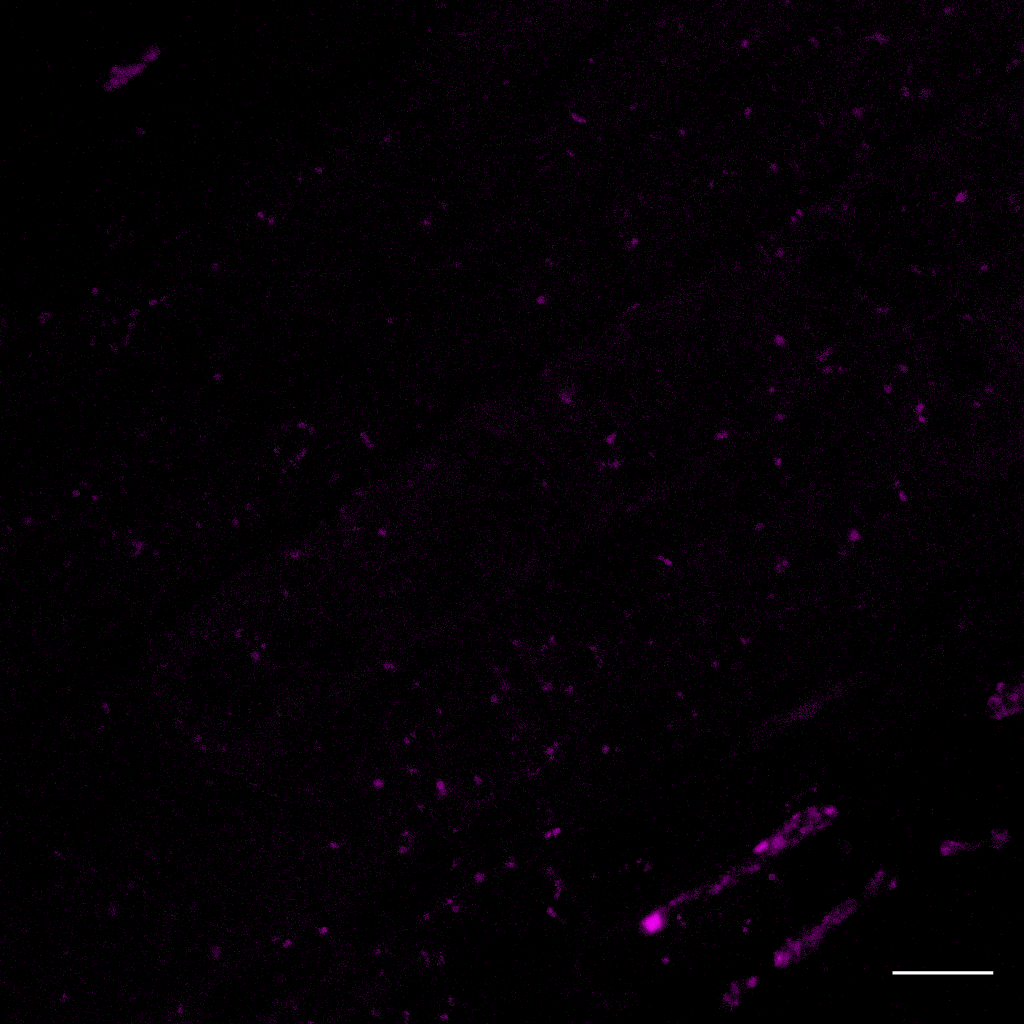

Supplement: Supplementary file 5 — Source data Fig. 5 [file 44319_2024_142_MOESM5_ESM.zip › Figure 5/5A/mCherry-ATG8 ARF7-Venus Meristem NAA mCherry.tif]

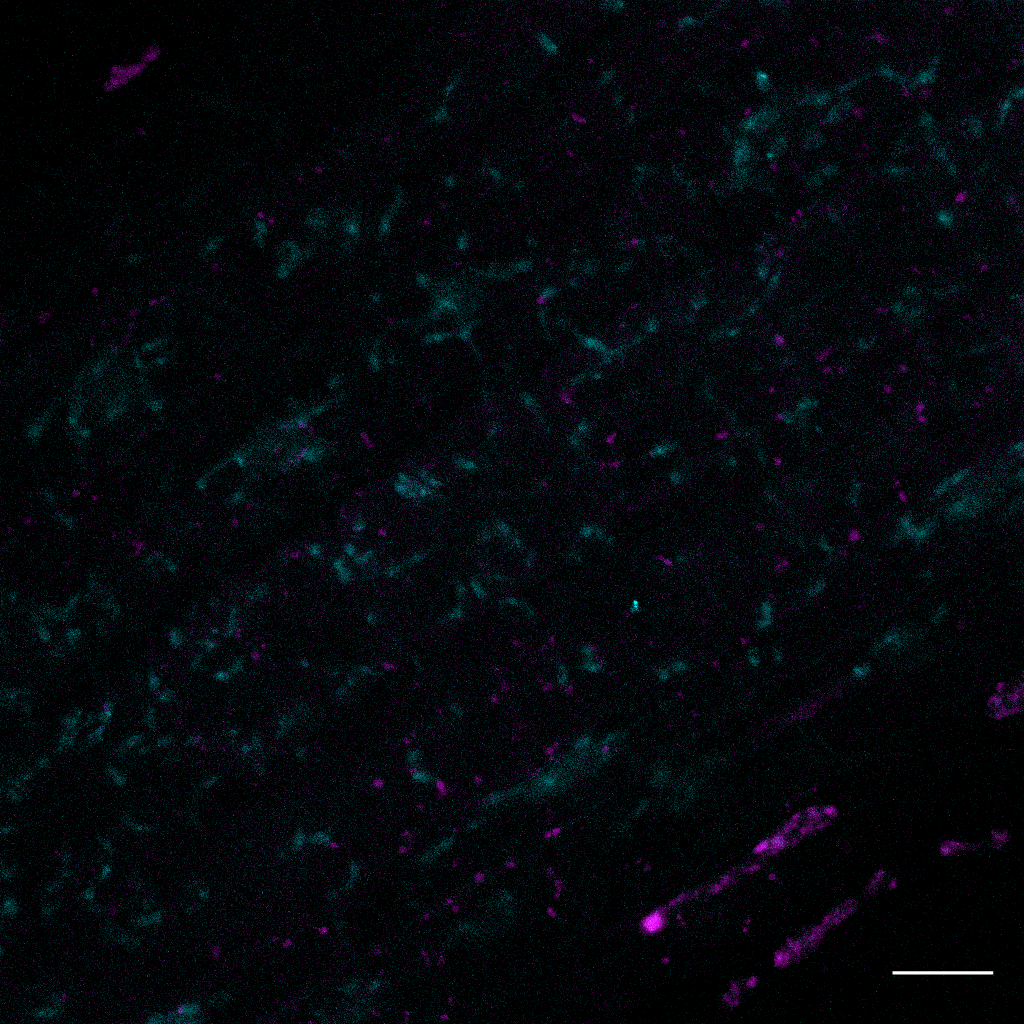

Supplement: Supplementary file 5 — Source data Fig. 5 [file 44319_2024_142_MOESM5_ESM.zip › Figure 5/5A/mCherry-ATG8 ARF7-Venus Meristem NAA Merged.tif]

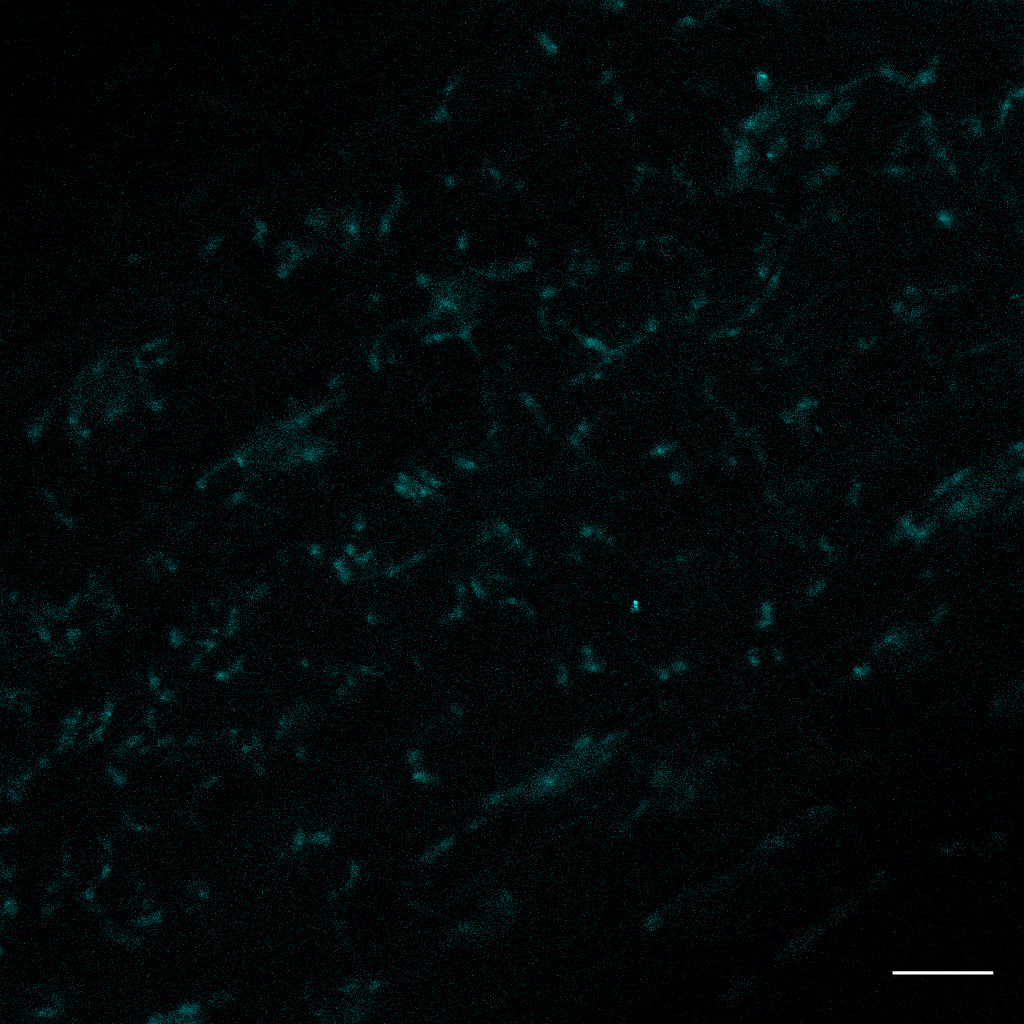

Supplement: Supplementary file 5 — Source data Fig. 5 [file 44319_2024_142_MOESM5_ESM.zip › Figure 5/5A/mCherry-ATG8 ARF7-Venus Meristem NAA Venus.tif]

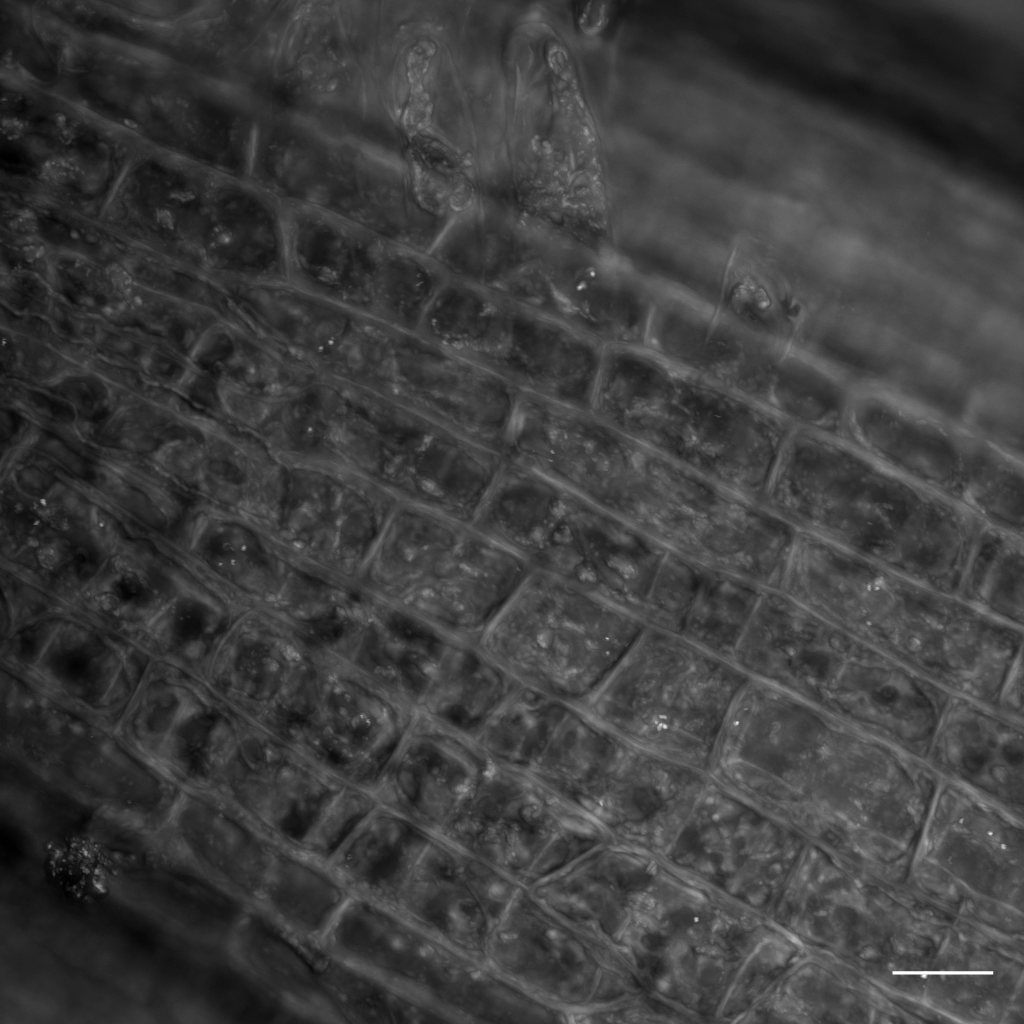

Supplement: Supplementary file 5 — Source data Fig. 5 [file 44319_2024_142_MOESM5_ESM.zip › Figure 5/5A/mCherry-ATG8 ARF7-Venus Meristem NT Bright-Field.tif]

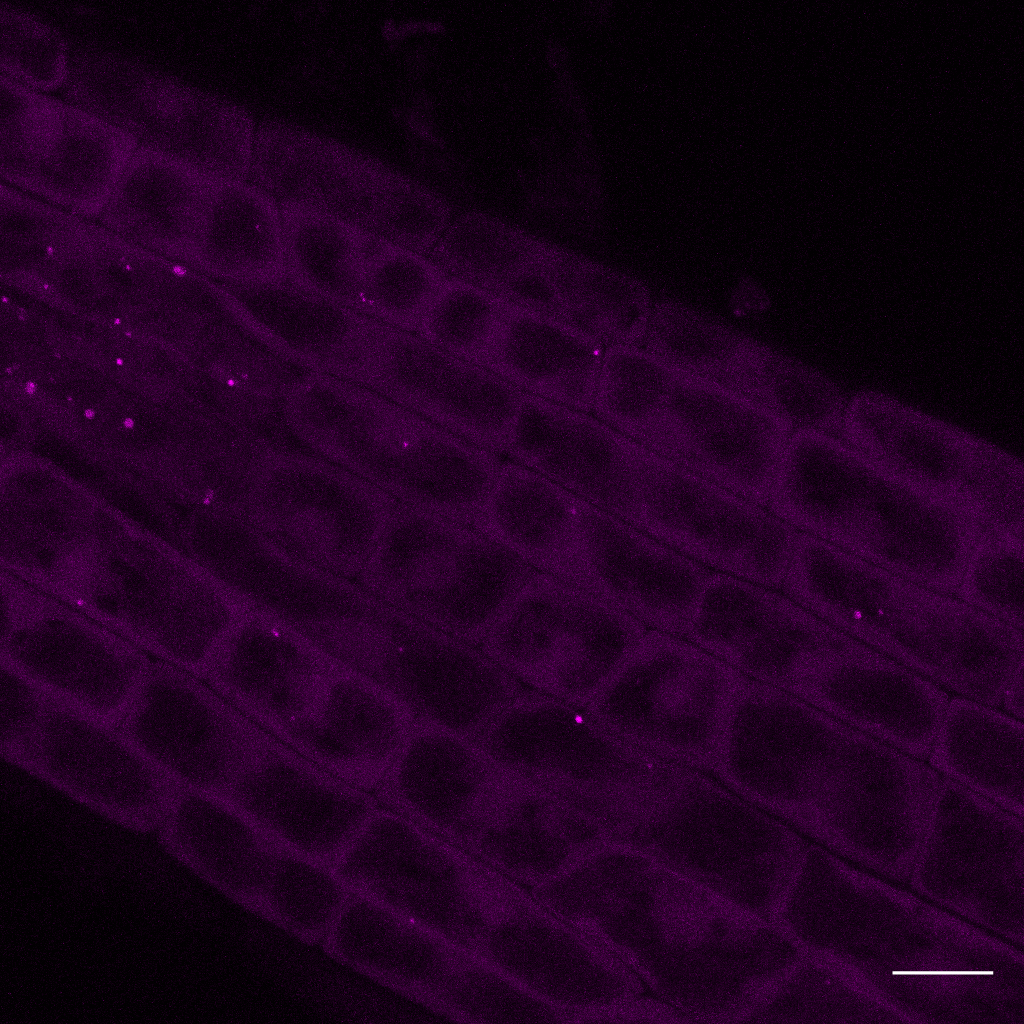

Supplement: Supplementary file 5 — Source data Fig. 5 [file 44319_2024_142_MOESM5_ESM.zip › Figure 5/5A/mCherry-ATG8 ARF7-Venus Meristem NT Mcherry.tif]

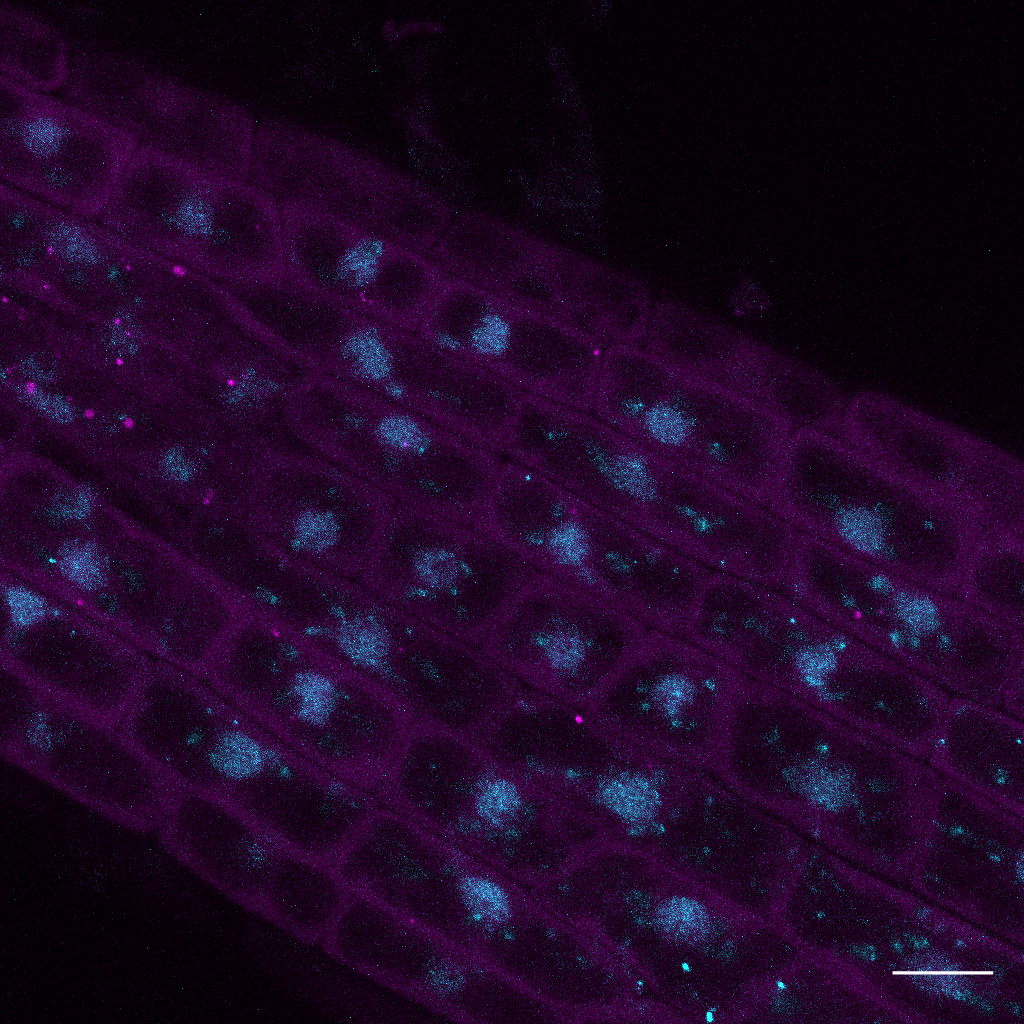

Supplement: Supplementary file 5 — Source data Fig. 5 [file 44319_2024_142_MOESM5_ESM.zip › Figure 5/5A/mCherry-ATG8 ARF7-Venus Meristem NT Merged.tif]

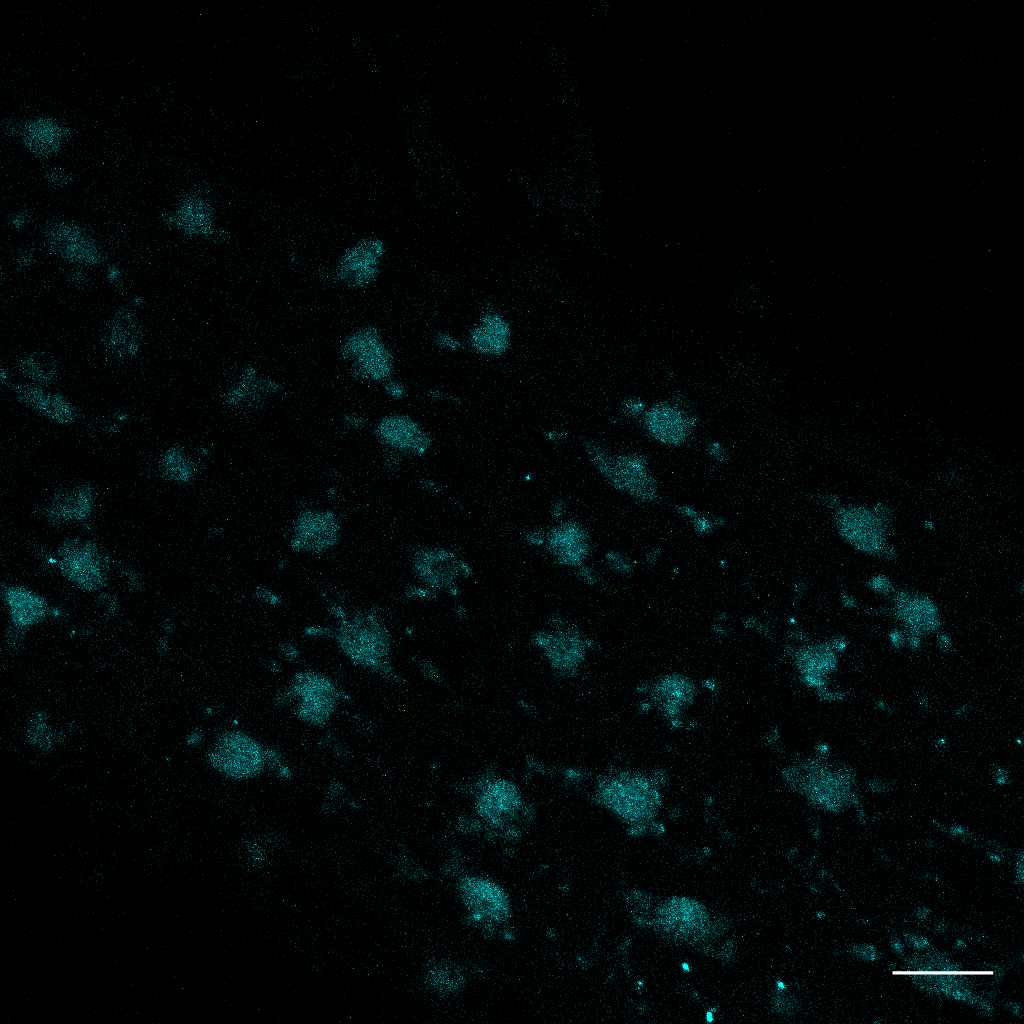

Supplement: Supplementary file 5 — Source data Fig. 5 [file 44319_2024_142_MOESM5_ESM.zip › Figure 5/5A/mCherry-ATG8 ARF7-Venus Meristem NT YFP.tif]

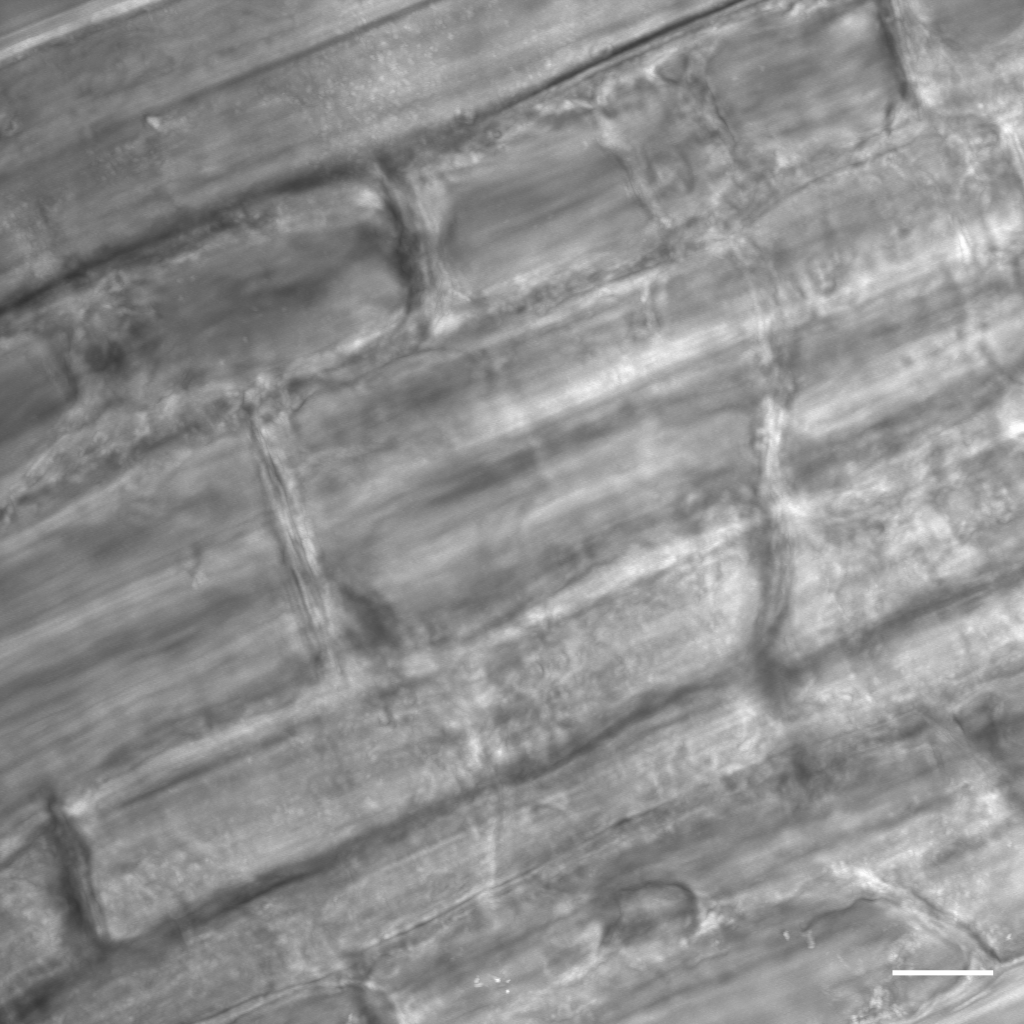

Supplement: Supplementary file 5 — Source data Fig. 5 [file 44319_2024_142_MOESM5_ESM.zip › Figure 5/5A/mCherry-ATG8 ARF7-Venus Oscillation Zone NAA Bright-Field.tif]

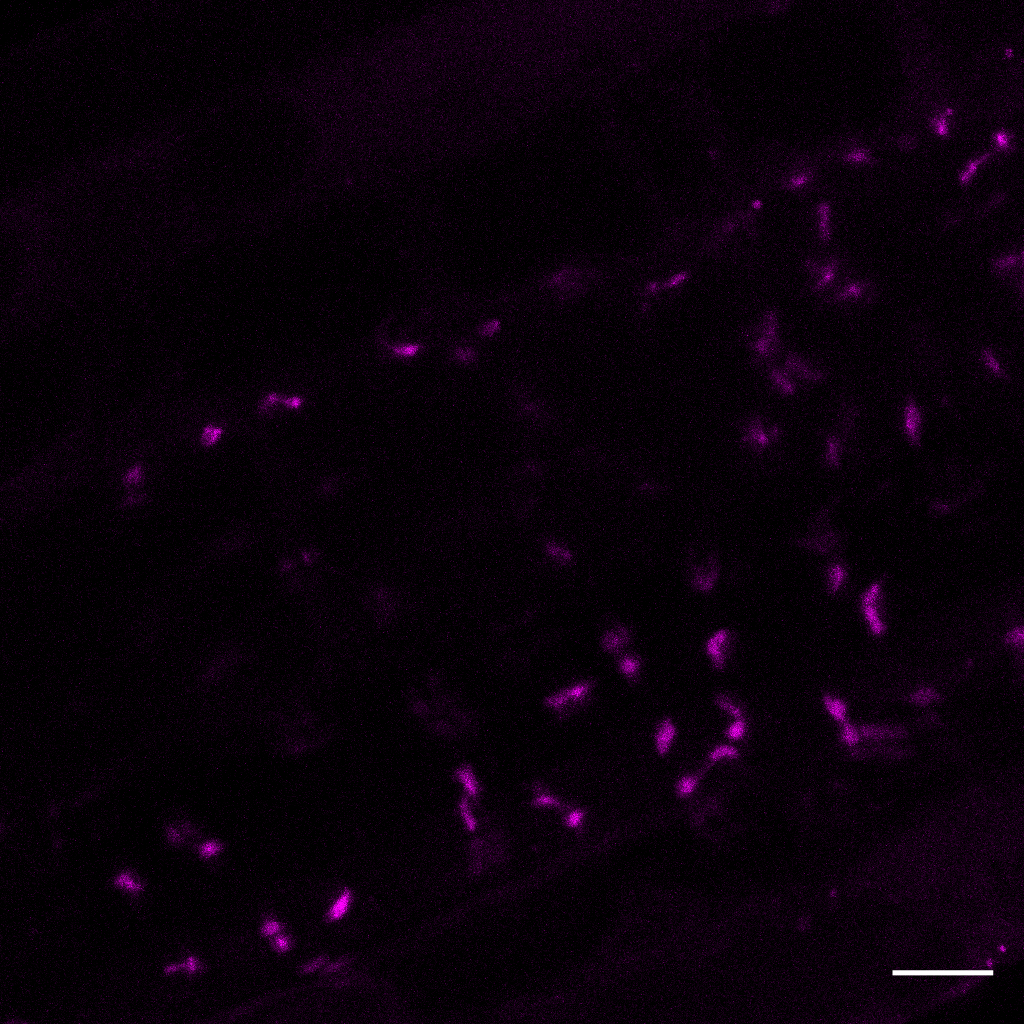

Supplement: Supplementary file 5 — Source data Fig. 5 [file 44319_2024_142_MOESM5_ESM.zip › Figure 5/5A/mCherry-ATG8 ARF7-Venus Oscillation Zone NAA mCherry.tif]

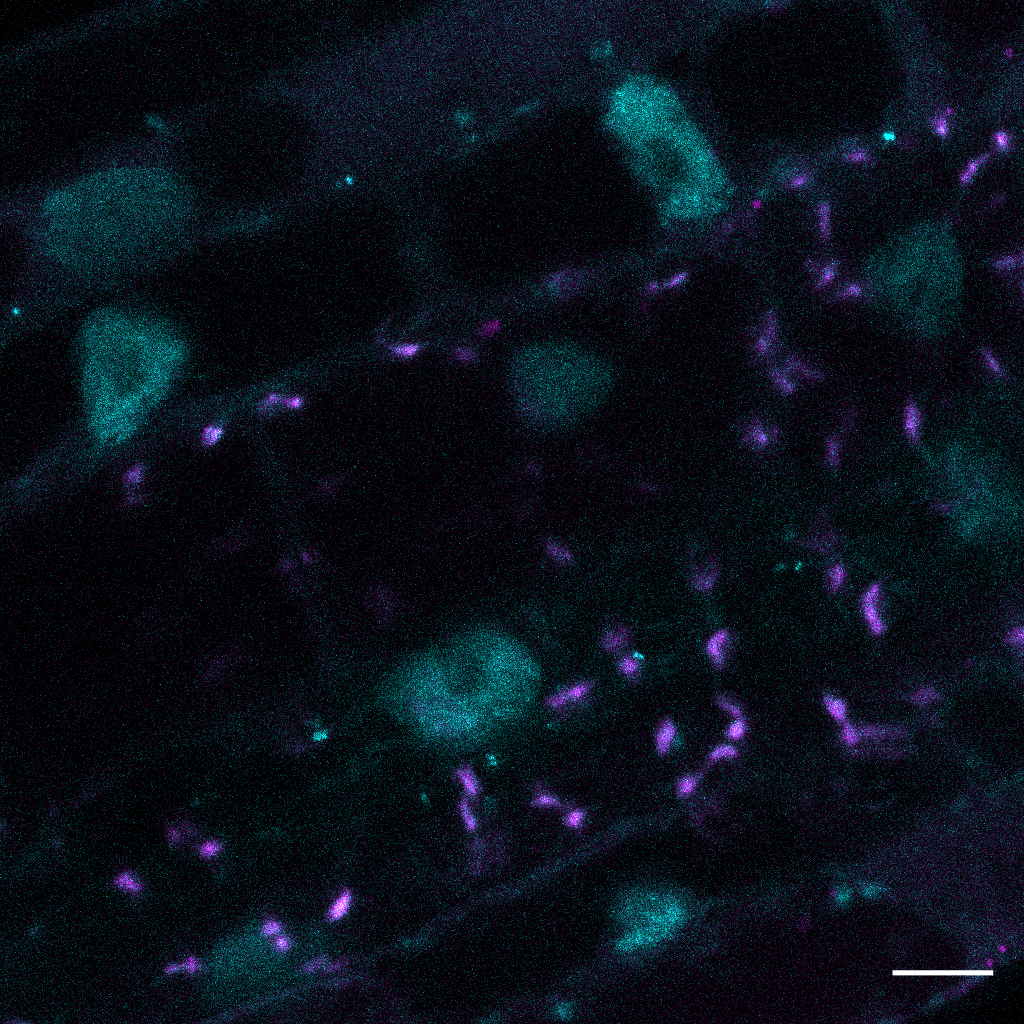

Supplement: Supplementary file 5 — Source data Fig. 5 [file 44319_2024_142_MOESM5_ESM.zip › Figure 5/5A/mCherry-ATG8 ARF7-Venus Oscillation Zone NAA Merged.tif]

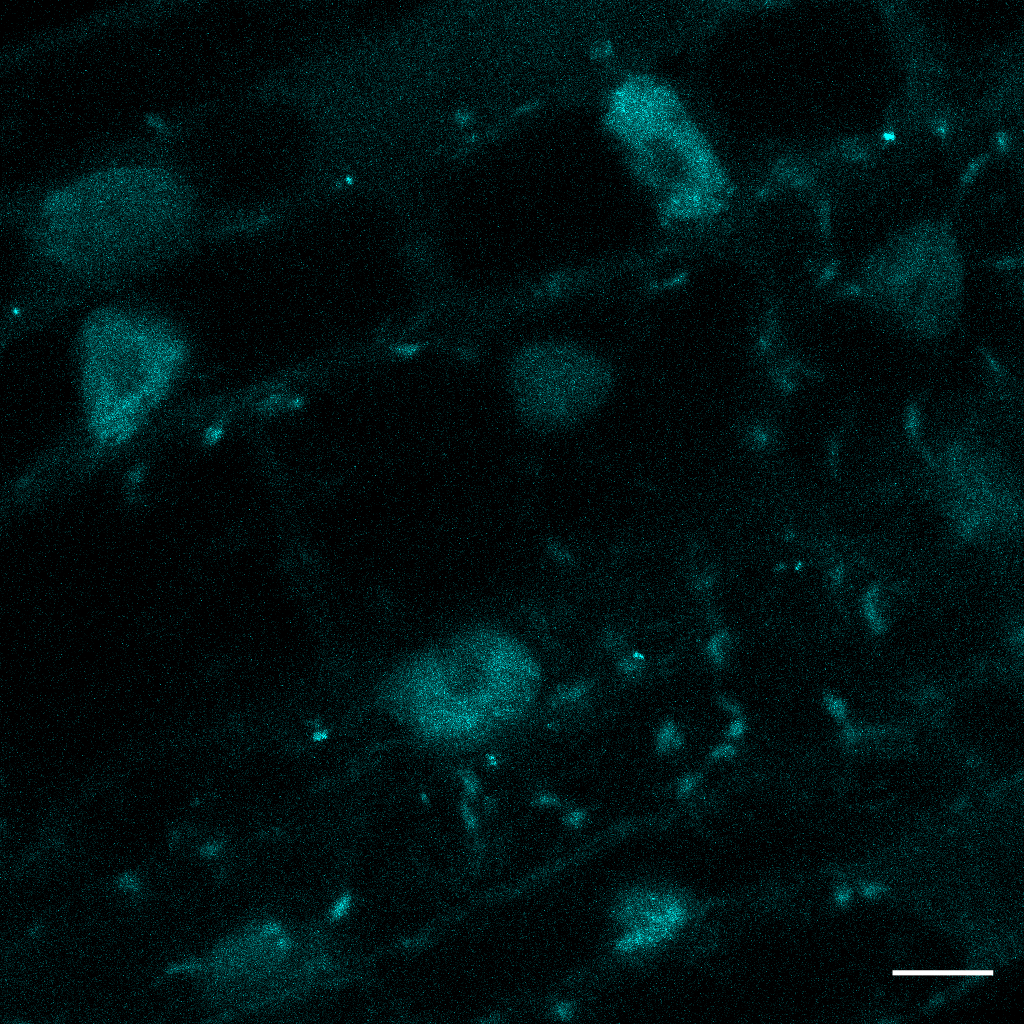

Supplement: Supplementary file 5 — Source data Fig. 5 [file 44319_2024_142_MOESM5_ESM.zip › Figure 5/5A/mCherry-ATG8 ARF7-Venus Oscillation Zone NAA YFP.tif]

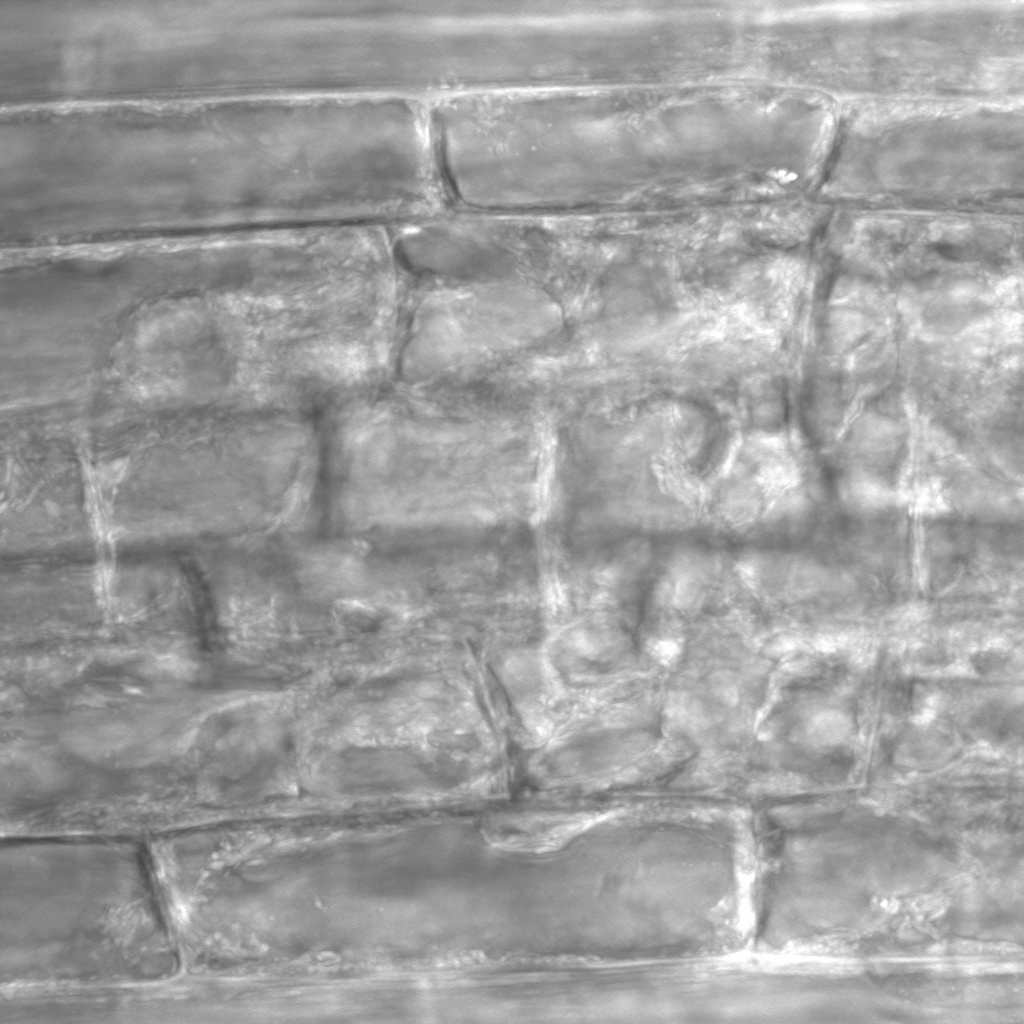

Supplement: Supplementary file 5 — Source data Fig. 5 [file 44319_2024_142_MOESM5_ESM.zip › Figure 5/5A/mCherry-ATG8 ARF7-Venus Oscillation Zone NT Bright-Field.tif]

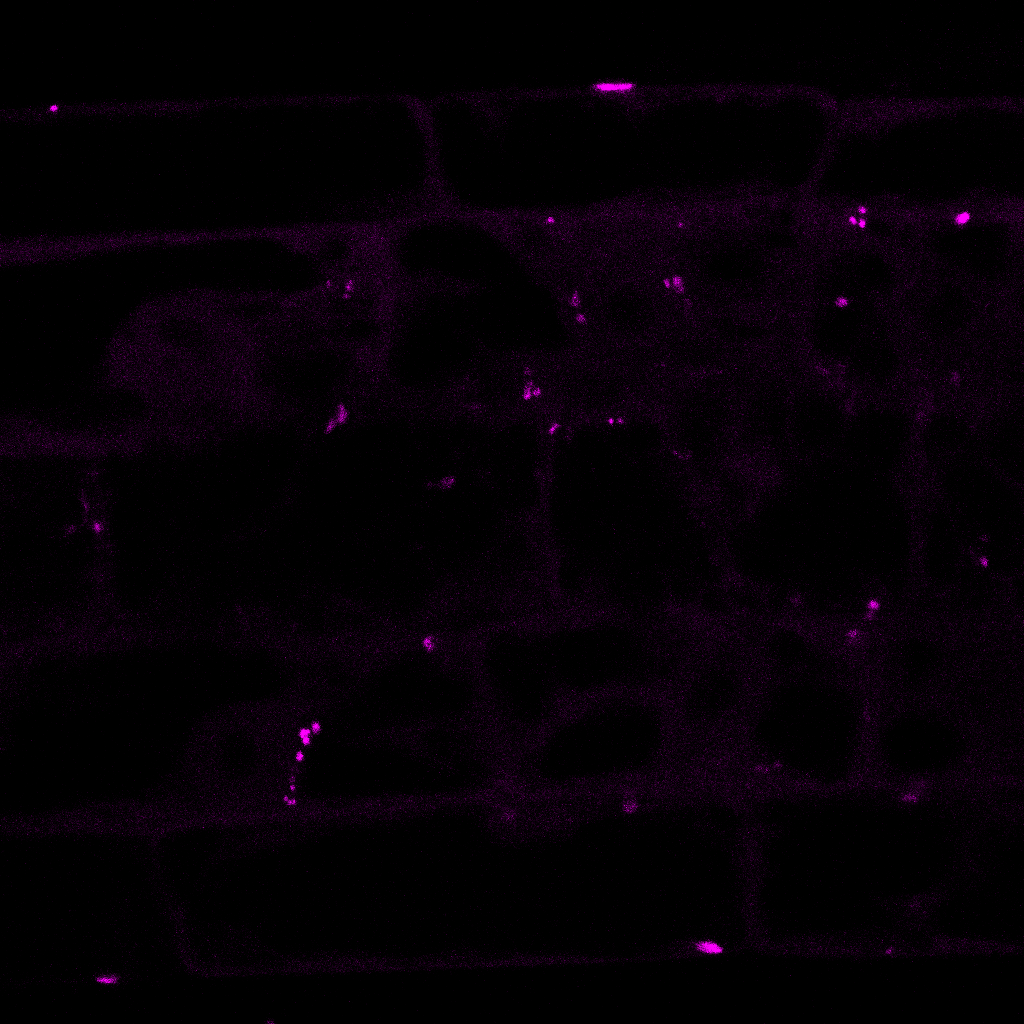

Supplement: Supplementary file 5 — Source data Fig. 5 [file 44319_2024_142_MOESM5_ESM.zip › Figure 5/5A/mCherry-ATG8 ARF7-Venus Oscillation Zone NT mCherry.tif]

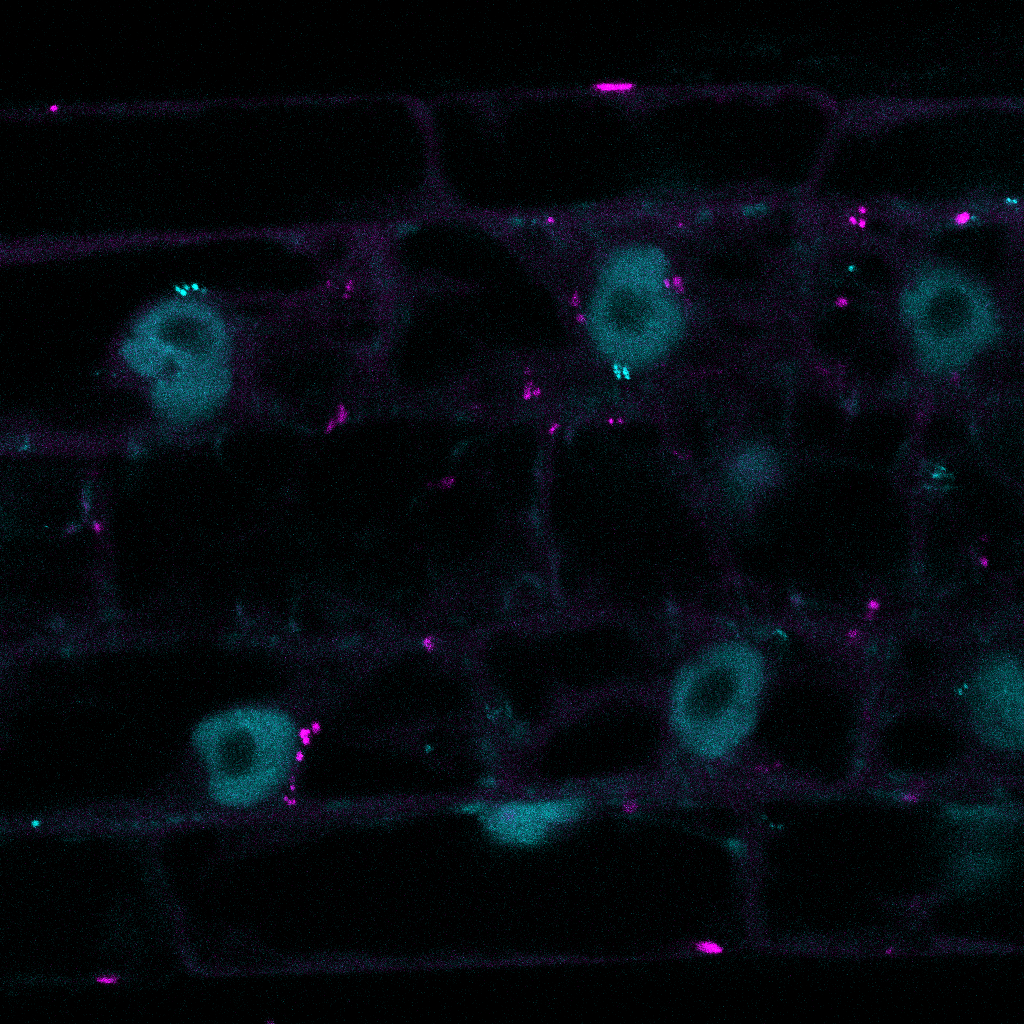

Supplement: Supplementary file 5 — Source data Fig. 5 [file 44319_2024_142_MOESM5_ESM.zip › Figure 5/5A/mCherry-ATG8 ARF7-Venus Oscillation Zone NT merged.tif]

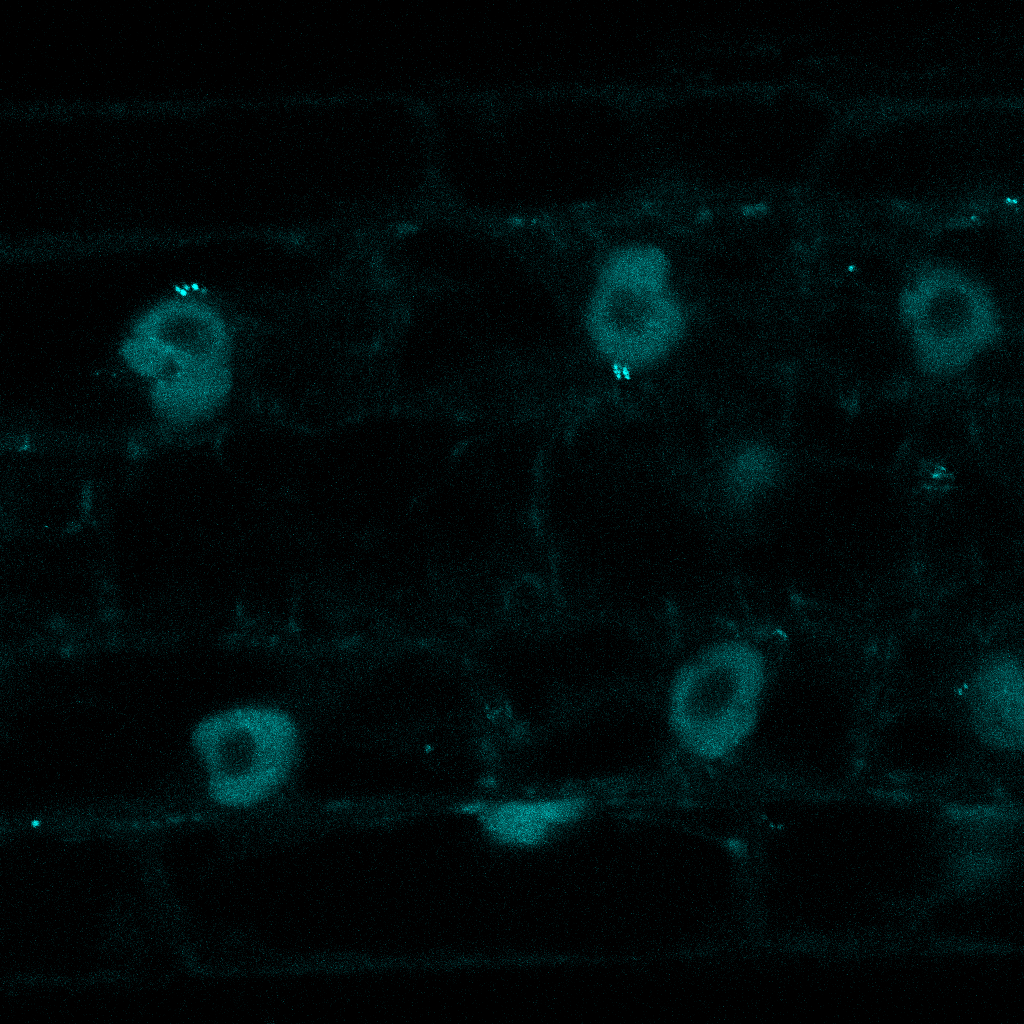

Supplement: Supplementary file 5 — Source data Fig. 5 [file 44319_2024_142_MOESM5_ESM.zip › Figure 5/5A/mCherry-ATG8 ARF7-Venus Oscillation Zone NT YFP.tif]

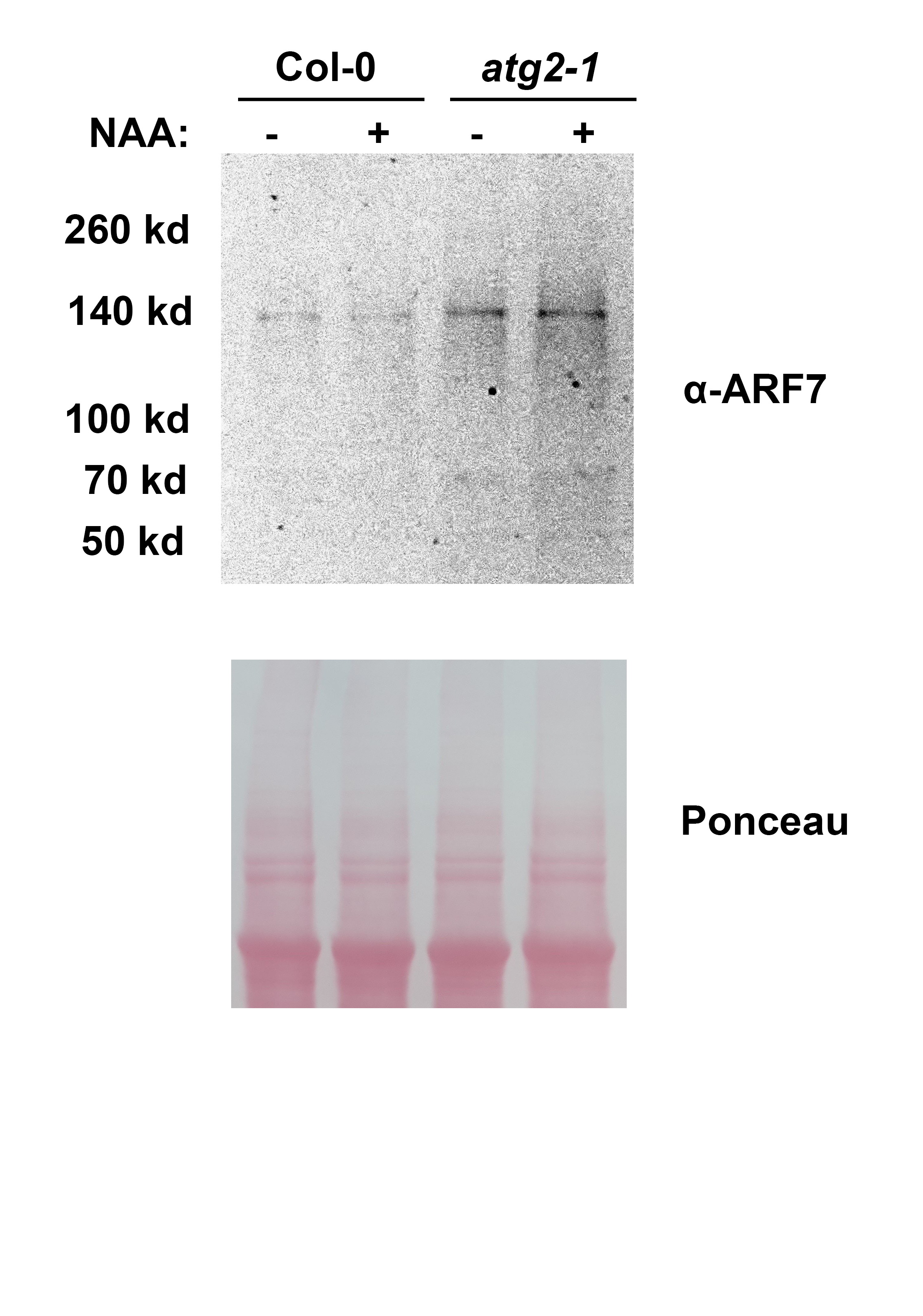

Supplement: Supplementary file 5 — Source data Fig. 5 [file 44319_2024_142_MOESM5_ESM.zip › Figure 5/5C/replicate/Western blot ARF7 NAA treatment replicate 1.tif]

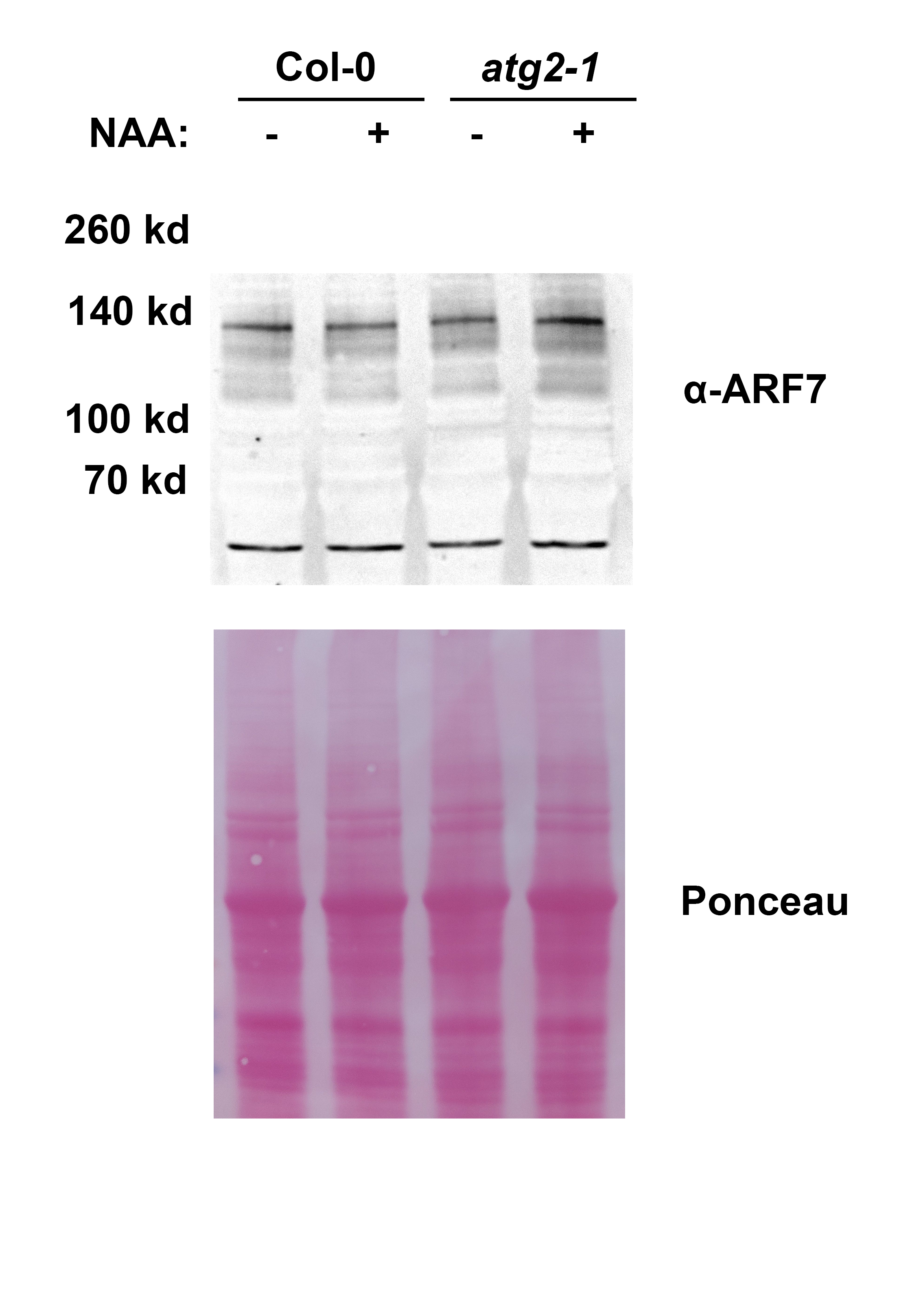

Supplement: Supplementary file 5 — Source data Fig. 5 [file 44319_2024_142_MOESM5_ESM.zip › Figure 5/5C/replicate/Western blot ARF7 NAA treatment replicate 2.tif]

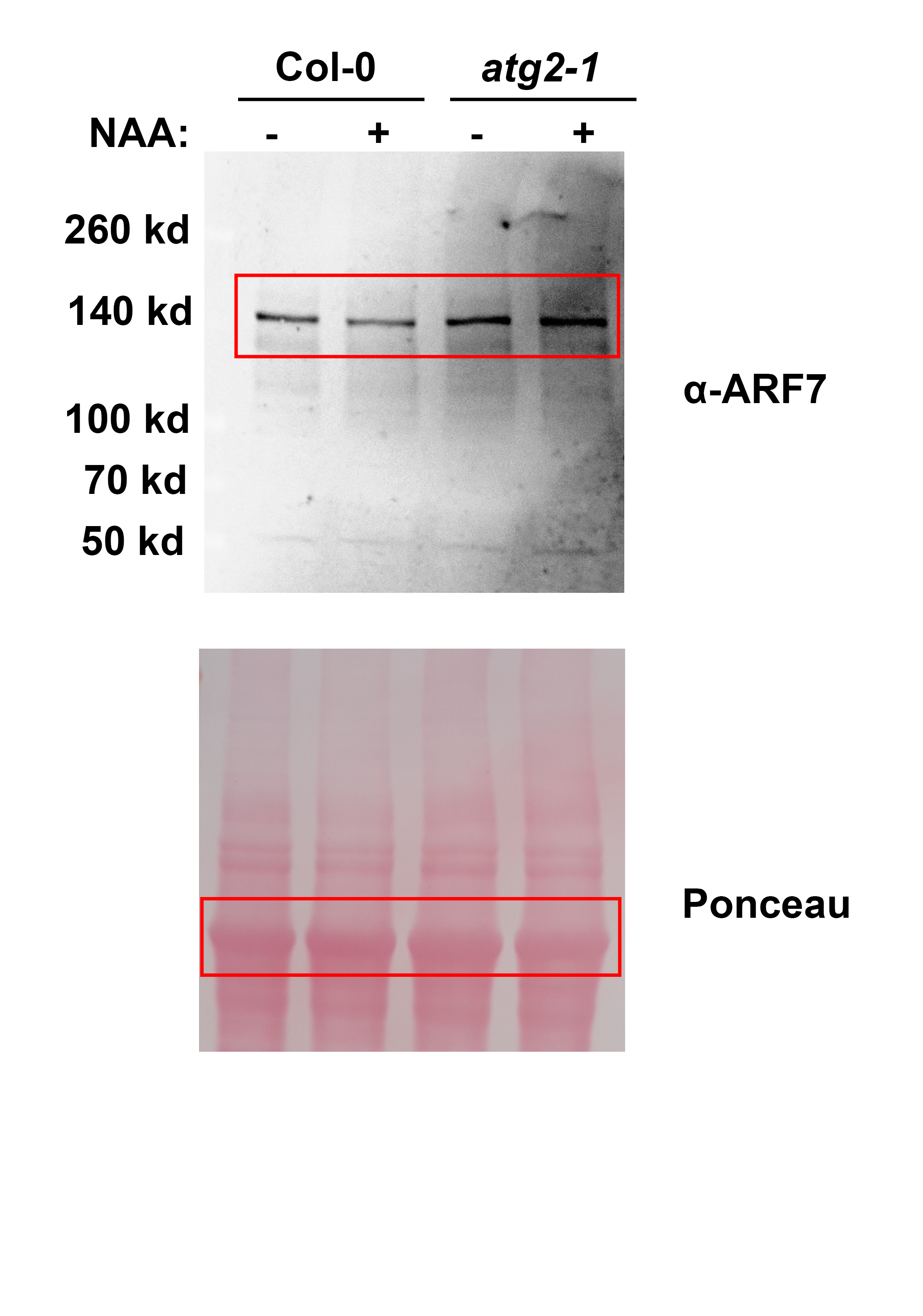

Supplement: Supplementary file 5 — Source data Fig. 5 [file 44319_2024_142_MOESM5_ESM.zip › Figure 5/5C/Western blot ARF7 NAA treatment.tif]

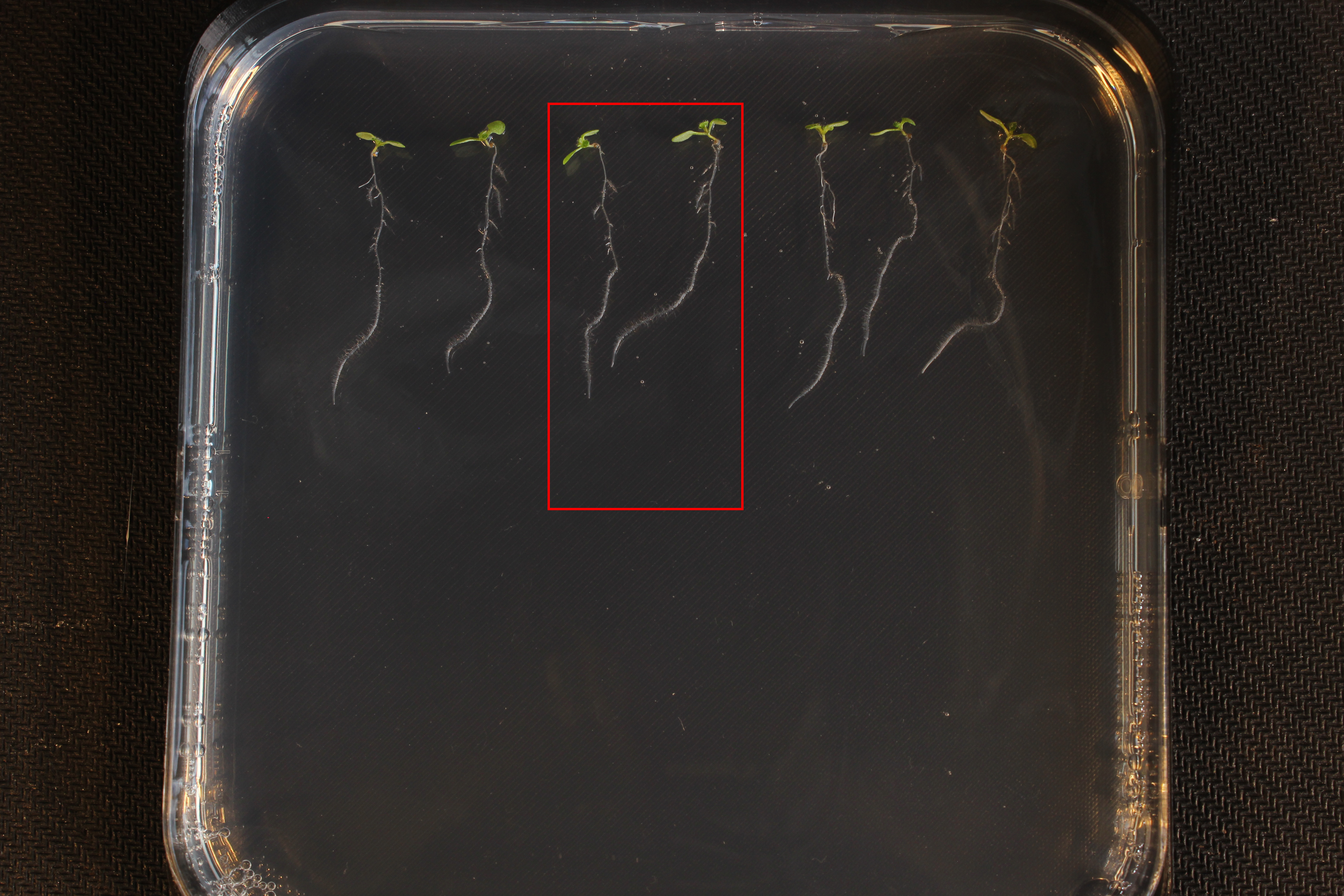

Supplement: Supplementary file 6 — Source data Fig. 6 [file 44319_2024_142_MOESM6_ESM.zip › Figure 6/6A/0.1 atg2.JPG]

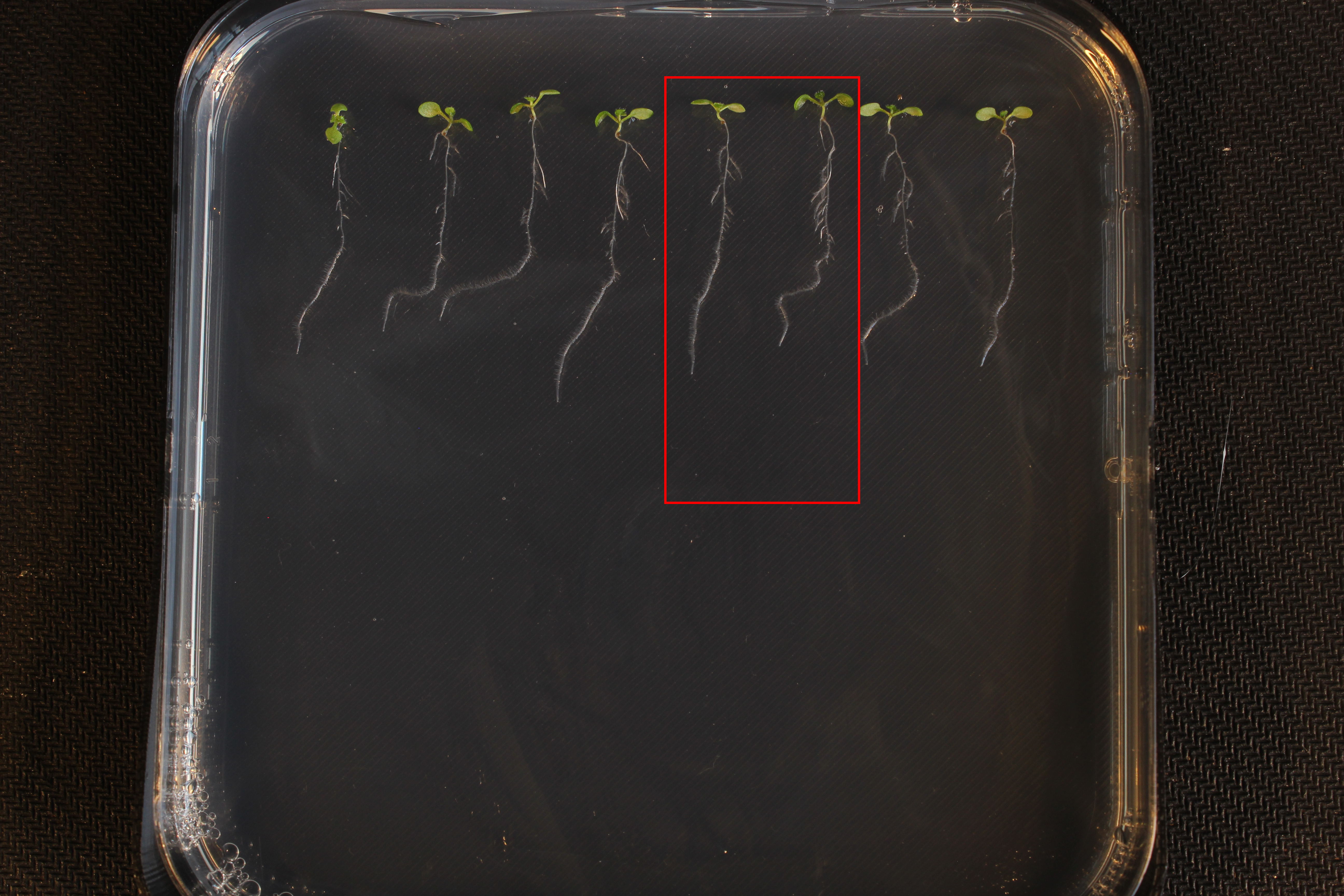

Supplement: Supplementary file 6 — Source data Fig. 6 [file 44319_2024_142_MOESM6_ESM.zip › Figure 6/6A/0.1 atg5.JPG]

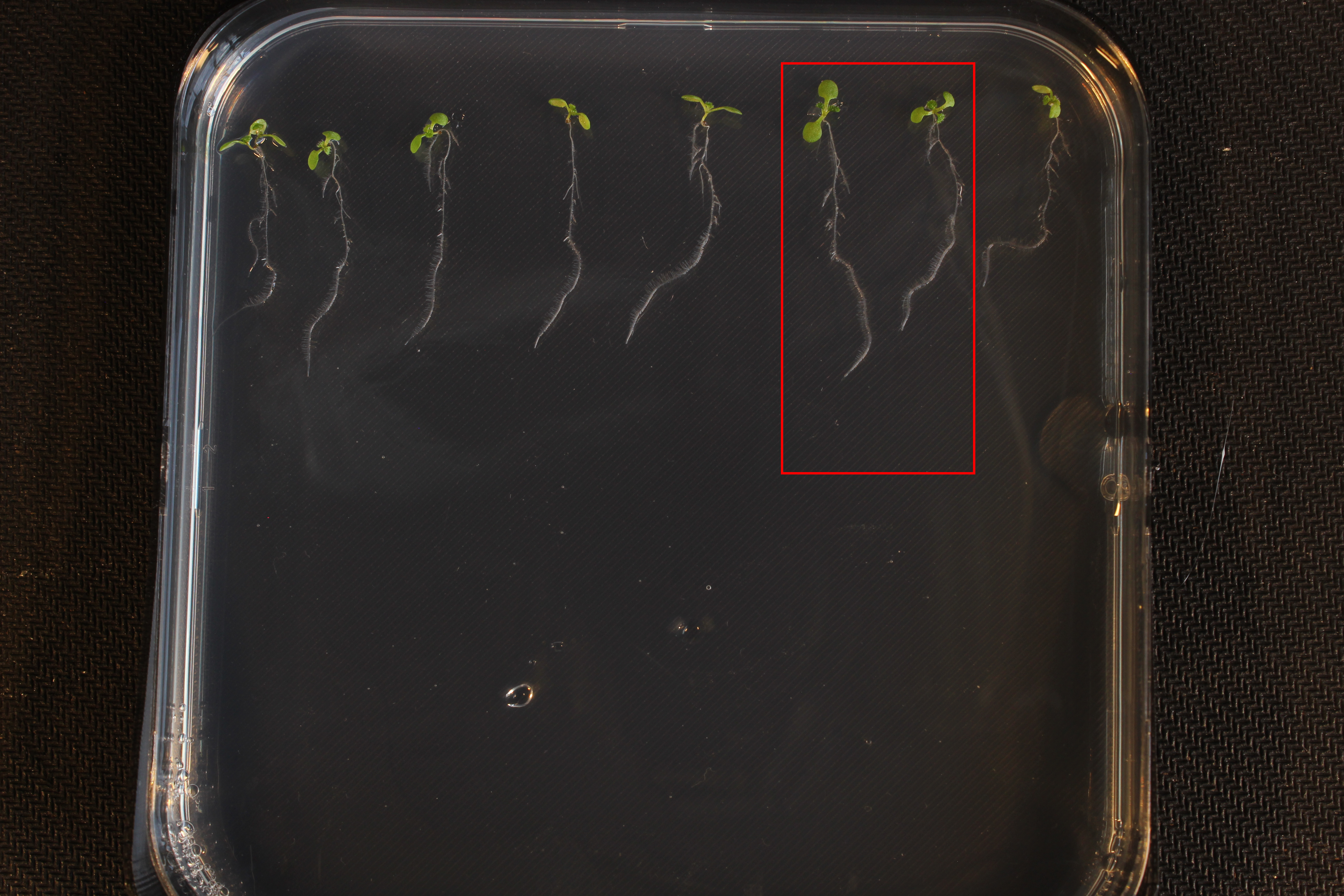

Supplement: Supplementary file 6 — Source data Fig. 6 [file 44319_2024_142_MOESM6_ESM.zip › Figure 6/6A/0.1 col.JPG]

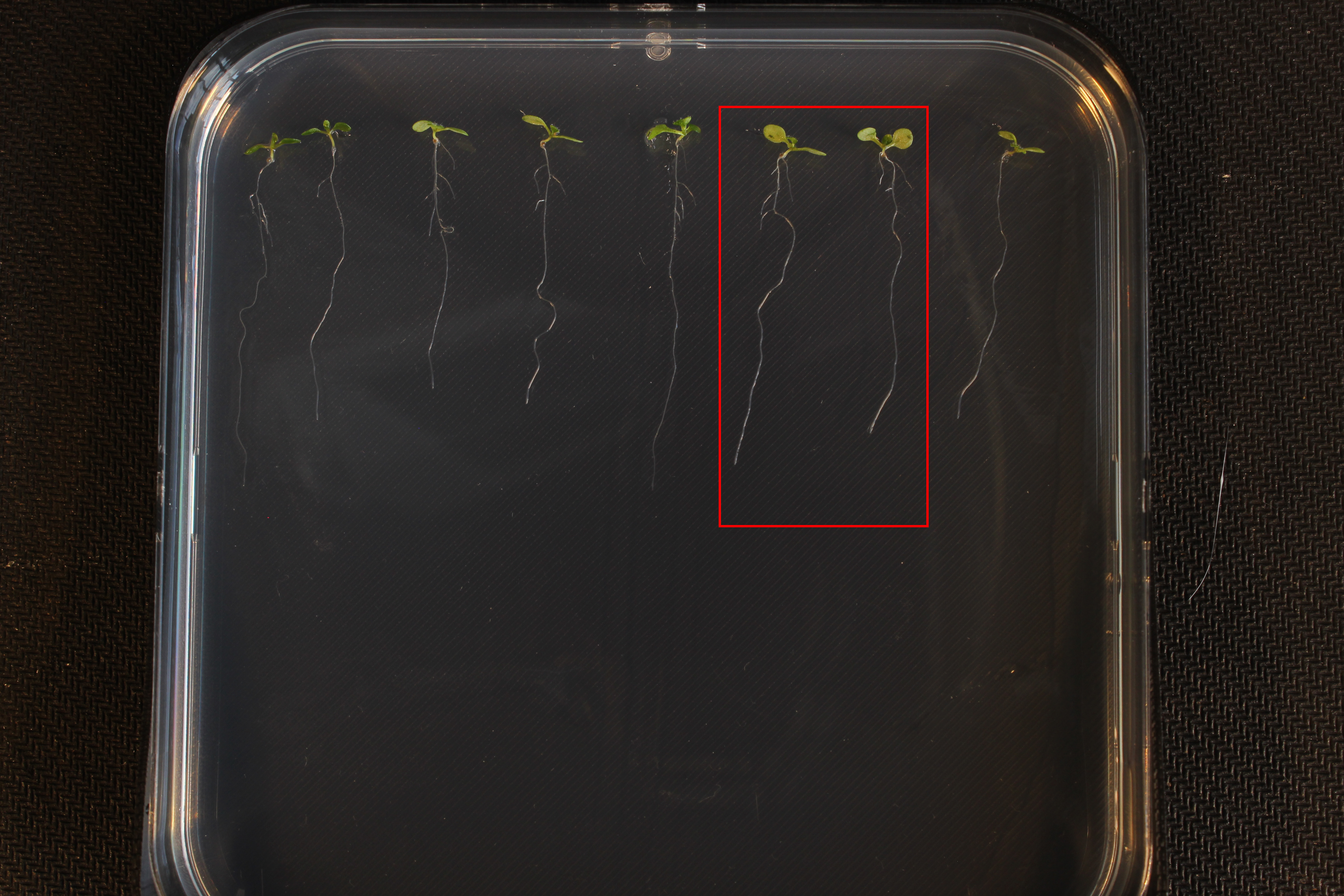

Supplement: Supplementary file 6 — Source data Fig. 6 [file 44319_2024_142_MOESM6_ESM.zip › Figure 6/6A/nt atg2.JPG]

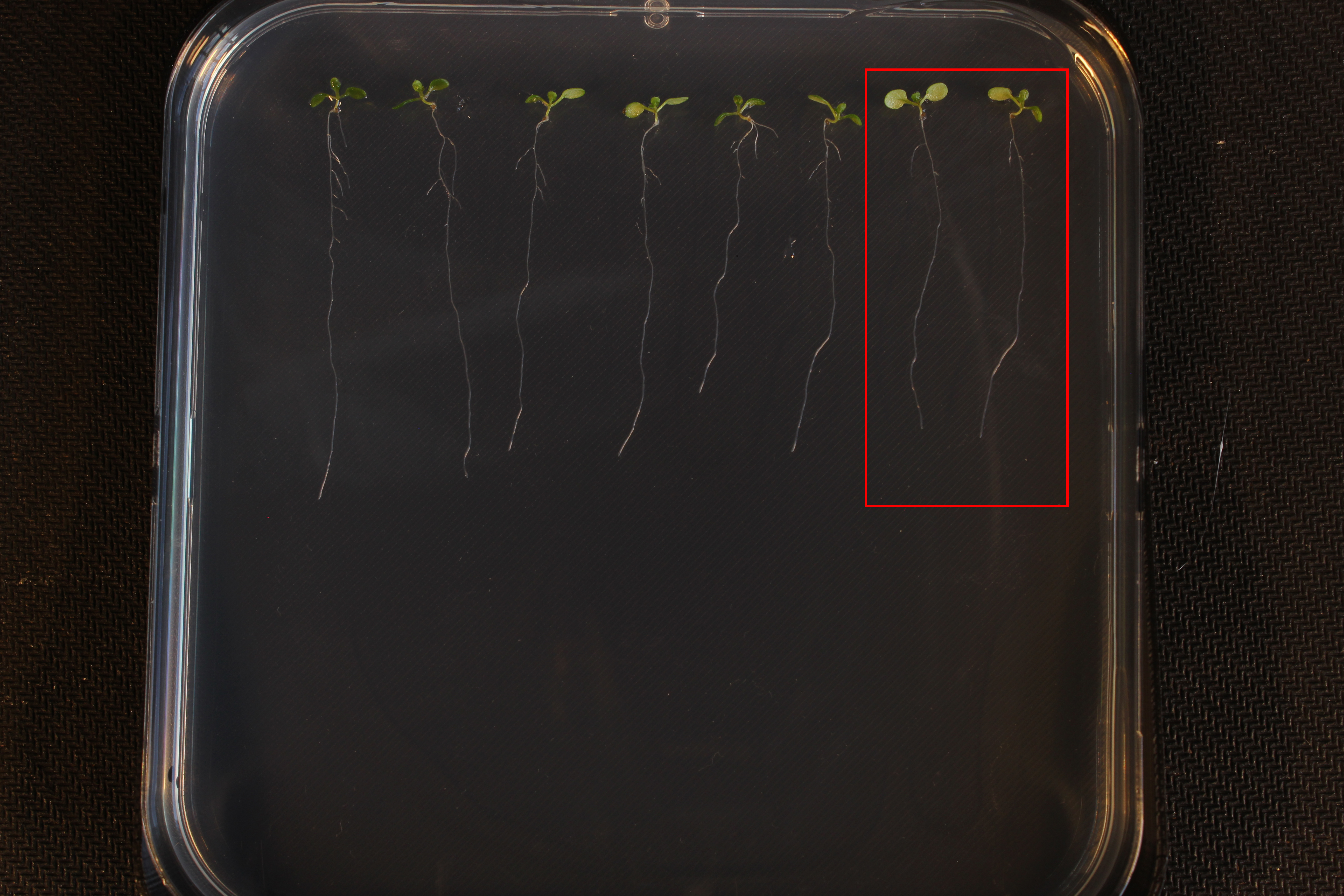

Supplement: Supplementary file 6 — Source data Fig. 6 [file 44319_2024_142_MOESM6_ESM.zip › Figure 6/6A/nt atg5.JPG]

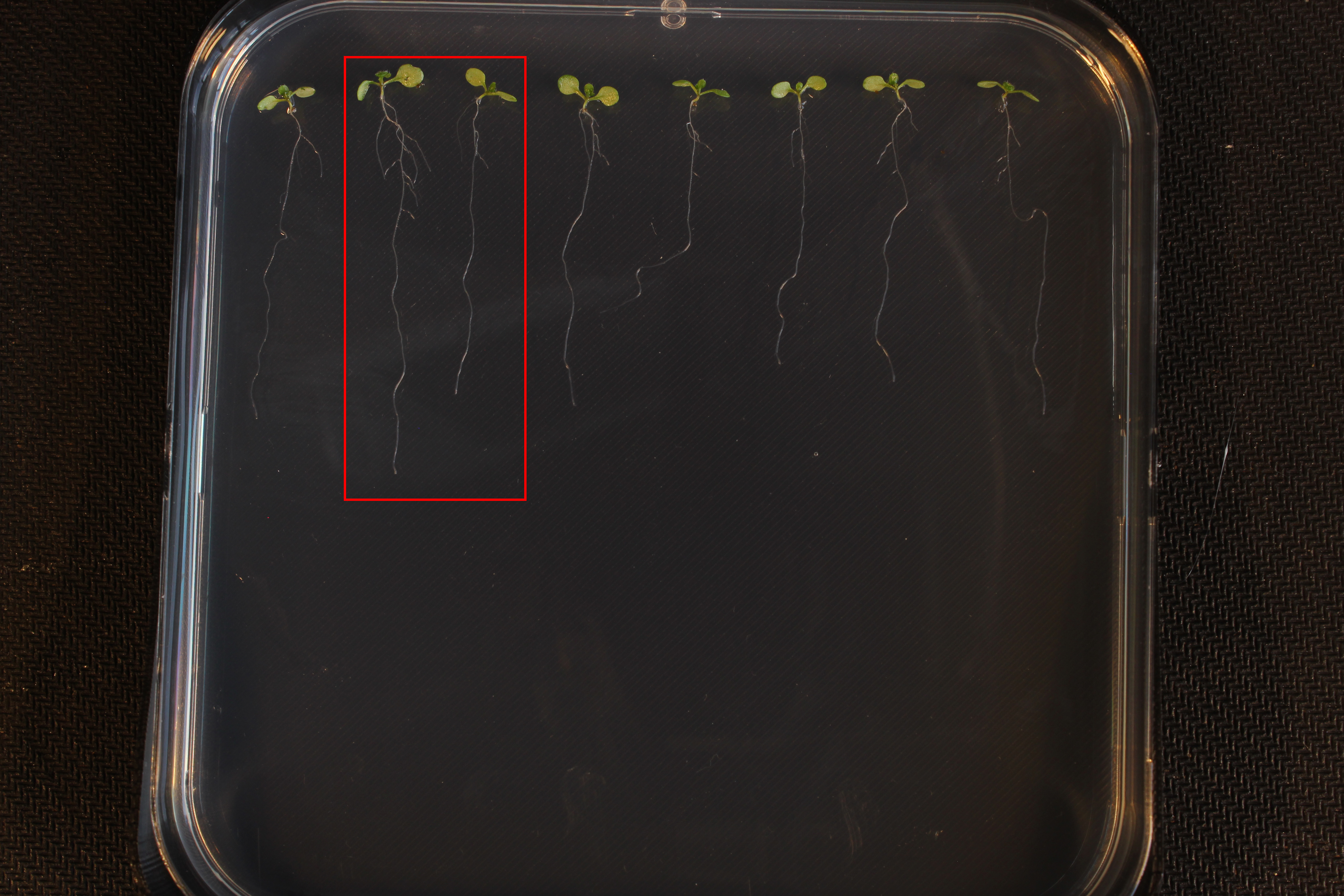

Supplement: Supplementary file 6 — Source data Fig. 6 [file 44319_2024_142_MOESM6_ESM.zip › Figure 6/6A/nt col.JPG]

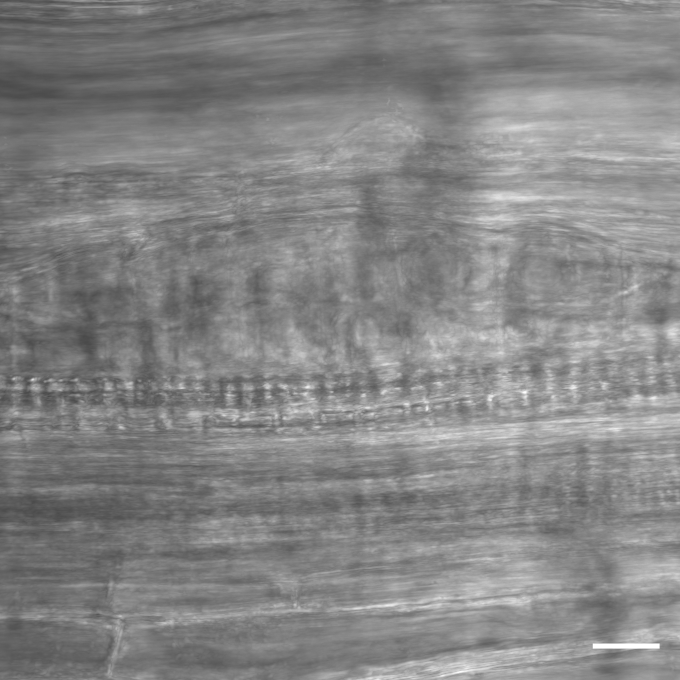

Supplement: Supplementary file 6 — Source data Fig. 6 [file 44319_2024_142_MOESM6_ESM.zip › Figure 6/6C/ARF7-Venus atg2-1 LRP Fluorescence 0 hours Bright-Field.tif]

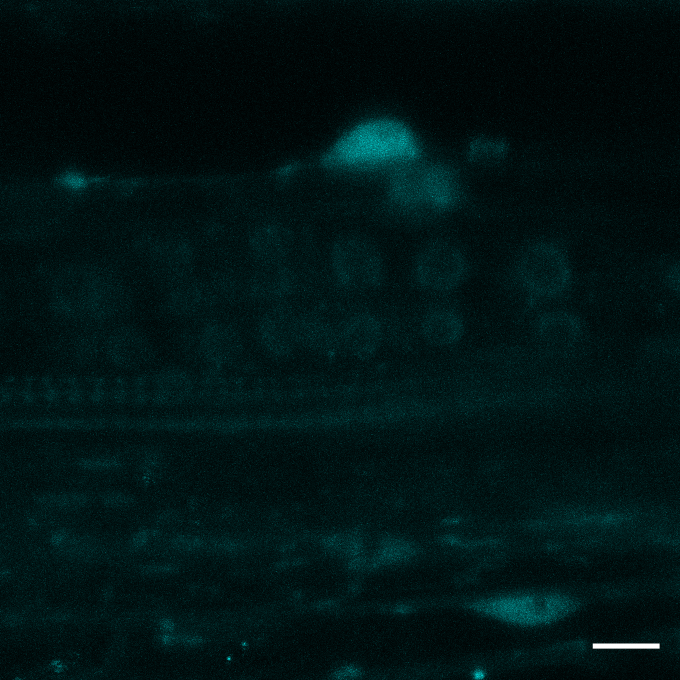

Supplement: Supplementary file 6 — Source data Fig. 6 [file 44319_2024_142_MOESM6_ESM.zip › Figure 6/6C/ARF7-Venus atg2-1 LRP Fluorescence 0 hours Venus.tif]

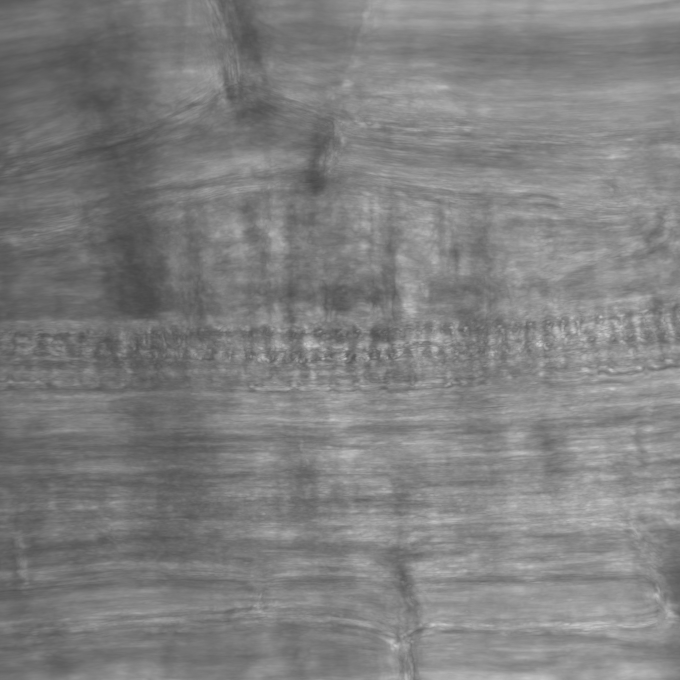

Supplement: Supplementary file 6 — Source data Fig. 6 [file 44319_2024_142_MOESM6_ESM.zip › Figure 6/6C/ARF7-Venus atg2-1 LRP Fluorescence 6 hours Bright-Field.tif]

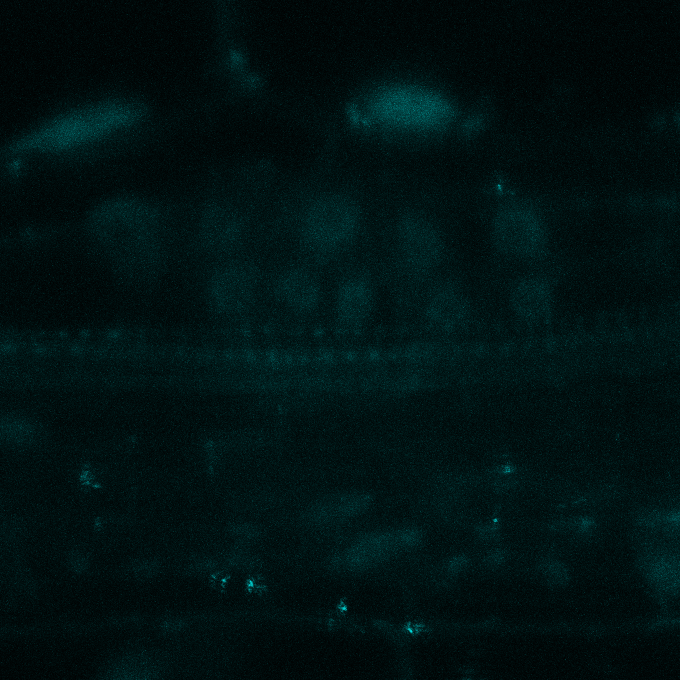

Supplement: Supplementary file 6 — Source data Fig. 6 [file 44319_2024_142_MOESM6_ESM.zip › Figure 6/6C/ARF7-Venus atg2-1 LRP Fluorescence 6 hours Venus.tif]

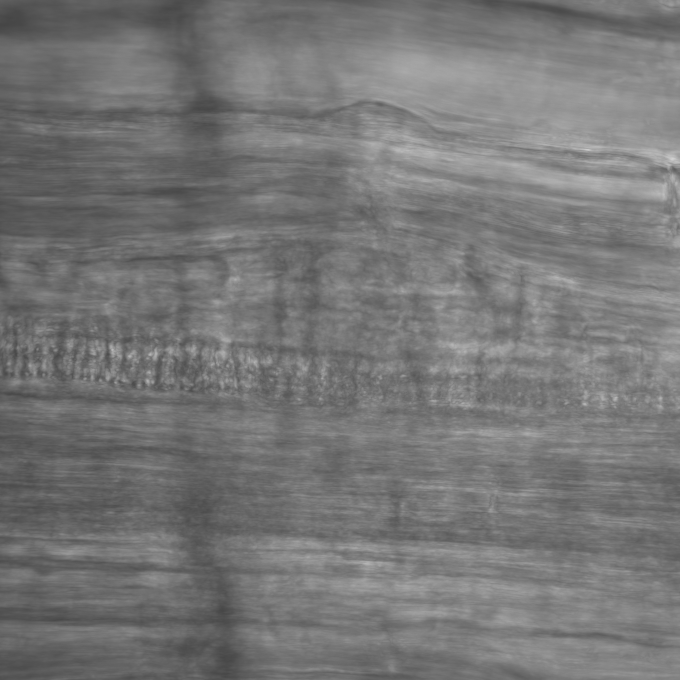

Supplement: Supplementary file 6 — Source data Fig. 6 [file 44319_2024_142_MOESM6_ESM.zip › Figure 6/6C/ARF7-Venus LRP Fluorescence 0 hours Bright-Field.tif]

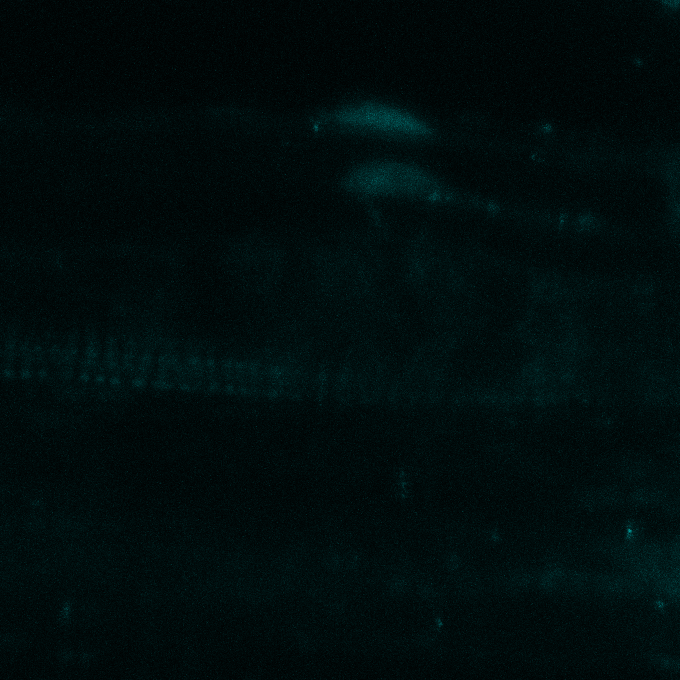

Supplement: Supplementary file 6 — Source data Fig. 6 [file 44319_2024_142_MOESM6_ESM.zip › Figure 6/6C/ARF7-Venus LRP Fluorescence 0 hours Venus.tif]

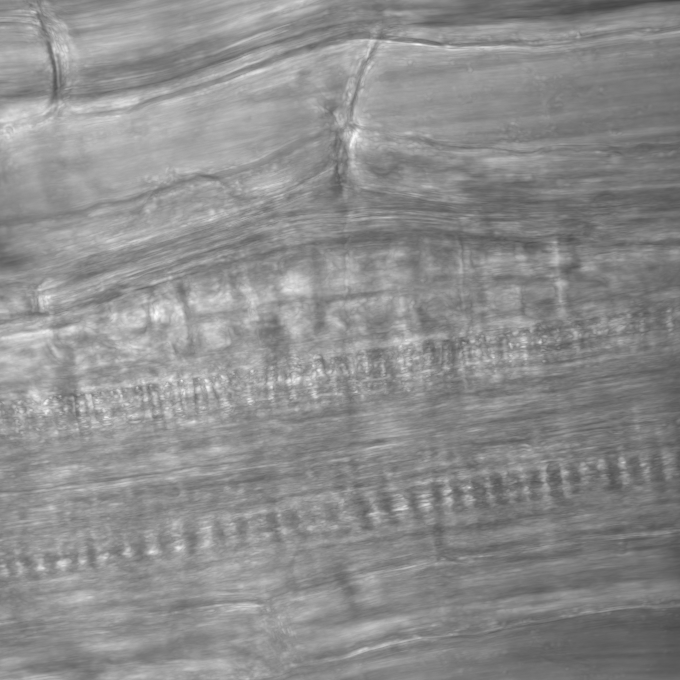

Supplement: Supplementary file 6 — Source data Fig. 6 [file 44319_2024_142_MOESM6_ESM.zip › Figure 6/6C/ARF7-Venus LRP Fluorescence 6 hours Bright-Field.tif]

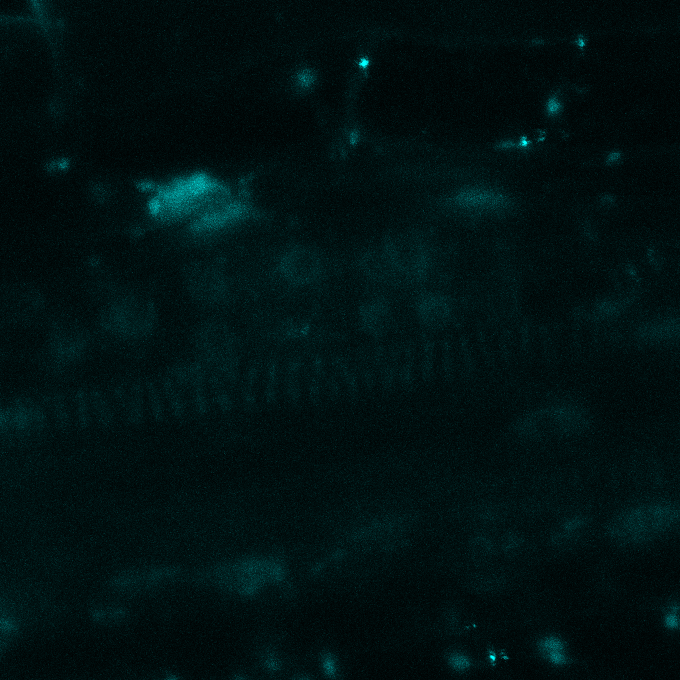

Supplement: Supplementary file 6 — Source data Fig. 6 [file 44319_2024_142_MOESM6_ESM.zip › Figure 6/6C/ARF7-Venus LRP Fluorescence 6 hours Venus.tif]
