# Supplementary figures and images for: NBR1-mediated selective autophagy of ARF7 modulates root branching (part 2 of 2)
Source: EMBO Rep. 2024 Apr 29;25(6):8. doi: 10.1038/s44319-024-00142-5 (PMC11169494; doi:10.1038/s44319-024-00142-5)

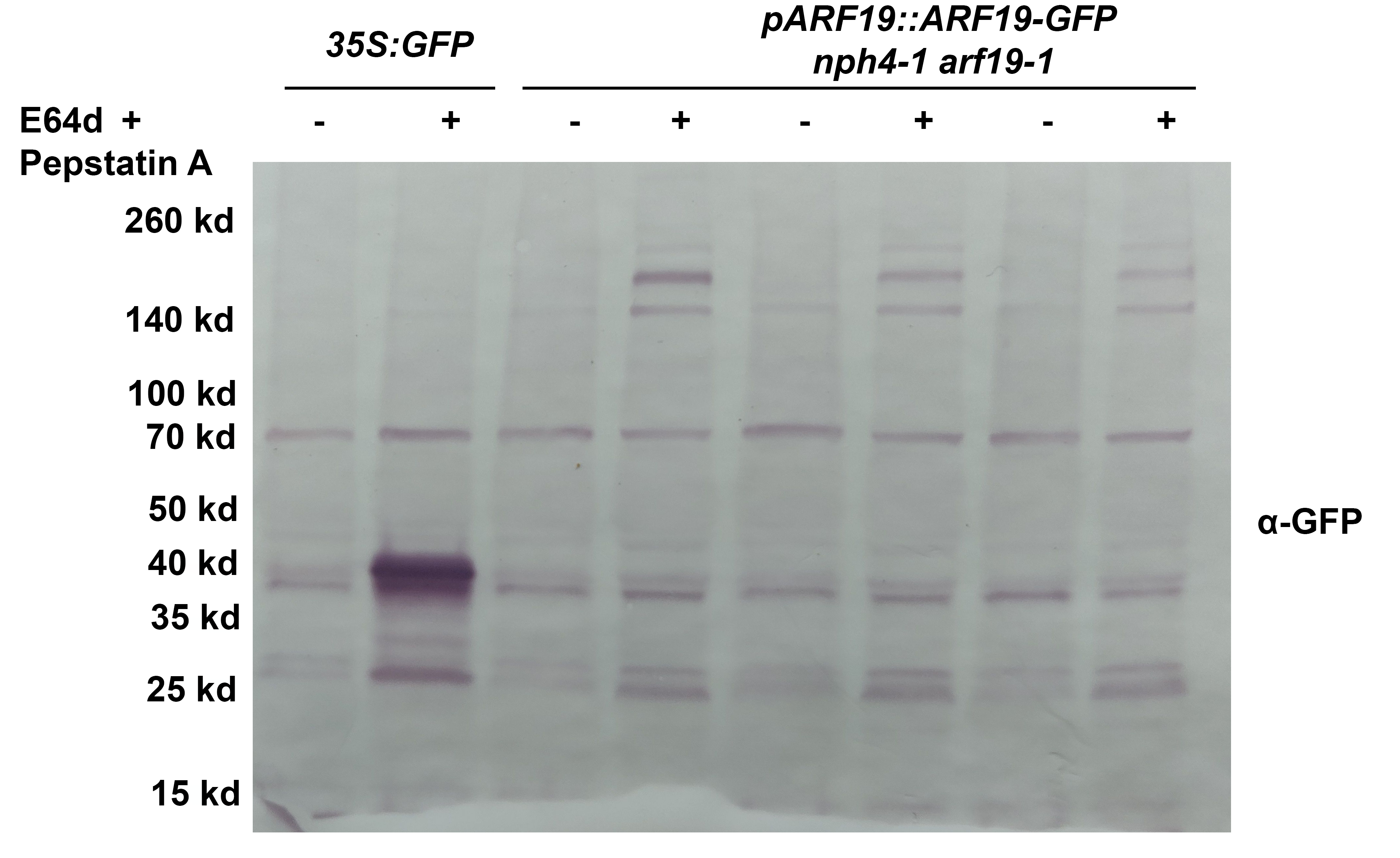

Supplement: Supplementary file 7 — Figure EV1-EV5 Source Data [file 44319_2024_142_MOESM7_ESM.zip › Expanded view figure 1/Western blot.tif]

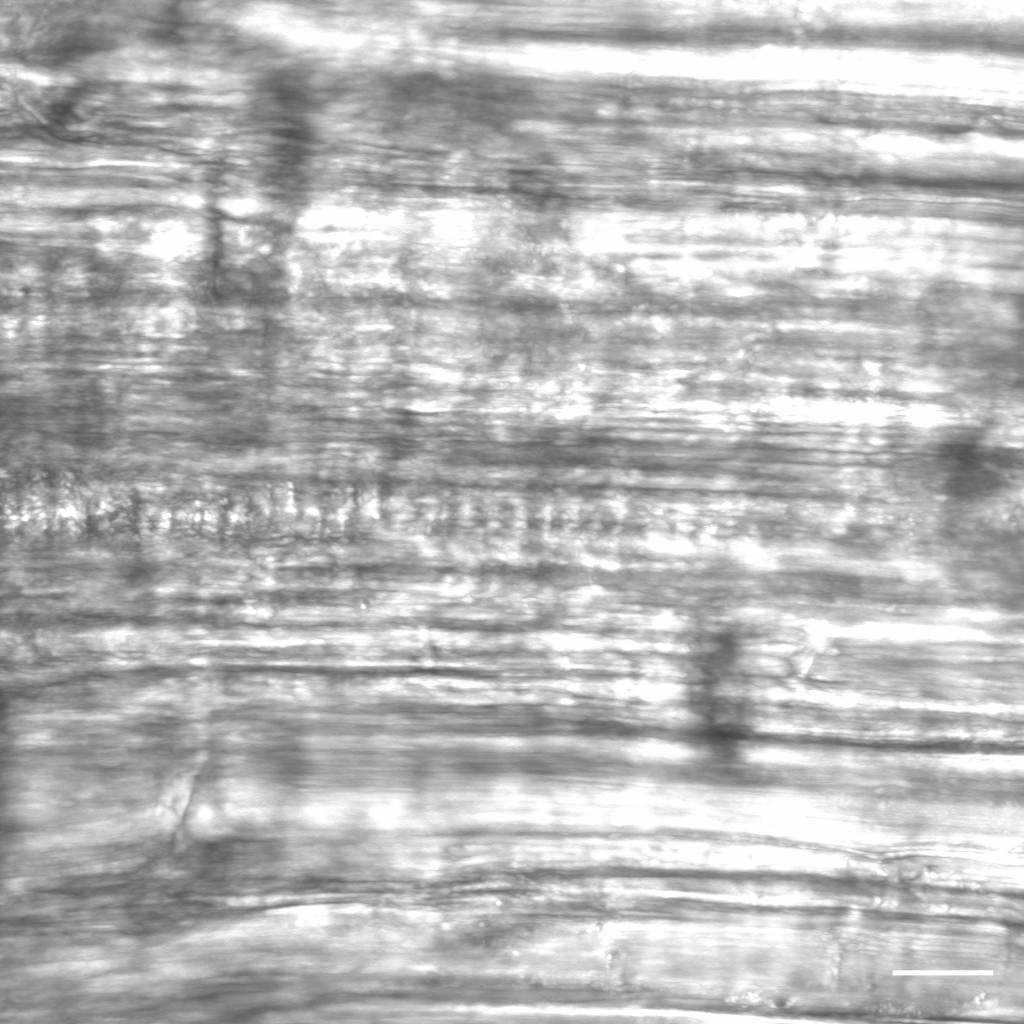

Supplement: Supplementary file 7 — Figure EV1-EV5 Source Data [file 44319_2024_142_MOESM7_ESM.zip › Expanded view figure 2/1,6 hexanediol ARF7-Venus mCherry-ATG8 0 mins Bright-Field.tif]

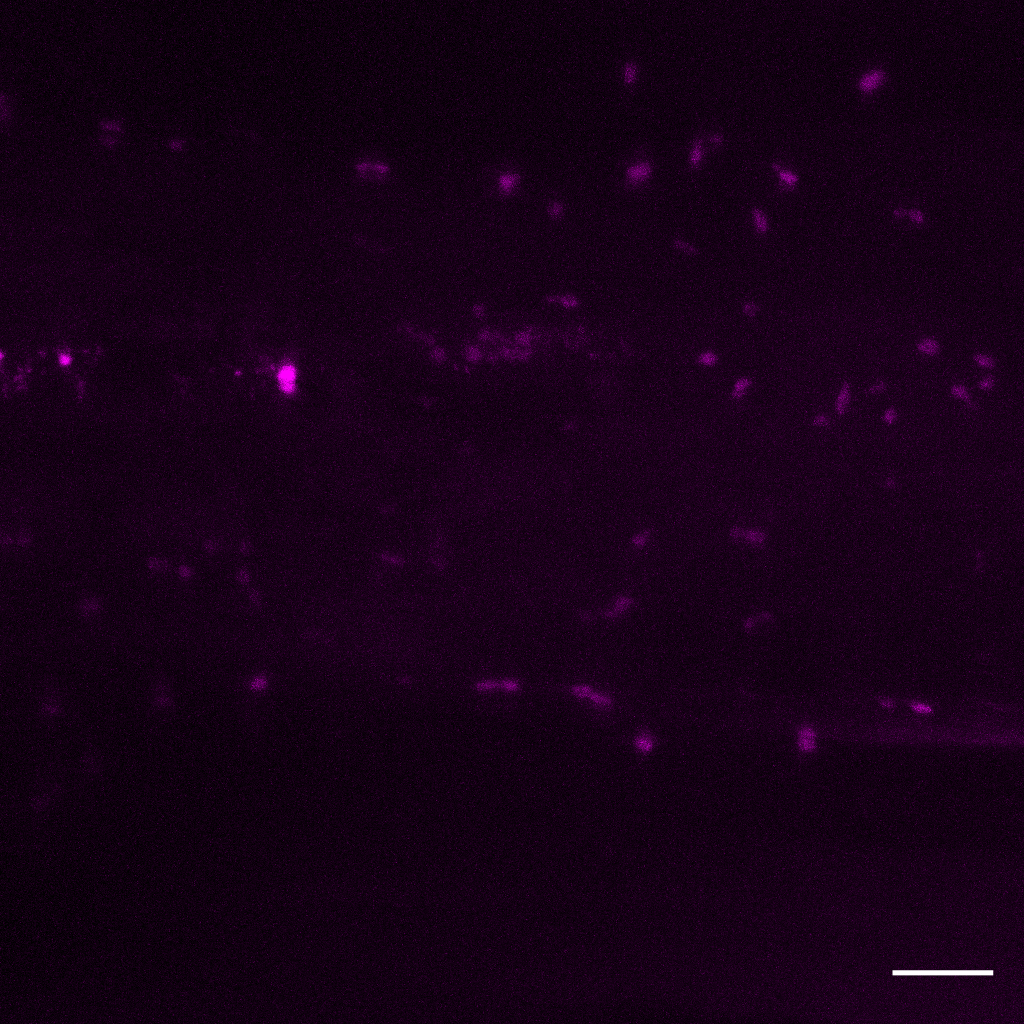

Supplement: Supplementary file 7 — Figure EV1-EV5 Source Data [file 44319_2024_142_MOESM7_ESM.zip › Expanded view figure 2/1,6 hexanediol ARF7-Venus mCherry-ATG8 0 mins mCherry.tif]

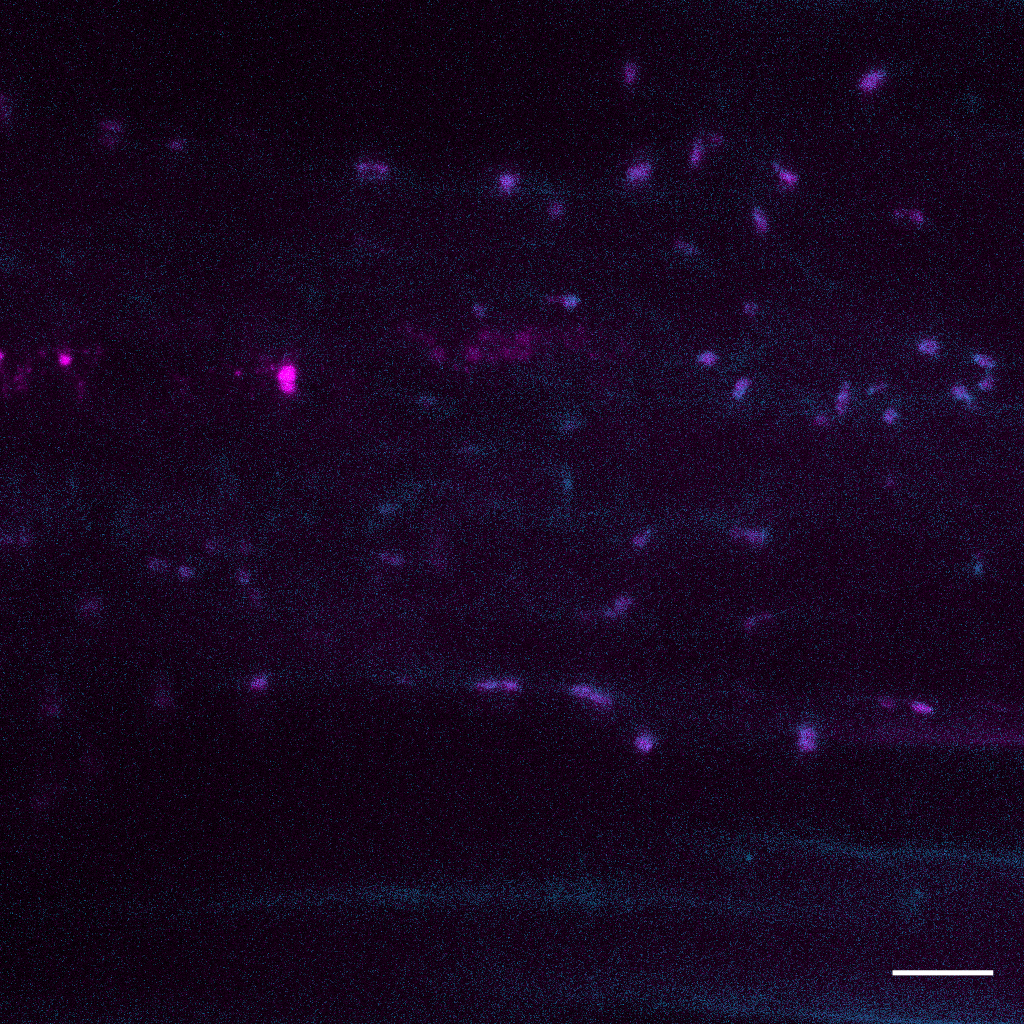

Supplement: Supplementary file 7 — Figure EV1-EV5 Source Data [file 44319_2024_142_MOESM7_ESM.zip › Expanded view figure 2/1,6 hexanediol ARF7-Venus mCherry-ATG8 0 mins merged 1.tif]

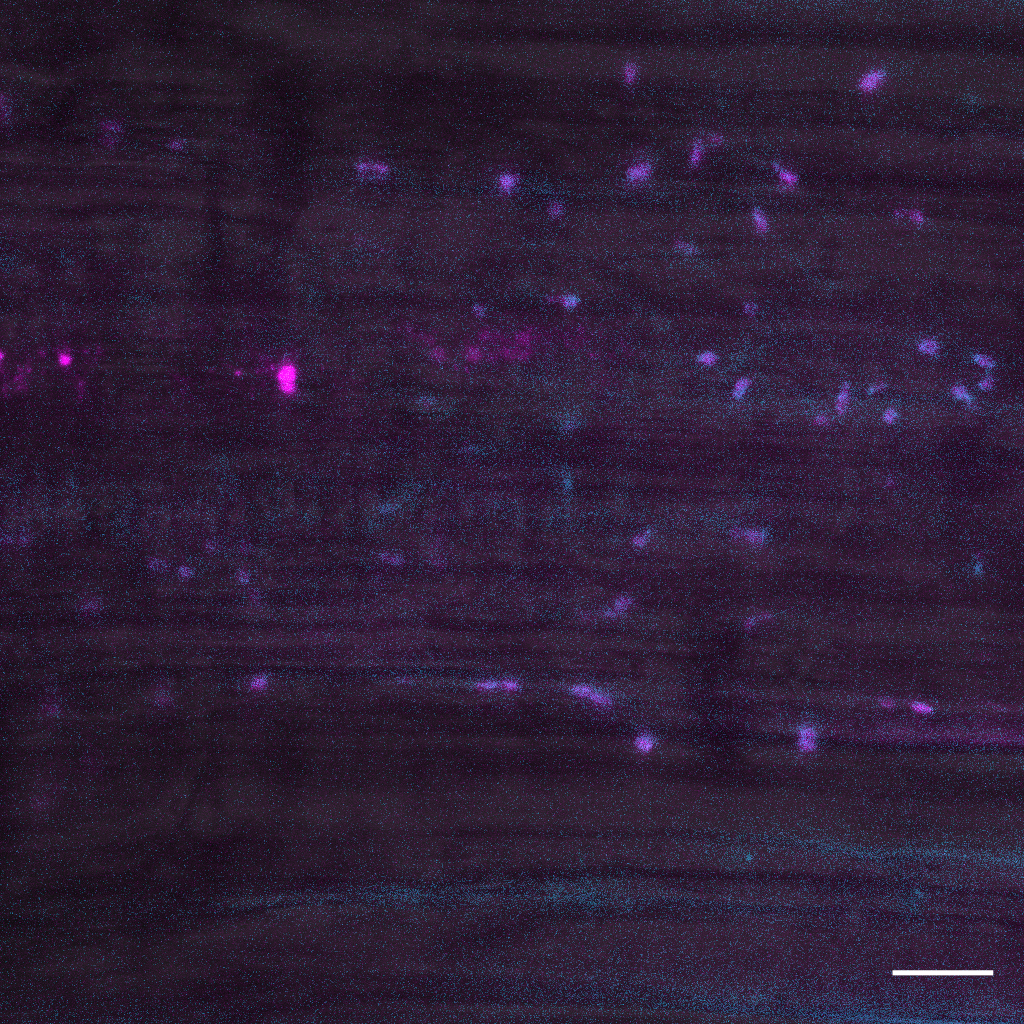

Supplement: Supplementary file 7 — Figure EV1-EV5 Source Data [file 44319_2024_142_MOESM7_ESM.zip › Expanded view figure 2/1,6 hexanediol ARF7-Venus mCherry-ATG8 0 mins merged 2.tif]

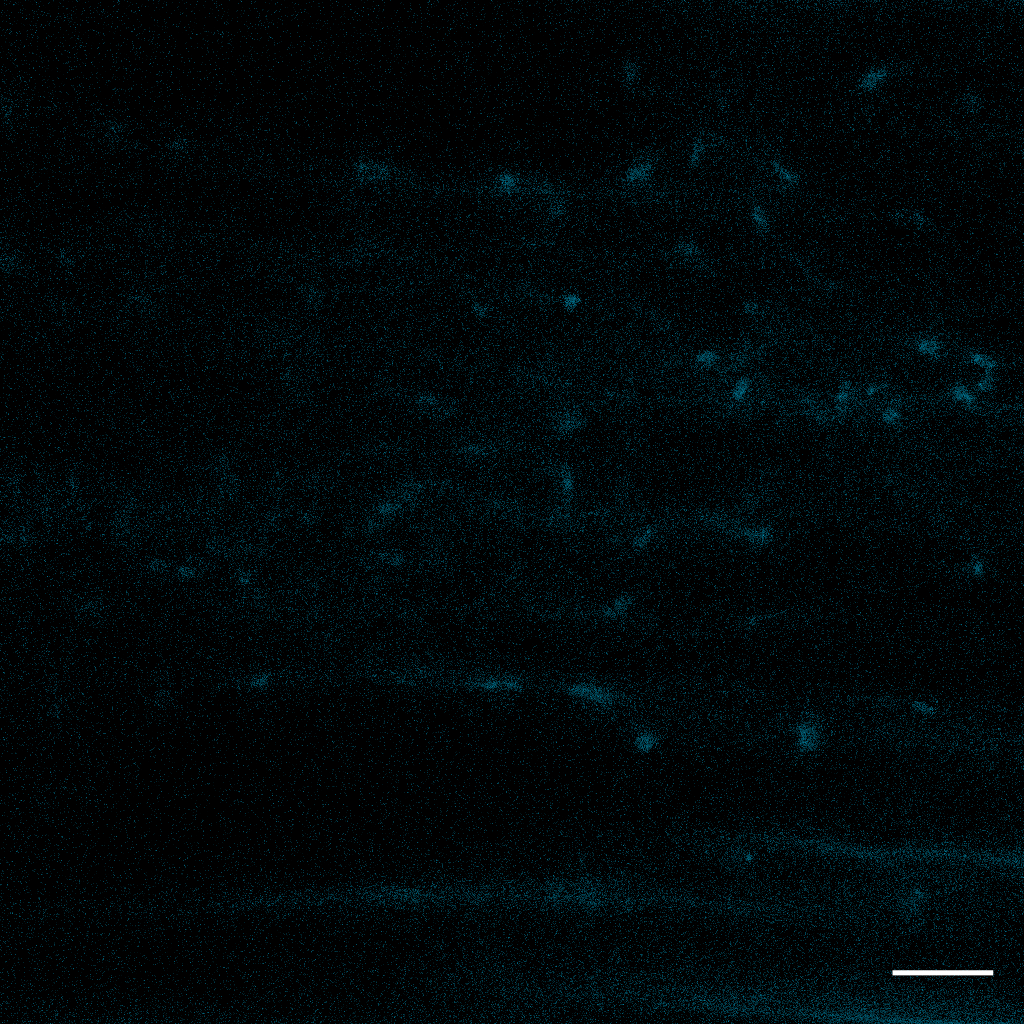

Supplement: Supplementary file 7 — Figure EV1-EV5 Source Data [file 44319_2024_142_MOESM7_ESM.zip › Expanded view figure 2/1,6 hexanediol ARF7-Venus mCherry-ATG8 0 mins YFP.tif]

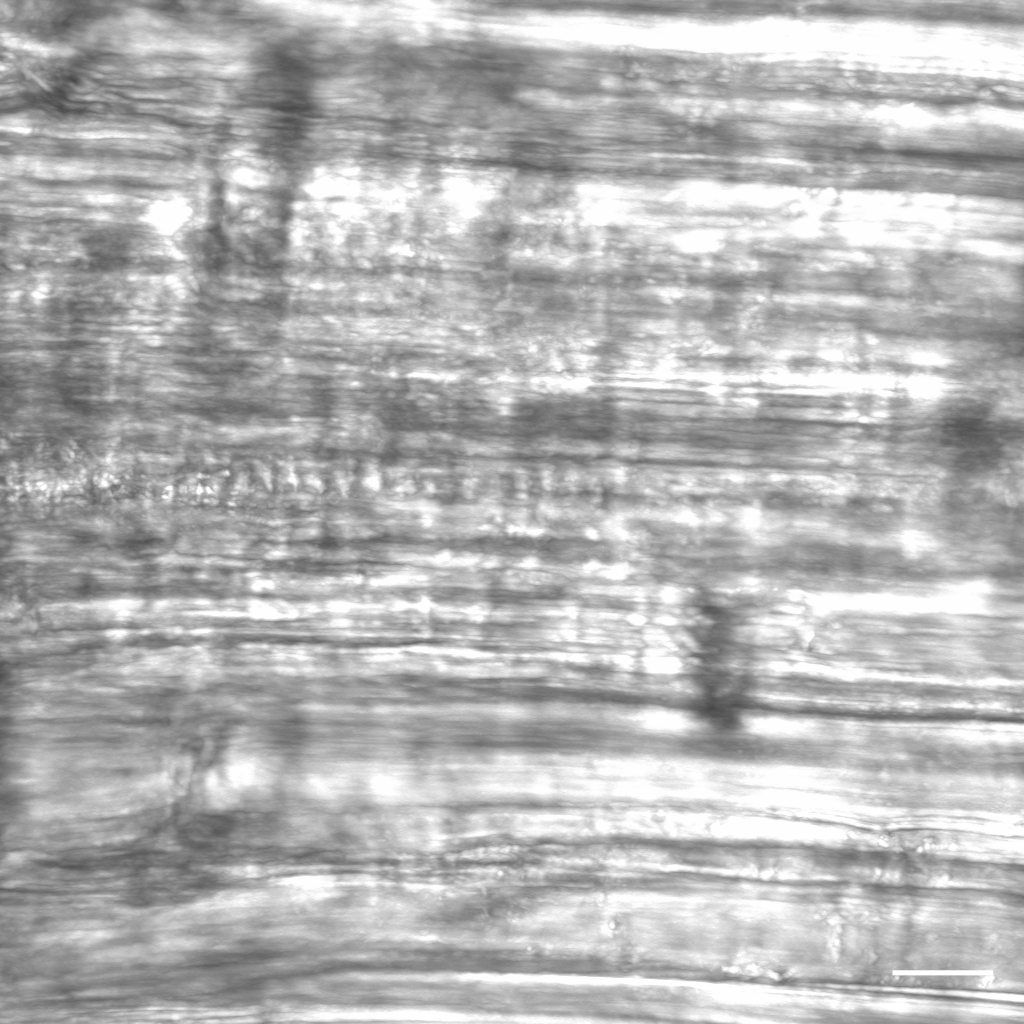

Supplement: Supplementary file 7 — Figure EV1-EV5 Source Data [file 44319_2024_142_MOESM7_ESM.zip › Expanded view figure 2/1,6 hexanediol ARF7-Venus mCherry-ATG8 10 mins Bright-Field.tif]

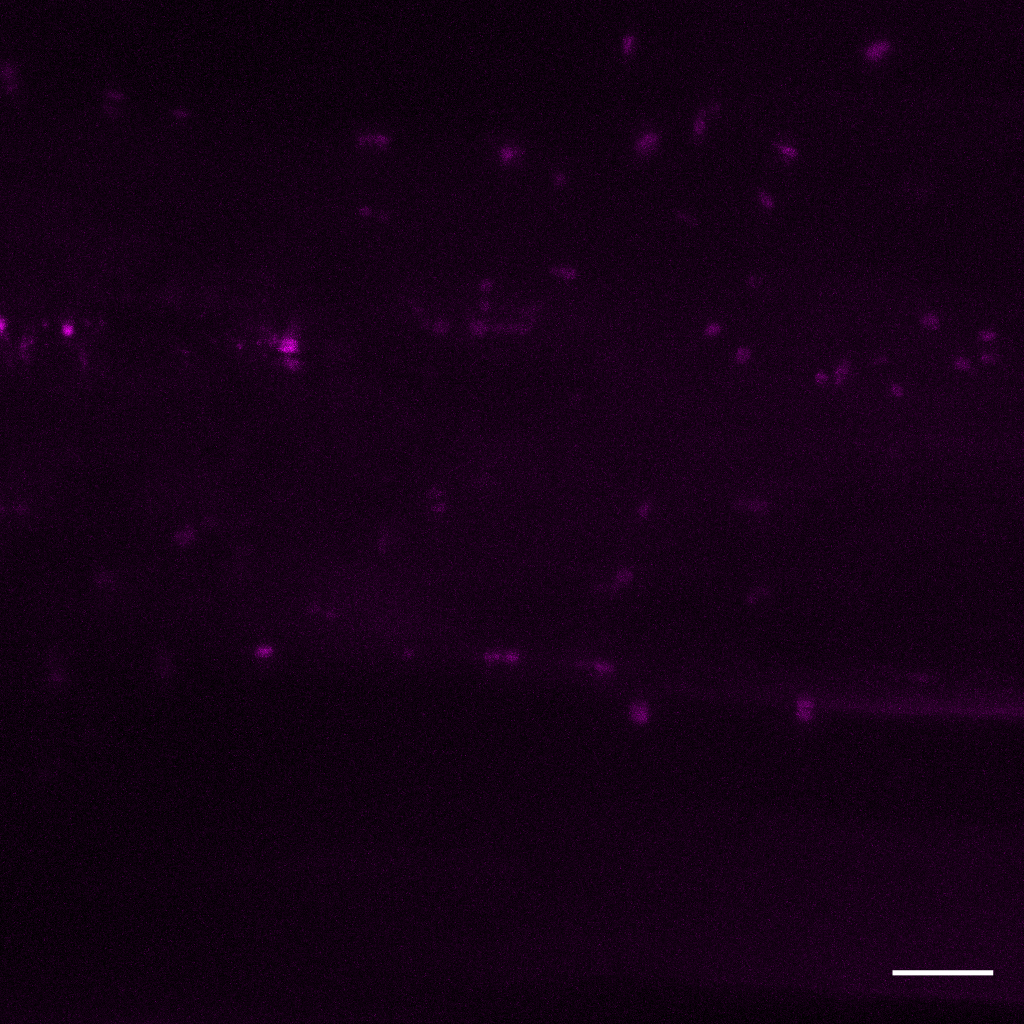

Supplement: Supplementary file 7 — Figure EV1-EV5 Source Data [file 44319_2024_142_MOESM7_ESM.zip › Expanded view figure 2/1,6 hexanediol ARF7-Venus mCherry-ATG8 10 mins mCherry.tif]

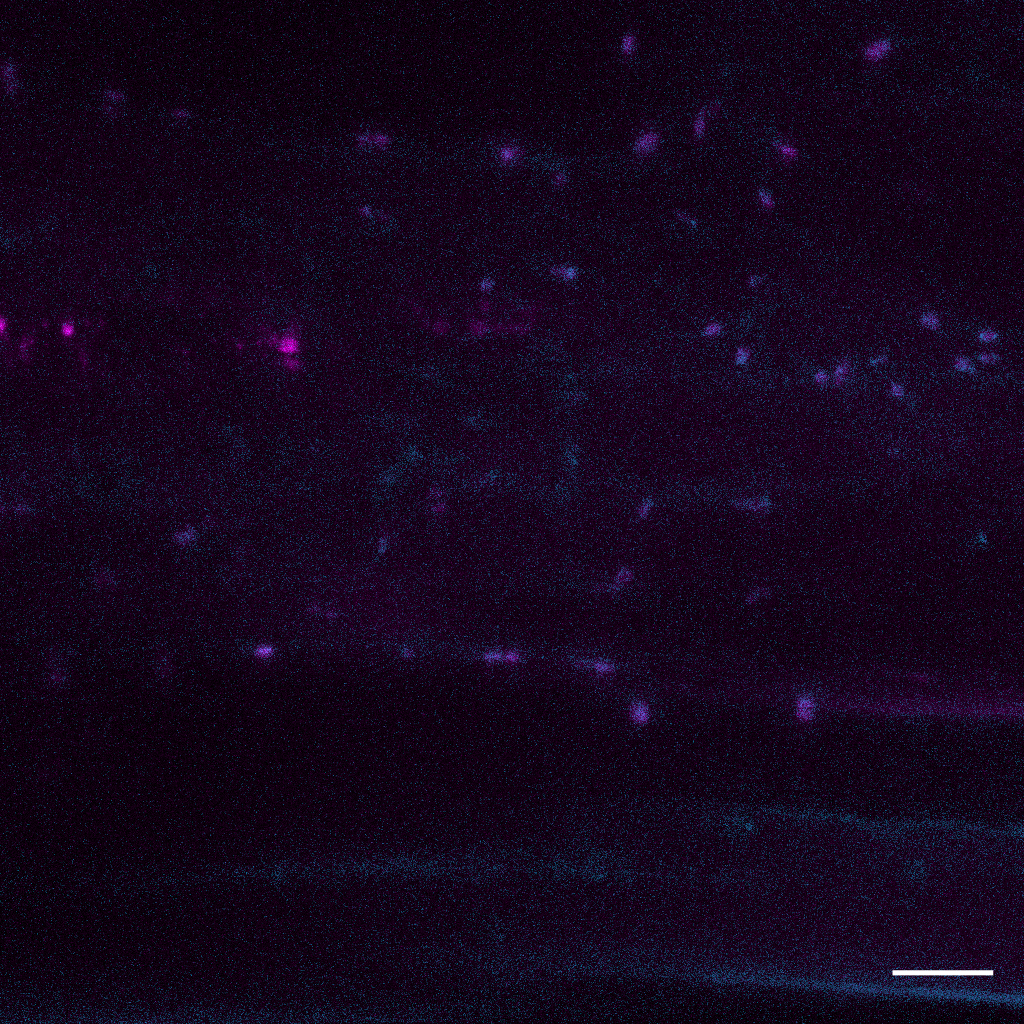

Supplement: Supplementary file 7 — Figure EV1-EV5 Source Data [file 44319_2024_142_MOESM7_ESM.zip › Expanded view figure 2/1,6 hexanediol ARF7-Venus mCherry-ATG8 10 mins merged 1.tif]

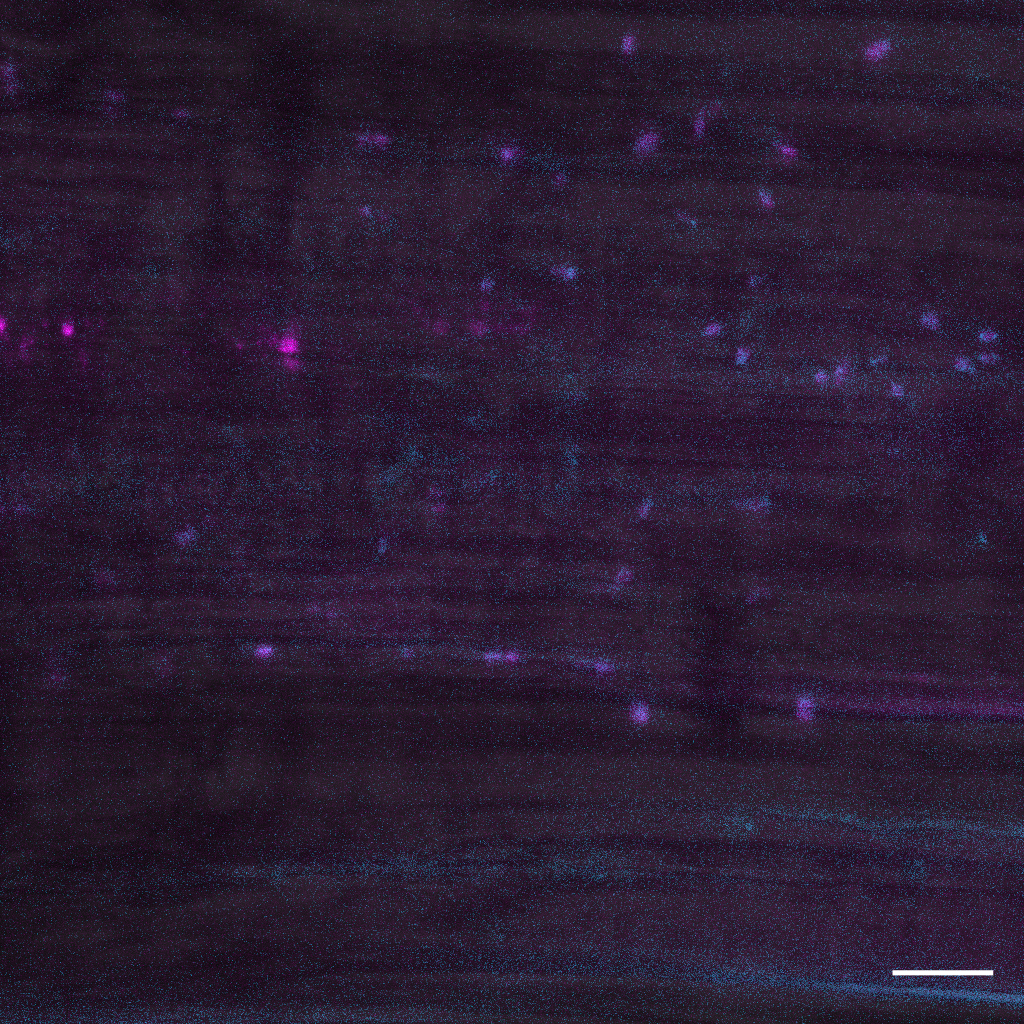

Supplement: Supplementary file 7 — Figure EV1-EV5 Source Data [file 44319_2024_142_MOESM7_ESM.zip › Expanded view figure 2/1,6 hexanediol ARF7-Venus mCherry-ATG8 10 mins merged 2.tif]

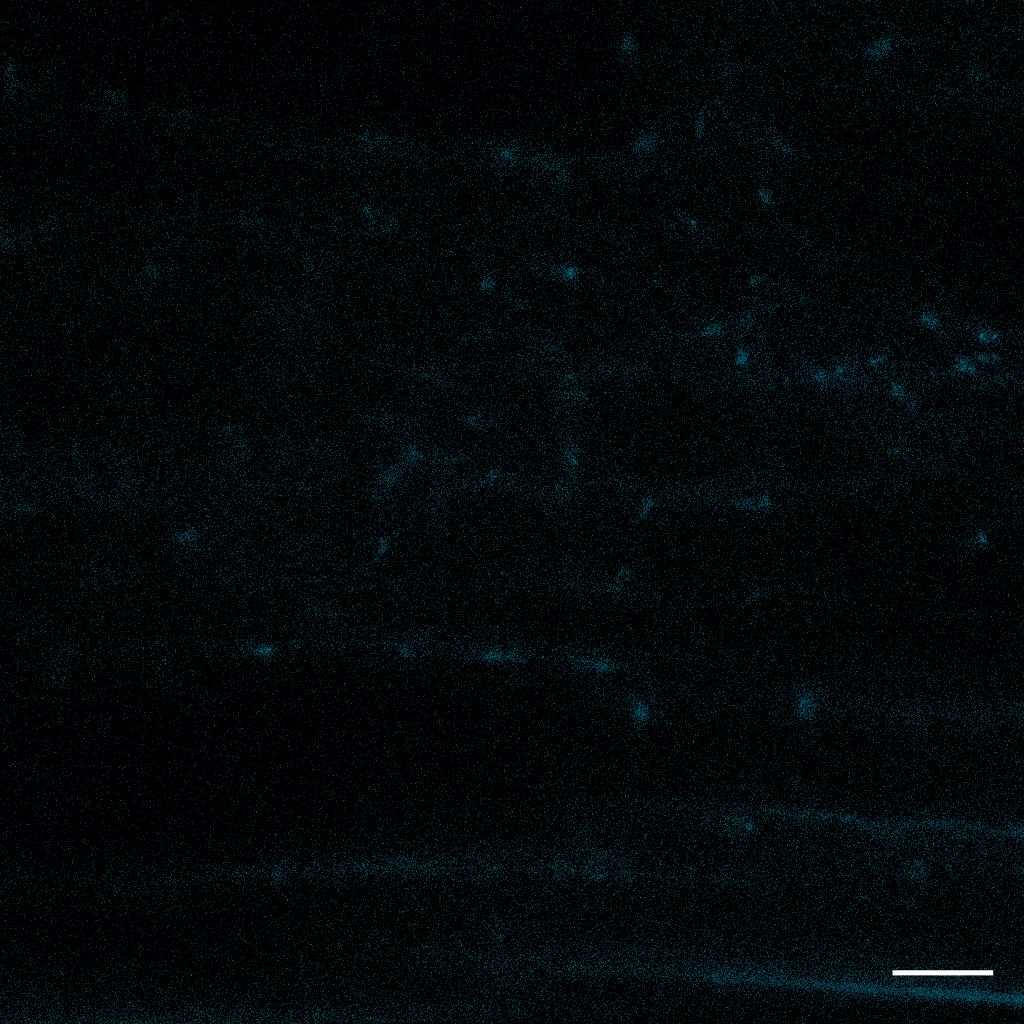

Supplement: Supplementary file 7 — Figure EV1-EV5 Source Data [file 44319_2024_142_MOESM7_ESM.zip › Expanded view figure 2/1,6 hexanediol ARF7-Venus mCherry-ATG8 10 mins YFP.tif]

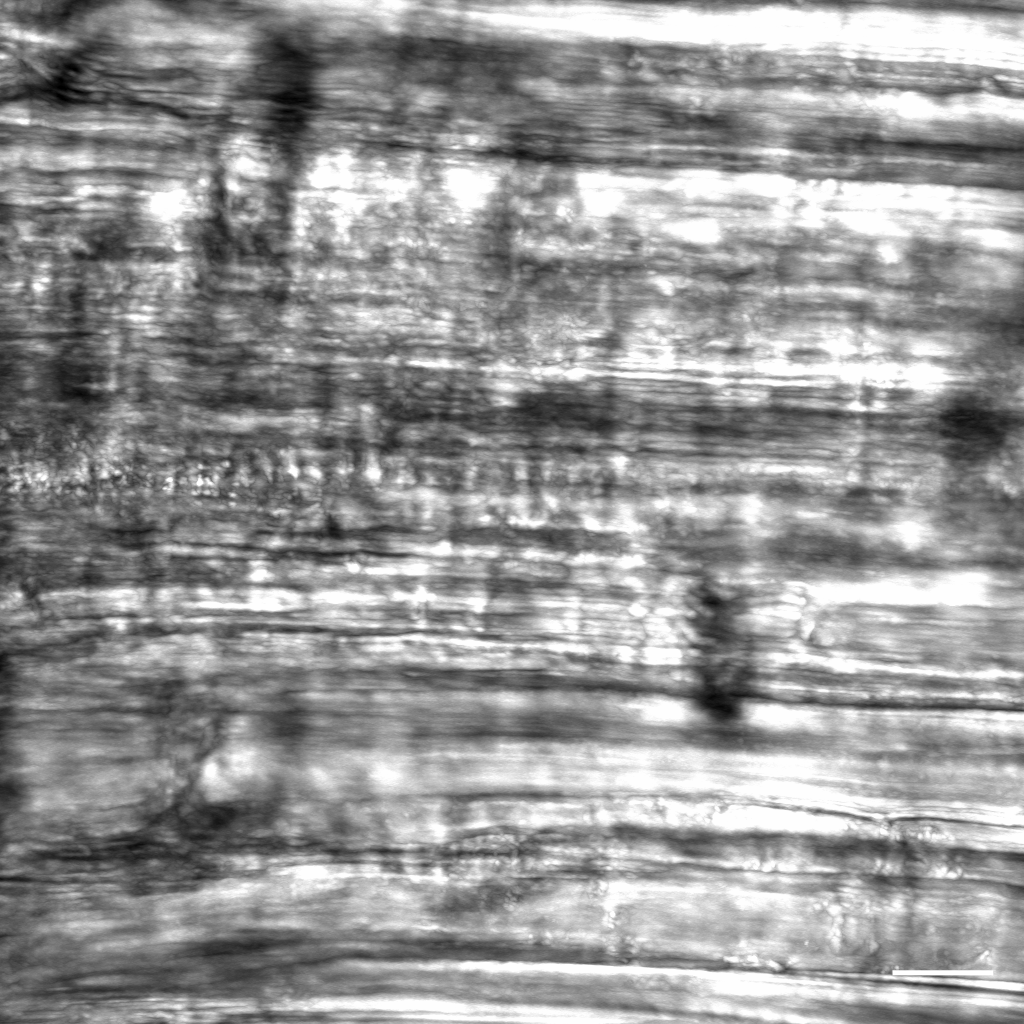

Supplement: Supplementary file 7 — Figure EV1-EV5 Source Data [file 44319_2024_142_MOESM7_ESM.zip › Expanded view figure 2/1,6 hexanediol ARF7-Venus mCherry-ATG8 20 mins Bright-Field.tif]

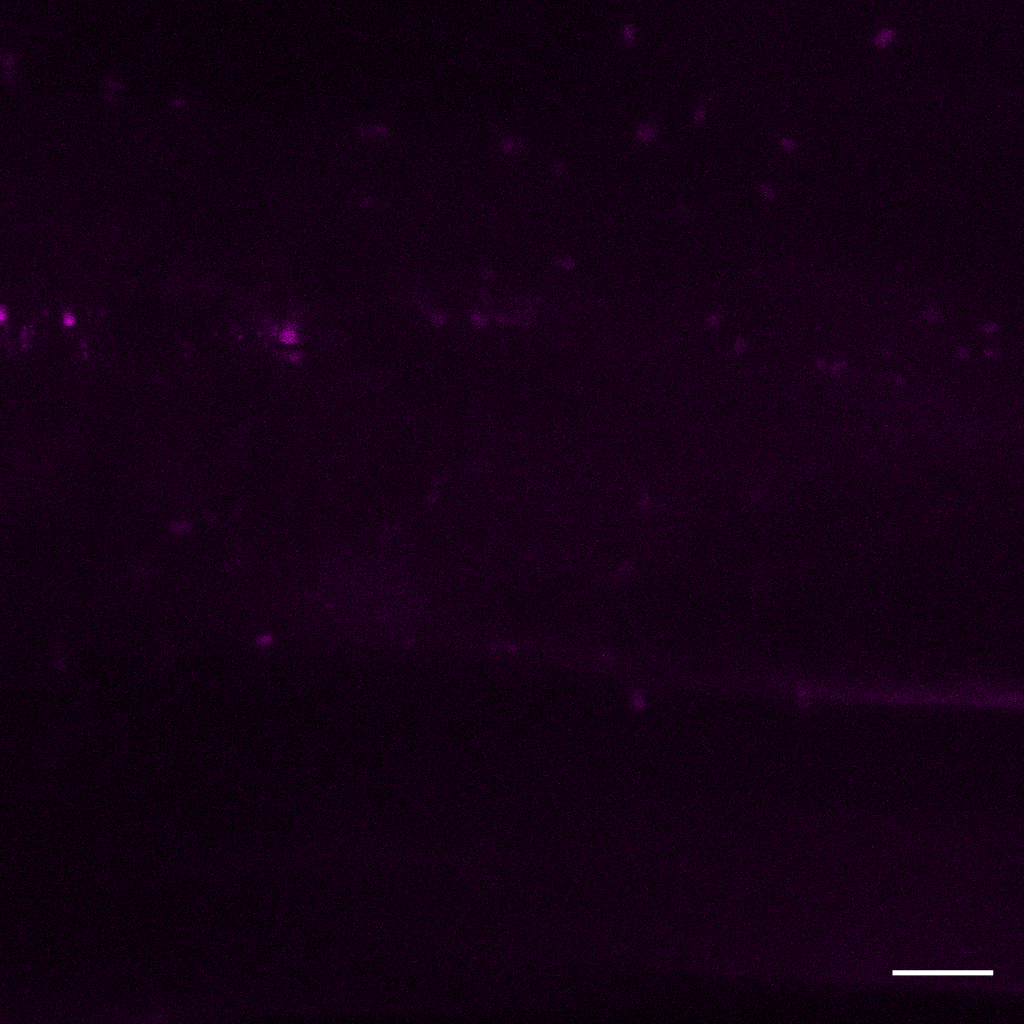

Supplement: Supplementary file 7 — Figure EV1-EV5 Source Data [file 44319_2024_142_MOESM7_ESM.zip › Expanded view figure 2/1,6 hexanediol ARF7-Venus mCherry-ATG8 20 mins mCherry.tif]

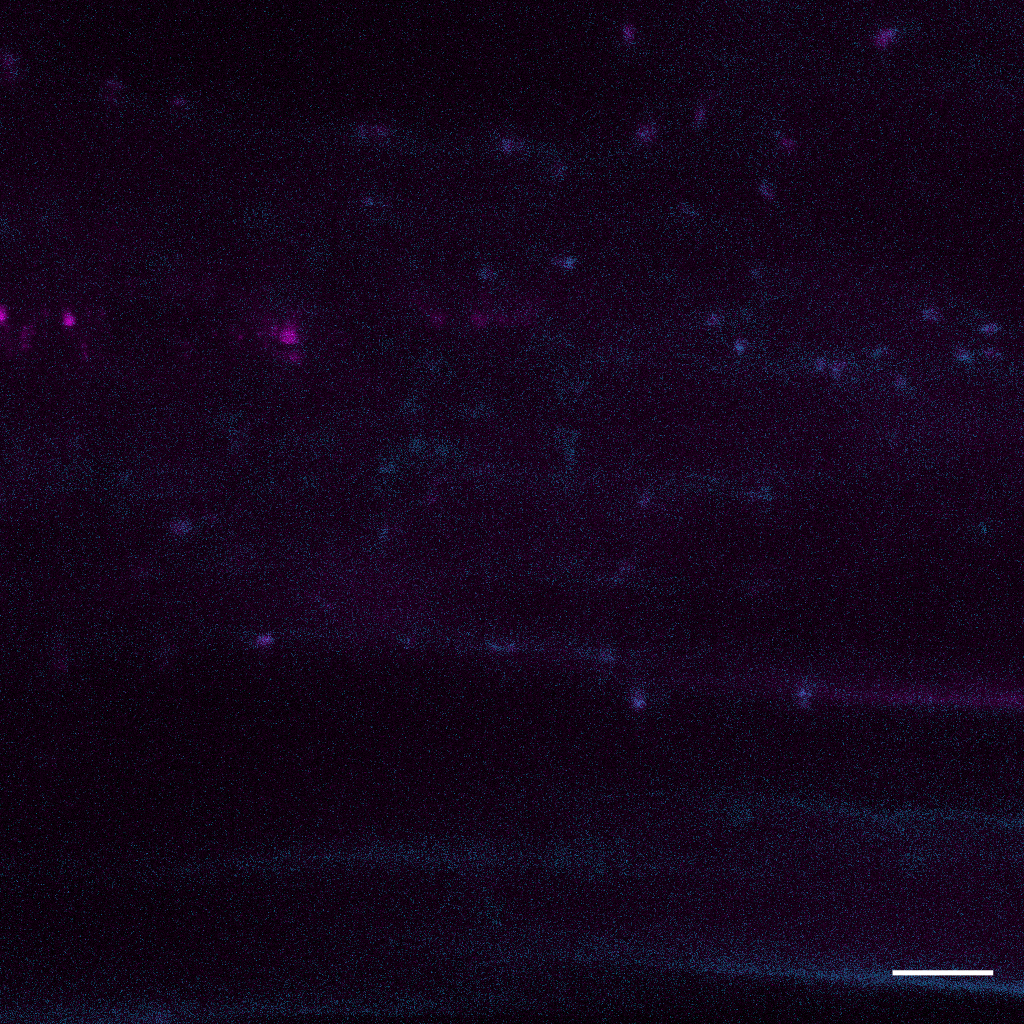

Supplement: Supplementary file 7 — Figure EV1-EV5 Source Data [file 44319_2024_142_MOESM7_ESM.zip › Expanded view figure 2/1,6 hexanediol ARF7-Venus mCherry-ATG8 20 mins merged 1.tif]

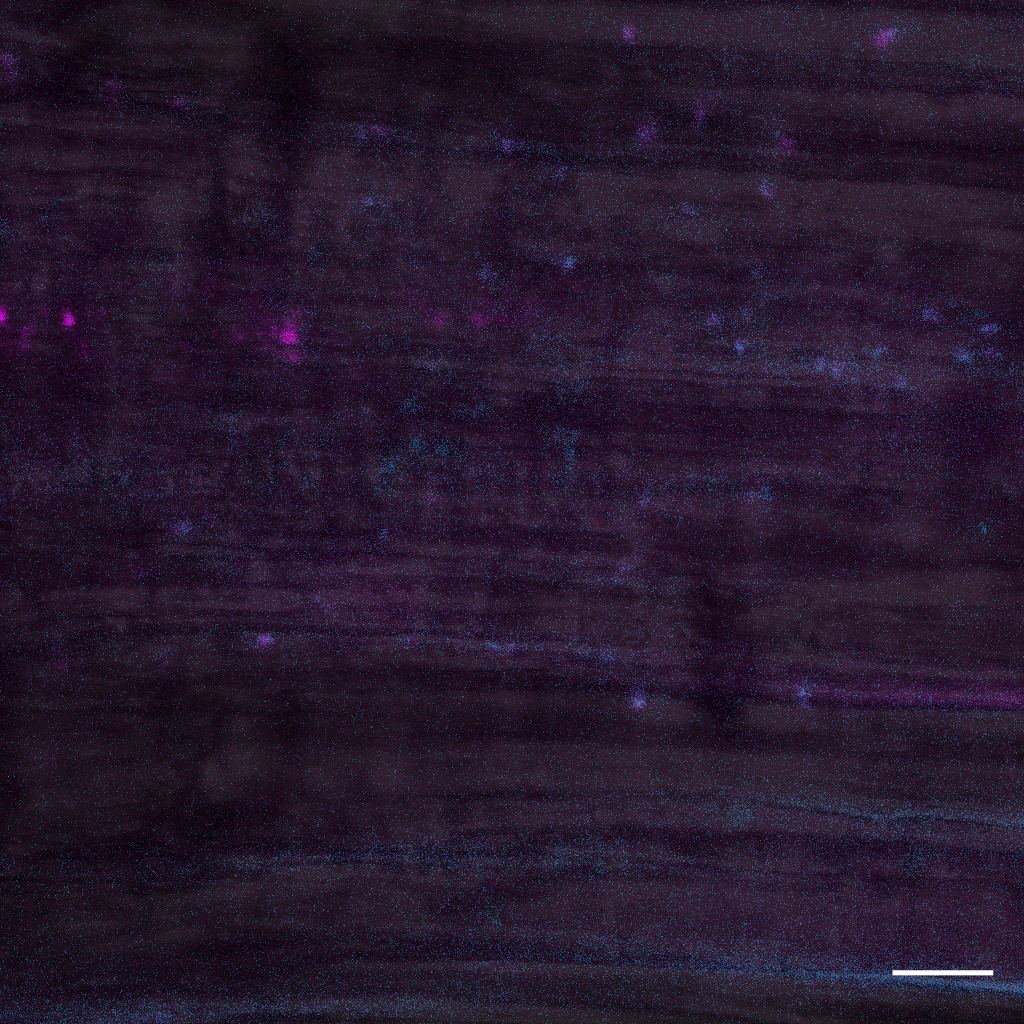

Supplement: Supplementary file 7 — Figure EV1-EV5 Source Data [file 44319_2024_142_MOESM7_ESM.zip › Expanded view figure 2/1,6 hexanediol ARF7-Venus mCherry-ATG8 20 mins merged 2.tif]

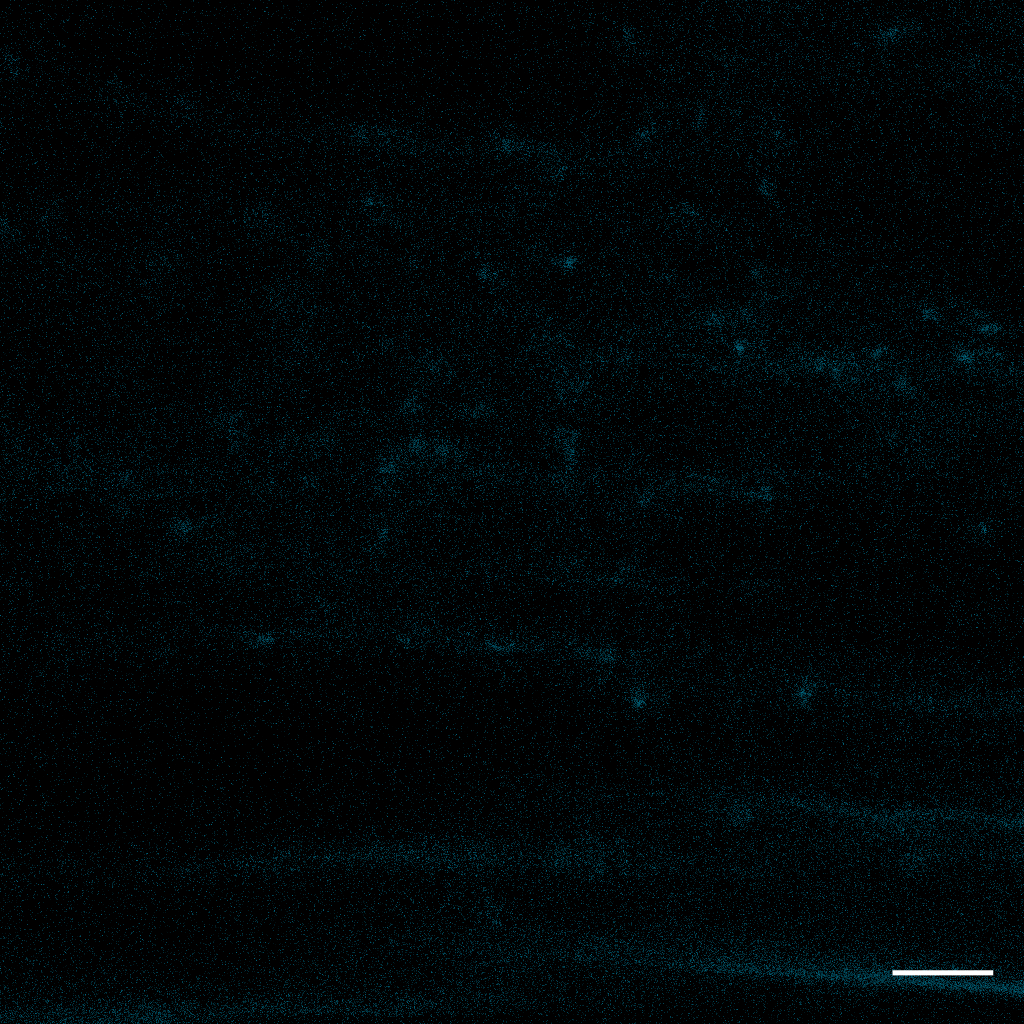

Supplement: Supplementary file 7 — Figure EV1-EV5 Source Data [file 44319_2024_142_MOESM7_ESM.zip › Expanded view figure 2/1,6 hexanediol ARF7-Venus mCherry-ATG8 20 mins YFP.tif]

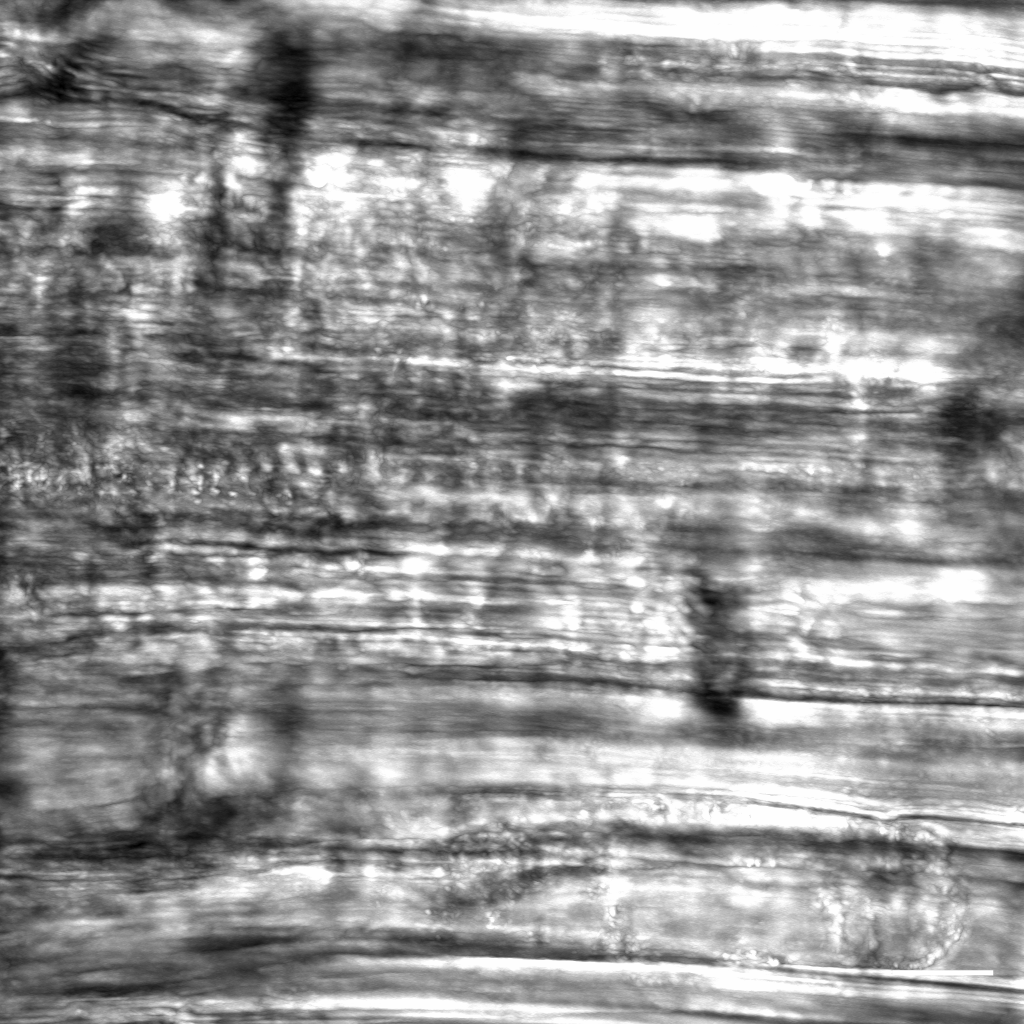

Supplement: Supplementary file 7 — Figure EV1-EV5 Source Data [file 44319_2024_142_MOESM7_ESM.zip › Expanded view figure 2/1,6 hexanediol ARF7-Venus mCherry-ATG8 30 mins Bright-Field.tif]

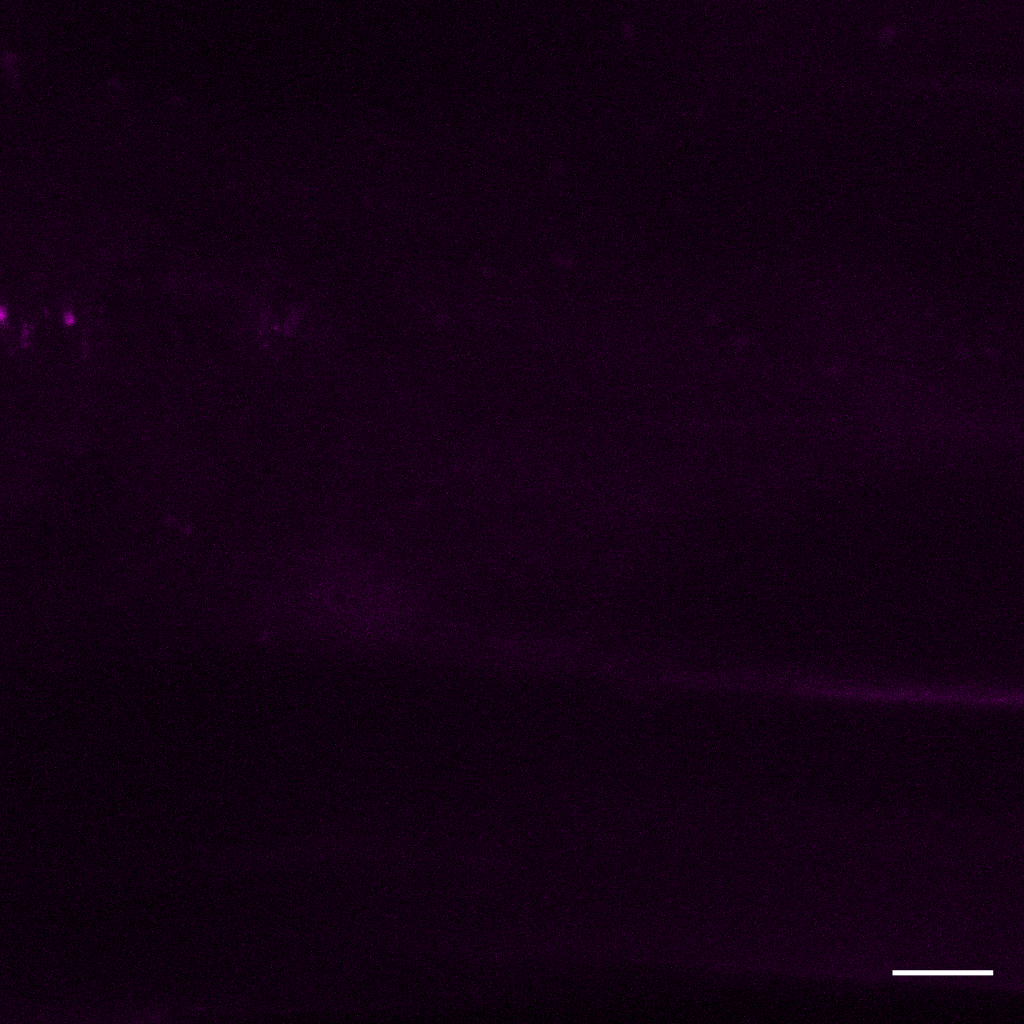

Supplement: Supplementary file 7 — Figure EV1-EV5 Source Data [file 44319_2024_142_MOESM7_ESM.zip › Expanded view figure 2/1,6 hexanediol ARF7-Venus mCherry-ATG8 30 mins mCherry.tif]

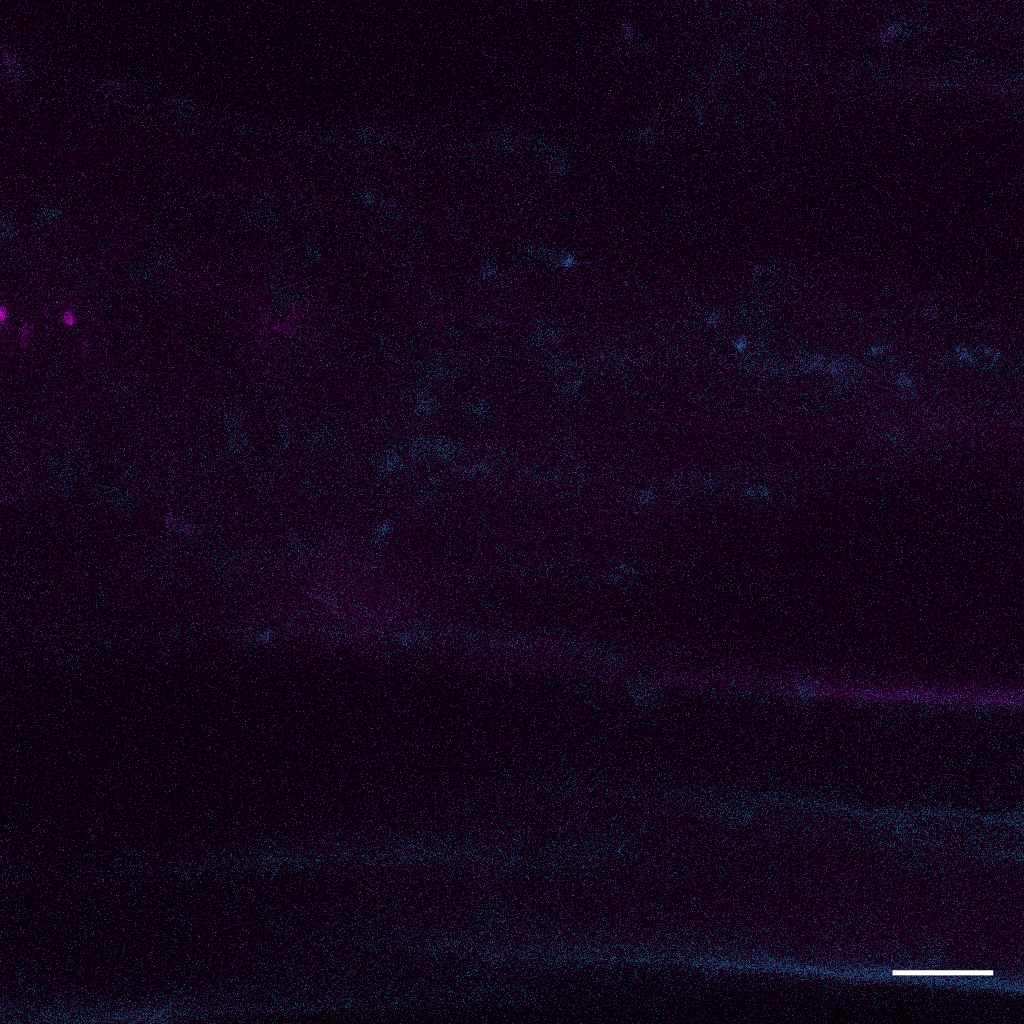

Supplement: Supplementary file 7 — Figure EV1-EV5 Source Data [file 44319_2024_142_MOESM7_ESM.zip › Expanded view figure 2/1,6 hexanediol ARF7-Venus mCherry-ATG8 30 mins merged 1.tif]

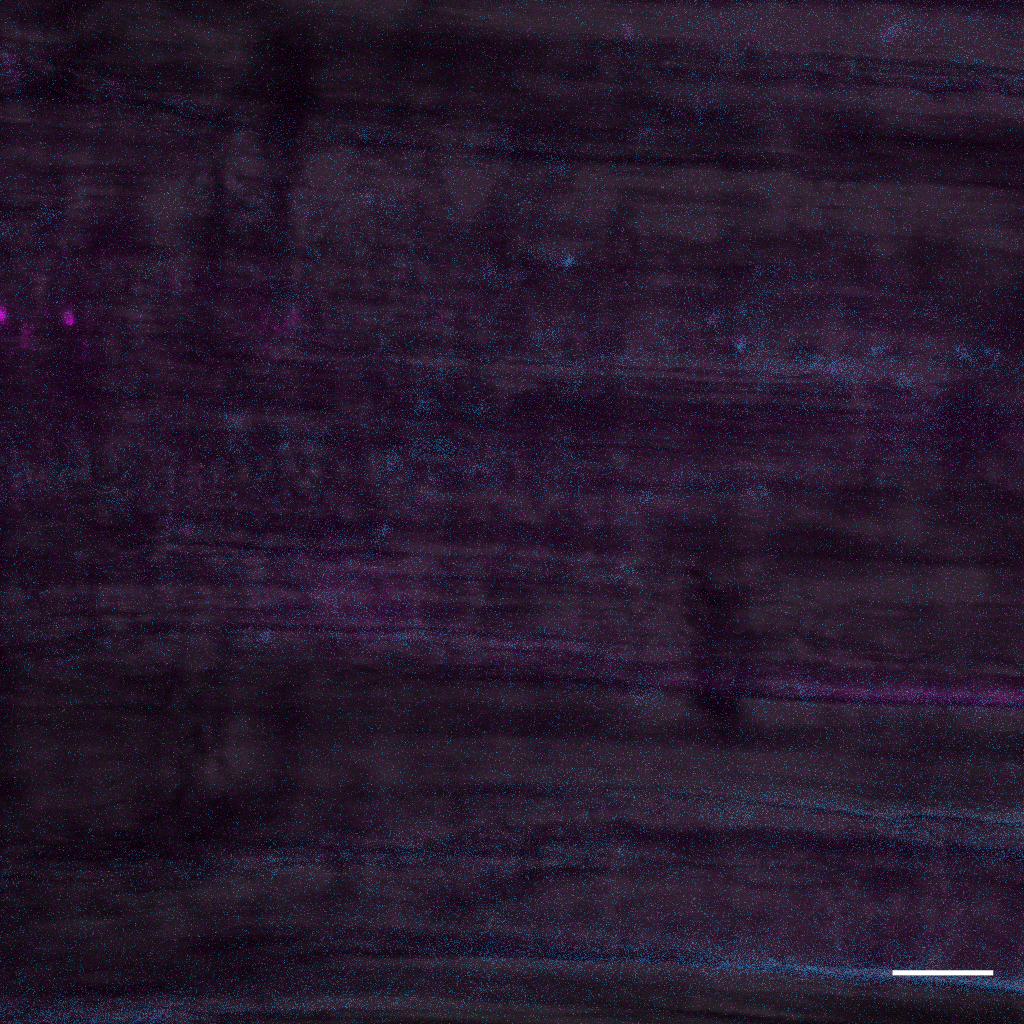

Supplement: Supplementary file 7 — Figure EV1-EV5 Source Data [file 44319_2024_142_MOESM7_ESM.zip › Expanded view figure 2/1,6 hexanediol ARF7-Venus mCherry-ATG8 30 mins merged 2.tif]

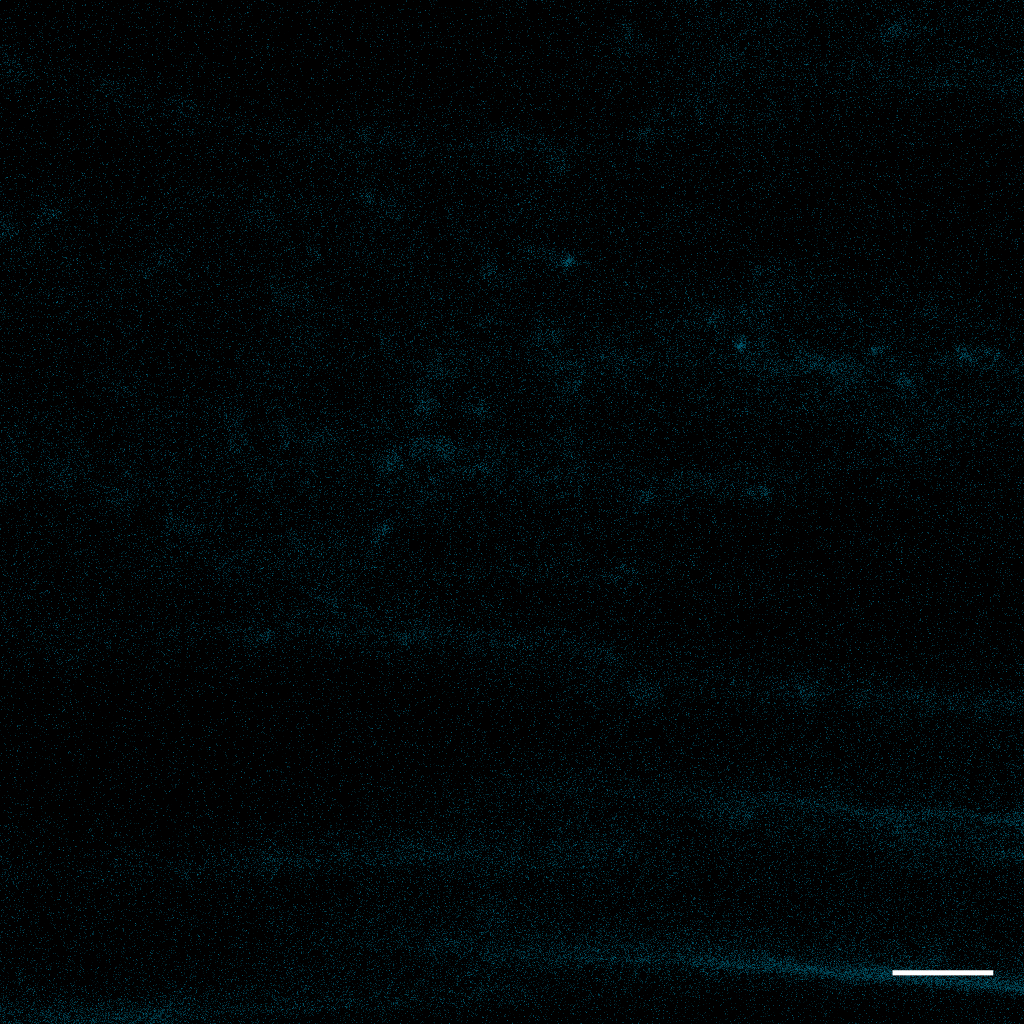

Supplement: Supplementary file 7 — Figure EV1-EV5 Source Data [file 44319_2024_142_MOESM7_ESM.zip › Expanded view figure 2/1,6 hexanediol ARF7-Venus mCherry-ATG8 30 mins YFP.tif]

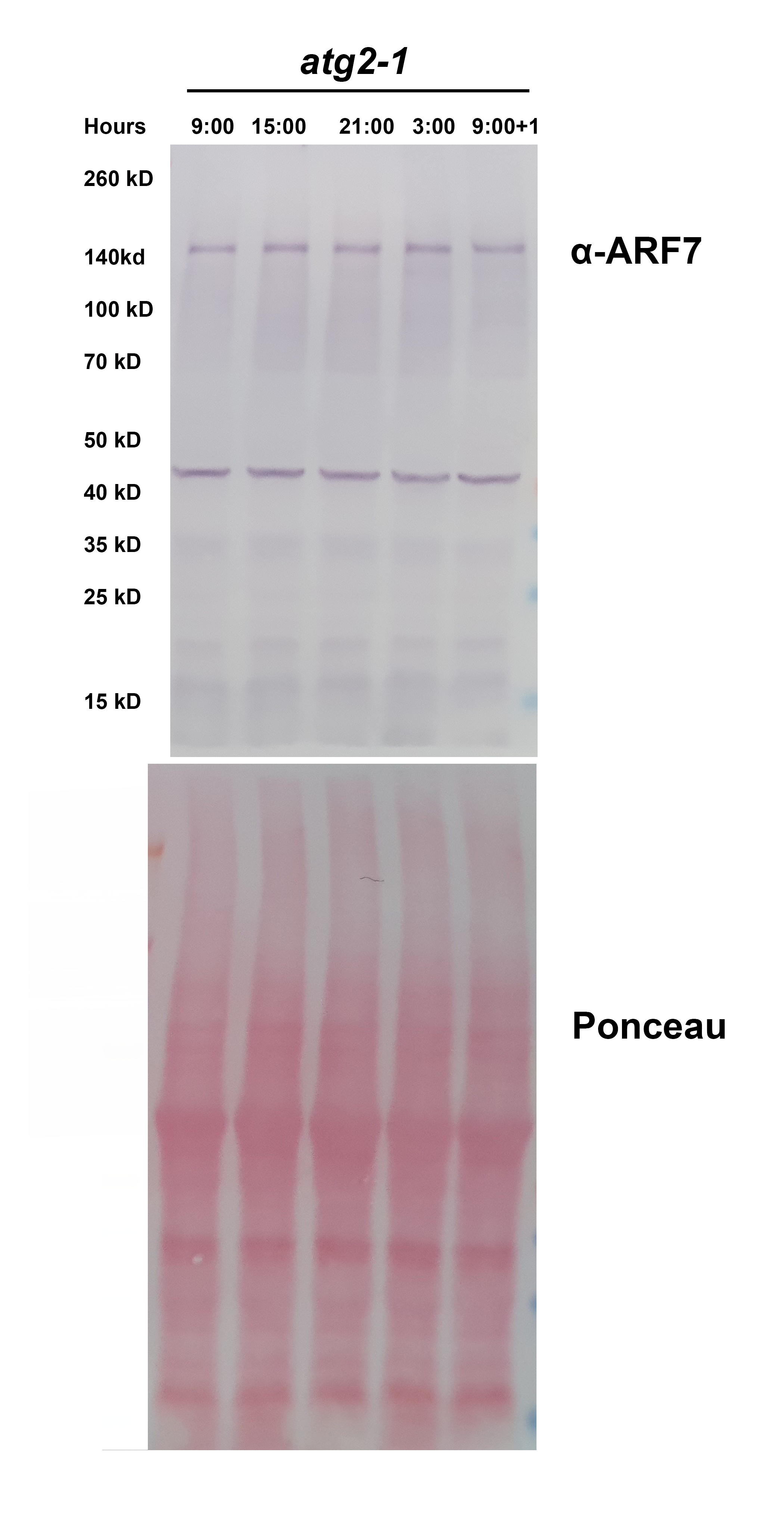

Supplement: Supplementary file 7 — Figure EV1-EV5 Source Data [file 44319_2024_142_MOESM7_ESM.zip › Expanded view figure 3/Replicate 1/Western blot atg2-1 replicate 1.tif]

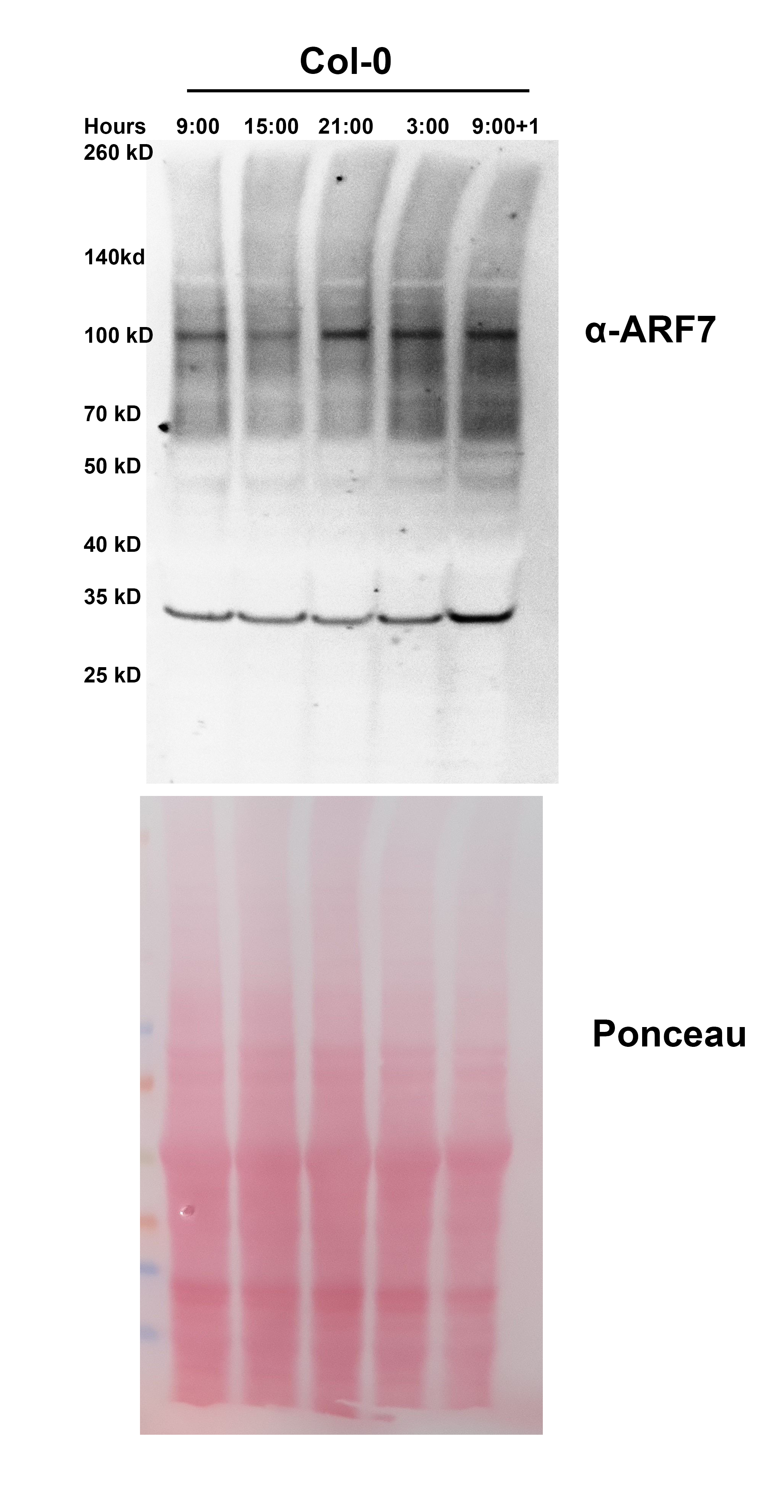

Supplement: Supplementary file 7 — Figure EV1-EV5 Source Data [file 44319_2024_142_MOESM7_ESM.zip › Expanded view figure 3/Replicate 1/Western blot col-0 replicate 1.tif]

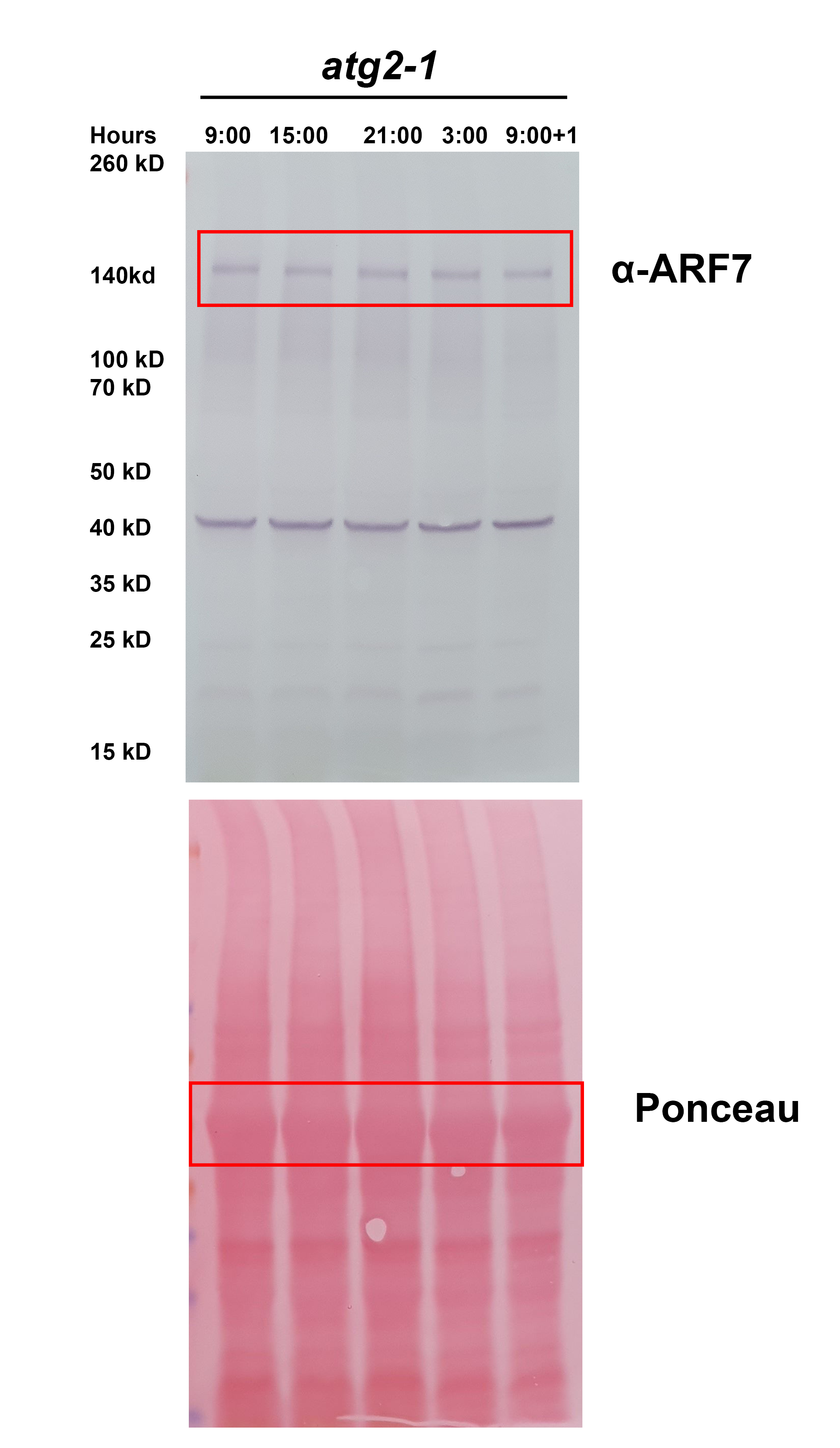

Supplement: Supplementary file 7 — Figure EV1-EV5 Source Data [file 44319_2024_142_MOESM7_ESM.zip › Expanded view figure 3/Western blot atg2.tif]

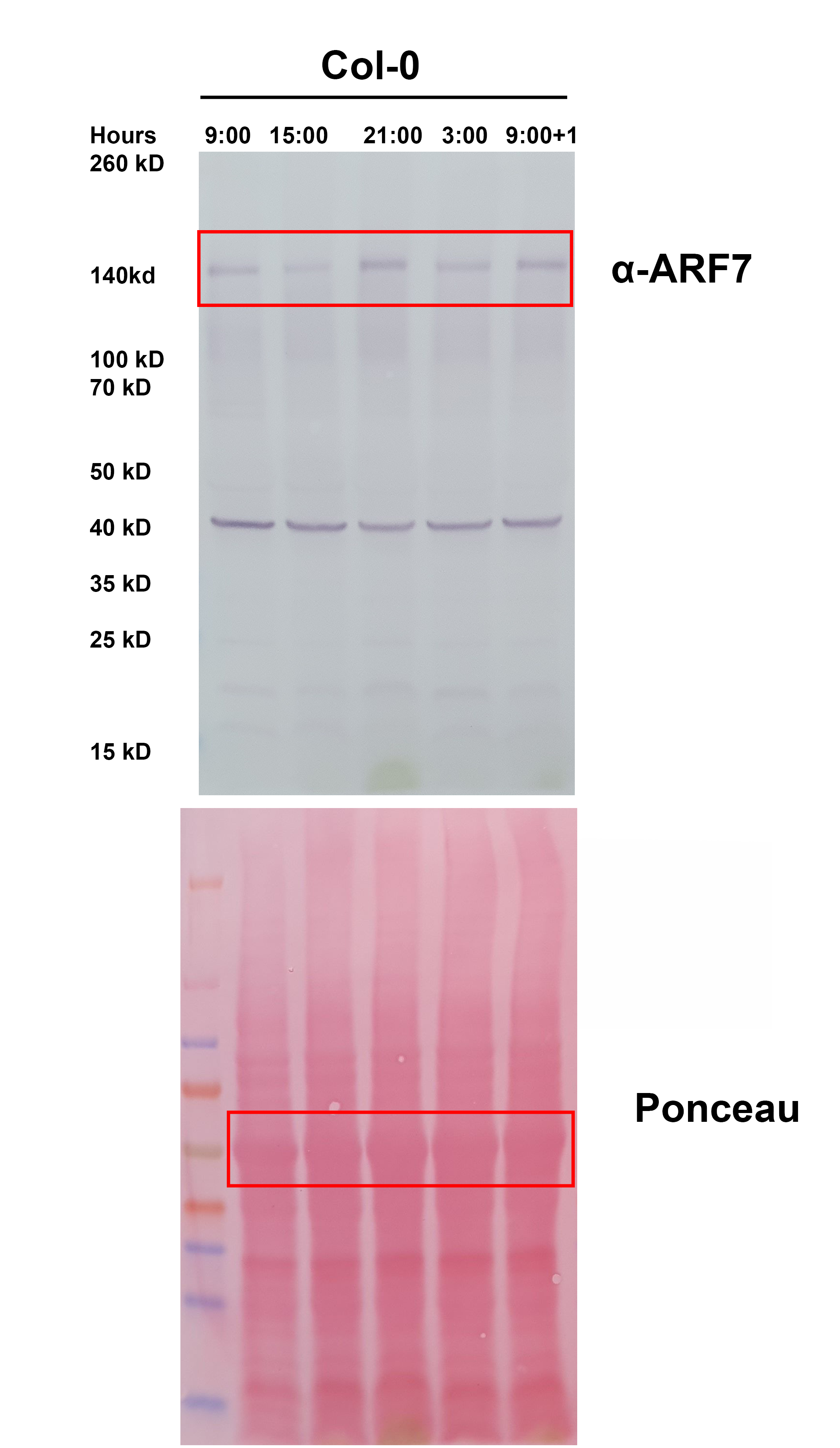

Supplement: Supplementary file 7 — Figure EV1-EV5 Source Data [file 44319_2024_142_MOESM7_ESM.zip › Expanded view figure 3/Western blot col-0.tif]

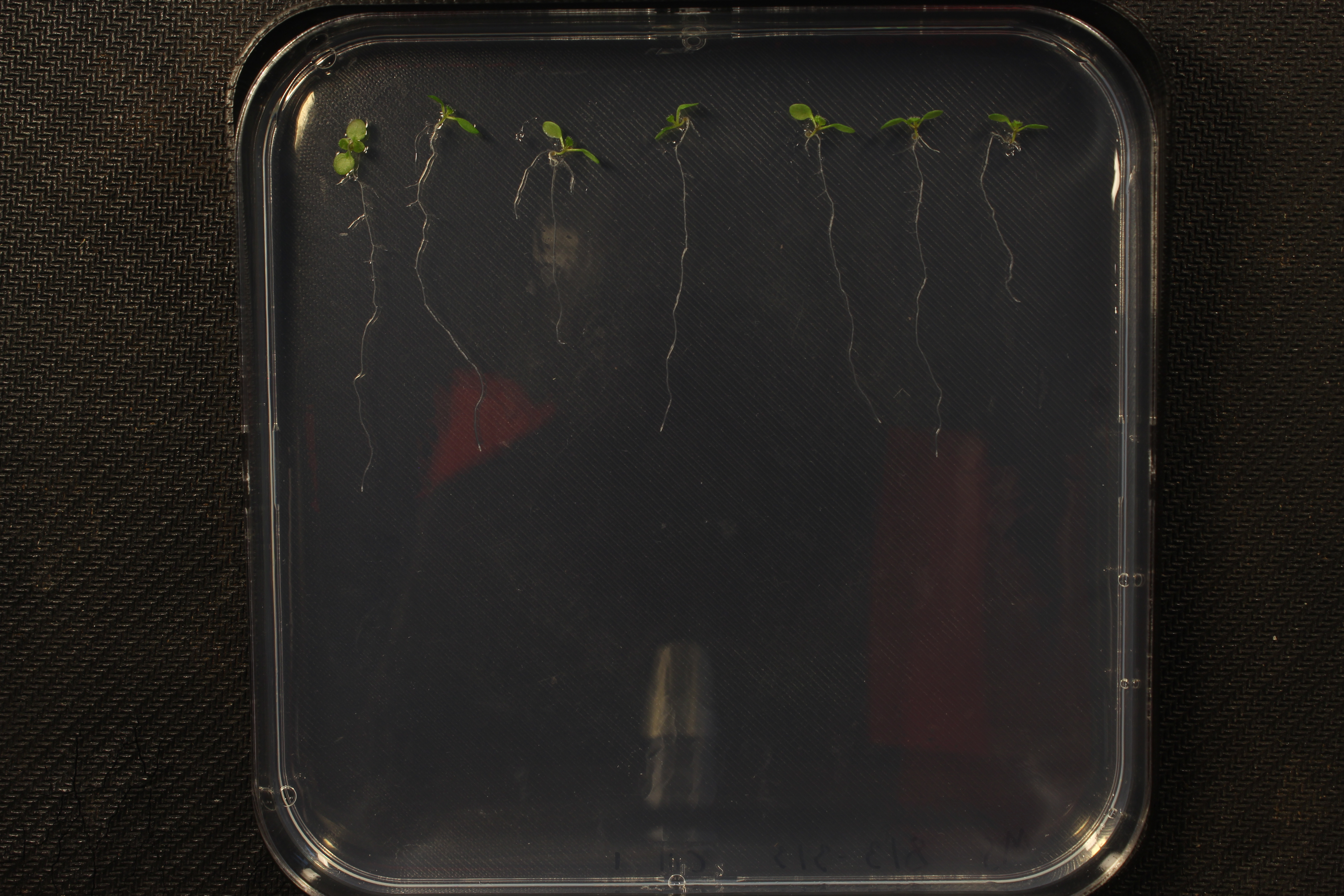

Supplement: Supplementary file 7 — Figure EV1-EV5 Source Data [file 44319_2024_142_MOESM7_ESM.zip › Expanded view figure 5/EV5 A/col ms.JPG]

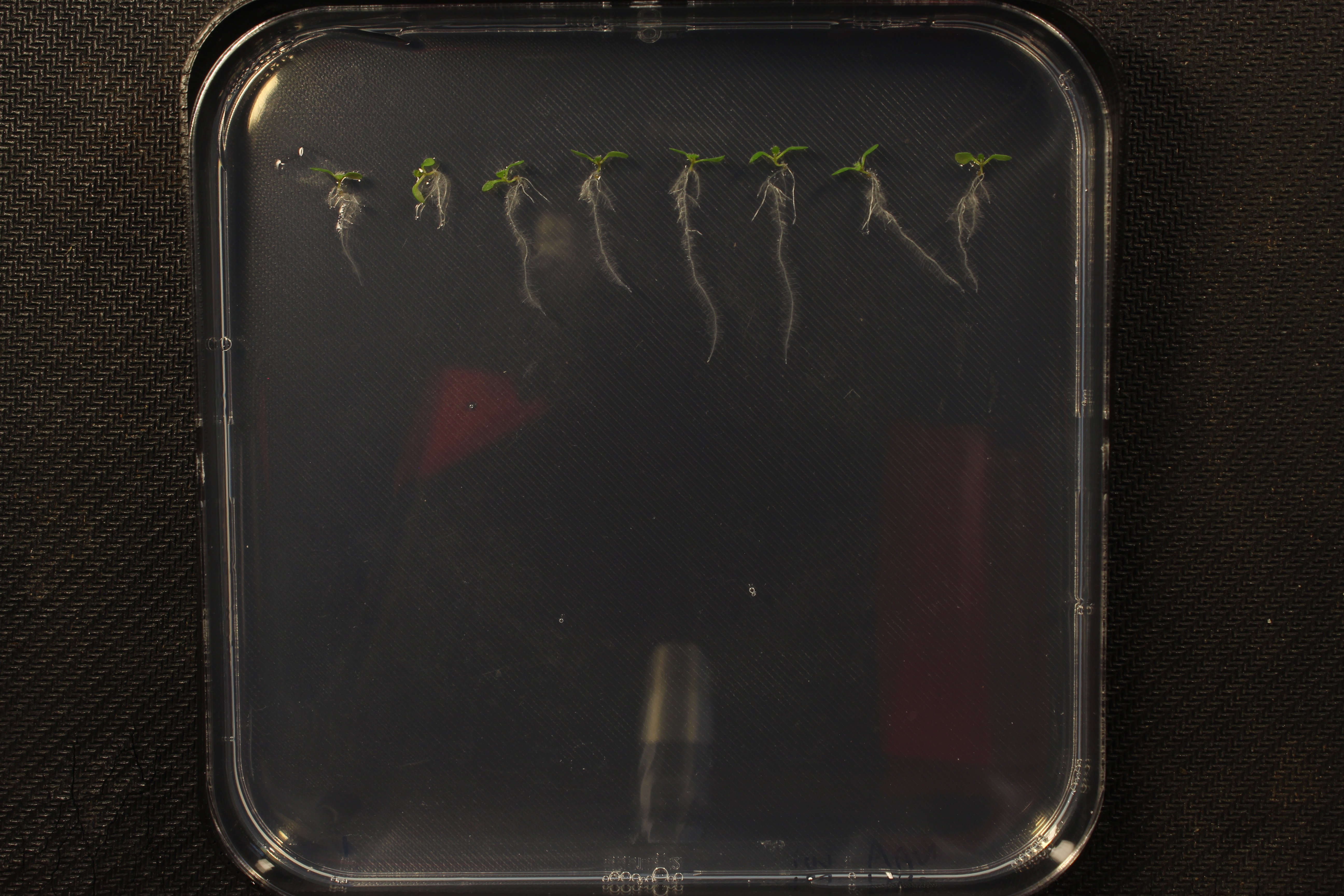

Supplement: Supplementary file 7 — Figure EV1-EV5 Source Data [file 44319_2024_142_MOESM7_ESM.zip › Expanded view figure 5/EV5 A/col naa.JPG]

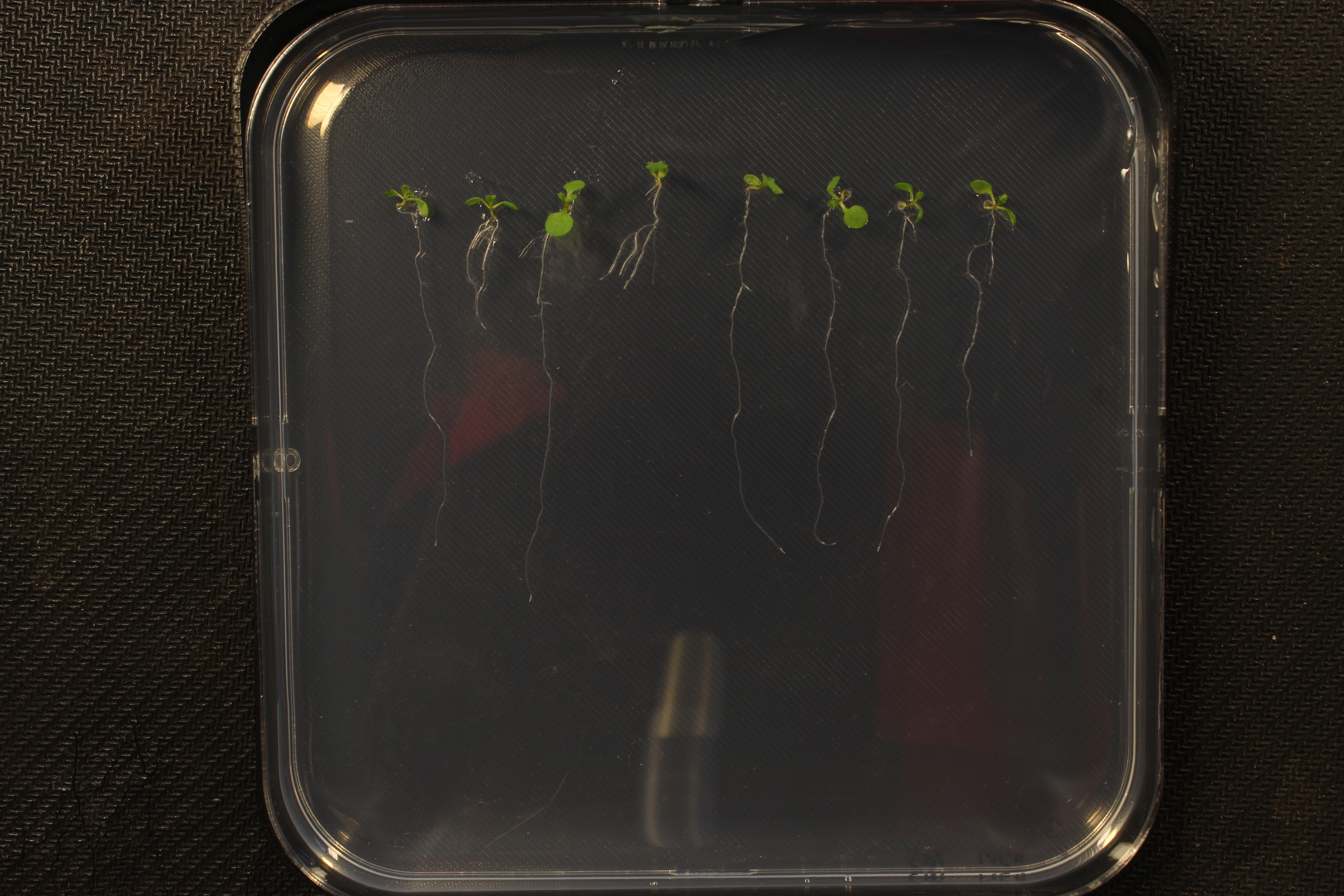

Supplement: Supplementary file 7 — Figure EV1-EV5 Source Data [file 44319_2024_142_MOESM7_ESM.zip › Expanded view figure 5/EV5 A/nbr1 ms.JPG]

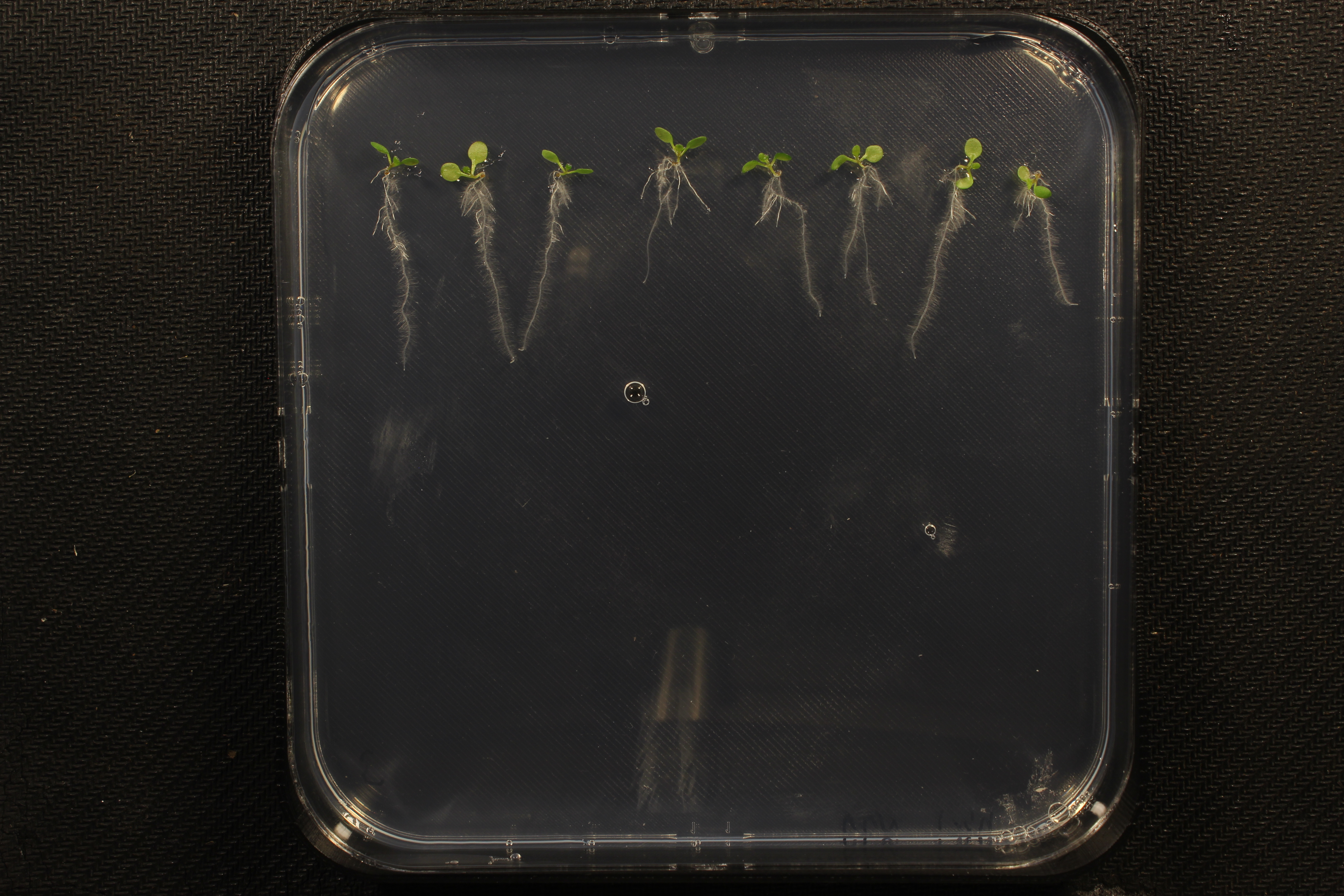

Supplement: Supplementary file 7 — Figure EV1-EV5 Source Data [file 44319_2024_142_MOESM7_ESM.zip › Expanded view figure 5/EV5 A/nbr1 naa.JPG]
