# Supplementary material for: NBR1-mediated selective autophagy of ARF7 modulates root branching
Source: EMBO Rep. 2024 Apr 29;25(6):8. doi: 10.1038/s44319-024-00142-5 (PMC11169494; doi:10.1038/s44319-024-00142-5)
Supplement: Supplementary file 9 — Expanded View Figures [file 44319_2024_142_MOESM9_ESM.pdf]

Expanded View Figures

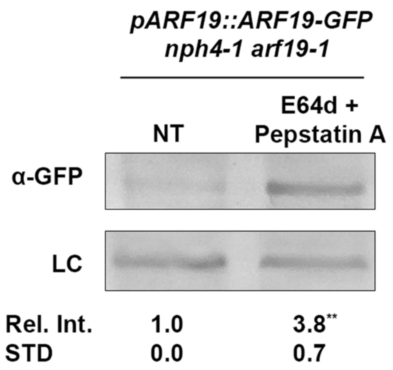

Figure EV1. Autophagy inhibition promotes ARF19 accumulation.

GFP western blot of 14-day-old *pARF19::ARF19-GFP/nph4-1 arf19-1* seedlings grown in MS or MS supplemented with vacuolar protease inhibitors E-64D and Pepstatin A for 24 h. Values below each band represent the ratio between ARF19-GFP and the loading control (LC) normalized to NT (MS+ solvent), which was set to 1. Image is representative of three biological replicates; asterisk depict statistical significance from NT according to a Man–Whitney *U* test ($P \leq 0.01$). Source data are available online for this figure.

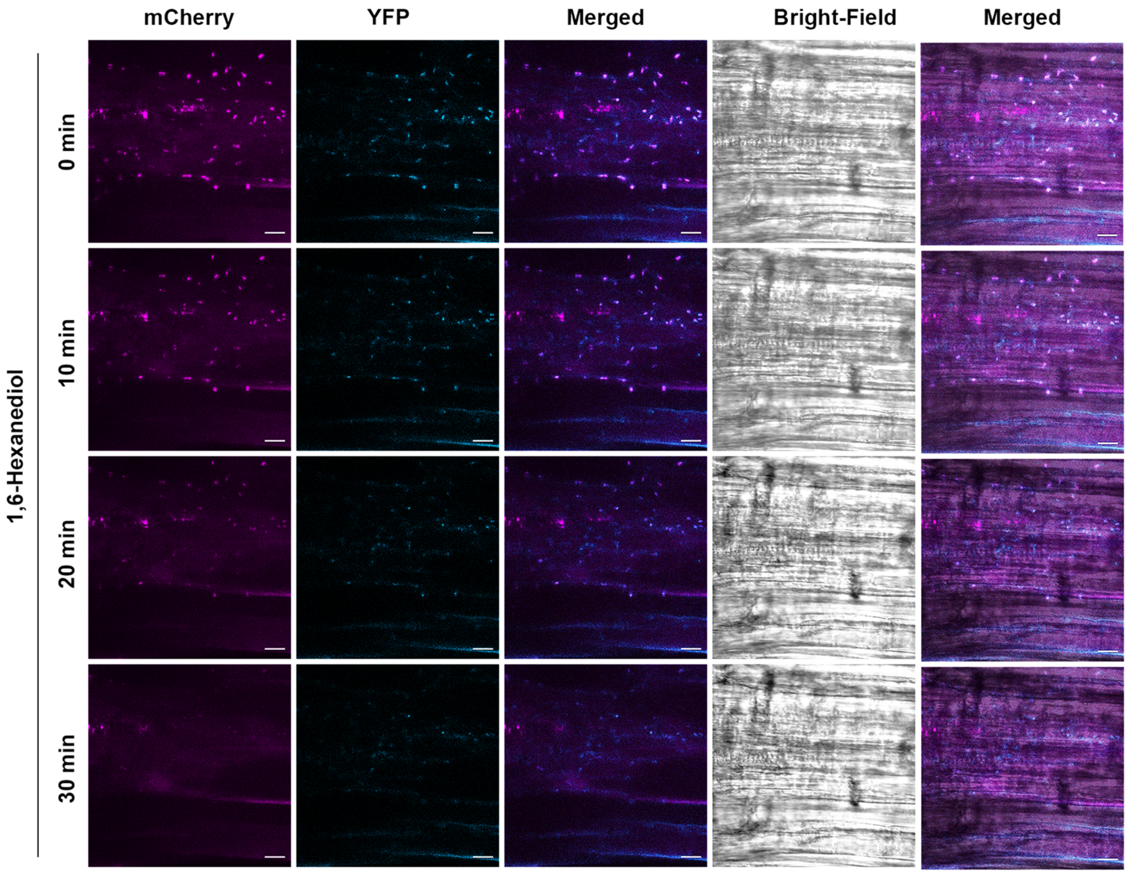

Figure EV2. ARF7 and ATG8 condensates have liquid properties.

pARF7::gARF7-Venus x *mCherry-ATG8a* plants were treated with the LLPS inhibitor 1,6-Hexanediol and imaged at given timepoints. Representative pictures were taken in the maturation zone of 10–14-day old *Arabidopsis* seedlings. Leftmost Merge column represent the merger of YFP and mCherry channels, while the rightmost also includes the T-PMT channel (brightfield). Scale bar 10 μM. Source data are available online for this figure.

 © The Author(s)

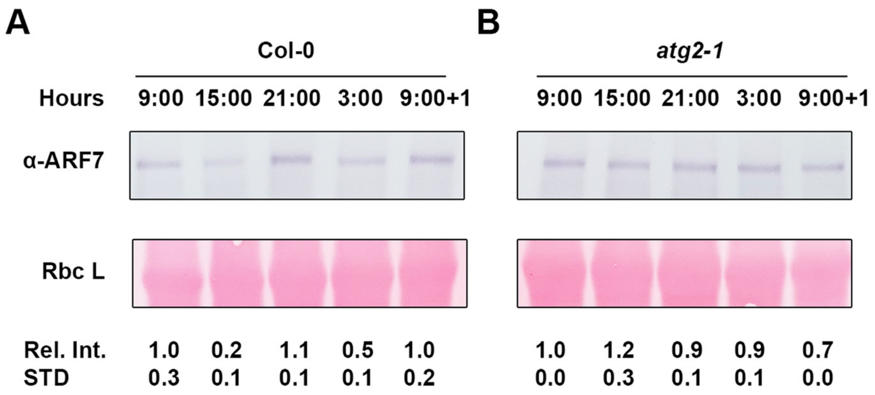

Figure EV3. ARF7 abundance rhythmically fluctuates over 24 h and this regulation is lost in *atg2-1*.

(**A**, **B**) ARF7 western blots from proteins extracted from Col-0 (**A**) and *atg2-1* (**B**) over a 24 h period (09:00 am, 15:00 pm, 21:00 pm, 03:00 am and 09:00 am of the following morning). Values below each band represent the relative intensity ratio between ARF7 and RuBisCO large subunit (Rbc L) as stained with Ponceau S used as loading control. Ratios were normalized to untreated (NT) which was arbitrarily set to 1. The experiment was repeated with similar results. Source data are available online for this figure.

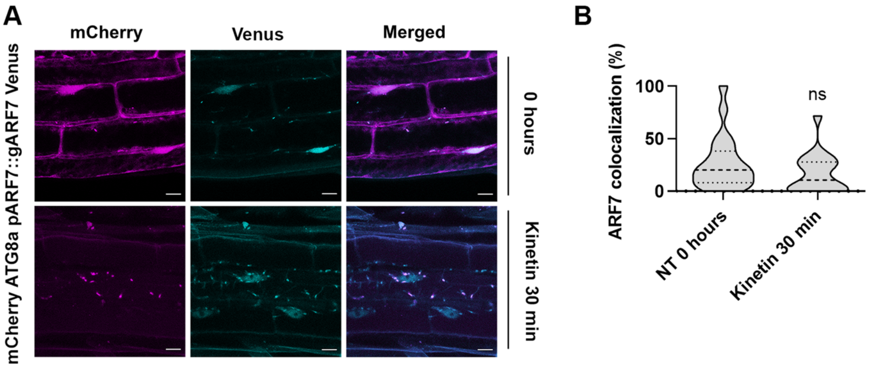

Figure EV4. Cytokinin has no effect on ARF7 co-localization with autophagosomes.

(**A**) Co-localization of ARF7-Venus with mCherry-ATG8a (nuclei and/or cytoplasmic foci) in the maturation zone. MS+ solvent (NT) or 1 μM Kinetin for 30 min were used for the treatment. Pictures were taken in the respective root zone of 10–14-day old *Arabidopsis* seedlings. Scale bar 10 μM. (**B**) Quantification of the co-localization of ARF7-Venus with mCherry-ATG8a. The outline of the violin plots represents the probability of the kernel density. Dotted lines represent interquartile ranges (IQR), with the thick horizontal line representing the median; whiskers extend to the highest and lowest data point. Statistical significance was done according to a Wilcoxon-rank test. At least five plants per conditions were analyzed. Source data are available online for this figure.

 © The Author(s)

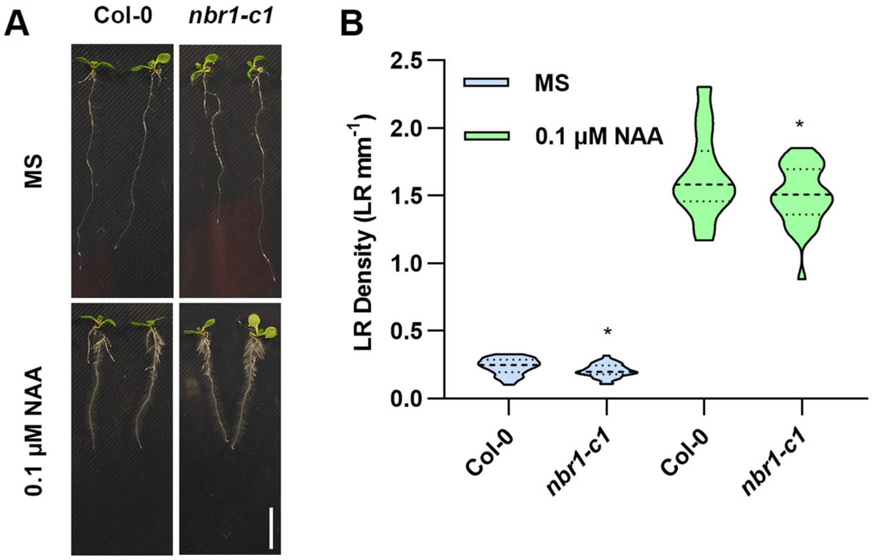

Figure EV5. NBR1 loss-of-function mutants show reduced LR formation.

(**A**, **B**) LR density quantification in Col-0 and nbr1-c1 grown in MS plates supplemented with Solvent (MS) or 0.1 μM NAA. The outline of the violin plots represents the probability of the kernel density. Dotted lines represent interquartile ranges (IQR), with the thick horizontal line representing the median; whiskers extend to the highest and lowest data point but no more than ±1.5 times the IQR from the box. Results were obtained from two independent experiments with at least 25 plants per condition, asterisks mark statistical significance to Col-0 according to the *t* test (*0.05). Scale bar: 1 cm). Source data are available online for this figure.
